# Forty-Three Loci Associated with Plasma Lipoprotein Size, Concentration, and Cholesterol Content in Genome-Wide Analysis

**DOI:** 10.1371/journal.pgen.1000730

**Published:** 2009-11-20

**Authors:** Daniel I. Chasman, Guillaume Paré, Samia Mora, Jemma C. Hopewell, Gina Peloso, Robert Clarke, L. Adrienne Cupples, Anders Hamsten, Sekar Kathiresan, Anders Mälarstig, José M. Ordovas, Samuli Ripatti, Alex N. Parker, Joseph P. Miletich, Paul M. Ridker

**Affiliations:** 1 Donald W. Reynolds Center for Cardiovascular Disease Prevention, Brigham and Women's Hospital, Boston, Massachusetts, United States of America; 2 Division of Preventive Medicine, Brigham and Women's Hospital, Boston, Massachusetts, United States of America; 3 Division of Cardiology, Brigham and Women's Hospital, Boston, Massachusetts, United States of America; 4 PROCARDIS Consortium; 5 Clinical Trial Service Unit, University of Oxford, Oxford, United Kingdom; 6 Department of Biostatistics, Boston University School of Public Health, Boston, Massachusetts, United States of America; 7 Framingham Heart Study, Boston University School of Medicine, Boston, Massachusetts, United States of America; 8 Atherosclerosis Research Unit, Department of Medicine Solna, Karolinska Institutet, Stockholm, Sweden; 9 Broad Institute of Massachusetts Institute of Technology and Harvard University, Cambridge, Massachusetts, United States of America; 10 USDA Human Nutrition Research Center on Aging, Tufts University, Boston, Massachusetts, United States of America; 11 Institute for Molecular Medicine Finland, FIMM, Helsinki, Finland; 12 Amgen, Inc., Cambridge, Massachusetts, United States of America; 13 Amgen, Inc., Thousand Oaks, California, United States of America; University of Michigan, United States of America

## Abstract

While conventional LDL-C, HDL-C, and triglyceride measurements reflect aggregate properties of plasma lipoprotein fractions, NMR-based measurements more accurately reflect lipoprotein particle concentrations according to class (LDL, HDL, and VLDL) and particle size (small, medium, and large). The concentrations of these lipoprotein sub-fractions may be related to risk of cardiovascular disease and related metabolic disorders. We performed a genome-wide association study of 17 lipoprotein measures determined by NMR together with LDL-C, HDL-C, triglycerides, ApoA1, and ApoB in 17,296 women from the Women's Genome Health Study (WGHS). Among 36 loci with genome-wide significance (*P*<5×10^−8^) in primary and secondary analysis, ten (*PCCB/STAG1* (3q22.3), *GMPR/MYLIP* (6p22.3), *BTNL2* (6p21.32), *KLF14* (7q32.2), 8p23.1, *JMJD1C* (10q21.3), *SBF2* (11p15.4), 12q23.2, *CCDC92/DNAH10/ZNF664* (12q24.31.B), and *WIPI1* (17q24.2)) have not been reported in prior genome-wide association studies for plasma lipid concentration. Associations with mean lipoprotein particle size but not cholesterol content were found for LDL at four loci (7q11.23, *LPL* (8p21.3), 12q24.31.B, and *LIPG* (18q21.1)) and for HDL at one locus (*GCKR* (2p23.3)). In addition, genetic determinants of total IDL and total VLDL concentration were found at many loci, most strongly at *LIPC* (15q22.1) and *APOC-APOE* complex (19q13.32), respectively. Associations at seven more loci previously known for effects on conventional plasma lipid measures reveal additional genetic influences on lipoprotein profiles and bring the total number of loci to 43. Thus, genome-wide associations identified novel loci involved with lipoprotein metabolism—including loci that affect the NMR-based measures of concentration or size of LDL, HDL, and VLDL particles—all characteristics of lipoprotein profiles that may impact disease risk but are not available by conventional assay.

## Author Summary

Genome-wide association studies (GWAS) of plasma lipoprotein fractions hold great promise for understanding lipid metabolism and its central role in cardiovascular disease and related disorders. Conventional assays for lipoprotein status determine total cholesterol content of low- or high-density lipoprotein particles (LDL-C or HDL-C, respectively) or total plasma triglyceride content (as an estimate of very-low density lipoprotein particle concentration [VLDL]). All three measures have been targets for recent GWAS. However, a more precise target for GWAS of lipoprotein metabolism would be the concentration of the individual lipoprotein particles according to class (LDL, HDL, VLDL) and size (small, medium, and large), all of which can be measured by NMR-based methods. In a population of 17,296 women of European ancestry from the Women's Genome Health Study, we have performed a GWAS for 22 lipoprotein measures derived from NMR-based and conventional assays. We find 43 genetic loci involved in lipoprotein metabolism, including 10 novel loci. The results offer a clearer picture of common genetic influences on lipoprotein metabolism than available previously, including genetic effects on the distribution of LDL, HDL, and VLDL particle size, as well as on IDL and VLDL particle concentration, neither of which can be assessed by conventional measures.

## Introduction

Standard measures of plasma lipoprotein concentration do not reveal heterogeneity in the size of lipoprotein particles or their content of cholesterol and triglycerides. Yet recognizing this heterogeneity may be essential for understanding qualitative differences in lipid metabolism among individuals. Some reports identify a pattern in the size distribution of lipoprotein sub-fractions as intimately connected with coronary heart disease [1],[2]. Related findings identify a link between lipoprotein profile and metabolic syndrome, and by inference to diabetes [3]. While these observations remain controversial for prognostic use [4], they point to alterations in lipoprotein metabolism in disease.

The variation in particle size and lipid content can be quantified accurately by NMR-based methods that determine lipoprotein particle concentration according to lipid class and particle size. Thus, NMR methods can measure concentration of large and small low density lipoprotein (LDL) particles as well as concentration of the related intermediate density lipoprotein (IDL) particles, and similarly concentration of small, medium, and large high density lipoprotein (HDL) or very low density lipoprotein (VLDL) particles. HDL and LDL particle concentration can also be estimated by chemical measures of apolipoprotein A1 (ApoA1) and apolipoprotein B (ApoB) protein concentration, respectively, but neither these assays nor other standard clinical assays provide information about particle size distribution, and consequently the apportionment of cholesterol and triglycerides to different sized particles. The greater precision in characterizing lipoprotein profiles using NMR-based techniques provides an opportunity for correspondingly greater detail in understanding lipid metabolism, for example by genome-wide genetic analysis, as has been done recently for plasma concentration LDL-C, HDL-C, triglycerides, ApoA1, and ApoB [5]–[13].

## Results

### Genome-wide association analysis of 22 NMR-based and conventional lipoprotein fractions

Among 17,296 WGHS participants with confirmed European ancestry (Table 1), we performed genome-wide association analysis assuming an additive genetic model for 22 plasma lipoprotein measures determined either by NMR methods or by standard clinical assay. On the basis of genome-wide significance (*P*<5×10^−8^), genetic variation at total of 31 loci was associated with at least one of the lipoprotein fractions (Table 2). Thirty of these 31 loci derive from analysis in the whole sample, while the remaining locus was identified with genome-wide significance in a subset of 12,489 (72%) strictly fasting participants, for whom there were small but significant differences in lipoprotein profiles compared with non-fasting participants (Table 1). Nearly all of the associations with genome-wide significance level in the fasting subsample also had genome-wide significance in the larger, better powered whole sample. One exception was the genome-wide significant association with ApoA1 at *ABCA1* (9q31.1), a locus that was identified in the whole sample on the basis of genome-wide significant associations with HDL-C and medium HDL particles but not for ApoA1. The other was an association with mean VLDL size at 8p23.1, a locus that appears only in analysis in the fasting sub-sample (Table 2). These additional associations remain strongly suggestive in the whole sample (*P*<1.6×10^−5^) even though they do not reach genome-wide significance. Statistics for the most significant genome-wide associations with *P*<5×10^−8^ at each of the candidate loci are shown in the Table S1.

**Table 1:** WGHS population.

	whole sample	fasting subsample
N with genotype	17,296	12,489
**Clinical characteristics** *		
age (yrs)∧	53 (49–59)	53 (49–59)
BMI (kg/m^2^)	25 (22–28)	25 (22–28)
smoking (%)	2055 (12)	1508 (12)
+HRT use (%)	7537 (44)	5460 (44)
diabetes (%)	0 (0)	0 (0)
hypertension (%)∧	3943 (23)	2950 (24)
#lipid lowering trt. (%)	0 (0)	0 (0)
**Lipoprotein fractions** *		
LDL large (nmol/l)∧	540 (404–680)	547 (407–685)
LDL small (nmol/l)	650 (399–1008)	646 (393–1010)
LDL mean size (nm)	21 (21–22)	21 (21–22)
IDL total (nmol/l)∧	32 (11–67)	34 (12–71)
LDL total (nmol/l)	1272 (1029–1591)	1274 (1032–1594)
LDL-C assay (mg/dl)∧	121 (100–145)	123 (102–146)
ApoB assay (mg/dl)∧	99 (83–120)	100 (84–121)
HDL total (µmol/l)∧	35 (31–39)	35 (31–40)
HDL large (µmol/l)∧	8 (5–10)	8 (5–10)
HDL medium (µmol/l)	3 (0.8–6.0)	3 (0.7–5.9)
HDL small (µmol/l)∧	24 (20–27)	24 (20–27)
HDL mean size (nm)∧	9 (9–9)	9 (9–9)
HDL-C by NMR (mg/dl)	53 (44–64)	53 (44–64)
HDL-C assay (mg/dl)	52 (44–63)	52 (44–63)
ApoA1 assay (mg/dl)	150 (133–169)	150 (133–169)
VLDL total (nmol/l)	69 (50–90)	68 (49–91)
VLDL large (nmol/l)∧	1 (0.4–3.7)	1 (0.3–3.5)
VLDL medium (nmol/l)∧	21 (11–32)	20 (11–32)
VLDL small (nmol/l)∧	45 (33–58)	45 (33–58)
VLDL mean size (nm)∧	47 (42–52)	46 (42–51)
TG by NMR (mg/dl)∧	109 (82–146)	107 (81–144)
TG assay (mg/dl)∧	117 (83–172)	113 (81–166)

*: Clinical characteristics are given as number (fraction) or median (interquartile range). Lipoprotein measures are given as median (interquartile range).

**∧:** p<0.001 for comparison of non-fasting (N = 4,807) fasting (N = 12,489) samples.

**+:** HRT is abbreviation for hormone replacement therapy.

# lipid lowering treatment.

**Table 2:** Loci and candidate genes with genome-wide significant associations (p<5×10^−8^) for 22 lipoprotein measures.

locus	whole sample	fasting subsample	candidate gene(s)
1p32.3	APOB, LDL-C, LDL:T, LDL:L, TG:N, VLDL:T, VLDL:S	APOB, LDL-C, LDL:T, LDL:L, VLDL:T, VLDL:S	*PCSK9*
1p31.3	TG:N, VLDL:T, VLDL:M, VLDL:S	TG:N, VLDL:T, VLDL:M, VLDL:S	*ANGPTL3*
1p13.3	APOB, LDL-C, LDL:T, LDL:L, LDL:S, VLDL:T, VLDL:S	APOB, LDL-C, LDL:T, LDL:S, VLDL:S	*CELSR2*, *PSRC1*, *SARS*, *SORT1*
1q23.3	HDL:M	HDL:M	*APOA2*
2p24.1	APOB, LDL-C, LDL:T, LDL:L, LDL:Z, TG:N, TG, VLDL:T, VLDL:M, VLDL:S, VLDL:Z	APOB, LDL-C, LDL:T, LDL:L, TG:N, TG, VLDL:T, VLDL:M, VLDL:S, VLDL:Z	*APOB*
2p23.3	APOA1, APOB, HDL:T, HDL:S, HDL:Z, IDL:T, LDL:T, LDL:S, LDL:Z, TG:N, TG, VLDL:L, VLDL:T, VLDL:M, VLDL:Z	APOA1, APOB, HDL:T, HDL:S, HDL:Z, IDL:T, LDL:T, LDL:S, LDL:Z, TG:N, TG, VLDL:L, VLDL:T, VLDL:M, VLDL:Z	*GCKR*
2p21	APOB, LDL-C	LDL-C	*ABCG5/8*
2q24.3	HDL-C	-	*COBLL1*, *GRB14*
**3q22.3**	**HDL:S**	-	***PCCB***, ***STAG1***
5q13.3	LDL-C, LDL:L	LDL-C, LDL:L	*HMGCR*
**6p21.32**	**TG:N**, **VLDL:L**	**TG:N**, **VLDL:L**	***BTNL2***,***HLA-DRA***, ***HLA-DRB5***
7q11.23	HDL:S, LDL:L, LDL:S, LDL:Z, TG:N, TG, VLDL:L, VLDL:T, VLDL:M	TG:N, TG, VLDL:M	*MLXIPL*
**7q32.2**	**HDL:Z**, **LDL:T**, **LDL:S**, **TG**	**-**	***COPG2***, ***KLF14***, ***TSGA13***
**8p23.1**	**-**	**VLDL:Z**	***intergenic PPP1R3B***
8p21.3	APOA1, HDL-C, HDL:L, HDL:Z, LDL:L, LDL:S, LDL:Z, HDL:N, TG:N, TG, VLDL:L, VLDL:T, VLDL:M, VLDL:S	APOA1, HDL-C, HDL:L, HDL:Z, LDL:L, LDL:S, LDL:Z, HDL:N, TG:N, TG, VLDL:L, VLDL:T, VLDL:M, VLDL:S	*LPL*
8q24.13	APOB, LDL:T, LDL:S, LDL:Z, TG:N, TG	APOB, LDL:T, LDL:S	*TRIB1*
9q31.1	HDL-C, HDL:M	APOA1, HDL-C	*ABCA1*
9q34.2	LDL-C, LDL:L, VLDL:S, VLDL:Z	LDL-C, VLDL:S	*ABO*
11q12.2	HDL:L, HDL:M, HDL:Z, LDL:L	HDL:L, HDL:M, HDL:Z, LDL:L	*FADS1-3*
11q23.3	APOA1, APOB, HDL-C, HDL:T, HDL:S, LDL:T, LDL:S, LDL:Z, HDL:N, TG:N, TG, VLDL:L, VLDL:T, VLDL:M, VLDL:S	APOA1, APOB, HDL-C, HDL:T, HDL:S, LDL:T, LDL:S, LDL:Z, HDL:N, TG:N, TG, VLDL:L, VLDL:T, VLDL:M, VLDL:S	*APOA1-A5*
**12q23.2**	**HDL:T**, **HDL:N**	**-**	***intergenic ASCL1***, ***PAH***
12q24.31.A	LDL-C	-	*HNF1A/TCF1*
**12q24.31.B**	**HDL:L**, **HDL:Z**, **LDL:T**, **LDL:S**, **LDL:Z**, **TG**	**HDL:L**	***CCDC92***, ***DNAH10***, ***ZNF664***
15q22.1	APOA1, HDL-C, HDL:L, HDL:M, HDL:S, HDL:Z, IDL:T, LDL:L, LDL:S, LDL:Z, HDL:N	APOA1, HDL-C, HDL:L, HDL:S, HDL:Z, IDL:T, LDL:L, LDL:S, LDL:Z, HDL:N	*LIPC*
16q13	APOA1, HDL-C, HDL:T, HDL:L, HDL:Z, IDL:T, LDL:T, LDL:L, LDL:S, LDL:Z, HDL:N, TG, VLDL:T, VLDL:S	APOA1, HDL-C, HDL:T, HDL:L, HDL:Z, IDL:T, LDL:T, LDL:L, LDL:S, LDL:Z, HDL:N, VLDL:T, VLDL:S	*CETP*
**17q24.2**	**HDL:M**	**-**	***PRKAR1A***, ***WIPI1***
18q21.1	APOA1, HDL-C, HDL:L, HDL:Z, LDL:L, LDL:Z, HDL:N	APOA1, HDL-C, HDL:L, HDL:Z, LDL:L, LDL:Z, HDL:N	*LIPG*
19p13.2	APOB, LDL-C, LDL:T, LDL:L, VLDL:S	APOB, LDL-C, LDL:T, LDL:L, VLDL:S	*LDLR*
19q13.32	APOA1, APOB, HDL-C, HDL:M, LDL-C, LDL:T, LDL:L, LDL:S, LDL:Z, HDL:N, TG:N, TG, VLDL:L, VLDL:T, VLDL:M, VLDL:S	APOA1, APOB, HDL-C, HDL:M, LDL-C, LDL:T, LDL:L, LDL:S, HDL:N, TG:N, TG, VLDL:L, VLDL:T, VLDL:S	*APOC1*,*2-APOE*
20q13.12.A	APOA1	-	*HNF4A*
20q13.12.B	HDL-C, HDL:T, HDL:L, HDL:S, HDL:Z, LDL:T, LDL:L, LDL:S, LDL:Z, TG	HDL-C, HDL:T, HDL:L, HDL:S, HDL:Z, LDL:L, LDL:S, LDL:Z, TG	*PLTP*

LDL = low density lipoprotein, IDL = intermediate density lipoprotein, HDL = high density lipoprotein, VLDL = very low density lipoprotein, APOB = apolipoprotein B, APOA1 = apolipoprotein A1.

X:L = large particles, X:M = medium particles, X:S = small particles, X:Z = mean particle size, X:T total particles, X:N = assay by NMR, X-C = cholesterol.

Bold type face indicates novel loci.

Seven of the 31 unique loci reveal novel genome-wide significant associations with the plasma lipoprotein fractions (see bold font type, Table 2). The associations at 3q22.3 (*PCCB/STAG1*), 6p21.32 (*BTNL2*), 7q32.2 (*KLF14*), 12q24.31.B (*CCDC9/DNAH10/ZNF664*) and 17q24.2 (*WIPI1*) are all near genes (Figure S1), while genome-wide significant associations at the remaining two novel loci, 8p23.1 and 12q23.2 are remote (i.e. >150kb) from known genic regions. Among the standard clinical measures LDL-C, HDL-C, and triglycerides only, novel genome-wide loci were found at *KLF14* (7q32.2) and *CCDC9/DNAH10/ZNF664* (12q24.31.B), both for triglycerides. The association at the novel locus 8p23.1 (which differentiated the fasting sample from the whole sample on the basis of mean VLDL particle size) is over 1.8 Mb from a recently described association at 8p23.1 between SNP rs7819412 and triglycerides [6].

The remaining 24 unique loci suggested genes recognized for a diversity of roles in lipid metabolism, broadly defined (Figure S1). Thus, SNPs with genome-wide significance, were confirmed in or near *PCSK9* (at 1p32.3), *APOA2* (1q23.3), *APOB* (2p24.1), *ABCG5/8* (2p21), *HMGCR* (5q13.3), *LPL* (8p21.3), *APOA1-A5* (11q23.3), *ABCA1* (9q31.1), *FADS1-3* (11q12.2), *LIPC* (15q22.1), *CETP* (16q13), *LIPG* (18q21.1), *LDLR* (19p13.2), the *APOC-APOE* complex (19q13.32), and *PLTP* (20q13.12). Similarly, association at 9q34.2 implicating the *ABO* gene recapitulates and extends the known association between blood group antigen and total cholesterol [14],[15]. Less well characterized genic regions, which nonetheless have been validated recently for roles in lipid metabolism, were confirmed for *ANGPTL3* (1p31.3), *CELSR2/MYBPHL/PSRC1/SORT1* (1p13.3), *GCKR* (2p23.3), *MLXIPL* (7q11.23), and *TRIB1* (8q24.13), *HNF1A* (12q24.31.A), and *HNF4A* (20q13.12). The association at *COBLL1/GRB14* (2q24.3) with HDL-C was recently described elsewhere in this same cohort and validated by replication [16]. The previous study found much stronger association in women than men, suggesting a potential interaction with gender. At this locus, the gene *GRB14* is thought to inhibit receptors in the insulin receptor class [17],[18]. The current analysis extends associations at this locus to concentrations of LDL, HDL, and VLDL particles according to size (Table S1).

**Figure 1:**
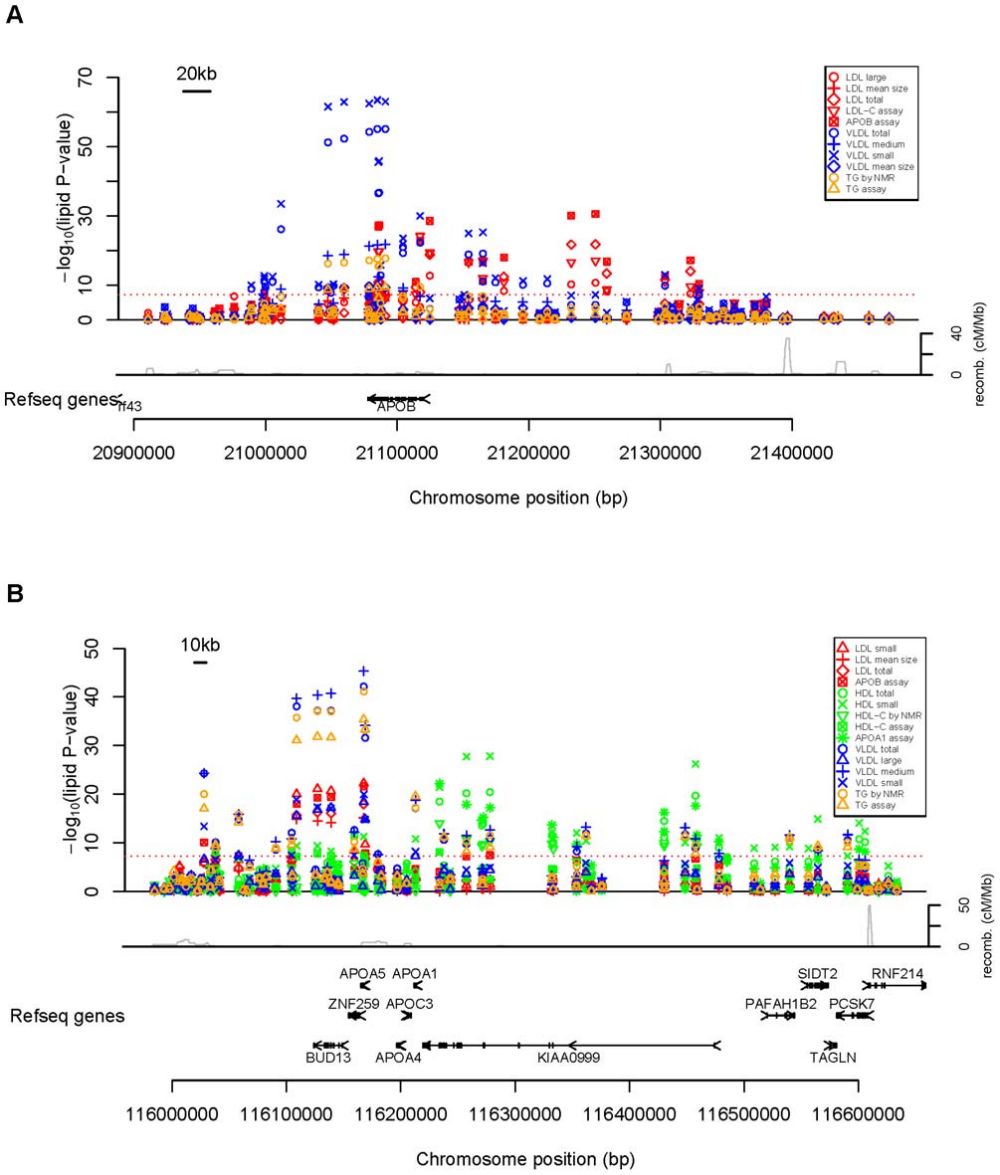
Loci with distinct classes of SNP associations among lipoprotein fractions with genome-wide significance. (A) APOB locus (2p24.1), (B) APOA1-A5 locus (11q23.3). Recombination rates are from [41].

Consistent with a high degree of correlation among the lipoprotein measures (Table S2), the rank order by p-value among the highly significant SNPs was similar for each measure with at least one genome-wide significant association (Figure S1). A notable exception was the *APOB* gene (2p24.1), where the ordering of the p-values, conditional analysis, and patterns of linkage disequlibrium (LD) among the top SNPs (Table S1) revealed three classes of associations. One class included VLDL-related fractions, triglycerides, and mean LDL size for which either rs673548 or rs676210 (LD r^2^ = 1.0) had the strongest association; a second class included ApoB, large LDL particles, and total LDL particles for which either rs1713222 or rs506585 (LD r^2^ = 0.5) had the strongest association; and a final class including only LDL-C for which rs137117 was most strongly associated (Figure 1A). Between SNPs in different classes, maximum LD ranged from r^2^ = 0.04–0.11. Similarly, at *APOA5-APOA1* (11q23.3), p-values revealed two classes of associations seemingly segregating between effects nearer the *APOA5* gene involving triglycerides and effects nearer the *APOA1* gene involving HDL related lipoprotein fractions (Figure 1B).

Large, well-characterized cohorts with NMR-based measurement of lipoprotein fractions are scant, but sub-samples of about 2700 participants in the Framingham Heart Study Offspring cohort (FHS) [19] and about 2000 total CHD cases and controls from PROCARDIS [20] had both the NMR-based lipoprotein measures and genome-wide genetic data already determined. Among all candidate loci, concordance of direction of effects was observed respectively at 124 out of 146 (84%) [84% in fasting sub-sample] and 125 out of 133 (94%) [99% in fasting sub-sample] of the candidate associations for which there was genotype information in FHS and PROCARDIS (Table S3 [whole WGHS sample candidates], Table S4 [fasting WGHS subsample candidates]). For each of the previously known loci except *ABCA1* (9q31), at least one of the candidate associations was nominally significant (*P*<0.05, two-sided) in at least one of the replication cohorts or in analysis combining p-values from the two replication cohorts when effect estimates (beta coefficients) indicated trends in lipoprotein measure consistent with the effects observed in the WGHS. Among the 7 novel loci from the primary analysis only, where the effect estimates for the WGHS were generally smaller and power for replication was less, concordance of the direction of effects remained high for the PROCARDIS sample [86% (25/29)], although only modest for the FHS sample [58% (22/38)], but these associations were not significant (two-sided *P*>0.05; Table S3). However, a recent genome-wide meta-analysis of LDL-C, HDL-C, and triglycerides found significant, but not genome-wide significant, associations among these fractions with candidate SNPs from the WGHS at *PCCB/STAG1* (3q22.3), *BTNL2* (6p21.32), *KLF14* (7q32.2), and 8p23.1 [10], although the significant SNP associations at *PCCB/STAG1* (3q22.3) and *BTNL2* (6p21.32) were not fully concordant between the two studies (Table 3). Independent evidence for functional consequence of the candidate SNP (rs10778213) at 12q23.2 is its genome-wide significant association in a smaller sample from the WGHS with plasma C-reactive protein (CRP), a biomarker of inflammation that is slightly correlated if at all with the two HDL measures associated at this locus (total HDL particle concentration [HDL:T], Spearman r = 0.22; HDL cholesterol estimated by the NMR [HDL:N], Spearman r = −0.04) [21]. With the larger sample of WGHS genotype information in the current study, the association with plasma CRP is more significant (*P*<5×10^−15^). Finally, the associations at *CCDC92/DNAH10/ZNF664* [12q24.31.B] and *WIPI1* (17q24.2) were not confirmed either in the meta-analysis shown in Table 3 or in a second genome-wide meta-analysis of LDL-C, HDL-C, and triglycerides that also evaluated gender stratified association [11] (data not shown). Nevertheless, ongoing genotyping in the WGHS of an additional 4639 samples (3305 with fasting status) completed subsequent to the main analysis provided significant support for these last two loci on the basis of internal replication, as well as significant or borderline significant support for four others, confirming directions of effects for all novel candidate associations, and leading to smaller p-values in analysis combining the main WGHS sample with the additional samples for all but three entries in Table 3 and at least one lipoprotein measure for each locus (compare to Table S1).

**Table 3:** Replication of associations with LDL-C, HDL-C, and triglycerides at novel loci.

		WGHS									Meta-analysis^a^		
Locus	gene(s)	sample	lead SNP^b^	lead fraction(s)^b^	internal replicationp-value^c^	Extende sample p-value^c^	LDL-C^d^	HDL-C^d^	TG^d^	N	LDL-C^d^	HDL-C^d^	TG^d^
3q22.3	*PCCB STAG1*	all	rs3856637	HDL:S (+)	0.018	**1.1×10^−9^**	n.s.	1.1×10^−2^ (+)	n.s.	18516	0.14 (+)	0.20 (−)	3.0×10^−5^ (+)
6p21.32	*BTNL2*	all	rs2076530	VLDL:L (+)	0.34	1.6×10^−7^	n.s.	n.s.	5.4×10^−7^ (+)	19584	0.94 (−)	0.034 (−)	0.33 (−)
				TG:N (+)	0.065	**1.3×10^−9^**							
		fasting	rs3129882	VLDL:L (−)	0.057	**1.8×10^−8^**	n.s.	5.9×10^−4^ (+)	2.1×10^−7^ (−)	19648	0.47(−)	7.0×10^−3^ (+)	0.35 (−)
				TG:N (−)	0.14	1.8×10^−8^							
7q32.2	*KLF14*	all	rs4731702	LDL:S (−)	0.00019	5.3×10^−9^	4.1×10^−3^ (−)	4.9×10^−7^ (+)	2.2×10^−9^ (−)	19648	0.56 (−)	8.8×10^−7^ (+)	5.9×10^−3^ (−)
				LDL:T (−)	0.0028	**5.4×10^−11^**							
				HDL:Z (+)	0.0065	**1.0×10^−10^**							
				TG (−)	0.0026	**2.7×10^−11^**							
8p23.1	*Intergenic PPP1R3B*	fasting	rs983309	VLDL:Z (+)	0.14	**3.8×10^−9^**	3.7×10^−5^ (−)	5.8×10^−3^ (−)	1.5×10^−2^ (+)	19648	2.4×10^−4^ (−)	5.7×10^−6^ (−)	0.52 (+)
12q23.2	*Intergenic ASCL1 PAH*	all	rs1818702	HDL:T (−)	0.04	**2.7×10^−10^**	n.s.	n.s.	3.4×10^−2^ (−)	19536	0.88 (+)	0.99 (−)	0.069 (−)
		all	rs10778213^e^	HDL:N (−)	0.096	**7.6×10^−9^**	0.021(+)	n.s.	n.s.	19648	0.12 (−)	0.22 (+)	0.47 (+)
12q24.31.B	*CCDC9 DNAH10 ZNF664*	all	rs7307277	HDL:L (+)	0.0032	**9.0×10^−13^**	1.4×10^−3^ (−)	5.5×10^−8^ (+)	6.1×^−10^ (−)	19648	0.40(+)	0.074 (−)	0.35 (+)
				HDL:Z (+)	0.0013	**1.5×10^−12^**							
				LDL:Z (+)	0.0073	**5.0×10^−10^**							
				LDL:T (−)	0.00024	**1.1×10^−11^**							
				LDL:S (−)	0.00029	**1.2×10^−12^**							
				TG (−)	0.015	**6.7×10^−11^**							
		fasting		HDL:L (+)	0.010	**1.4×10^−9^**							
17q24.2	*PRKAR1A WIPI1*	all	rs2909207	HDL:M (+)	0.045	**1.7×10^−9^**	n.s.	n.s.	2.2×10^−3^ (+)	19648	0.87 (−)	0.73 (+)	0.17 (+)

a Meta-analysis of LDL-C, HDL-C, and TG among 12 European populations [10].

b Most significant genome-wide SNP and lipoprotein fraction(s) association at locus for tests among the 22 lipoprotein fractions. (+/−) indicates trend of fraction with increasing copies of the minor allele (same as sign of beta coefficient, see Tables 2 and S2).

c P-value (two-sided) for association in additional 4639 (all) or 3305 (fasting) samples from the WGHS (internal replication). All association trends were consistent with discovery sample. Combining these new samples with the original discovery samples leads to p–values for the extended WGHS sample. Bold font indicates p-value smaller than in main discovery sample.

d P-value and trend (+/−) for association of increasing copies of the minor allele with indicated lipoprotein fraction in either WGHS or the meta-analysis. n.s. in WGHS analysis indicates not significant (*P*>0.05).

e Genome-wide significant association with plasma C-reactive protein in the WGHS [21].

### Magnitudes of genetic effects

**Figure 2:**
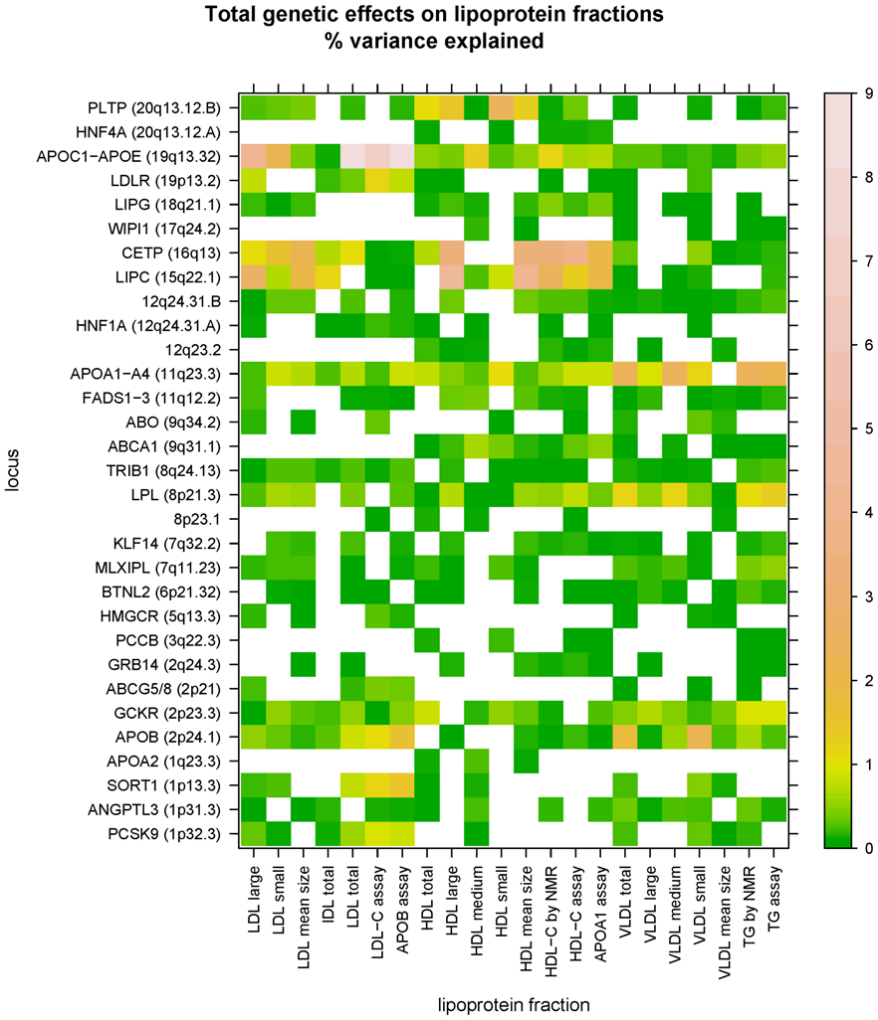
Variance explained in adjusted lipoprotein measures by common variation at the candidate loci by SNPs retained in model selection procedures. See also Figure S2 and Figure S3.

To assess the contribution of common genetic variation at each of the candidate loci to each of the adjusted lipoprotein fractions, we constructed regression models by stepwise selection of SNPs in the vicinity of the primary genome-wide significant associations. Most of these models explain less than 1% of the variation in the adjusted lipoprotein fractions (Figure 2, Table S5, and Table S6). The top three effects, all at *APOC-APOE* complex (19q13.32), explain 8.9%, 8.4%, and 7.1% of the variance in ApoB particle concentration, the related total LDL particle concentration, and LDL-C, respectively. Fasting status had an influence on retention of SNPs in the model selection procedure, but only for loci with modest effects (Compare Table S5 and Table S6). There were no genetic contributions remaining from the model selection procedure for any of LDL-C, HDL-C, triglycerides, ApoA1, or ApoB concentration at *APOA2* (1q23.3) in the whole sample and at *WIPI1* (17q24.2) in the fasting subsample, suggesting that these loci would not have been identified for genome-wide association with the five conventional lipoprotein fractions even in a much larger sample with the genome-wide SNP genotyping panel used in this study. Clustering loci on the basis of the profile of associated lipoprotein fractions suggests sub-groups of loci with related patterns of effects (Figure S2, Figure S3), perhaps suggesting distinct but possibly overlapping biological pathways for lipoprotein metabolism. For example, *HNF1A*, *LDLR*, *ABCG5/8*, *PCSK9*, and *CELSR2/PSRC1/SARS/SORT1* largely share associations with IDL, small VLDL, total VLDL large LDL, LDL-C, total LDL, and ApoB.

The total genetic effects for each lipoprotein determined by summing over the effects at all loci ranged from 2.1% for mean VLDL size to 17.2% for ApoB (Table 4). The effects were not substantially different when the entire model selection procedure was performed in the fasting subsample (Table 4), and only slightly smaller in general among the unadjusted lipoprotein fractions (Table S7). Notably, the common genetic variation in this study at the genome-wide loci had a greater total effect on mean particle size than on standard clinical cholesterol measures for HDL but not for LDL or VLDL (Table 4).

**Table 4:** Proportion (%) variance in fully adjusted lipoprotein fractions explained by common variation at candidate loci.

lipoprotein fraction	whole sample	fasting subsample
LDL large	12.0	11.4
LDL small	8.9	9.4
LDL mean size	8.5	8.7
IDL total	3.5	3.5
LDL total	15.2	15.0
LDL-C assay	13.7	13.8
ApoB assay	17.2	16.8
HDL total	5.6	5.6
HDL large	13.1	12.5
HDL medium	4.6	4.4
HDL small	6.4	5.7
HDL mean size	12.2	11.7
HDL-C by NMR	10.3	9.9
HDL-C assay	9.9	9.1
ApoA1 assay	8.3	7.8
VLDL total	8.9	8.6
VLDL large	3.8	4.1
VLDL medium	6.0	6.0
VLDL small	7.6	7.4
VLDL mean size	2.1	2.5
TG by NMR	7.9	7.6
TG assay	7.7	8.1

### Secondary genome-wide analysis

To examine the possibility that other loci might include SNPs with genome-wide significant association conditional on effects at the primary loci, we adjusted the primary lipoprotein fraction measurements (which were already adjusted for clinical covariates) for SNPs retained by the model selection procedure at the candidate loci, and repeated the genome-wide association testing. Quantile-quantile analysis confirmed that all of the excess of extremely small p-values in the original analysis could be explained by the variation at the candidate loci (not shown). Similarly, genotype-based statistical models (as opposed to the allele-based additive models used in the primary analysis) did not reveal other loci with genetic influences at the genome-wide significance level in the whole sample.

While we adjusted the lipoprotein measures with a full set of clinical characteristics to reduce variance and enhance power in the primary analysis, it remained possible that relevant SNPs would be overlooked if they acted through effects on the adjustment covariates. Similarly, subtle effects on the association estimates due to non-normality of the (possibly log-transformed) adjusted lipoprotein measures or due sub-European population stratification might confound hypothesis testing. To evaluate whether our discovery procedure was robust, we performed secondary analyses repeating the entire genome-wide discovery procedure for alternative nested subsets of clinical covariates with and without further adjustment for population structure and quantile normalization (Table S8). Comparing the full adjustment procedure to alternatives using either a reduced set of clinical covariates or age only, with or without additional adjustment for potential sub-European population stratification and quantile normalization yielded further genome-wide significant associations at three loci with known lipid metabolic genes, *LPA* (6q25.3), *LCAT* (16q22.1), and *APOH* (17q24.2), and two additional loci, 6p22.3 and 10q21.3. All of the additional loci were present in the age-adjusted analysis. Associations at 6p22.3 and 10q21.3 appear to be novel and implicate, respectively the *GMPR* or *MYLIP* genes and the *JMJD1C* gene. The lead SNPs at each of these loci were significantly associated with at least one of LDL-C, HDL-C or triglycerides in the recently published meta-analysis (Table 5) [10]. Similarly, in internal replication among the additional 4639 WGHS samples with genotype available after the main analysis was complete, associations at the candidate SNPs were all significant and the trends of effects were all consistent with effects in the discovery sample (Table 5). We note that at *JMJD1C* (10q21.3), the candidate SNPs have minor allele frequency near 0.5, and that available data does not allow us to determine whether the differences in the direction of the minor allele effect on VLDL fractions in the WGHS and triglycerides in the previously published replication study are truly physiological or rather that the frequency of the coded (i.e. minor) allele from the WGHS is greater than 0.5 in the replication cohort resulting in an opposite sign of the effect estimates.

**Table 5:** Genome-wide significant associations (p<5×10^−8^) at novel loci in analysis of age-adjusted and full-plus-triglycerides-adjusted* lipoprotein fractions.

				WGHS						replication best association+			
Adjustment	Locus	candidate gene(s)	SNP	MAF	lipoprotein fraction#	beta (se)	p-value	internal replication p-value∧	extended sample p-value∧	N	fr.	est.	p-val.
age	6q22.3	*GMPR*, *MYLIP*	rs2480	0.47	LDL-C	−2.02 (0.36)	2.8×10^−8^	0.00020	5.8×10^−11^	7344	LDL-C	−2.89	0.003
	10q21.3	*JMJD1C*	rs7923609	0.49	VLDL:T	−1.93 (0.33)	5.2×10^−9^	0.14	7.4×10^−9^	19840	TG	3.31	0.0009
			rs12768534	0.46	VLDL:M	0.92 (0.17)	2.8×10^−8^	0.39	8.3×10^−8^	19840	TG	2.80	0.0005
triglycerides	11p15.4	*SBF2*	rs7938647	0.31	HDL:T	0.36 (0.065)	3.7×10^−8^	0.0049	9.7×10^−10^	19794	n.s.	n.s	n.s.

*: Full adjustment procedure (age, BMI, smoking status, menopausal status, use of hormone replacement therapy; see Materials and Methods) with addition of log-transformed triglyceride levels.

**+:** Most significant association among LDL-C, HDL-C, and triglycerides (TG) in recent meta-analysis [10]. n.s. = not significant (*P*>0.05).

# Abbreviation as in Table 2.

**∧:** P-value (two-sided) for association in additional 4639 samples from the WGHS. All association trends were consistent with discovery sample. Combining these new samples with the original discovery samples leads to p–values for the extended WGHS sample.

Since lipoprotein particle size is closely related to triglyceride content, we also performed secondary analysis examining genome-wide significant associations after adjustment of the lipoprotein fractions by the full set of clinical covariates and (log-transformed) triglyceride levels (Table 5 and Table S8). This analysis identified only one new genome-wide significant association. At 11p15.4, rs7938647 in the intron of the *SBF2* gene was associated with full-plus-triglyceride adjusted total HDL particle concentration. Again, internal replication provided support for this association although there was no association (*P*>0.05) with LDL-C, HDL-C, or triglycerides in the recent meta-analysis for replication.

### Associations distinguishing NMR-based from conventional lipoprotein measures

Among its unique characteristics, the NMR-based methodology provides information about IDL and VLDL particle concentration, both aspects of lipoprotein profiles that are difficult to measure by conventional methods. For IDL, genetic associations were observed at many of the candidate loci (Figure 2, Table 2, Table S1) and most strongly at *LIPC* (15q22.1), where rs1532085 had an estimated 0.11 nmol/l shift in particle concentration for each copy of the minor allele (p = 1.5×10^−20^). For total VLDL concentration, association with genetic variation was observed at many loci but none more strongly than at the *APOC-APOE* complex where rs439401, which is in perfect LD with rs7412 (the SNP that distinguishes *APOE* alleles E2 and E3), had an estimated −2.4nmol/l shift in concentration per copy of the minor allele (p = 2.1×10^−12^; Table S1).

Loci strongly affecting the relative concentration of NMR-based estimates of small, medium, and large particle size could be identified on the basis of genome-wide effects on mean particle size, and these associations were of special interest when there was no accompanying association with the corresponding cholesterol measure retained in the model selection procedures (Table 6, Figure S4). For LDL, mean particle size was associated with genome-wide significance at 12 loci (Table 2), among which the model selection procedures failed to identify any association with LDL-C at *MLXIPL* (7q11.23), *LPL* (8p21.3), *CCDC92/DNAH10/ZNF664* (12q24.31.B), and *LIPG* (18q21.1). These loci implicate genes related to glucose or triglyceride metabolism as well as unrecognized biological function at one novel locus (*CCDC92/DNAH10/ZNF664* [12q24.31.B]). The associations with mean LDL particle size were a consequence of strong inverse effects on large and small LDL particles (*MLXIPL* [7q11.23], *LPL* [8p21.3], *LIPG* [18q21.1]) or of exclusive effects on small LDL (*CCDC92/DNAH10/ZNF664* [12q24.31.B]) [see Figure S4]. In the fasting subsample, the associations with the NMR based measures at *LPL* (8p21.3) and *LIPG* (18q21.1) also met genome-wide significance, but the associations at *MLXIPL* (7q11.23) and *CCDC92/DNAH10/ZNF664* (12q24.31.B) did not. For HDL, 9 loci had genome-wide significance for mean particle size (Table 2), among which the clinical measure of HDL-C was not associated with genetic variation only at *GCKR* (2p23.3), as was also found in the fasting subsample (Figure 2, Table 6). The discordant effects on LDL size and cholesterol content at *LPL* (8p21.3), *CCDC92/DNAH10/ZNF664* (12q24.31.B), and *LIPG* (18q21.1) but not those of HDL size and cholesterol content were independent of triglyceride level in as much as associations persisted in analysis that further adjusted the lipoprotein fractions for (log-transformed) triglycerides, although only at nominal significance rather than genome-wide significance (Table 6).

**Table 6:** Loci with genome-wide significant association (*P*<5.0×10^−8^) for mean particle size but no associations in model selection procedures with cholesterol content or particle number*.

locus		Fraction∧	
LDL mean size v.		LDL-C	LDL:T
7q11.23 (*MLXIPL*)		W	
8p21.3 (*LPL*)		**w/f**	
12q24.31.B		**w**	
15q22.1 (*LIPC*)			w/**f**
18q21.1 (*LIPG*)		w/**f**	**w**/f
HDL mean size v.		HDL-C	HDL:T
2p23.3 (*GCKR*)		w/f	
7q32.2			**w**
8p21.3 (*LPL*)			f
11q12.2 (*FADS1-3*)			**w/f**
12q24.31.B			**w**
15q22.1 (*LIPC*)			**w**/**f**
VLDL mean size v.		VLDL:T	
8p23.1		F	

*: See Figure S4 for effects of individual SNPs across lipoprotein measures.

**∧:** Bold typeface indicates differential associations for triglyceride adjusted fractions with at least nominal significance. w = whole sampe, f = fasting subsample.

By the same standards, loci could be identified with effects on mean particle size but not total particle concentration (Table 6). Thus, SNPs at *LIPC* (15q22.1) and *LIPG* (18q21.1) had genome-wide significant associations for mean LDL particle size, but were null for particle concentration in model selection procedures in both the whole sample and the fasting subsample. These loci are characterized by genes known to influence triglyceride metabolism. Similarly, for HDL, comparison of associations with mean particle size and total particle concentration identified variation at *KLF14* (7q32.2), *FADS1-3* (11q12.2), *CCDC92/DNAH10/ZNF664* (12q24.31.B) and *LIPC* (15q22.1), implicating roles for known lipid candidate genes as well as loci with unknown functions. Variation at the novel locus *WIPI1* (17q24.2), while not affecting mean HDL particle size, was associated with the concentration of medium-sized HDL, but not large or small HDL, total HDL particle concentration, or HDL-C (Table 2, Figure 2, Figure S4). In addition, associations at *LPL* (8p21.3) in the fasting subsample distinguished total HDL particle concentration from HDL-C (Table 6). VLDL particle size but not concentration was influenced by variation at 8p23.1 in the fasting subsample but there were no genome-wide significant associations at this locus in the whole sample. Again, in triglyceride-adjusted analysis, discordant effects on mean particle size and total concentration persisted but at some of the candidate loci in the analysis of LDL and HDL (Table 6).

### Lipoprotein candidate loci from other genome-wide association studies

Recent genome-wide meta-analysis of lipoprotein LDL-C, HDL-C, and triglycerides identified and validated 17 loci that were not found at the genome-wide significance level in the current population [10]–[12] in spite of comparable statistical power. We examined SNPs within 100kb of each of these additional candidate loci to extend associations to each of the NMR-based lipoprotein fractions. The choice of a threshold p-value for significance is a controversial issue in these analyses: although all of the candidate loci had been validated previously, the current analysis was performed in the context of a genome-wide association study. We present all locus associations when statistical significance of Bonferroni corrected p-value for the most significant locus association was less than 0.05, accounting for the product of the number of lipoprotein fractions tested (22) and the number of locus SNPs considered (range 8–125) (Table S9 and Table S10). Seven loci (*TMEM57*, *GALNT2*, *TIMD4/HAVCR1*, *MADD/FOLH1/NR1H3*, *MVK/MMAB*, *LCAT*, *CLIP2/PBX4/NCAN/SF4*) met this criterion in the whole WGHS sample and, at the same standard, one more locus (*MAFB*) could be added in the fasting subsample. Among these loci, associations with lipoprotein size measures were found for LDL at *GALNT2*, and for HDL at *GALNT2*, *MADD/FOLH1/NR1H3*, *MVK/MMAB*, *CLIP2/PBX4/NCAN/SF4*. No associations at the stringent significance level were found with mean VLDL size or total IDL concentration. Associations with HDL and LDL total particle concentration were largely consistent with parallel associations with ApoA1 and ApoB respectively.

## Discussion

By performing genome-wide association analysis among 17,296 Women with European ancestry for 22 NMR-based and conventional lipoprotein fractions, we identified 36 loci in the primary and secondary analyses for roles in lipoprotein metabolism, broadly defined. Ten of these loci have not been reported in other recent genome-wide association studies, including one identified only after adjustment for triglyceride levels. The functional bases for the associations are uncertain for five, including associations at 8p32.1 and 12q23.2 that map to intergenic regions. In spite of the high degree of correlation among some of the NMR-based and conventional measures, two of the novel loci (*PCCB* [3q22.3] and *PPP1R3B* [8p23.1]) could not have been found at the genome-wide significance standard solely with conventional measures (or their NMR-based equivalents) of lipoprotein profile in the WGHS. Replication in independent cohorts of men and women as well as other observations provided confirmatory evidence for candidate variation at all novel, although only through internal replication at *SBF2* (11p15.4), *CCDC92/DNAH10/ZNF664* (12q24.31.B), and *WIPI1* (17q24.2). The failure of external replication to validate these two novel loci may simply reflect intrinsic differences from the WGHS in NMR-based assay protocols (FHS) or clinical features of the cohort (e.g. lipid lowering treatment in PROCARDIS) as well as limiting power; alternatively, the associations observed in the WGHS may not reflect true genetic effects. Among the primary loci, total genetic effects were largest and appreciable for ApoB, total LDL, and others. They were the smallest for mean VLDL size. While the heritability for the NMR-based fractions has not been thoroughly explored, the present analysis suggests some aspects of lipoprotein profiles may be much less affected by common genetic variation than others. Combining the 31 loci in the primary analysis, the five loci in the secondary analysis (three novel loci plus *APOH* and *LCAT*), and the seven previously recognized loci for which the WGHS extends associations to the NMR-based lipoprotein measures brings the total to 43 loci characterized by the present study.

As important as the total number of candidate loci, some loci harbored variation exclusively correlated with the size of lipoprotein particles rather their cholesterol or total concentration (Table 6). *A priori*, one might have argued that triglyceride metabolic processes would be critical in this respect. This notion was confirmed by several candidate genes with known function in triglyceride metabolism, for example the enzymes encoded by *LPL*, *LIPC*, *LIPG*, and *GCKR* as well as the transcriptional regulatory protein encoded *MLXIPL* all have activities that may alter equilibrium pools of triglycerides and hence particle size or concentration. Other loci with only partly understood function were also identified, and these loci may now be further characterized through the current analysis. While it remains possible that the loci in Table 6 contain genetic variants not evaluated in this study and yet associated with cholesterol content or total particle concentration, the discordant effects on particle size compared with cholesterol or total particle concentration suggest biochemical pathways impinging on aspects of lipoprotein metabolism that are overlooked by standard clinical testing. To the extent that the pathophysiology of cardiovascular disease and related metabolic disorders, e.g. diabetes, is influenced by the distribution of lipoprotein particle size there may be therapeutic opportunities targeting the biochemical pathways identified by the discordant associations.

The procedures in the primary analysis enforced a genome-wide significance standard of *P*<5×10^−8^ for each lipoprotein measure. This standard was likely adequate for performing separate tests in the whole sample and the fasting subsample (see Materials and Methods) but does not explicitly address the multiplicity of testing the 22 lipoprotein measures at once. In part, the burden of significance is attenuated by correlations between the lipoprotein measures (Table S2), but the correlations are not exact and independent aspects of each measure are revealed by the diversity of effects shown in Figure 2 as well as by the discordant associations of Table 6. However, the choice of *P*<5×10^−8^ for genome-wide significance can be further justified by false discovery rate (FDR) analysis. For p-values from all of the lipoprotein measures considered at once, the conventional standard requiring FDR<0.05 implied a *P*<2×10^−5^, more than two order of magnitude less significant than the genome-wide p-value threshold. Similarly, among the individual lipoprotein measures, FDR<0.05 implied at worst *P*<7×10^−7^ for the case of IDL, still less significant than our genome-wide standard by over an order of magnitude. Thus, on a post-hoc basis, applying the conventional genome-wide standard *P*<5×10^−8^ for all fractions appears to have been justified.

Four of the 10 novel loci (7 from primary analysis, 3 from secondary analysis) have functional links to lipoprotein metabolism or disease status, even if strict biochemical roles of the candidate genes and protein are not yet known. Variation at *BTNL2* (6p21.32) has been associated with Grave's disease, multiple sclerosis, and sarcoidosis, apparently independent of the neighboring HLA class DR genes [22]–[24]. In addition, the lipoprotein association at this locus is within 780kb of a recently reported association of rs2254387 with LDL-C attributed to the *B3GALT4* gene encoding a galactosyltransferase [6]. At *STAG1/PCCB* (3q22.3), the genome-wide significant association with small HDL particle concentration is in the *STAG1* gene, but a more likely candidate for lipid metabolism may be the adjacent *PCCB* gene encoding the propionyl coenzyme A carboxylase beta subunit, in which substitutions cause Mendelian forms of proprionic acidemia (see, for example [25]). At 8p23.1, over 150kb from the candidate SNP rs983309, *PPP1R3B* encodes a phosphatase regulating glycogen phosphorylase, a plausible regulator of glucose and triglycerides. At 17q24.3, the connection to lipid metabolism can be made through an encoded domain of *WIPI1* protein, the WD40 domain, which is a structural motif thought to interact with phospholipids [26]. The strongest association at this locus is over 2Mb away but statistically independent from the associations of rs1801689 with full-plus-triglyceride-adjusted total LDL particle concentration or rs2909207 with age-adjusted medium HDL particles (Table 5), both adjacent to the lipid candidate gene *APOH* [27]. The remaining six loci have intergenic status, or are proximal to genes with unresolved connections to lipoprotein metabolism.

Nevertheless, association at one of these six loci, 12q23.2, between rs7307277 and HDL-C measured by NMR involves the same SNP we previously reported for genome-wide significant association with plasma C-Reactive Protein (CRP) in a subset of the current population [21], an association that remains highly significant in the current sample (*P* = 4.5×10^−15^). Previous reports, including our own, had also identified associations at *GCKR*, *APOC-APOE* complex, and *HNF1A* with both lipid fractions and CRP [21]. We could now also add *HNF4A* to this list since rs4810479 at 20q13.12.A is associated in the WGHS with both CRP and the lipoprotein fractions (Table 2, Table S1). These links between lipoprotein metabolism and CRP are particularly intriguing given the efficacy of lipid lowering therapy with statins among individuals identified as at risk on the basis of elevated CRP [28].

The etiology of cardiovascular disease is complex, and is believed to include an interplay between cell-based processes, including inflammation, and blood components, including lipoprotein fractions. The latter aspect may be summarized by clinical measures of cholesterol or triglycerides, or by ApoA1 and ApoB concentration. However, none of these aggregate measures reflects the full diversity of lipoprotein species in blood. The current investigation not only identifies novel loci for lipid metabolism in general, but may also help delineate the impact of lipoprotein metabolic genes on lipoprotein profile viewed with the highest resolution currently available.

## Materials and Methods

### Ethics statement

All analyses were performed with approval of local institutional review boards (IRBs).

### Study populations

All samples in the discovery analysis derive from the Women's Genome Health Study (WGHS), a prospective cohort of North American women with phenotypes related to cardiovascular disease, extensive clinical and demographic data, blood samples at baseline, and ongoing genome-wide genotyping [29]. The current data derive from 17,296 WGHS participants with confirmed, self-reported European ancestry who were non-diabetic, not using lipid lowering therapy at baseline, and for whom genotype information was available. Within this group, 12,489 (72%) provided the baseline blood sample at least 8 hours after a meal and these participants constitute the fasting subsample. Samples in the replication analysis derive from PROCARDIS, an ongoing European study of premature coronary artery disease [30],[31], and from the Framingham Heart Study (FHS) [32], an ongoing, family-based longitudinal cohort designed to identify correlates with cardiovascular health, including subgroup analysis of the impact of plasma lipoprotein fractions. The FHS samples with NMR-based lipoprotein measurements for replication derive from the Offspring cohort within the FHS [19].

### Lipoprotein determinations

In the WGHS, lipoprotein determinations were performed on baseline plasma samples that had been stored in liquid nitrogen (−170°C) since collection. LDL-C, HDL-C, triglycerides, ApoA1, and ApoB_100_ levels were all measured by direct assay and had low coefficients of variation [29]. NMR-based lipoprotein fractions were determined as described by proton NMR spectroscopy (LipoProtein-II assay, Liposcience Inc., Raleigh, NC) [33]. The coefficients of variation for these measures were also low (range 0.4–7.1%), except for the concentration of medium HDL particles (CV<30%) and IDL particle concentration (CV = 13.1%) [4]. PROCARDIS measurements were also performed with LipoProtein-II assays. Lipoprotein fractions for the FHS [19] samples were measured with the LipoProtein-I assay (Liposcience Inc. Raleigh, NC), which provides less accuracy for some measurements but is otherwise similar to LipoProtein-II.

### Genotyping

Genotyping in the WGHS sample was performed using the HumanHap300 Duo “+” chips or the combination of the HumanHuman300 Duo and iSelect chips (Illumina, San Diego, CA) with the Infinium II protocol. In either case, the custom SNP content was the same; these custom SNPs were chosen without regard to minor allele frequency (MAF) to saturate candidate genes for cardiovascular disease as well as to increase coverage of SNPs with known or suspected biological function, e.g. disease association, non-synonymous changes, substitutions at splice sites, etc. For quality control, all samples were required to have successful genotyping using the BeadStudio v. 3.3 software (Illumina, San Diego, CA) for at least 98% of the SNPs. In the final dataset, SNPs were retained with MAF >1%, successful genotyping in 90% of the subjects, and deviations from Hardy-Weinberg equilibrium not exceeding *P* = 10^−6^ in significance. A total of 335,603 unique SNPs, of which 32,521 derive from the custom content, remained in the final data. Although assays for two non-synonymous SNPs at the *APOE* locus (19q13.32), rs429358 and rs7412, which determine ApoE isotype, failed in the design of the Illumina custom content, genotypes for these two SNPs were determined separately by an allele-specific, PCR based method (Celera, Alameda, CA) [34]. These additional SNPs are in linkage disequilibrium with SNPs in the Illumina panel. The targeted genotypes for *APOE* were included during the model selection procedures but not during the primary analysis to discover loci with genome-wide significant associations.

### Analytic methods

Primary analysis to discover loci with highly significant associations in the WGHS discovery cohort was performed by linear regression in PLINK [35] assuming an additive relationship between the number of copies of the minor allele of each SNP and the mean values of the adjusted lipoprotein measures. A conservative threshold of *P*<5×10^−8^ was assumed for genome-wide significance [36]. For each lipoprotein measure, a full adjustment was performed by linear regression using the clinical covariates: age at baseline (continuous), BMI (continuous), menopausal status (yes/no), current smoking status (yes/no), and use of hormone replacement therapy (yes/no). Concentrations of IDL particles, total LDL particles, medium HDL particles, triglycerides determined by NMR, and triglycerides determined by chemical assay were log-transformed before adjustment to approximate normality. Self-reported European ancestry was confirmed among the WGHS participants included in the primary analysis by clustering in a principal component analysis in PLINK with 1443 ancestry informative SNPs chosen for large Fst values (>0.4) among the HapMap CEU, YRI, and JPN+CHB populations [37]. Discrepancy between self reported European ancestry and the clustering pattern was observed only for 68 samples (<0.5%), and these samples were excluded from the analysis. In addition, genomic control parameters for the primary analysis were close to unity, ranging from 1.013–1.061. There was an estimated 80% power at the genome-wide significance level to detect effects explaining 0.23% and 0.32% of the variance in the adjusted lipoprotein measures respectively in the whole sample and the fasting subsample.

The primary analysis also included association testing in a nested subset of 72% of the study participants who reported fasting for at least eight hours before providing the baseline blood sample. Analysis in this subset was expected to differ from the analysis in the whole sample by opposing trends: a loss of power due to reduced sample size was contrasted with possibly smaller variance among lipoprotein fractions that are influenced by prandial status, e.g. triglycerides. Because the majority of the sample was fasting, the association statistics in the two samples were expected to be highly correlated, and the statistical penalty for this additional testing in the Bonferroni framework was expected to be less than a factor of two. Our genome-wide significance threshold (*P*<5×10^−8^) was already smaller than required by correction for the number of SNPs tested by a factor of three, and justified including testing the fasting subset in the primary analysis.

Once loci having at least one genome-wide significant association with at least one lipoprotein fraction had been identified, a non-redundant set of SNPs contributing to each lipoprotein fraction at each locus was constructed by forward-backward stepwise selection using the Bayesian Information Criterion (BIC) from among all genotyped locus SNPs within 100 kb of the locus genome-wide SNP associations. Separately, these model selection procedures were performed also at each of the candidate loci with the unadjusted, but possibly log-transformed, lipoprotein fractions to estimate the proportion of variance explained without adjustment.

To assess the degree to which the adjustment procedure or sub-European population stratification might influence the identification of genome-wide loci, we performed a secondary analysis to evaluate the sensitivity of the locus discovery procedure to the adjustments applied to lipoprotein fractions before association testing. First, we adjusted for all of the clinical covariates as well as ten eigenvectors corresponding to a principal component analysis of genotype frequency in EIGENSTRAT [38] among 64,208 SNPs chosen with inter-SNP LD r^2^<0.2 and followed by quantile normalization of the residuals. Second, we adjusted with all of the clinical covariates except BMI, either with or without inclusion of the eigenvectors and subsequent quantile normalization. Finally, we adjusted only for baseline age, again either with or without inclusion of the eigenvectors and subsequent quantile normalization. In an additional secondary analysis, the genome-wide association procedures were performed with lipoprotein fractions transformed and fully adjusted as for the primary analysis, including also log transformed triglyceride levels among the adjustment variables (see text).

Additional analytic procedures, including the hierarchical clustering of loci according to effects on lipoprotein fractions, as well as the graphical representations were programmed in R [39], and included the False Discovery Rate analysis with the R-package QVALUE [40]. All annotations derive from human genome reference sequence hg18 (NCBI build 36.1), the UCSC Refseq as of October 27, 2008, and the dbSNP database (build 129) as represented by the UCSC database.

### Analysis in replication cohorts

In the Framingham Heart Study (FHS) sample, residual lipoprotein fractions were created by adjusting for gender, age at exam lipoprotein fraction collection (continuous), age-squared (continuous), and the top ten principal components from EIGENSTRAT [38] before analysis. When appropriate, log transformations were applied to approximate normality before computing residuals. Association testing was performed in R [39] using a linear mixed effect regression model with a kinship matrix to account for the family structure in the sample. Genotype data were derived by imputation using MACH 1.0 (http://www.sph.umich.edu/csg/abecasis/mach/) from raw genotypes collected with the Affymetrix (Santa Clara, CA) 500K array, and the regression models assumed a linear relationship between the dosage of the minor allele (ranging from 0 to 2) and the lipoprotein measures [10]. Only SNPs with high quality imputation measures (squared correlation of imputed and true genotype >0.3) were used in the analysis. In the PROCARDIS study [20], where genotype data derive from the Illumina (San Diego, CA) Human 1M platform representing a superset of the SNPs in the WGHS data, lipoprotein fractions were adjusted for case/control specific effects of age at baseline (continuous), gender, country of recruitment (Germany, Italy, Sweden, United Kingdom), self-reported hypertension (yes/no), diabetes (yes/no), current smoking status by questionnaire (yes/no), and statin therapy (yes/no). Regression models assumed a linear relationship between the number of copies of the minor allele and adjusted mean lipoprotein measure.

## Supporting Information

Figure S1 Locus p-values for lipoprotein fractions with at least one SNP reaching genomewide significance at each of the candidate loci. All plots correspond to analysis in the whole sample except for locus 8p23.1, for which genomewide association was observed only in the fasting subsample as shown.
(0.41 MB PDF)

Figure S2 Primary loci clustered hierarchically according to Cartesian distance corresponding to whether ( = 1) or not ( = 0) there were associations with each of the lipoprotein fractions in the model selection procedures (see Materials and Methods).
(0.02 MB PDF)

Figure S3 Dendorgram showing bierarchical relationships between loci clustered as in Figure S2.
(0.01 MB PDF)

Figure S4 Normalized SNP effects (beta coefficients) from univariate regression models. All plots correspond to analysis in the whole sample except for locus 8p23.1, for which genome-wide association was detected only in the fasting subsample as shown. Locus SNPs are shown if they were retained in the model selection procedure for at least one lipoprotein fraction. Absence of shading indicates the univariate beta coefficient was not significant (p>0.05). A small black dot for some combinations of SNPs and lipoprotein fractions indicates genomewide significance for the univariate beta coefficient.
(0.10 MB PDF)

Table S1 Best genome-wide associations with the lipoprotein fractions at each candidate locus.
(1.10 MB DOC)

Table S2 Correlations between all pairs of lipoprotein fractions.
(0.12 MB DOC)

Table S3 Replication of WGHS candidate associations from whole sample in PROCARDIS and the Framingham Heart Study.
(0.56 MB DOC)

Table S4 Replication of WGHS candidate associations from fasting sub-sample in PROCARDIS and the Framingham Heart Study.
(0.45 MB DOC)

Table S5 Proportion of variance in fully adjusted lipoprotein fractions explained in the whole sample by genetic variation at the candidate loci.
(0.15 MB DOC)

Table S6 Proportion of variance in fully adjusted lipoprotein fractions explained in the fasting sub-sample by genetic variation at the candidate loci.
(0.15 MB DOC)

Table S7 Total proportion of variance explained by candidate loci for each of the unadjusted lipoprotein fractions.
(0.04 MB DOC)

Table S8 Sensitivity analysis for locus discovery procedure.
(0.10 MB DOC)

Table S9 Lipoprotein associations in the whole sample at loci in previous lipid fraction GWAS.
(0.34 MB DOC)

Table S10 Lipoprotein associations in the fasting sub-sample at loci in previous lipid fraction GWAS.
(0.16 MB DOC)
