# Supplementary material for: Forty-Three Loci Associated with Plasma Lipoprotein Size, Concentration, and Cholesterol Content in Genome-Wide Analysis
Source: PLoS Genet. 2009 Nov 20;5(11):e1000730. doi: 10.1371/journal.pgen.1000730 (PMC2777390; doi:10.1371/journal.pgen.1000730)
Supplement: Figure S1 — Locus p-values for lipoprotein fractions with at least one SNP reaching genomewide significance at each of the candidate loci. All plots correspond to analysis in the whole sample except for locus 8p23.1, for which genomewide association was observed only in the fasting subsample as shown. (0.41 MB PDF) [file pgen.1000730.s001.pdf]

Figure S1. Locus p-values for lipoprotein fractions with at least one SNP reaching genomewide significance at each of the candidate loci.  All plots correspond to analysis in the whole sample except for locus 8p23.1, for which genomewide association was observed only in the fasting subsample as shown.

# Lipid fraction associations at 1p32.3

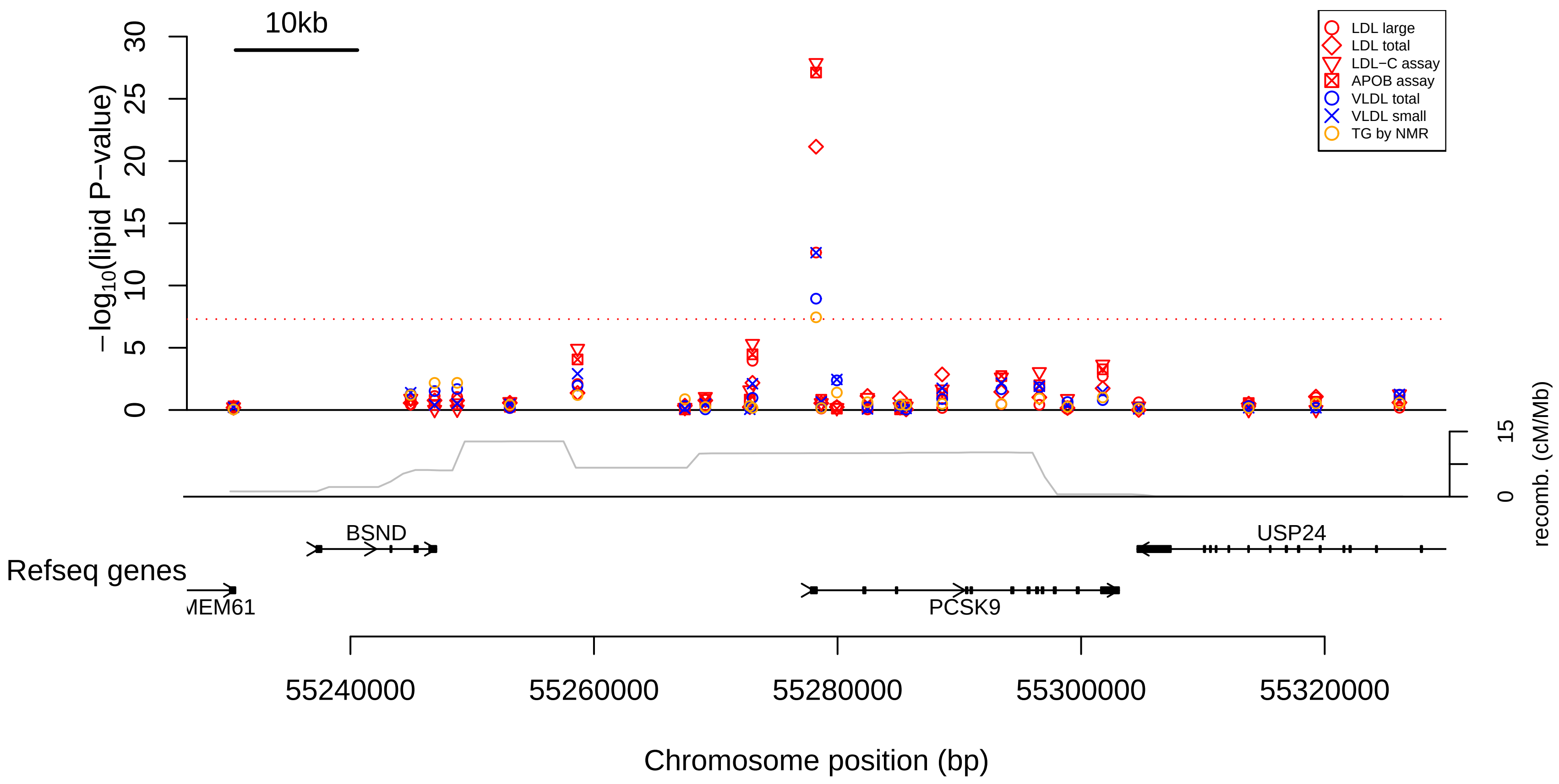

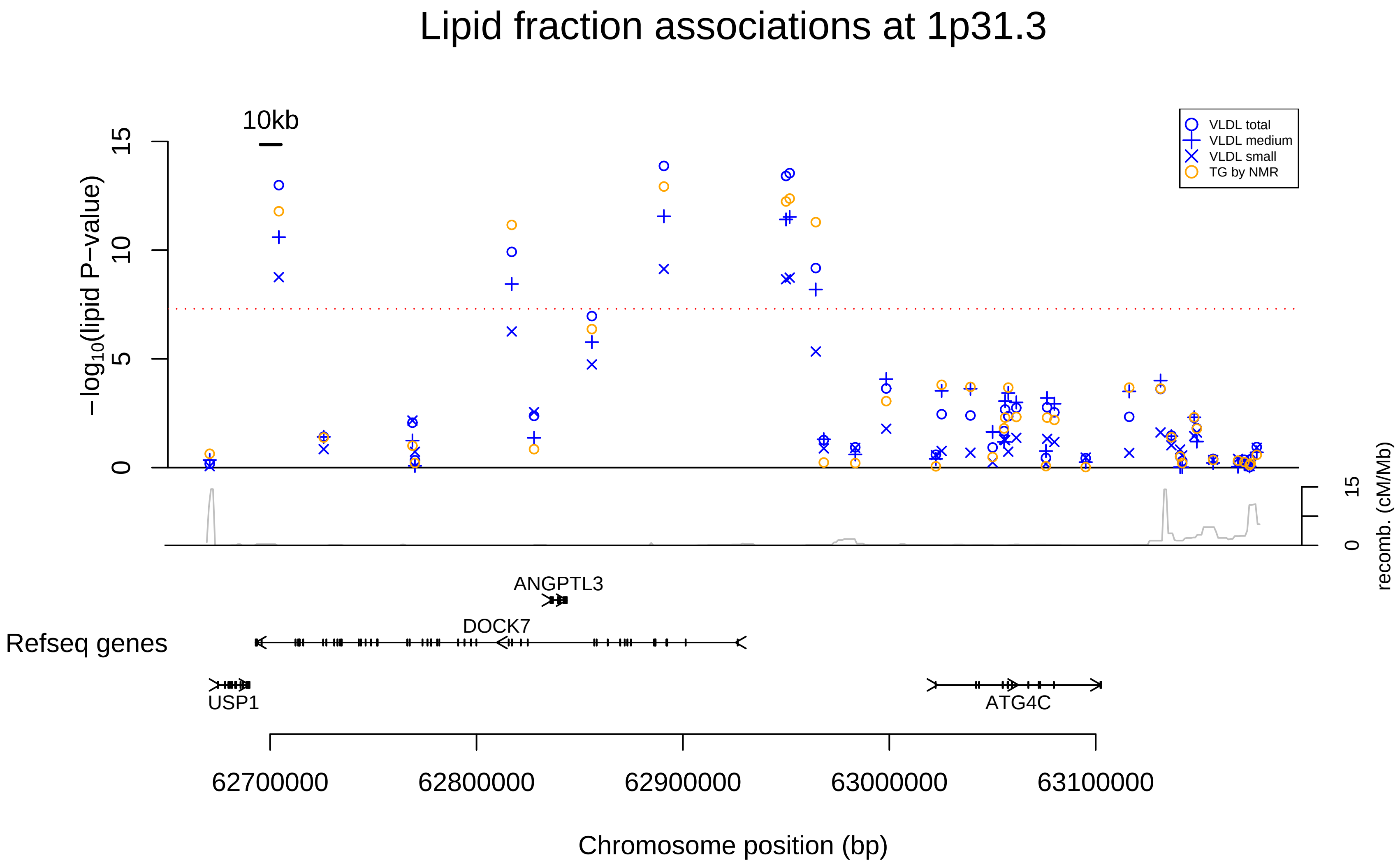

Lipid fraction associations at 1p31.3
10kb
−log10(lipid P−value)
0
5
10
15
VLDL total
VLDL medium
VLDL small
TG by NMR
recomb. (cM/Mb)
ANGPTL3
DOCK7
Refseq genes
USP1
ATG4C
62700000
62800000
62900000
63000000
63100000
Chromosome position (bp)

## Lipid fraction associations at 1p13.3

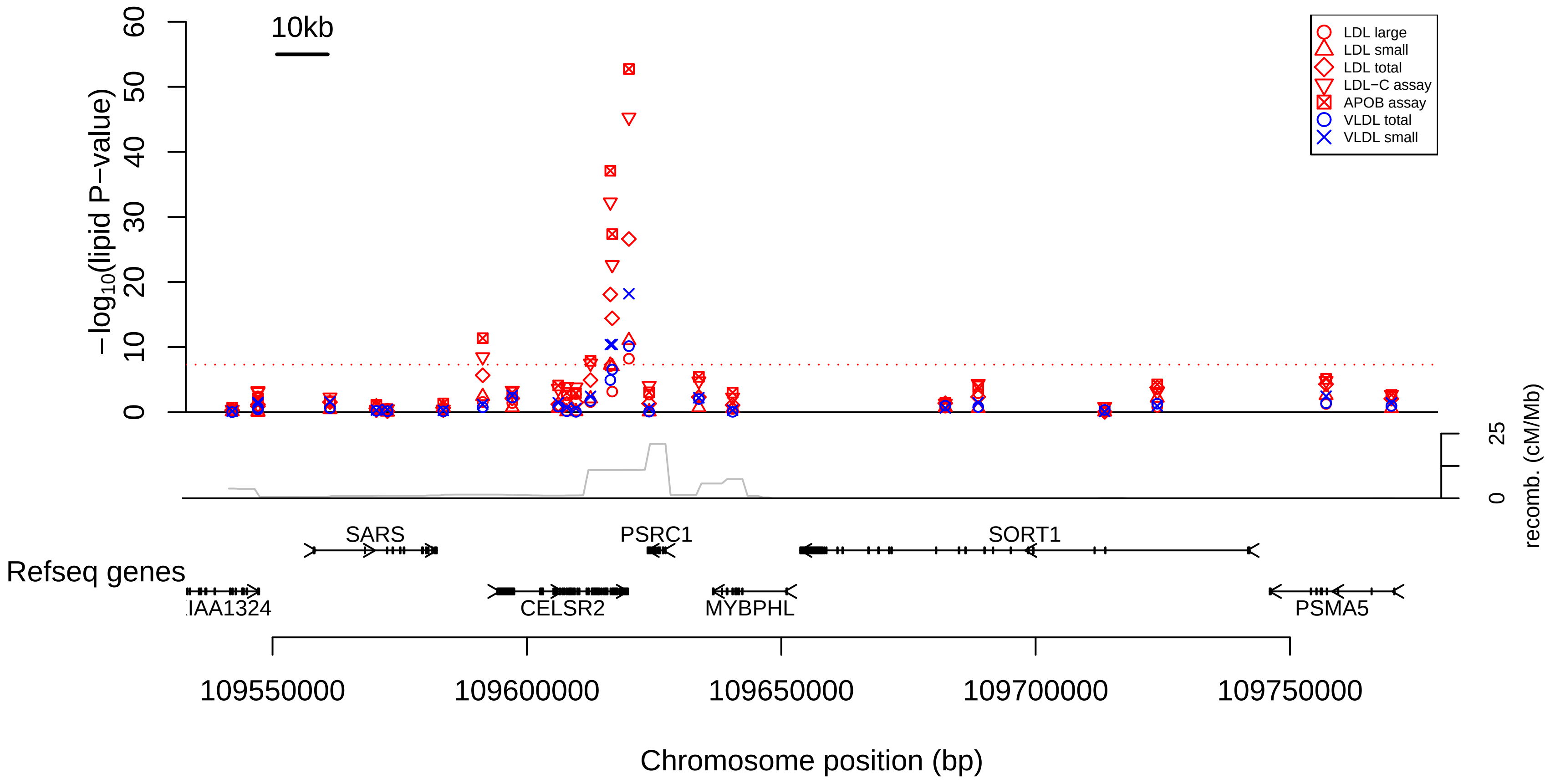

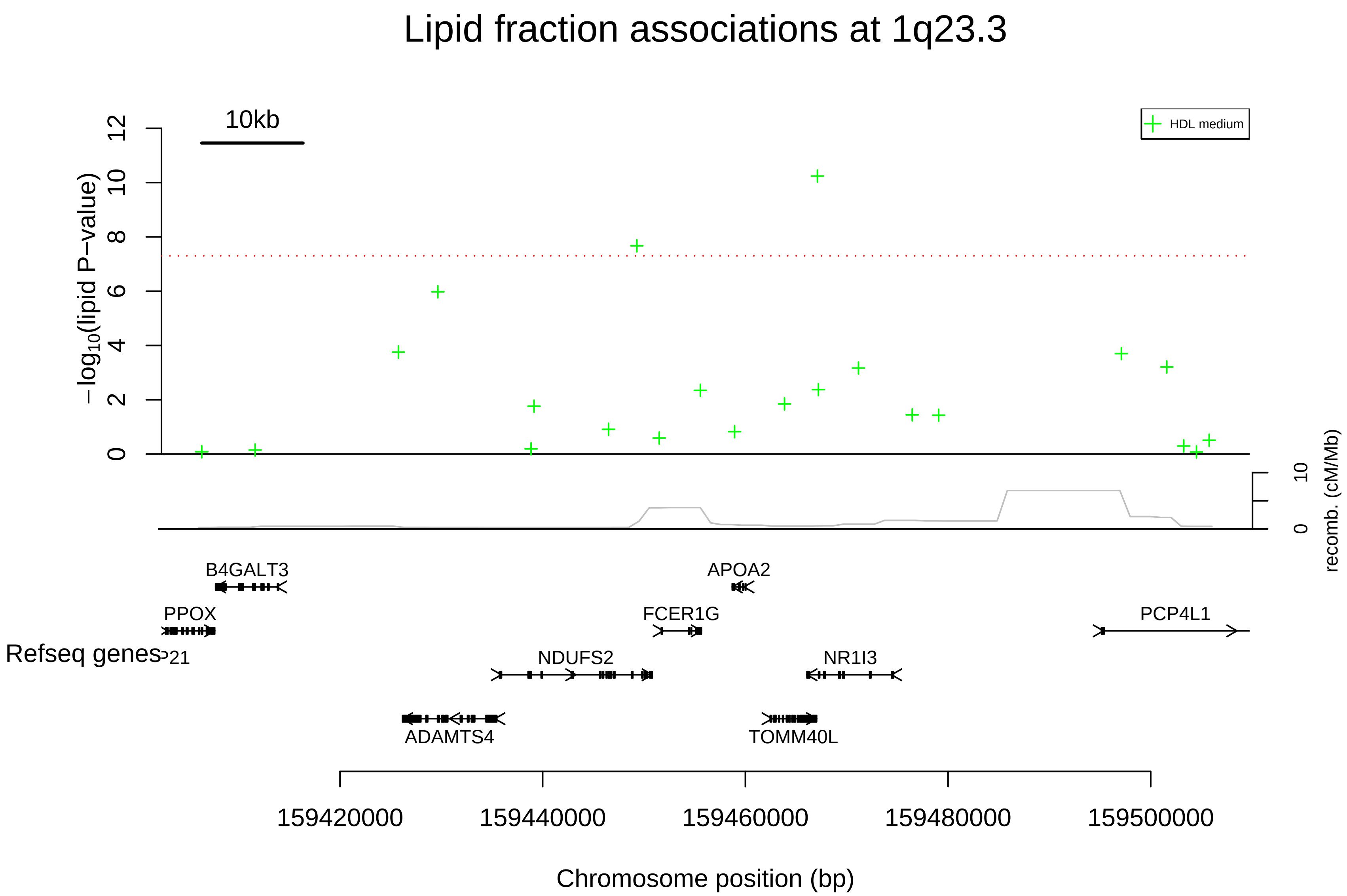

Lipid fraction associations at 1q23.3
10kb
HDL medium
−log10(lipid P−value)
0
2
4
6
8
10
12
recomb. (cM/Mb)
0
10
B4GALT3
APOA2
PPOX
FCER1G
PCP4L1
Refseq genes
NDUFS2
NR1I3
ADAMTS4
TOMM40L
159420000
159440000
159460000
159480000
159500000
Chromosome position (bp)

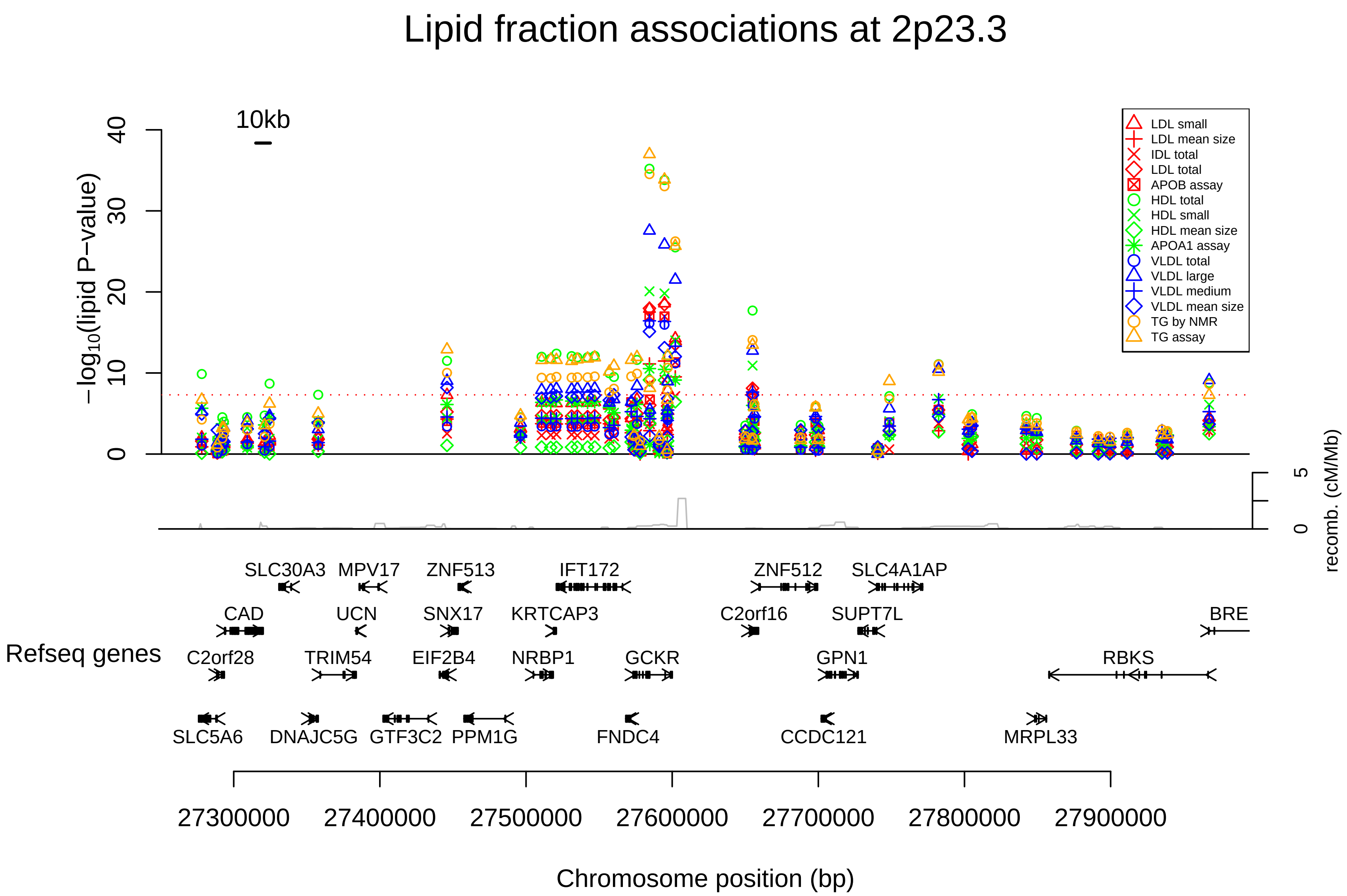

Lipid fraction associations at 2p23.3
−log10(lipid P−value)
0
10
20
30
40
10kb
LDL small
LDL mean size
IDL total
LDL total
APOB assay
HDL total
HDL small
HDL mean size
APOA1 assay
VLDL total
VLDL large
VLDL medium
VLDL mean size
TG by NMR
TG assay
recomb. (cM/Mb)
0
5
Refseq genes
SLC30A3
MPV17
ZNF513
IFT172
ZNF512
SLC4A1AP
CAD
UCN
SNX17
KRTCAP3
C2orf16
SUPT7L
BRE
C2orf28
TRIM54
EIF2B4
NRBP1
GCKR
GPN1
RBKS
SLC5A6
DNAJC5G
GTF3C2
PPM1G
FNDC4
CCDC121
MRPL33
27300000
27400000
27500000
27600000
27700000
27800000
27900000
Chromosome position (bp)

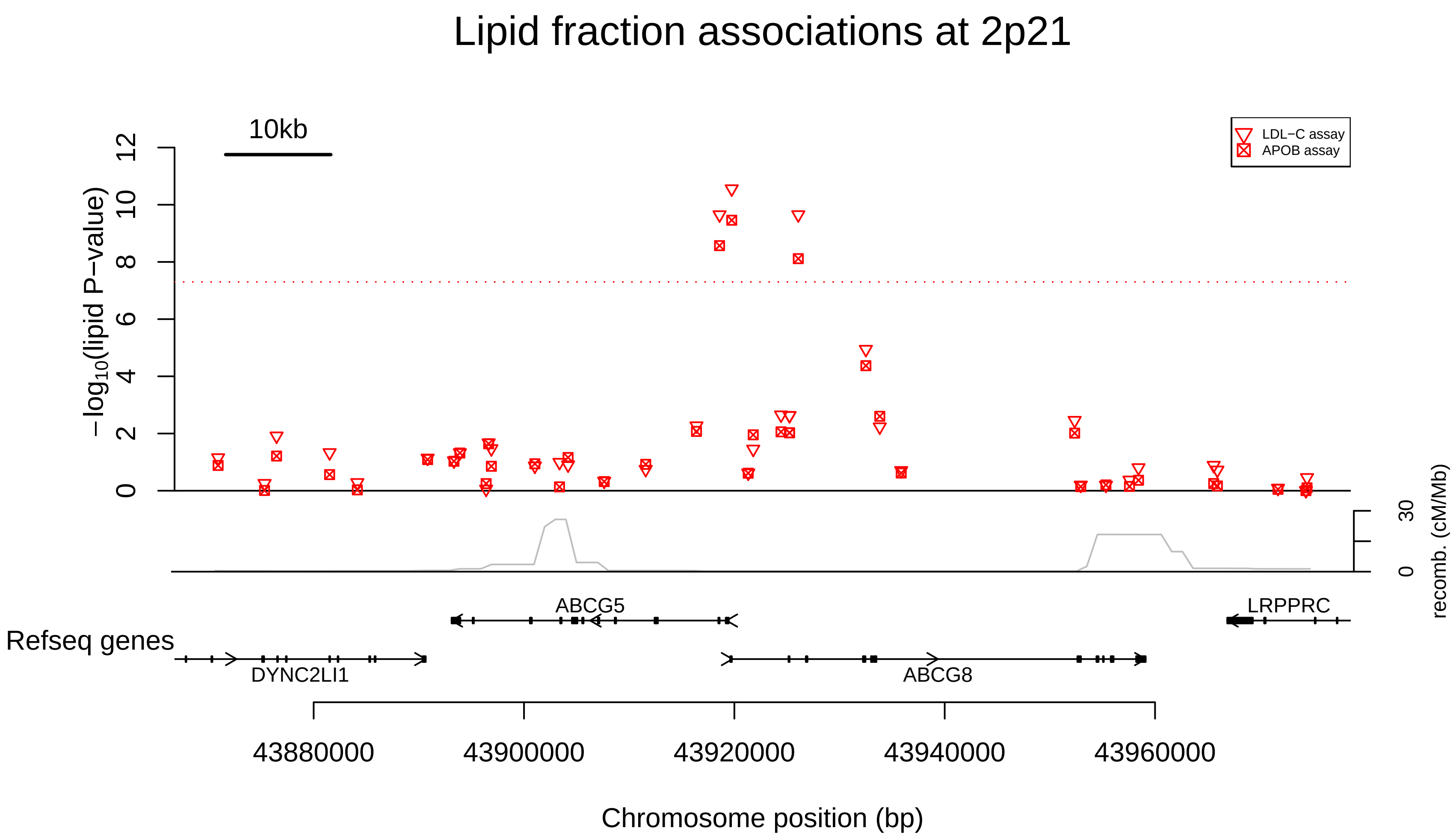

Lipid fraction associations at 2p21
10kb
−log10(lipid P−value)
0
2
4
6
8
10
12
LDL−C assay
APOB assay
30
0
recomb. (cM/Mb)
Refseq genes
ABCG5
LRPPRC
DYNC2LI1
ABCG8
43880000
43900000
43920000
43940000
43960000
Chromosome position (bp)

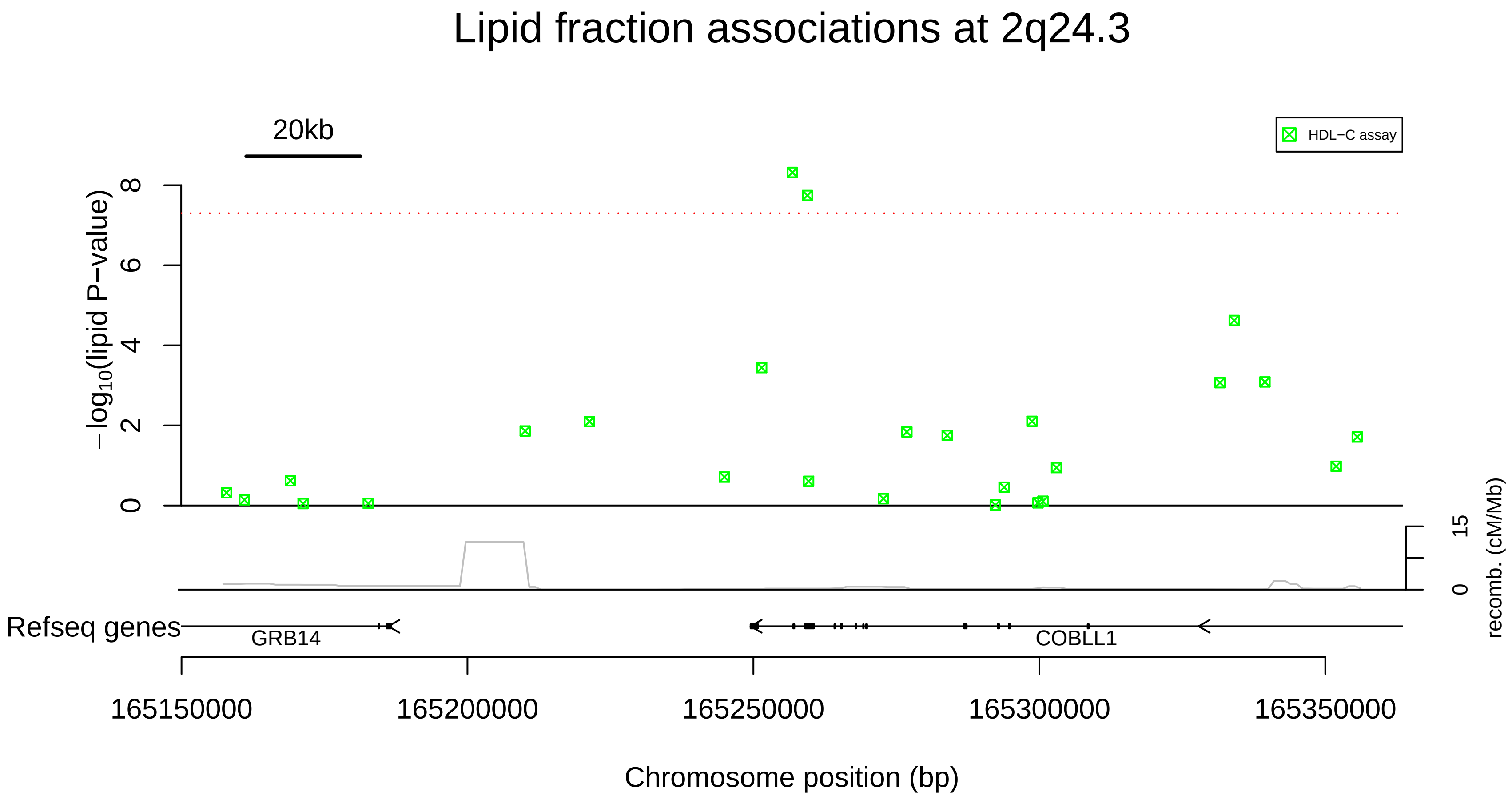
Lipid fraction associations at 2q24.3
20kb
HDL−C assay
−log10(lipid P−value)
0
2
4
6
8
15
0
recomb. (cM/Mb)
Refseq genes
GRB14
COBLL1
165150000
165200000
165250000
165300000
165350000
Chromosome position (bp)

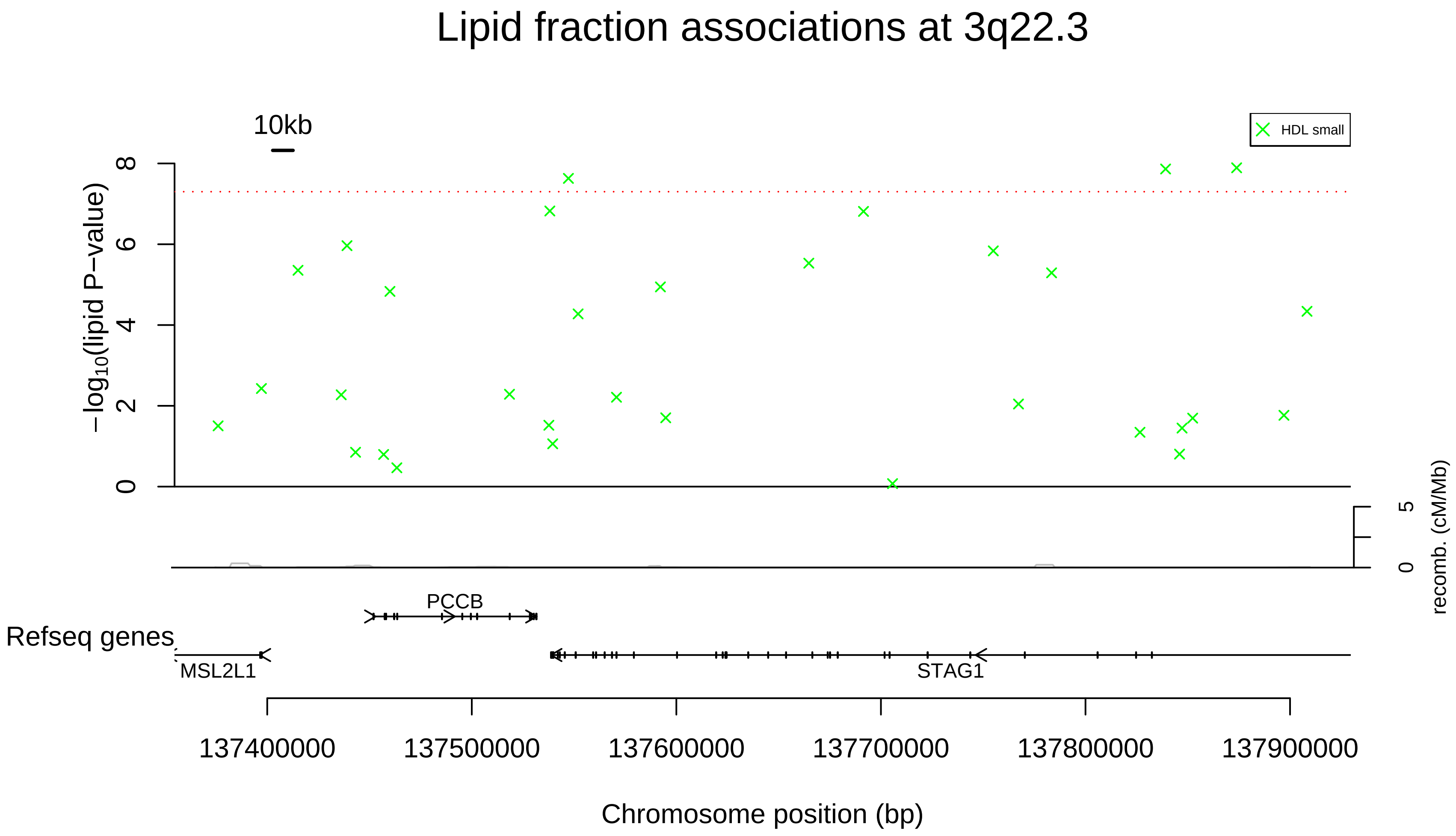

Lipid fraction associations at 3q22.3
10kb
HDL small
−log10(lipid P−value)
0
2
4
6
8
recomb. (cM/Mb)
0
5
Refseq genes
PCCB
MSL2L1
STAG1
137400000
137500000
137600000
137700000
137800000
137900000
Chromosome position (bp)

Lipid fraction associations at 5q13.3

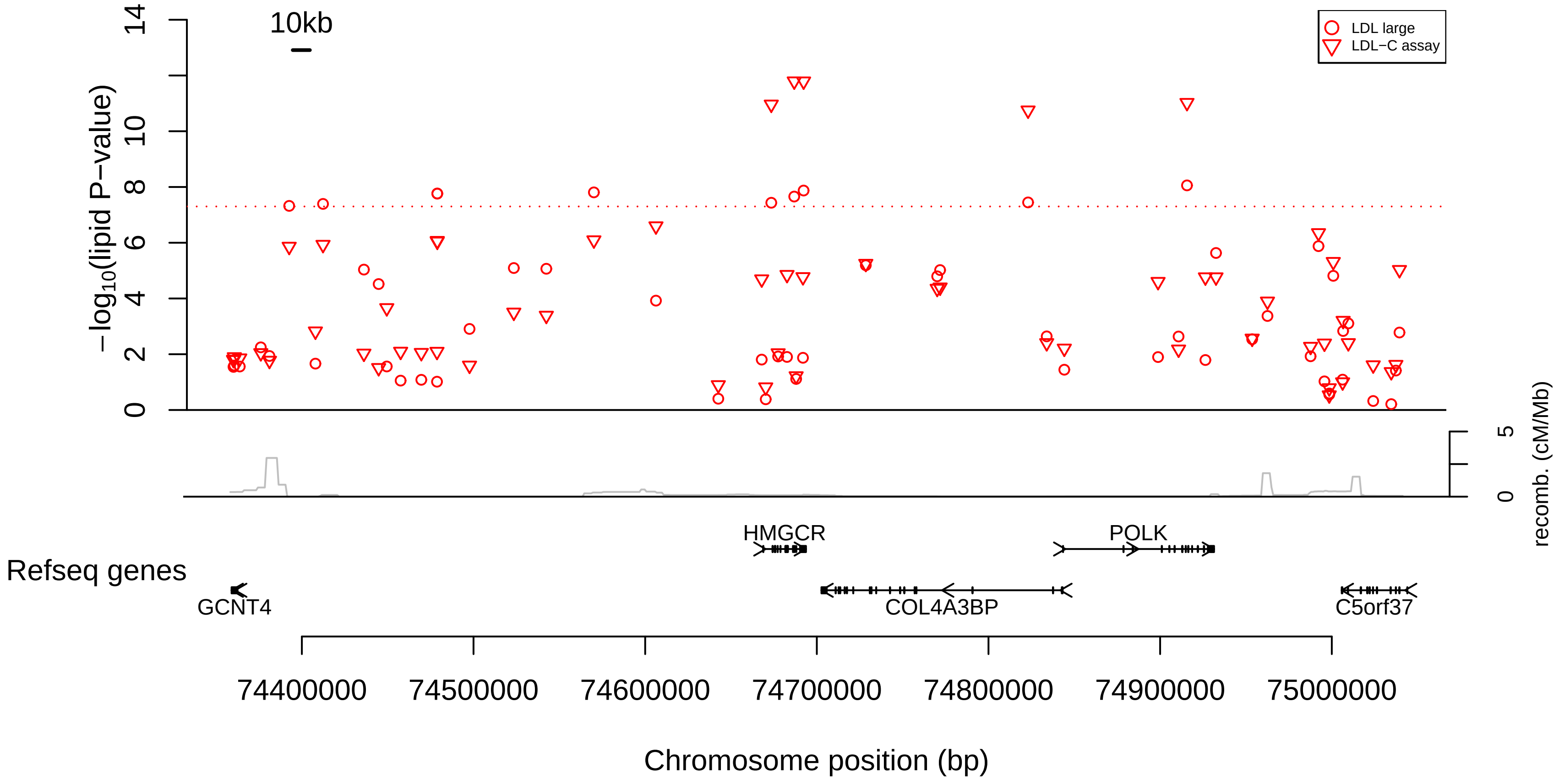

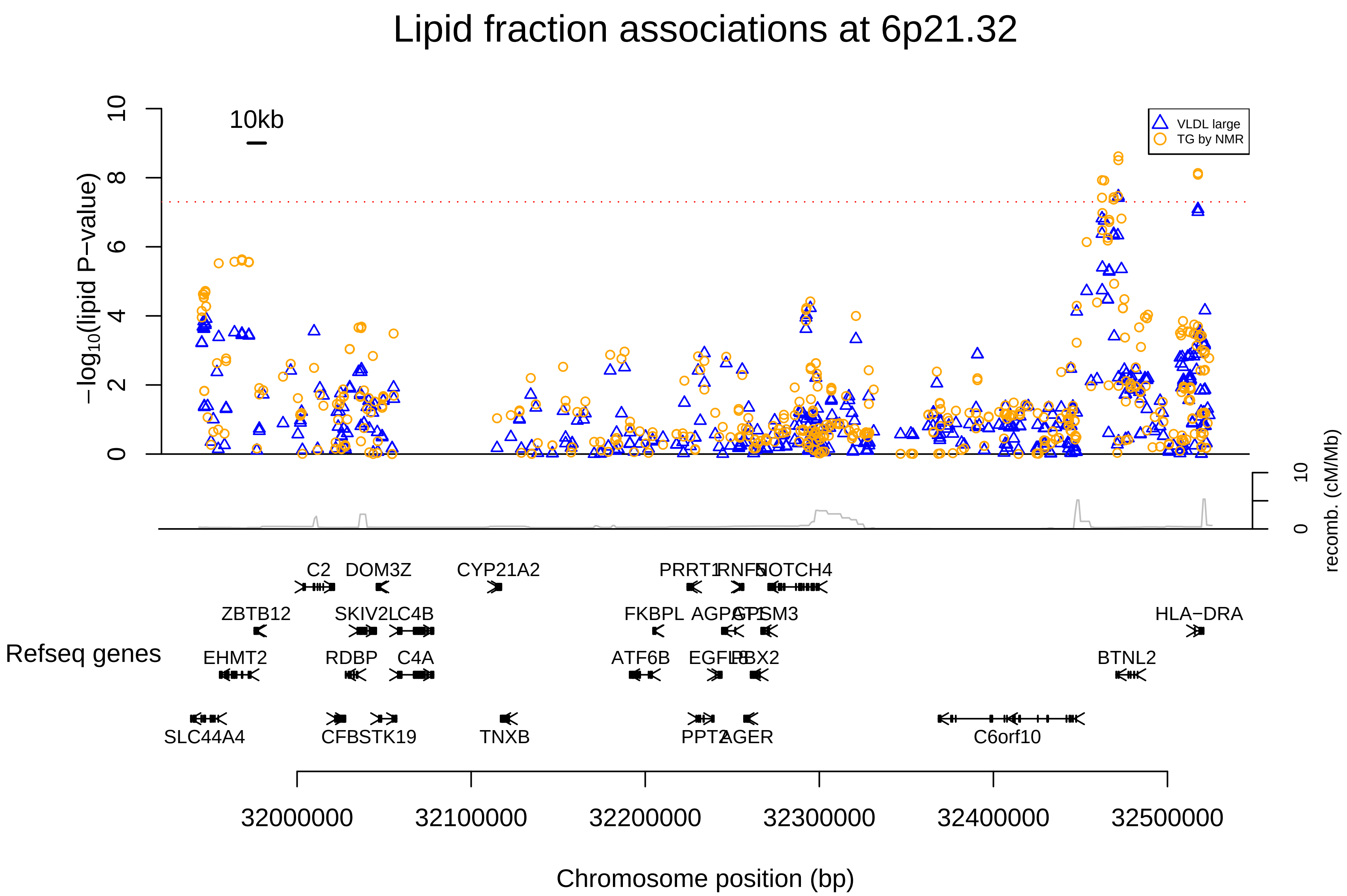

Lipid fraction associations at 6p21.32
10kb
VLDL large
TG by NMR
−log10(lipid P−value)
0
2
4
6
8
10
recomb. (cM/Mb)
Refseq genes
C2
DOM3Z
CYP21A2
PRRT1
RNF5
NOTCH4
ZBTB12
SKIV2L
C4B
FKBPL
AGPAT1
GPSM3
HLA−DRA
EHMT2
RDBP
C4A
ATF6B
EGFL8
PBX2
BTNL2
SLC44A4
CFB
STK19
TNXB
PPT2
AGER
C6orf10
32000000
32100000
32200000
32300000
32400000
32500000
Chromosome position (bp)

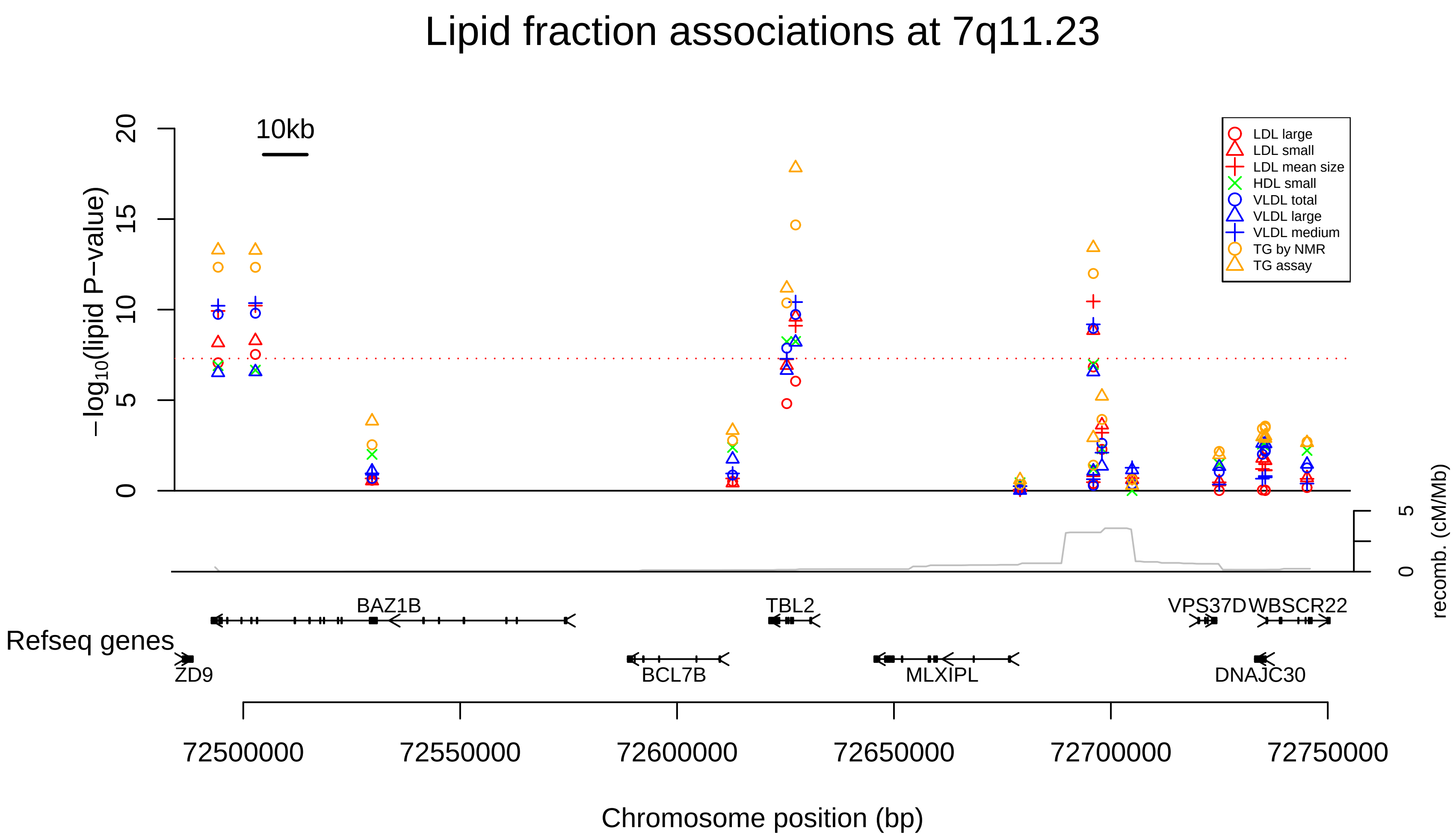

Lipid fraction associations at 7q11.23
10kb
$-\log_{10}$(lipid P−value)
0
5
10
15
20
LDL large
LDL small
LDL mean size
HDL small
VLDL total
VLDL large
VLDL medium
TG by NMR
TG assay
recomb. (cM/Mb)
0
5
Refseq genes
BAZ1B
TBL2
VPS37D
WBSCR22
ZD9
BCL7B
MLXIPL
DNAJC30
72500000
72550000
72600000
72650000
72700000
72750000
Chromosome position (bp)

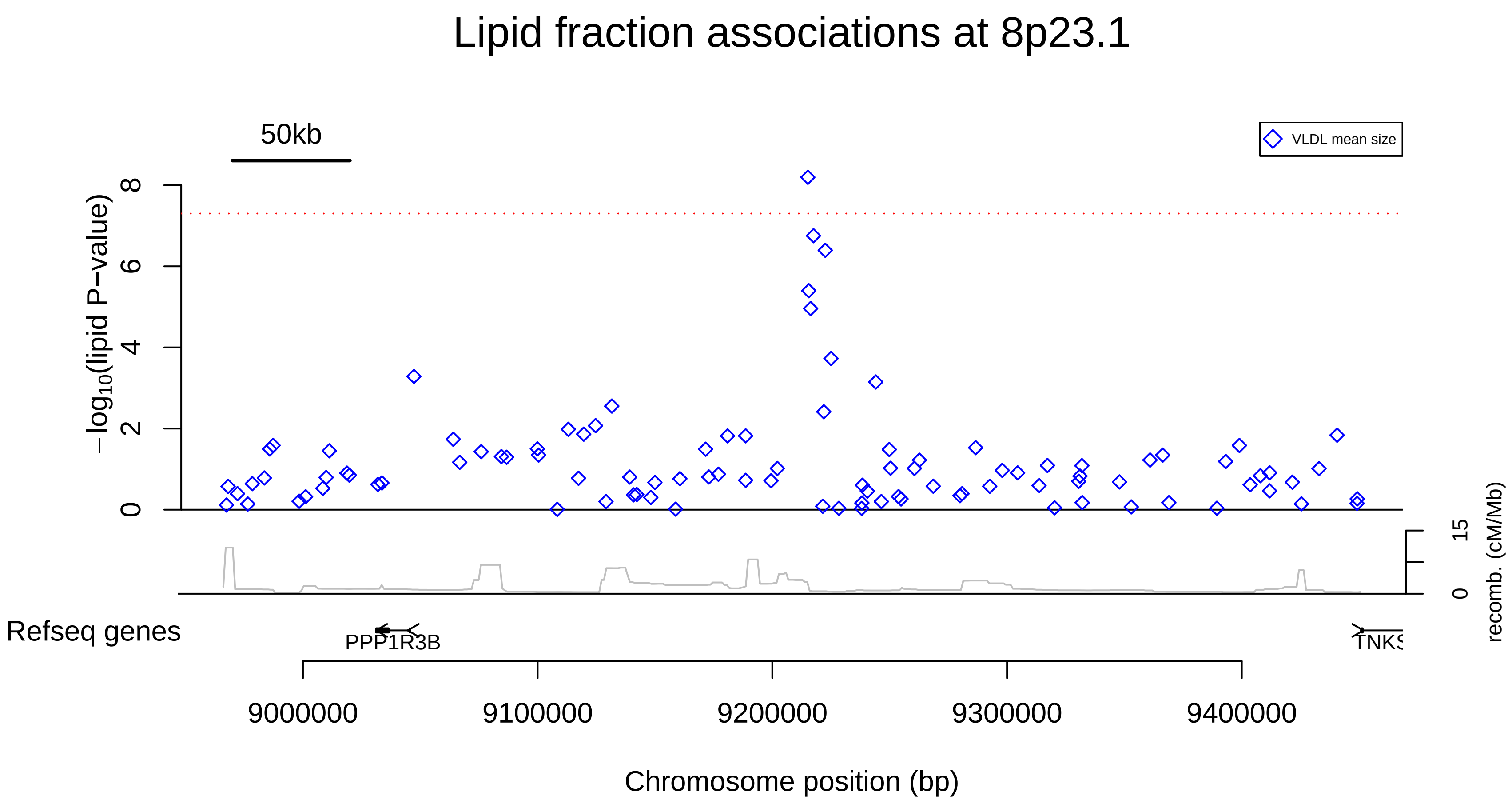

Lipid fraction associations at 8p23.1
50kb
VLDL mean size
−log10(lipid P−value)
0
2
4
6
8
15
0
recomb. (cM/Mb)
Refseq genes
PPP1R3B
TNKS
9000000
9100000
9200000
9300000
9400000
Chromosome position (bp)

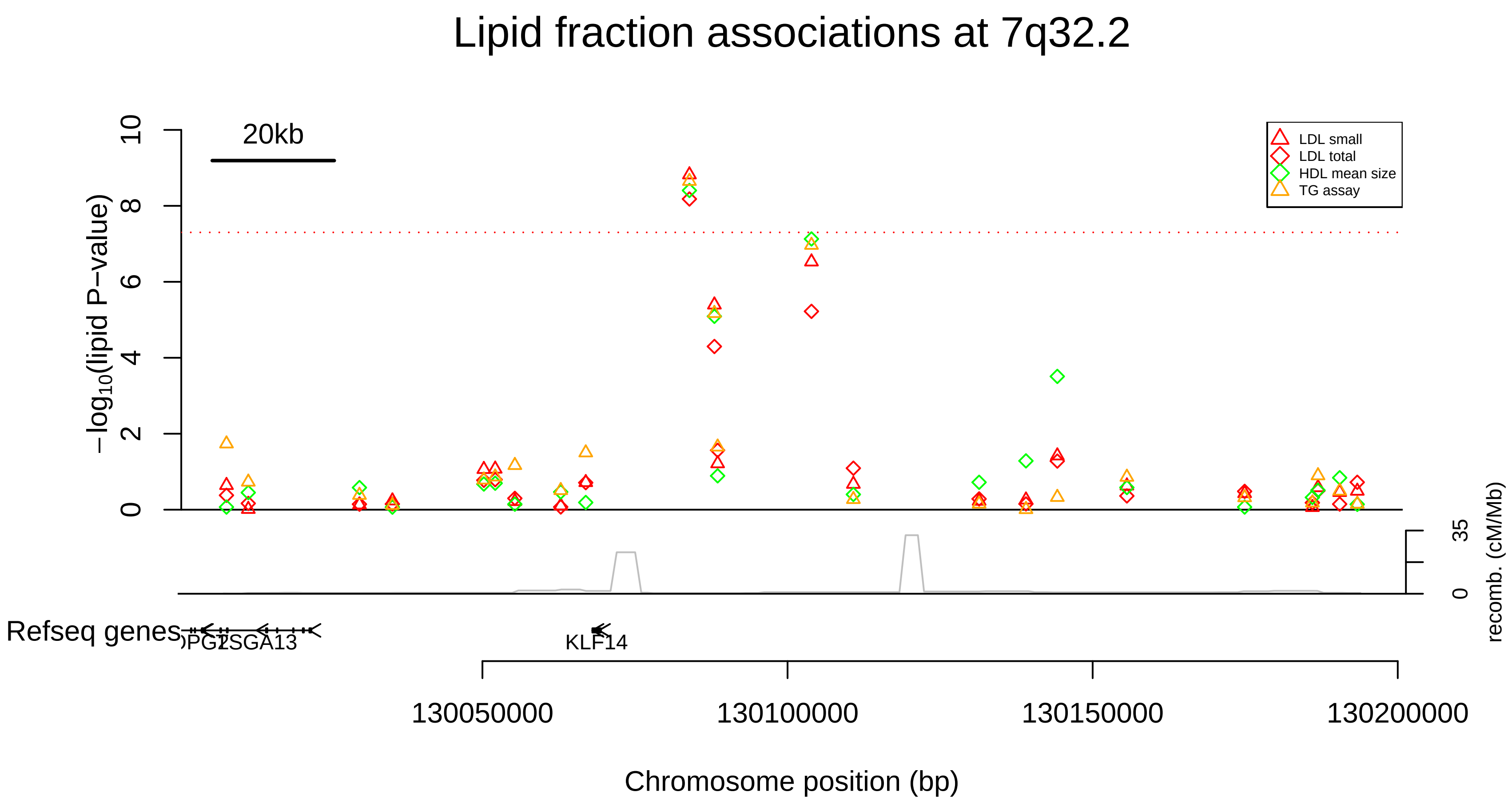
Lipid fraction associations at 7q32.2
20kb
LDL small
LDL total
HDL mean size
TG assay
−log10(lipid P−value)
0
2
4
6
8
10
recomb. (cM/Mb)
0
35
Refseq genes
KLF14
130050000
130100000
130150000
130200000
Chromosome position (bp)

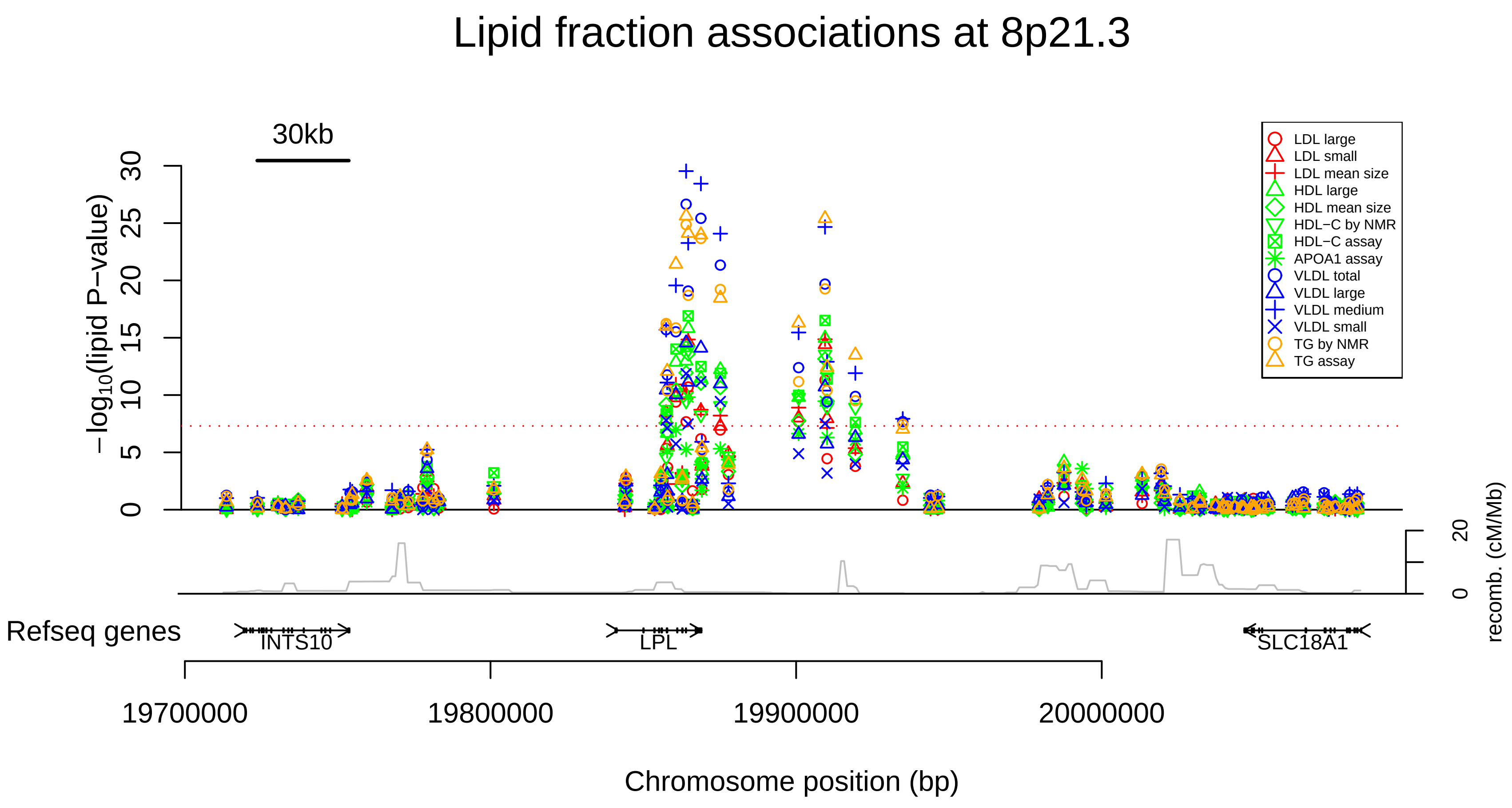

Lipid fraction associations at 8p21.3
30kb
−log10(lipid P−value)
0
5
10
15
20
25
30
LDL large
LDL small
LDL mean size
HDL large
HDL mean size
HDL−C by NMR
HDL−C assay
APOA1 assay
VLDL total
VLDL large
VLDL medium
VLDL small
TG by NMR
TG assay
recomb. (cM/Mb)
0
20
Refseq genes
INTS10
LPL
SLC18A1
19700000
19800000
19900000
20000000
Chromosome position (bp)

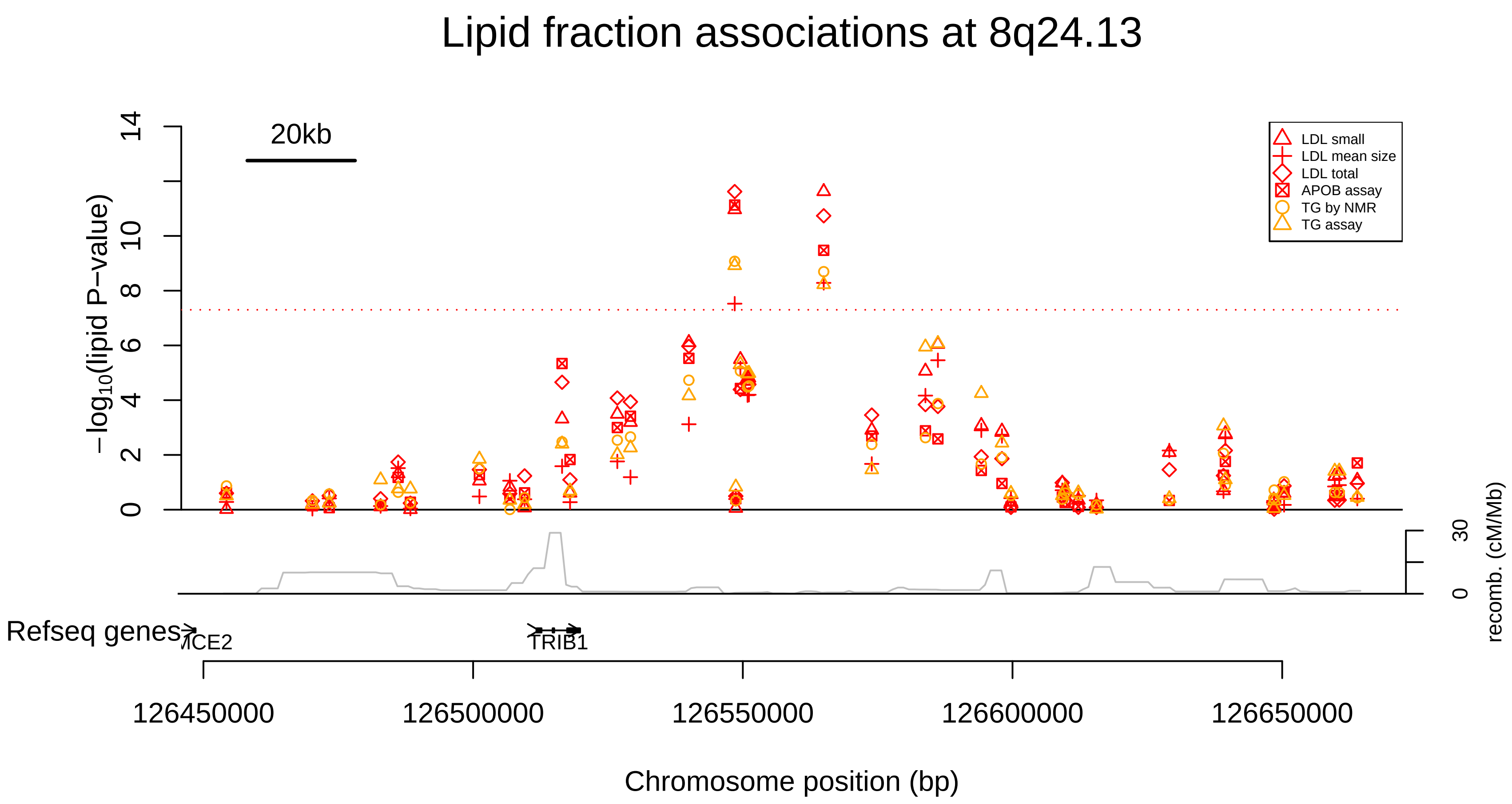
Lipid fraction associations at 8q24.13
20kb
−log10(lipid P−value)
0
2
4
6
8
10
14
LDL small
LDL mean size
LDL total
APOB assay
TG by NMR
TG assay
30
0
recomb. (cM/Mb)
Refseq genes
TRIB1
126450000
126500000
126550000
126600000
126650000
Chromosome position (bp)

## Lipid fraction associations at 9q31.1

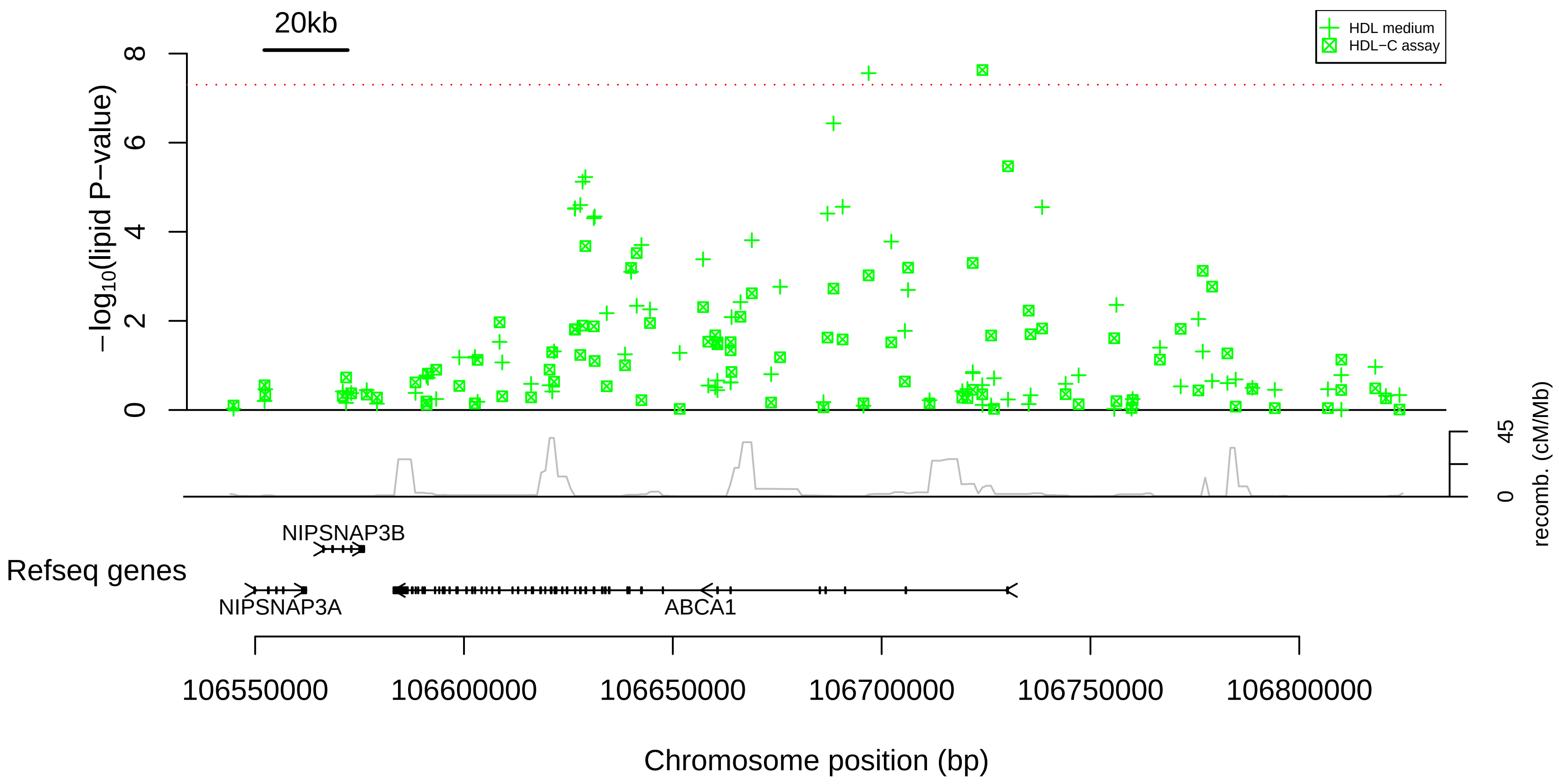

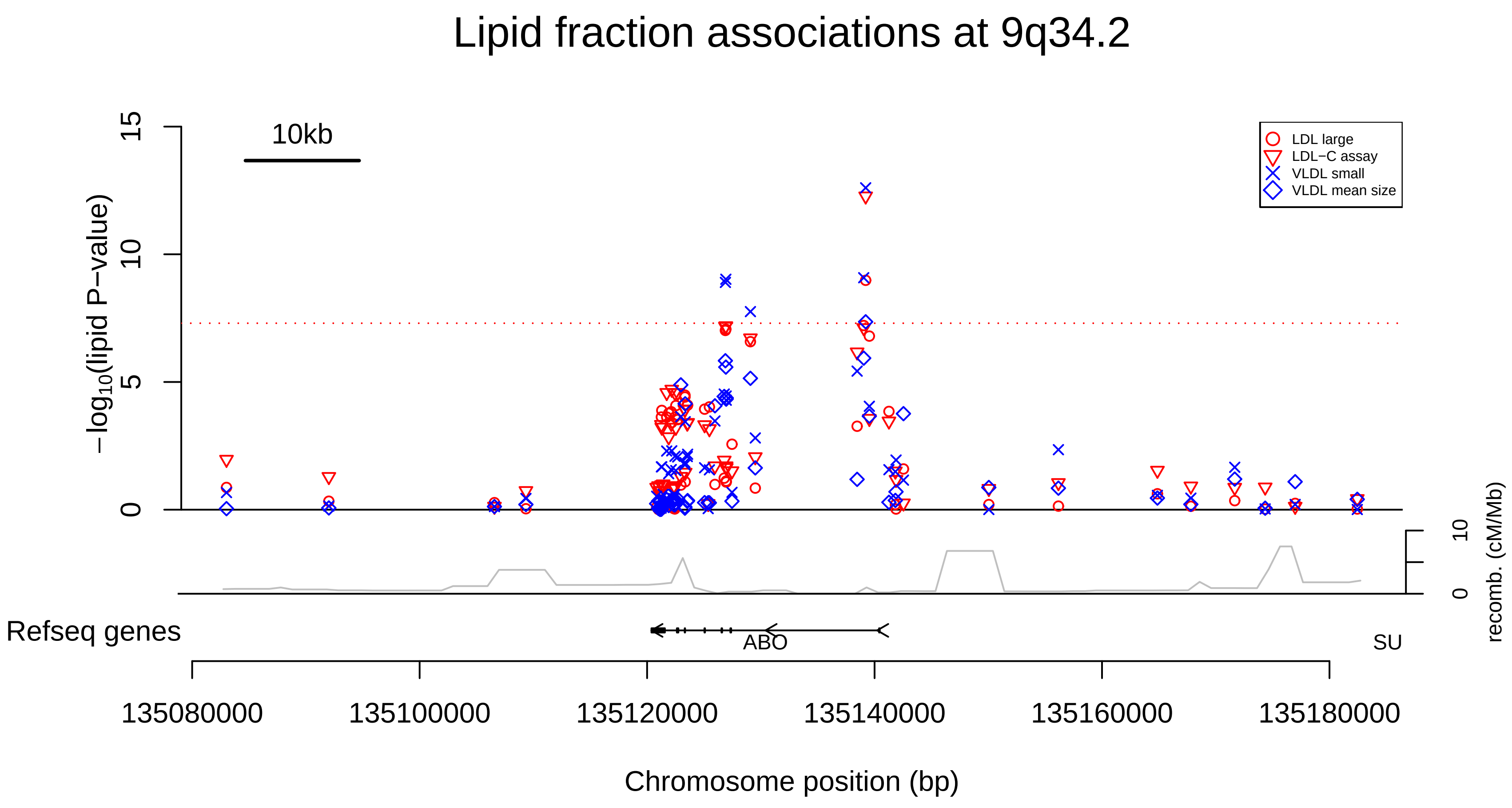
Lipid fraction associations at 9q34.2
10kb
LDL large
LDL−C assay
VLDL small
VLDL mean size
−log10(lipid P−value)
0
5
10
15
recomb. (cM/Mb)
0
10
Refseq genes
ABO
SU
135080000
135100000
135120000
135140000
135160000
135180000
Chromosome position (bp)

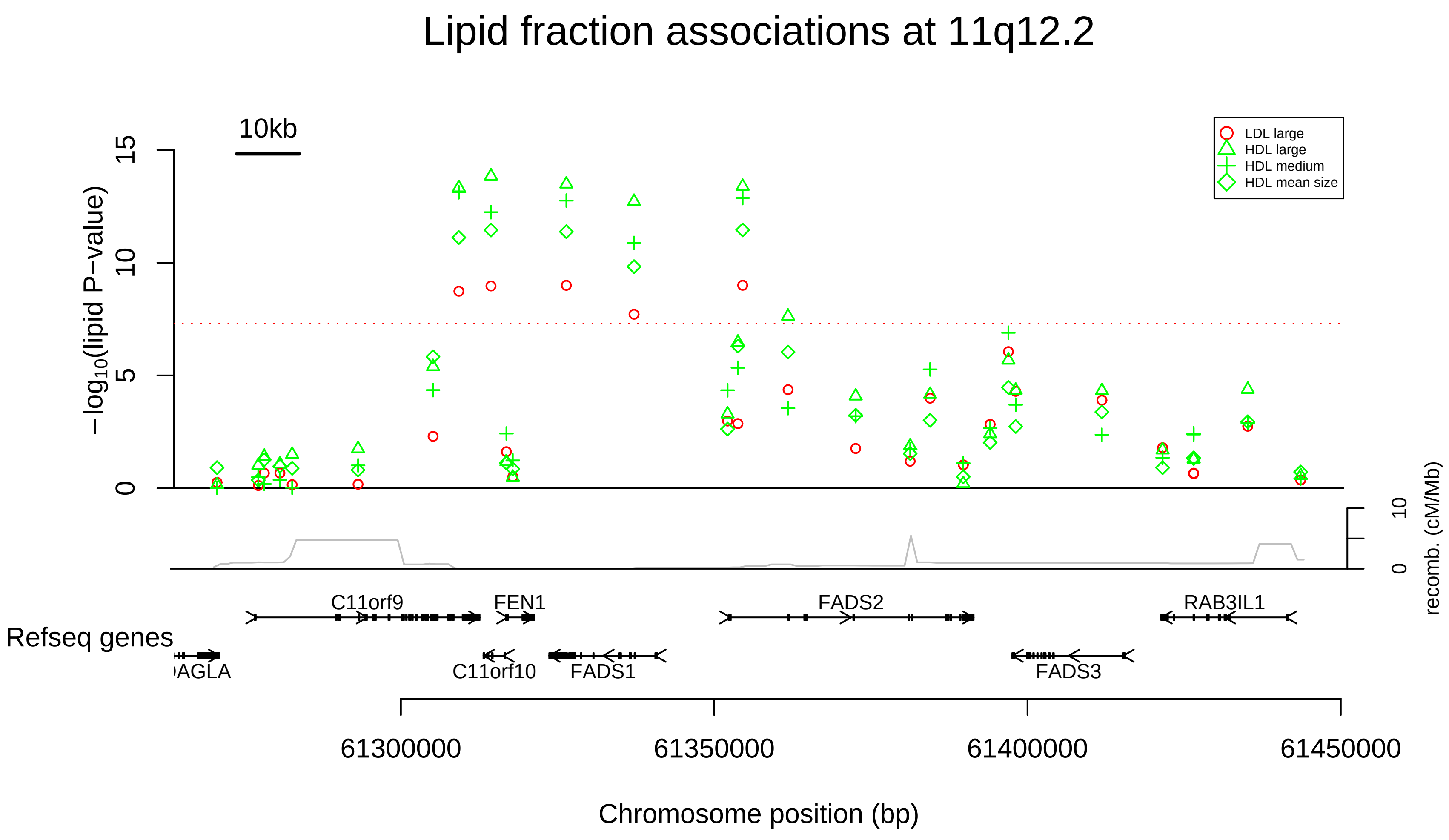

Lipid fraction associations at 11q12.2
10kb
−log10(lipid P−value)
0
5
10
15
LDL large
HDL large
HDL medium
HDL mean size
recomb. (cM/Mb)
0
10
Refseq genes
C11orf9
FEN1
FADS2
RAB3IL1
AGLA
C11orf10
FADS1
FADS3
61300000
61350000
61400000
61450000
Chromosome position (bp)

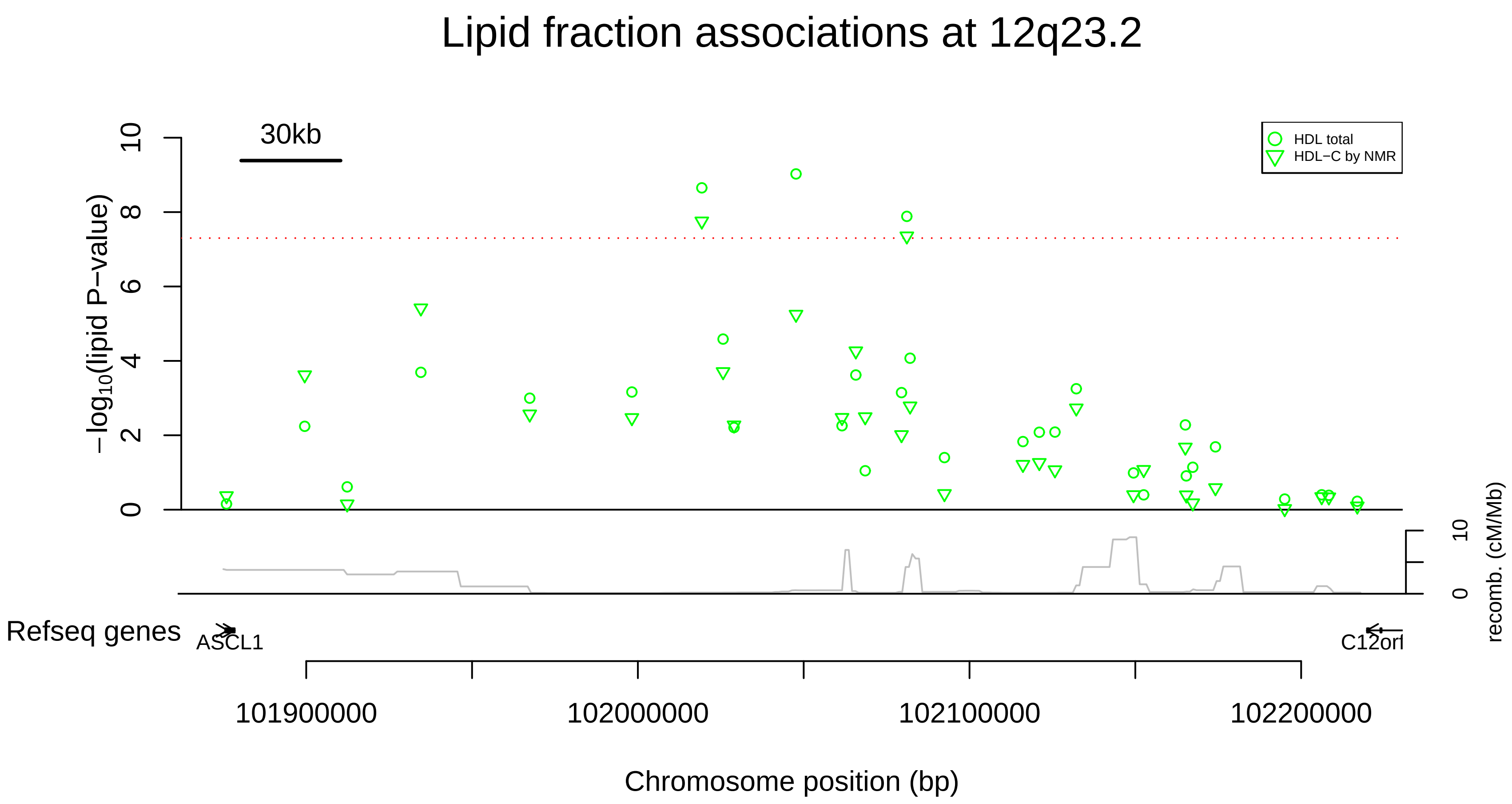
Lipid fraction associations at 12q23.2
30kb
HDL total
HDL−C by NMR
−log10(lipid P−value)
0
2
4
6
8
10
recomb. (cM/Mb)
0
10
Refseq genes
ASCL1
C12orf
101900000
102000000
102100000
102200000
Chromosome position (bp)

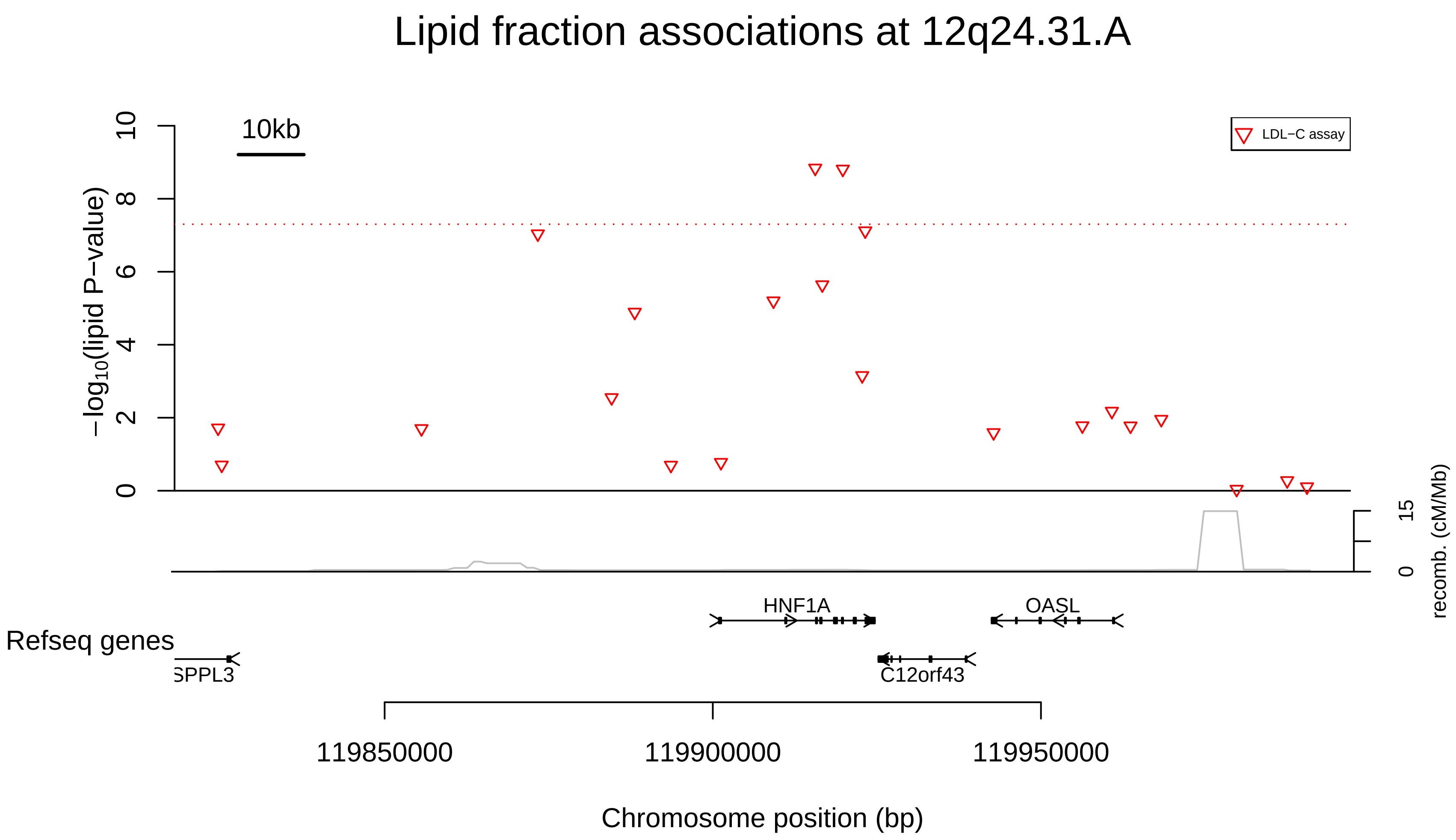

Lipid fraction associations at 12q24.31.A
10kb
LDL−C assay
$-\log_{10}$(lipid P−value)
0
2
4
6
8
10
15
0
recomb. (cM/Mb)
Refseq genes
HNF1A
OASL
SPPL3
C12orf43
119850000
119900000
119950000
Chromosome position (bp)

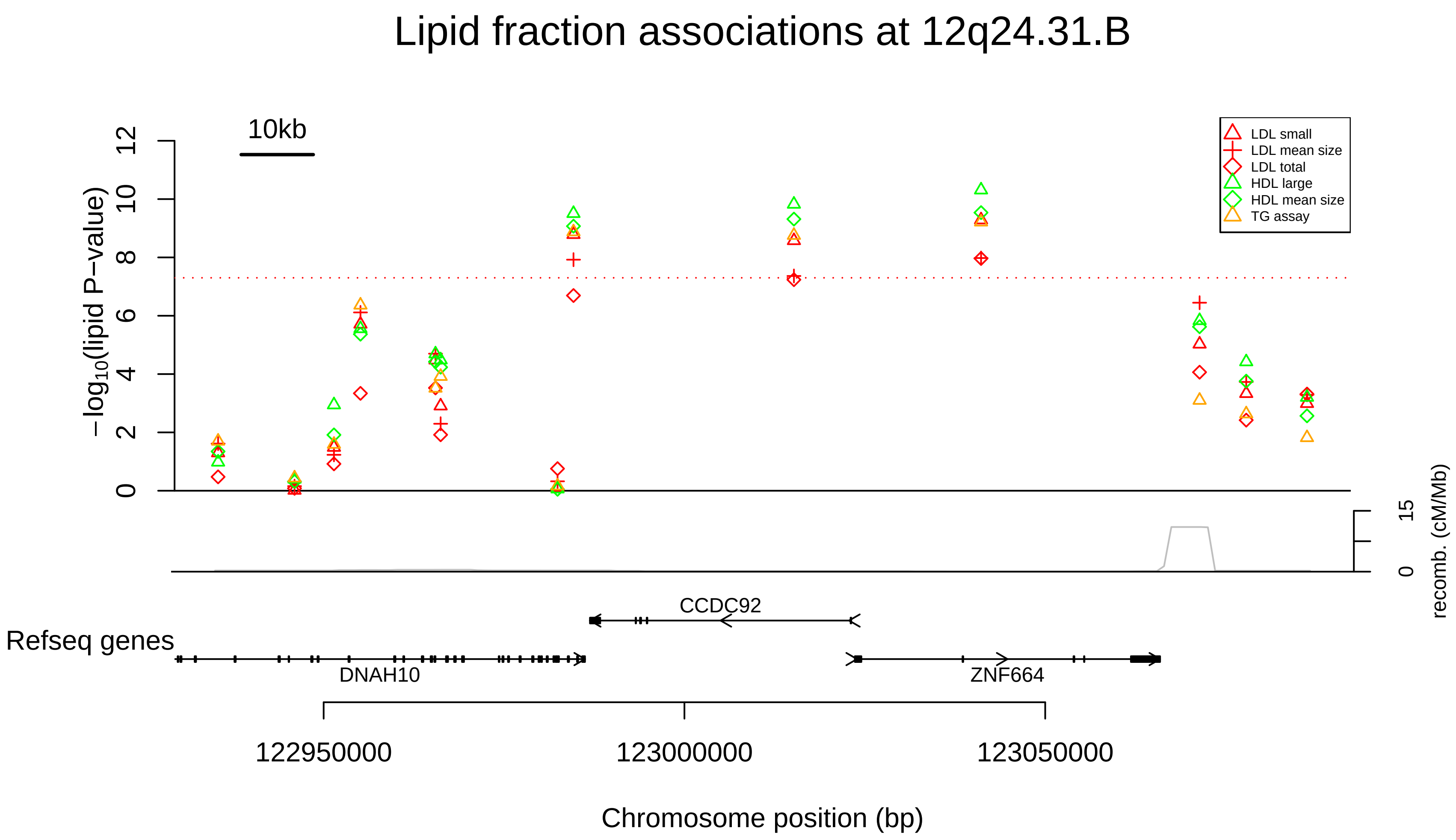
Lipid fraction associations at 12q24.31.B
10kb
−log10(lipid P−value)
0
2
4
6
8
10
12
LDL small
LDL mean size
LDL total
HDL large
HDL mean size
TG assay
15
0
recomb. (cM/Mb)
Refseq genes
CCDC92
DNAH10
ZNF664
122950000
123000000
123050000
Chromosome position (bp)

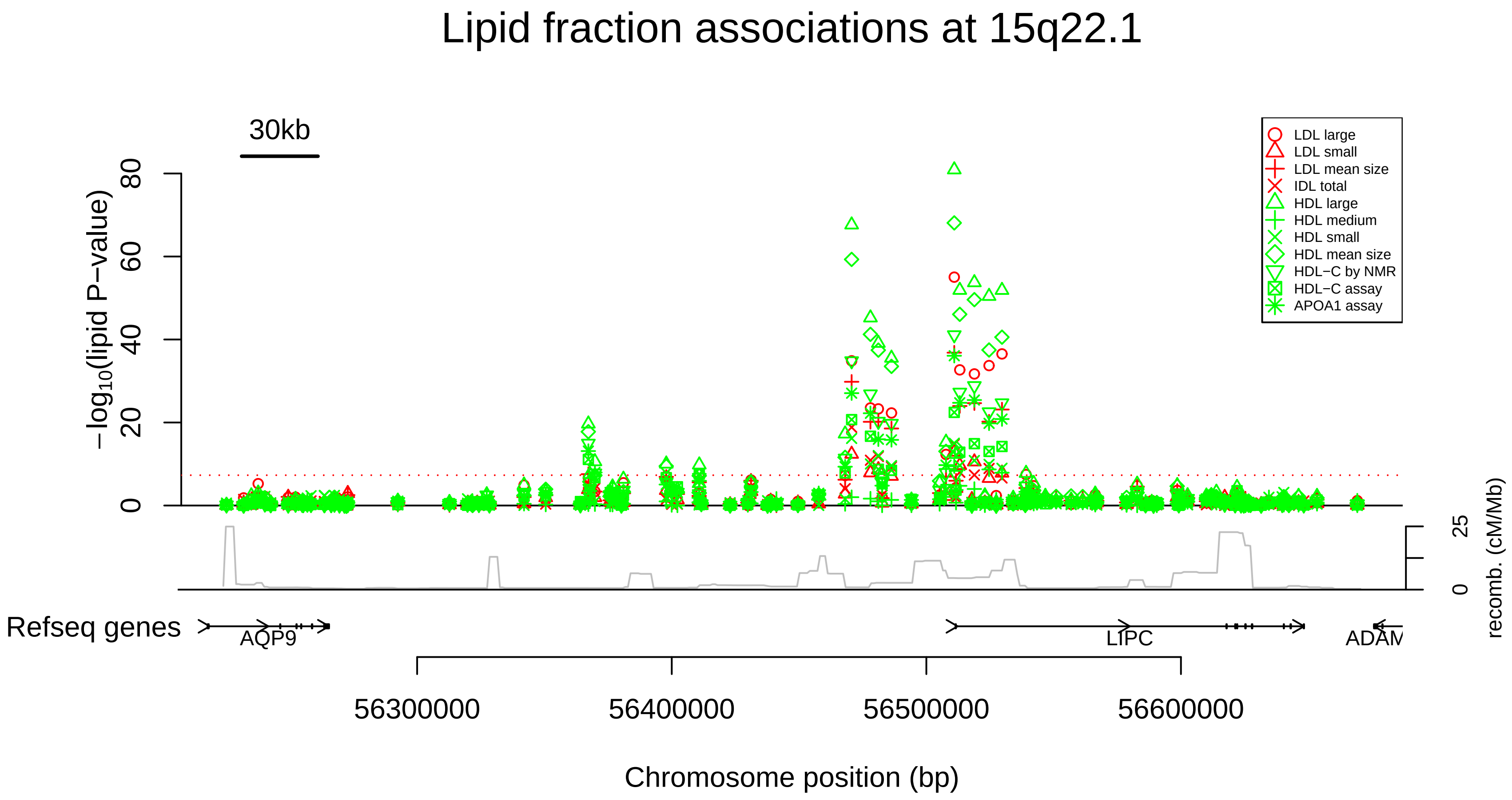

Lipid fraction associations at 15q22.1
30kb
−log10(lipid P−value)
0
20
40
60
80
LDL large
LDL small
LDL mean size
IDL total
HDL large
HDL medium
HDL small
HDL mean size
HDL−C by NMR
HDL−C assay
APOA1 assay
recomb. (cM/Mb)
0
25
Refseq genes
AQP9
LIPC
ADAM
56300000
56400000
56500000
56600000
Chromosome position (bp)

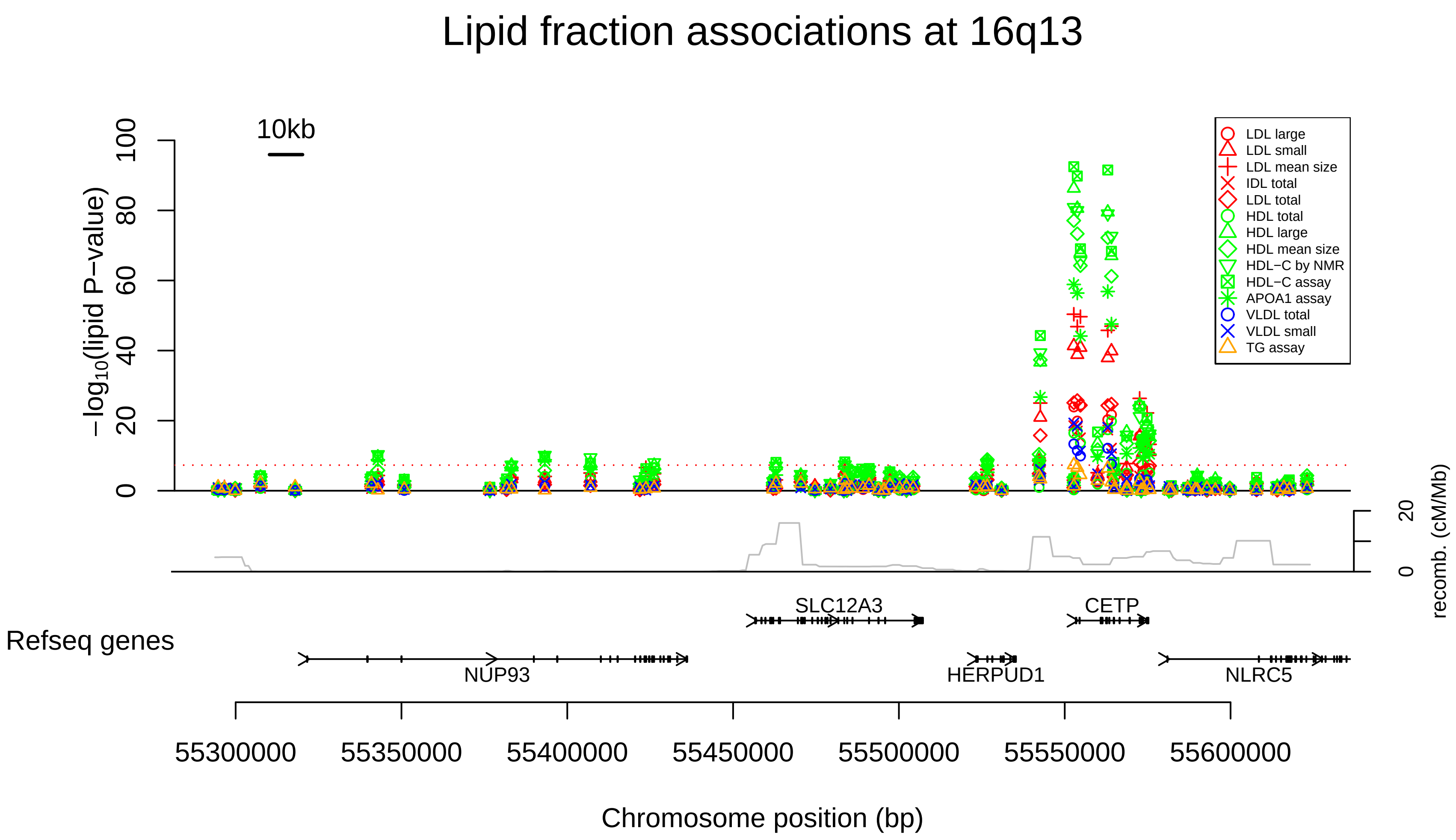

Lipid fraction associations at 16q13
10kb
−log10(lipid P−value)
0
20
40
60
80
100
LDL large
LDL small
LDL mean size
IDL total
LDL total
HDL total
HDL large
HDL mean size
HDL−C by NMR
HDL−C assay
APOA1 assay
VLDL total
VLDL small
TG assay
recomb. (cM/Mb)
0
20
Refseq genes
SLC12A3
CETP
NUP93
HERPUD1
NLRC5
55300000
55350000
55400000
55450000
55500000
55550000
55600000
Chromosome position (bp)

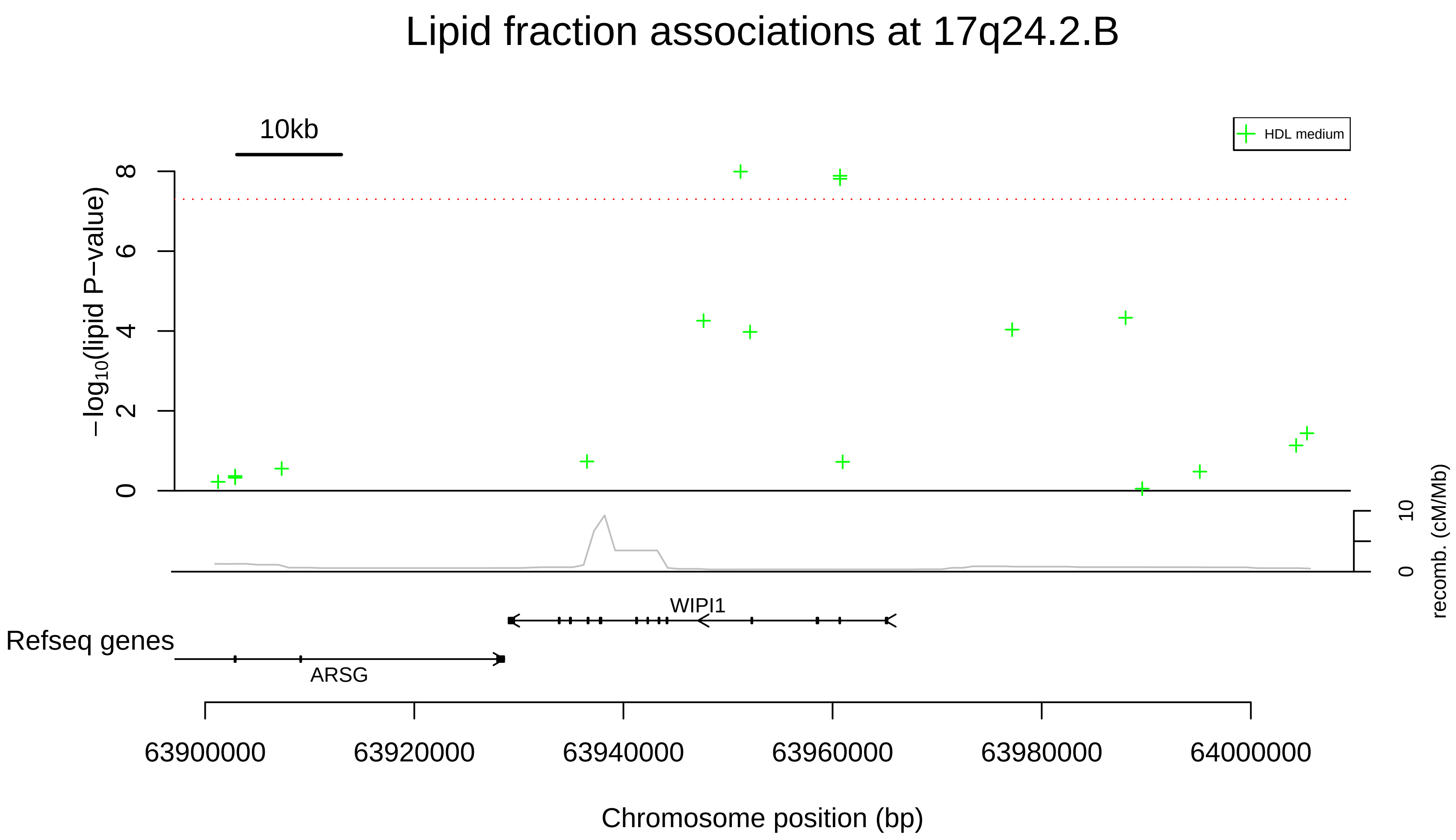

Lipid fraction associations at 17q24.2.B
10kb
HDL medium
$-\log_{10}$(lipid P−value)
0
2
4
6
8
10
0
recomb. (cM/Mb)
WIPI1
Refseq genes
ARSG
63900000
63920000
63940000
63960000
63980000
64000000
Chromosome position (bp)

Lipid fraction associations at 18q21.1

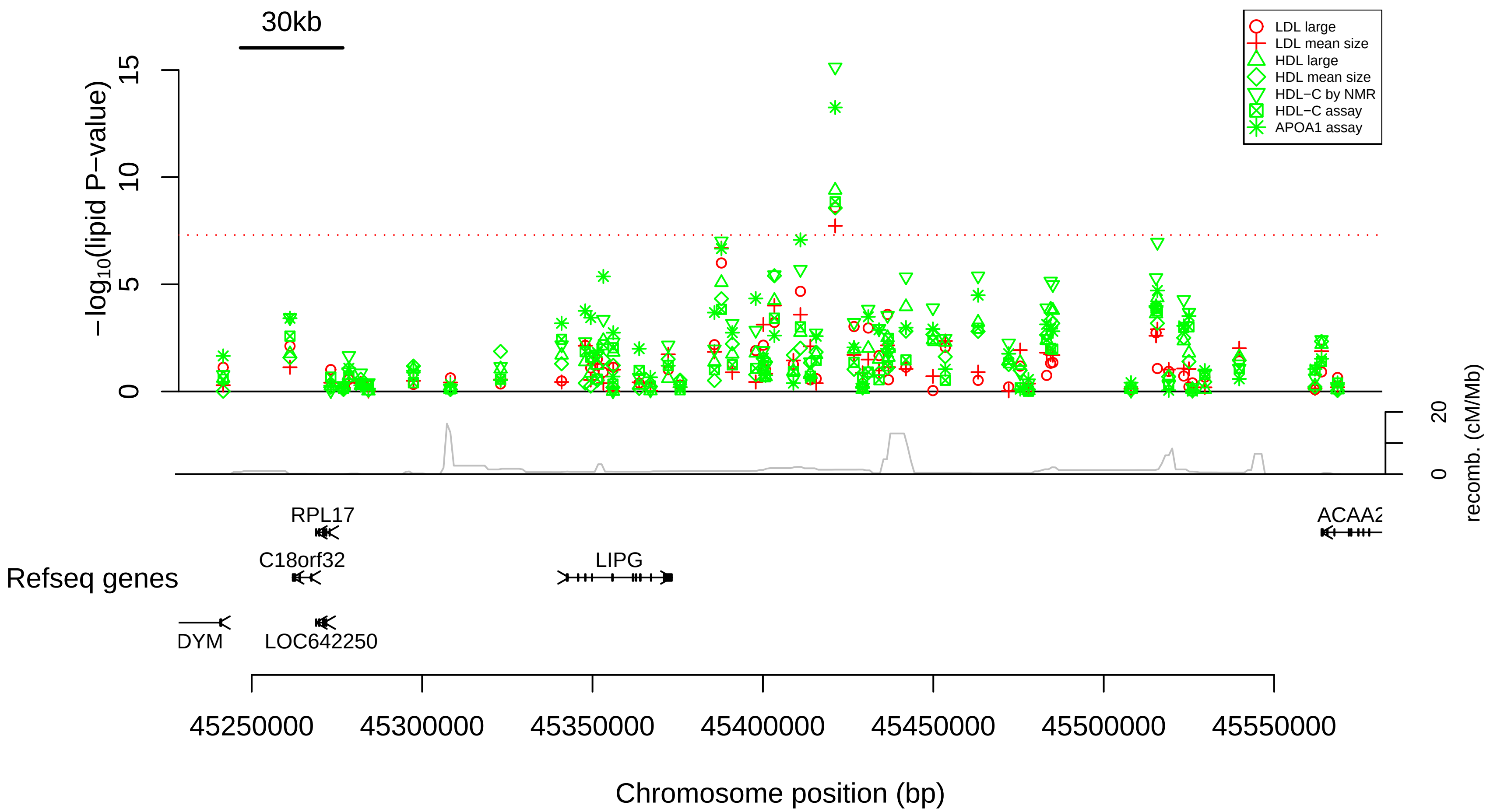

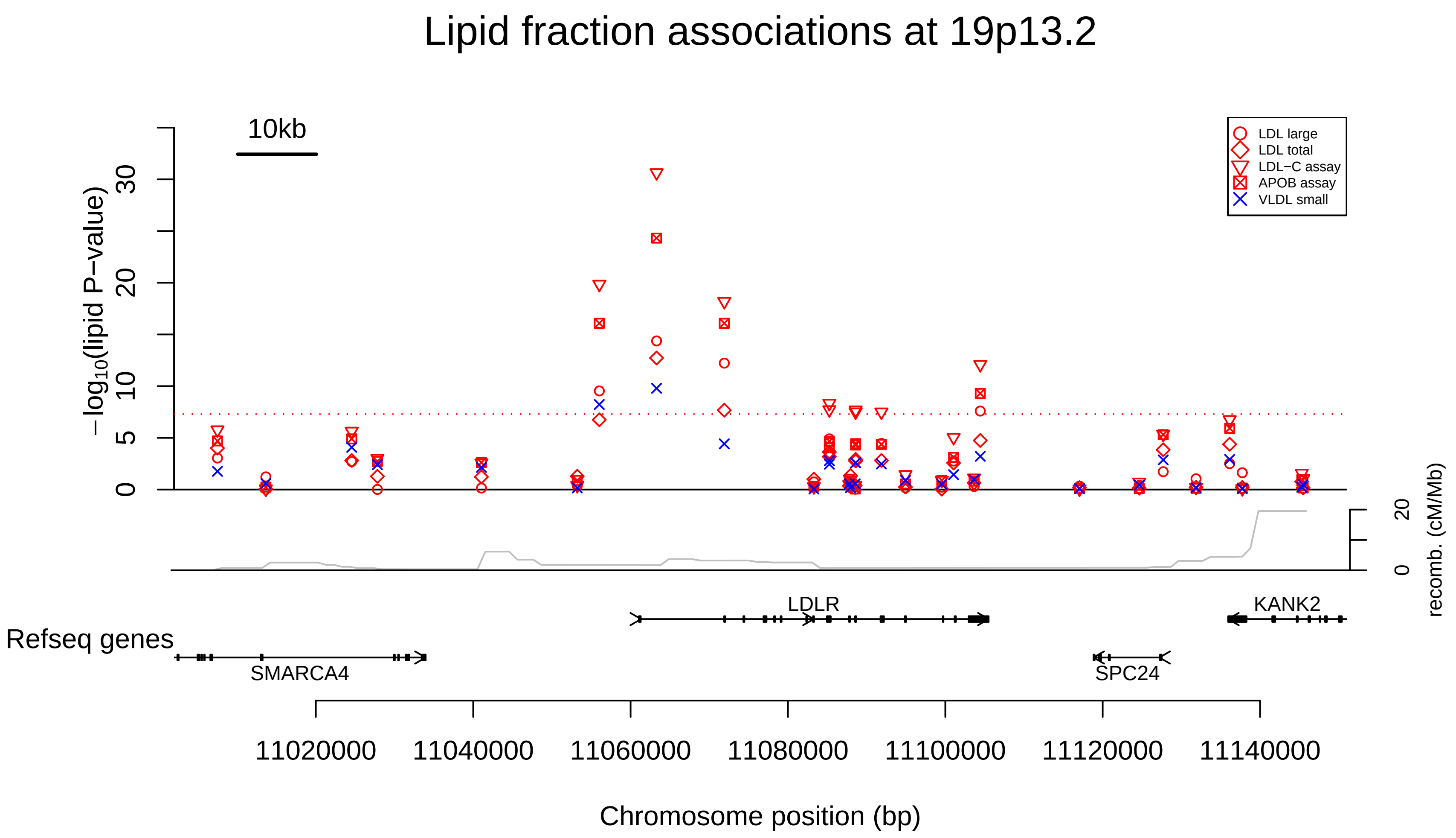
Lipid fraction associations at 19p13.2
10kb
−log10(lipid P−value)
0
5
10
20
30
LDL large
LDL total
LDL−C assay
APOB assay
VLDL small
20
0
recomb. (cM/Mb)
Refseq genes
LDLR
KANK2
SMARCA4
SPC24
11020000
11040000
11060000
11080000
11100000
11120000
11140000
Chromosome position (bp)

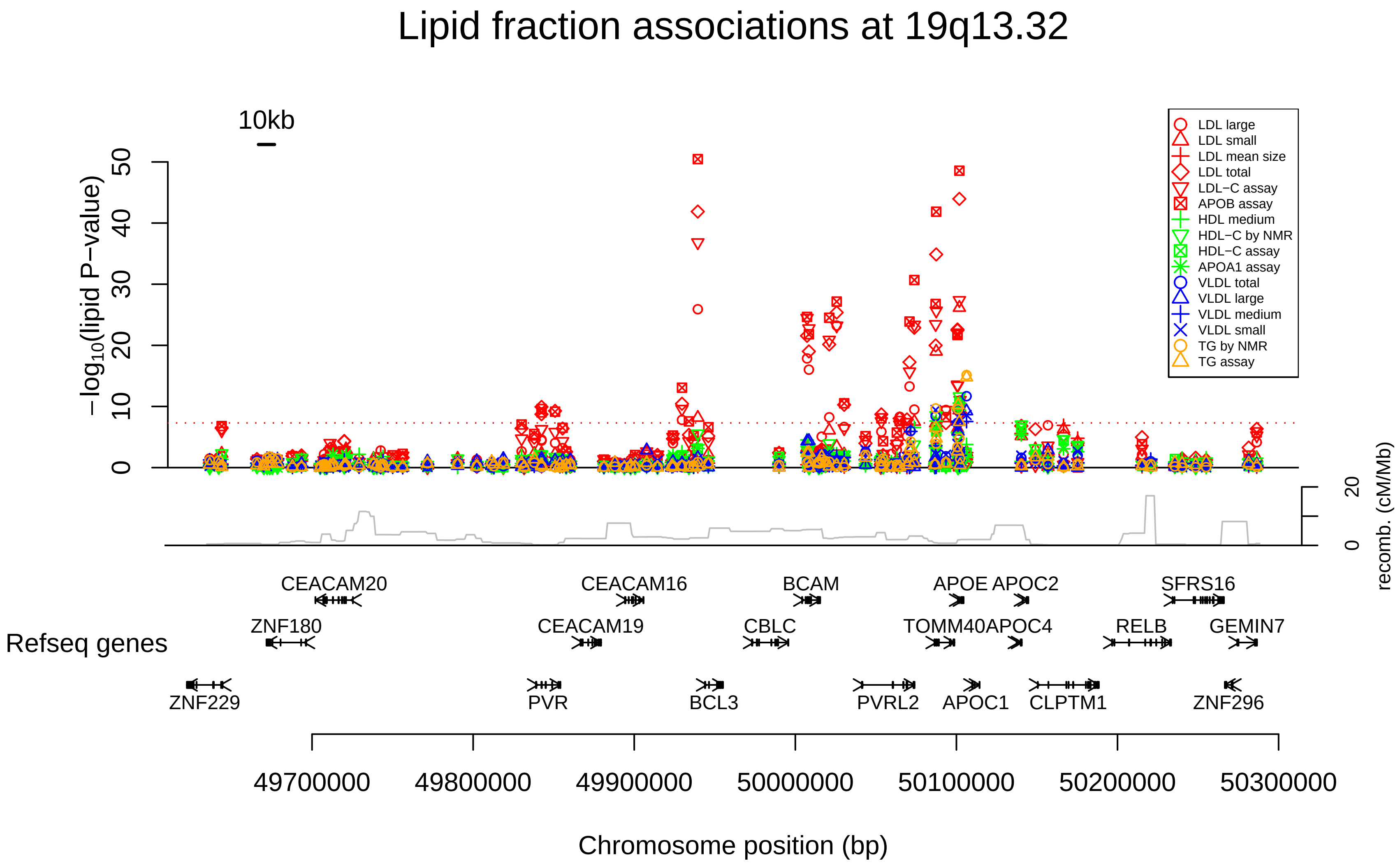

Lipid fraction associations at 19q13.32
10kb
−log10(lipid P−value)
0
10
20
30
40
50
LDL large
LDL small
LDL mean size
LDL total
LDL−C assay
APOB assay
HDL medium
HDL−C by NMR
HDL−C assay
APOA1 assay
VLDL total
VLDL large
VLDL medium
VLDL small
TG by NMR
TG assay
recomb. (cM/Mb)
0
20
Refseq genes
CEACAM20
CEACAM16
BCAM
APOE APOC2
SFRS16
ZNF180
CEACAM19
CBLC
TOMM40APOC4
RELB
GEMIN7
ZNF229
PVR
BCL3
PVRL2
APOC1
CLPTM1
ZNF296
49700000
49800000
49900000
50000000
50100000
50200000
50300000
Chromosome position (bp)

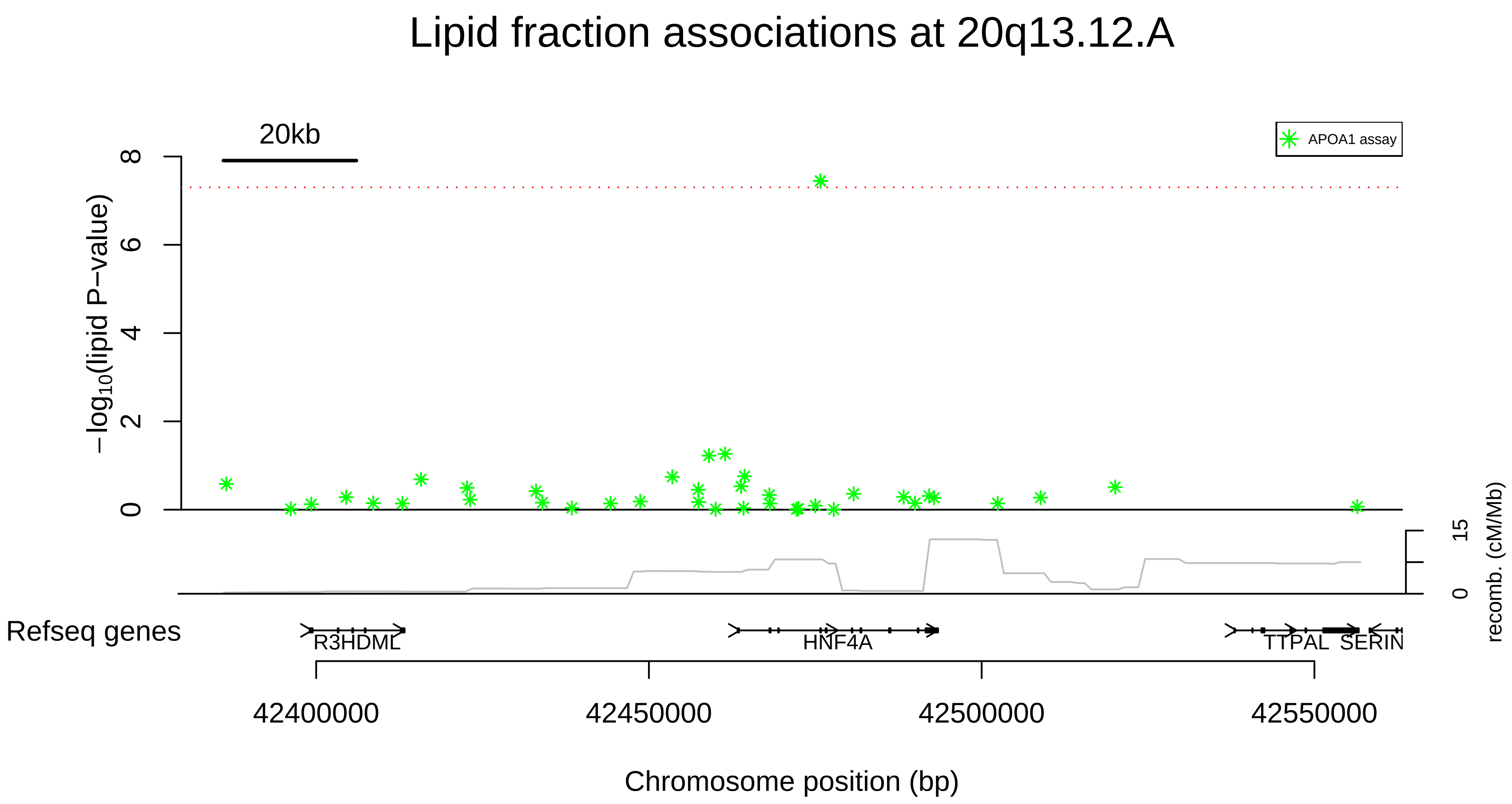
Lipid fraction associations at 20q13.12.A
20kb
APOA1 assay
−log10(lipid P−value)
0
2
4
6
8
15
0
recomb. (cM/Mb)
Refseq genes
R3HDML
HNF4A
TTPAL
SERIN
42400000
42450000
42500000
42550000
Chromosome position (bp)

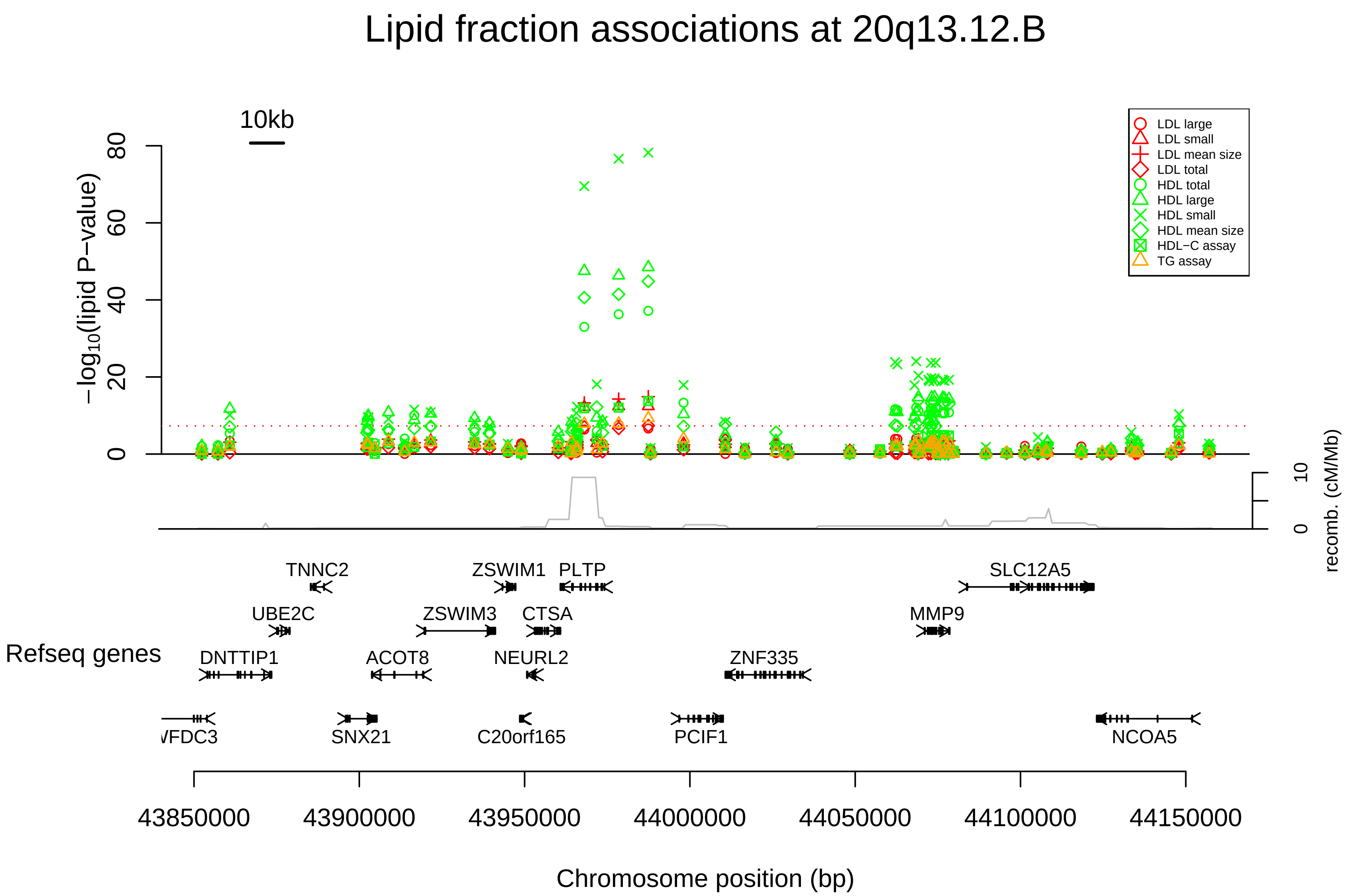
Lipid fraction associations at 20q13.12.B
10kb
−log10(lipid P−value)
0
20
40
60
80
LDL large
LDL small
LDL mean size
LDL total
HDL total
HDL large
HDL small
HDL mean size
HDL−C assay
TG assay
recomb. (cM/Mb)
0
10
Refseq genes
TNNC2
ZSWIM1
PLTP
SLC12A5
UBE2C
ZSWIM3
CTSA
MMP9
DNTTIP1
ACOT8
NEURL2
ZNF335
WFDC3
SNX21
C20orf165
PCIF1
NCOA5
43850000
43900000
43950000
44000000
44050000
44100000
44150000
Chromosome position (bp)
