# Supplementary material for: Forty-Three Loci Associated with Plasma Lipoprotein Size, Concentration, and Cholesterol Content in Genome-Wide Analysis
Source: PLoS Genet. 2009 Nov 20;5(11):e1000730. doi: 10.1371/journal.pgen.1000730 (PMC2777390; doi:10.1371/journal.pgen.1000730)
Supplement: Figure S2 — Primary loci clustered hierarchically according to Cartesian distance corresponding to whether ( = 1) or not ( = 0) there were associations with each of the lipoprotein fractions in the model selection procedures (see Materials and Methods). (0.02 MB PDF) [file pgen.1000730.s002.pdf]

Figure S2. Primary loci clustered hierarchically according to Cartesian distance corresponding to whether (=1) or not (=0) there were associations with each of the lipoprotein fractions in the model selection procedures (see Methods).

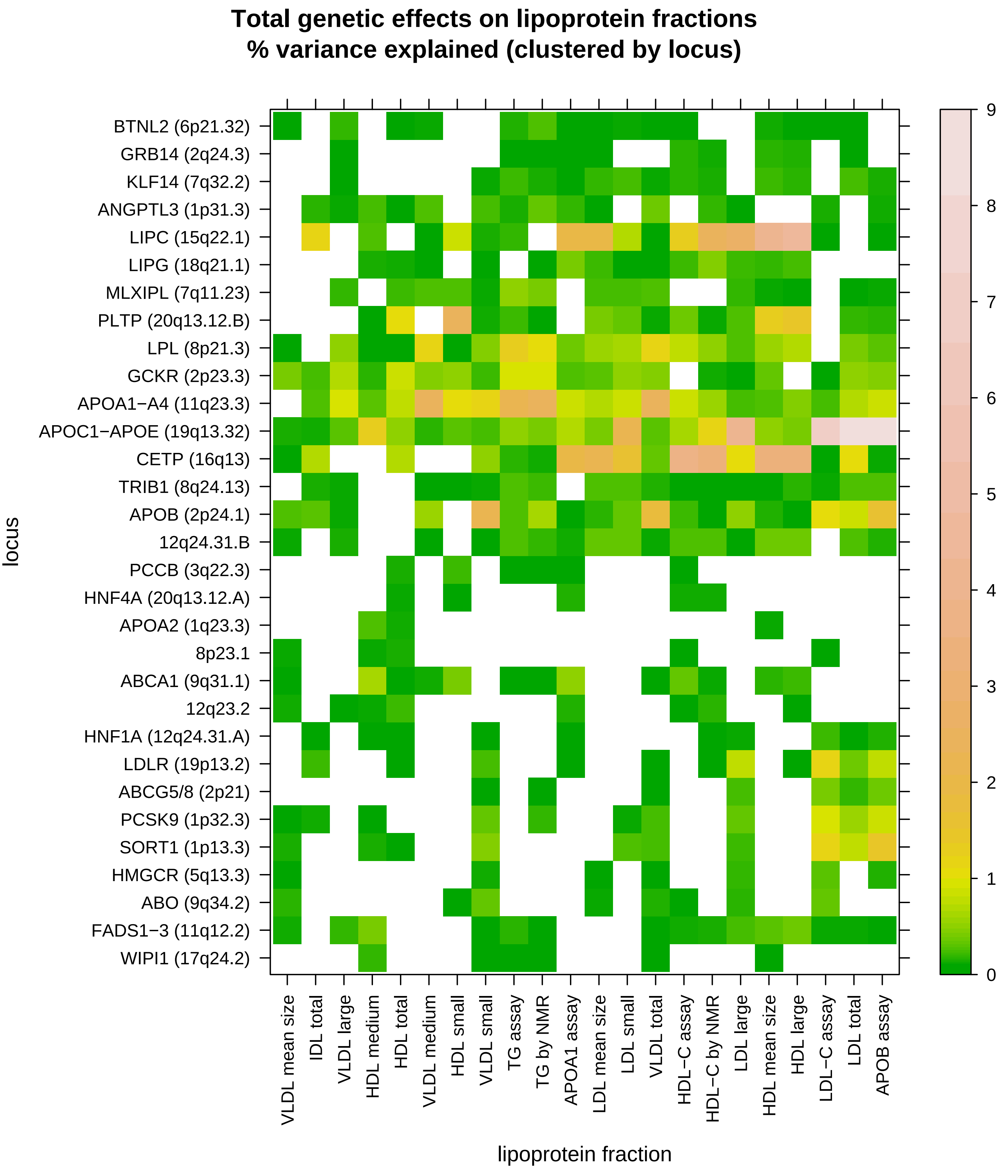
Total genetic effects on lipoprotein fractions
% variance explained (clustered by locus)
locus
BTNL2 (6p21.32)
GRB14 (2q24.3)
KLF14 (7q32.2)
ANGPTL3 (1p31.3)
LIPC (15q22.1)
LIPG (18q21.1)
MLXIPL (7q11.23)
PLTP (20q13.12.B)
LPL (8p21.3)
GCKR (2p23.3)
APOA1−A4 (11q23.3)
APOC1−APOE (19q13.32)
CETP (16q13)
TRIB1 (8q24.13)
APOB (2p24.1)
12q24.31.B
PCCB (3q22.3)
HNF4A (20q13.12.A)
APOA2 (1q23.3)
8p23.1
ABCA1 (9q31.1)
12q23.2
HNF1A (12q24.31.A)
LDLR (19p13.2)
ABCG5/8 (2p21)
PCSK9 (1p32.3)
SORT1 (1p13.3)
HMGCR (5q13.3)
ABO (9q34.2)
FADS1−3 (11q12.2)
WIPI1 (17q24.2)
VLDL mean size
IDL total
VLDL large
HDL medium
HDL total
VLDL medium
HDL small
VLDL small
TG assay
TG by NMR
APOA1 assay
LDL mean size
LDL small
VLDL total
HDL−C assay
HDL−C by NMR
LDL large
HDL mean size
HDL large
LDL−C assay
LDL total
APOB assay
lipoprotein fraction
0
1
2
3
4
5
6
7
8
9
