# Supplementary material for: Forty-Three Loci Associated with Plasma Lipoprotein Size, Concentration, and Cholesterol Content in Genome-Wide Analysis
Source: PLoS Genet. 2009 Nov 20;5(11):e1000730. doi: 10.1371/journal.pgen.1000730 (PMC2777390; doi:10.1371/journal.pgen.1000730)

Figure S3. Dendorgram showing bierarchical relationships between loci clustered as in Figure S2.

Loci, clustered by effects on lipoprotein fractions

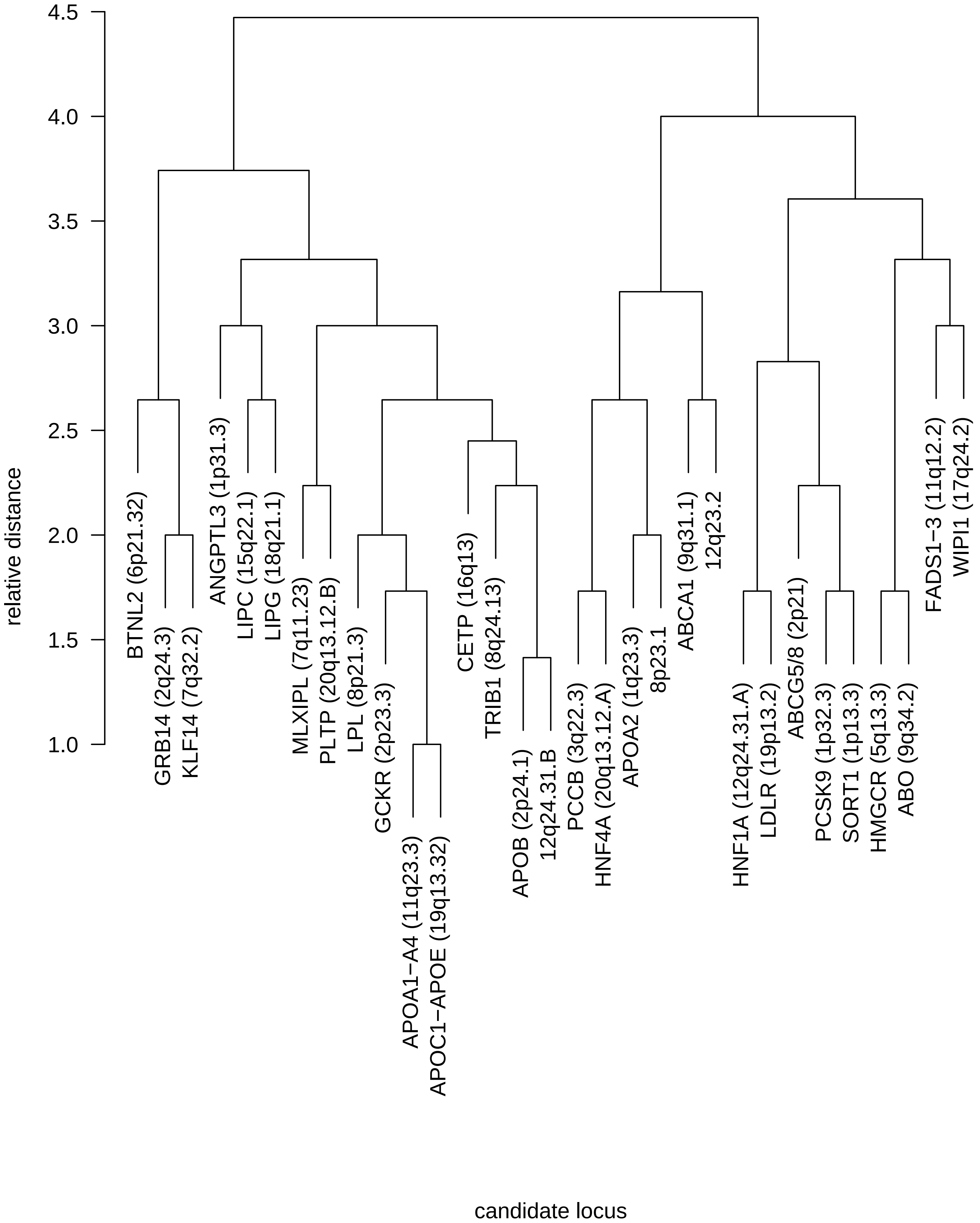

Supplement: Figure S3 — Dendorgram showing bierarchical relationships between loci clustered as in Figure S2. (0.01 MB PDF) [file pgen.1000730.s003.pdf]
