# Supplementary material for: Forty-Three Loci Associated with Plasma Lipoprotein Size, Concentration, and Cholesterol Content in Genome-Wide Analysis
Source: PLoS Genet. 2009 Nov 20;5(11):e1000730. doi: 10.1371/journal.pgen.1000730 (PMC2777390; doi:10.1371/journal.pgen.1000730)
Supplement: Figure S4 — Normalized SNP effects (beta coefficients) from univariate regression models. All plots correspond to analysis in the whole sample except for locus 8p23.1, for which genome-wide association was detected only in the fasting subsample as shown. Locus SNPs are shown if they were retained in the model selection procedure for at least one lipoprotein fraction. Absence of shading indicates the univariate beta coefficient was not significant (p>0.05). A small black dot for some combinations of SNPs and lipoprotein fractions indicates genomewide significance for the univariate beta coefficient. (0.10 MB PDF) [file pgen.1000730.s004.pdf]

Figure S4. Normalized SNP effects (beta coefficients) from univariate regression models. All plots correspond to analysis in the whole sample except for locus 8p23.1, for which genomewide association was detected only in the fasting subsample as shown. Locus SNPs are shown if they were retained in the model selection procedure for at least one lipoprotein fraction. Absence of shading indicates the univariate beta coefficient was not significant ($p>0.05$). A small black dot for some combinations of SNPs and lipoprotein fractions indicates genomewide significance for the univariate beta coefficient.

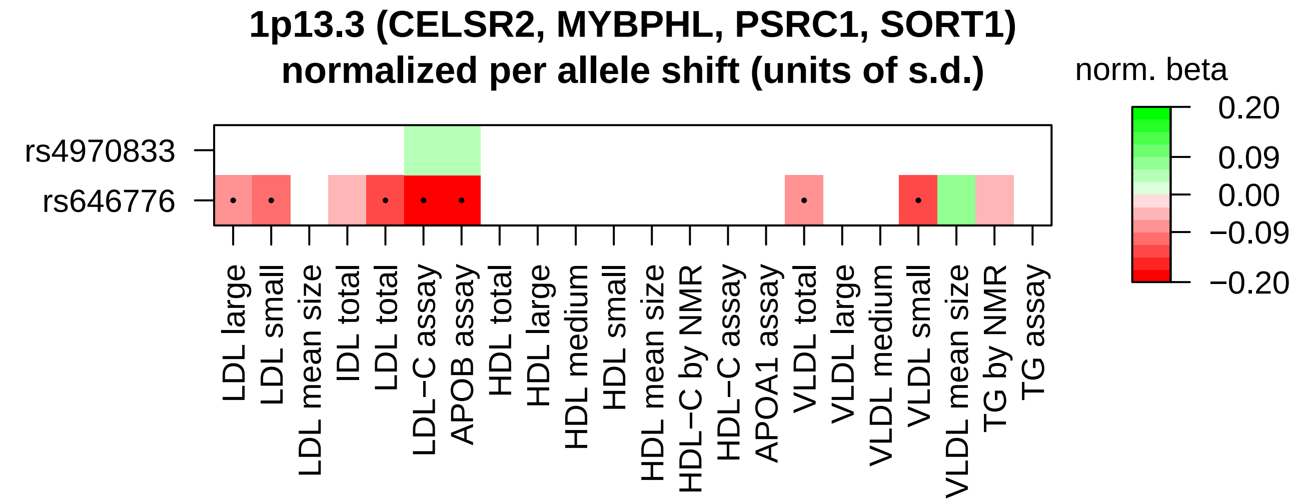
1p13.3 (CELSR2, MYBPHL, PSRC1, SORT1)
normalized per allele shift (units of s.d.)
norm. beta
0.20
0.09
0.00
−0.09
−0.20
rs4970833
rs646776
LDL large
LDL small
LDL mean size
IDL total
LDL total
LDL−C assay
APOB assay
HDL total
HDL large
HDL medium
HDL small
HDL mean size
HDL−C by NMR
HDL−C assay
APOA1 assay
VLDL total
VLDL large
VLDL medium
VLDL small
VLDL mean size
TG by NMR
TG assay

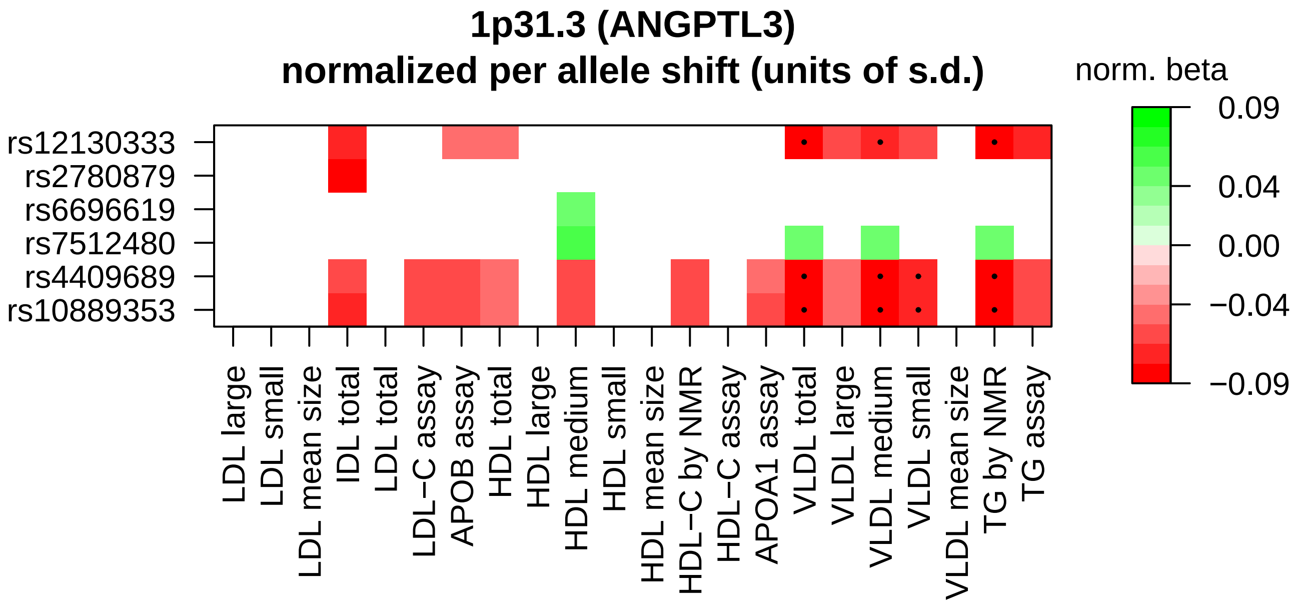
1p31.3 (ANGPTL3)
normalized per allele shift (units of s.d.)
norm. beta
0.09
0.04
0.00
−0.04
−0.09
rs12130333
rs2780879
rs6696619
rs7512480
rs4409689
rs10889353
LDL large
LDL small
LDL mean size
IDL total
LDL total
LDL−C assay
APOB assay
HDL total
HDL large
HDL medium
HDL small
HDL mean size
HDL−C by NMR
HDL−C assay
APOA1 assay
VLDL total
VLDL large
VLDL medium
VLDL small
VLDL mean size
TG by NMR
TG assay

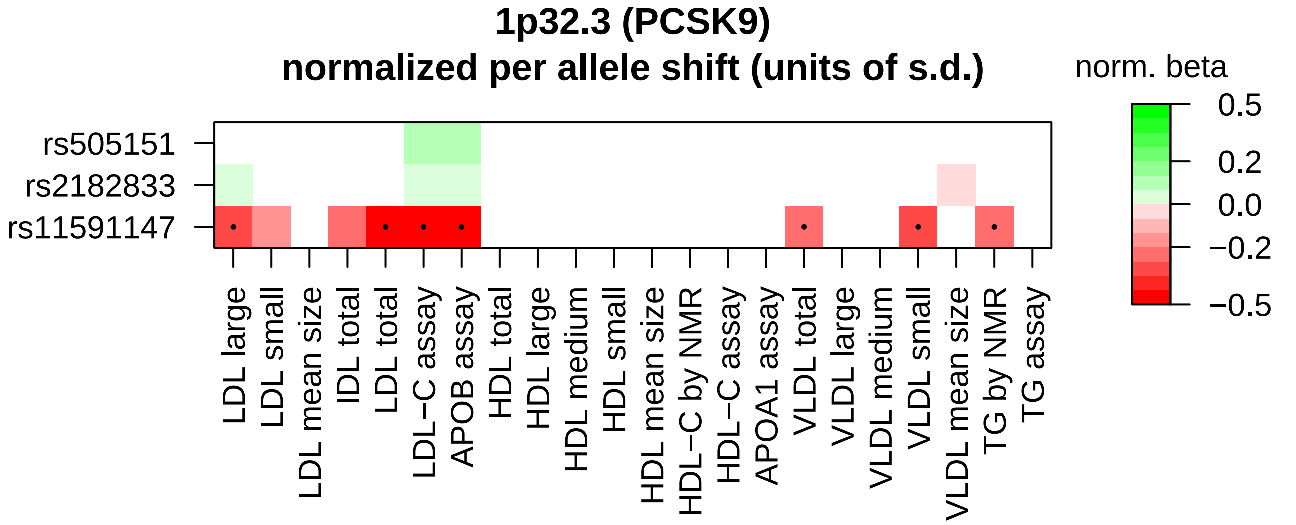
1p32.3 (PCSK9)
normalized per allele shift (units of s.d.)
norm. beta
0.5
0.2
0.0
−0.2
−0.5
rs505151
rs2182833
rs11591147
LDL large
LDL small
LDL mean size
IDL total
LDL total
LDL−C assay
APOB assay
HDL total
HDL large
HDL medium
HDL small
HDL mean size
HDL−C by NMR
HDL−C assay
APOA1 assay
VLDL total
VLDL large
VLDL medium
VLDL small
VLDL mean size
TG by NMR
TG assay

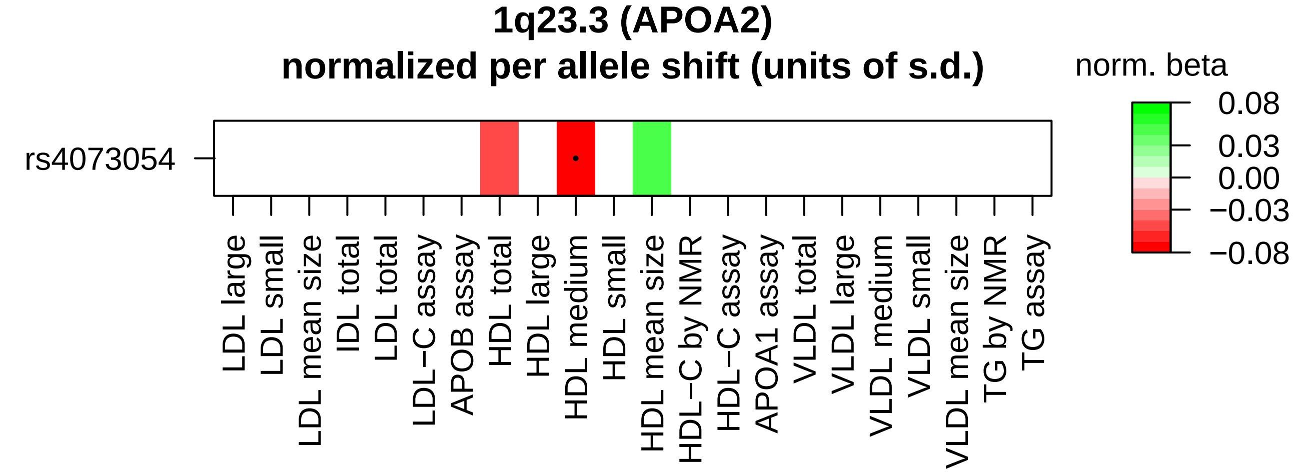
1q23.3 (APOA2)
normalized per allele shift (units of s.d.)
norm. beta
0.08
0.03
0.00
−0.03
−0.08
rs4073054
LDL large
LDL small
LDL mean size
IDL total
LDL total
LDL−C assay
APOB assay
HDL total
HDL large
HDL medium
HDL small
HDL mean size
HDL−C by NMR
HDL−C assay
APOA1 assay
VLDL total
VLDL large
VLDL medium
VLDL small
VLDL mean size
TG by NMR
TG assay

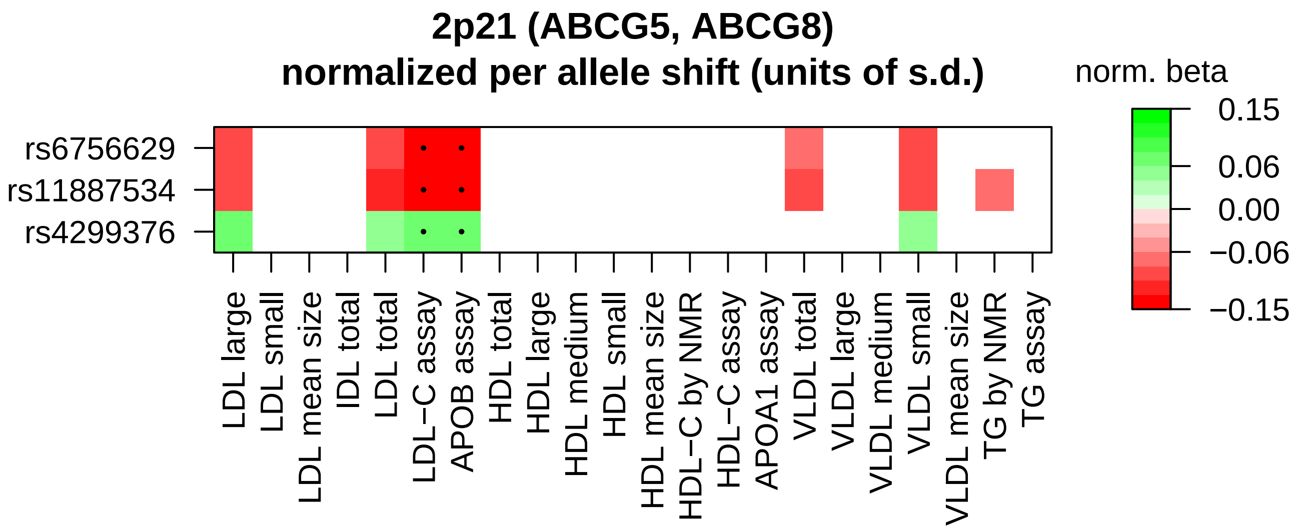
2p21 (ABCG5, ABCG8)
normalized per allele shift (units of s.d.)
norm. beta
0.15
0.06
0.00
−0.06
−0.15
rs6756629
rs11887534
rs4299376
LDL large
LDL small
LDL mean size
IDL total
LDL total
LDL−C assay
APOB assay
HDL total
HDL large
HDL medium
HDL small
HDL mean size
HDL−C by NMR
HDL−C assay
APOA1 assay
VLDL total
VLDL large
VLDL medium
VLDL small
VLDL mean size
TG by NMR
TG assay

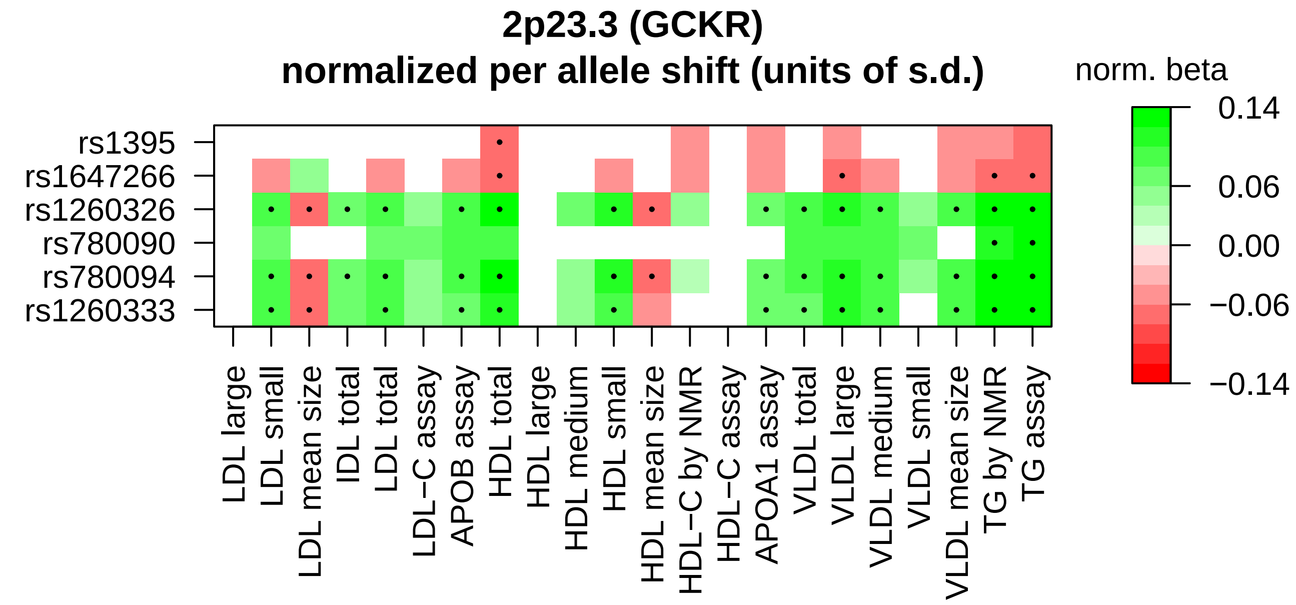
2p23.3 (GCKR)
normalized per allele shift (units of s.d.)
norm. beta
0.14
0.06
0.00
−0.06
−0.14
rs1395
rs1647266
rs1260326
rs780090
rs780094
rs1260333
LDL large
LDL small
LDL mean size
IDL total
LDL total
LDL−C assay
APOB assay
HDL total
HDL large
HDL medium
HDL small
HDL mean size
HDL−C by NMR
HDL−C assay
APOA1 assay
VLDL total
VLDL large
VLDL medium
VLDL small
VLDL mean size
TG by NMR
TG assay

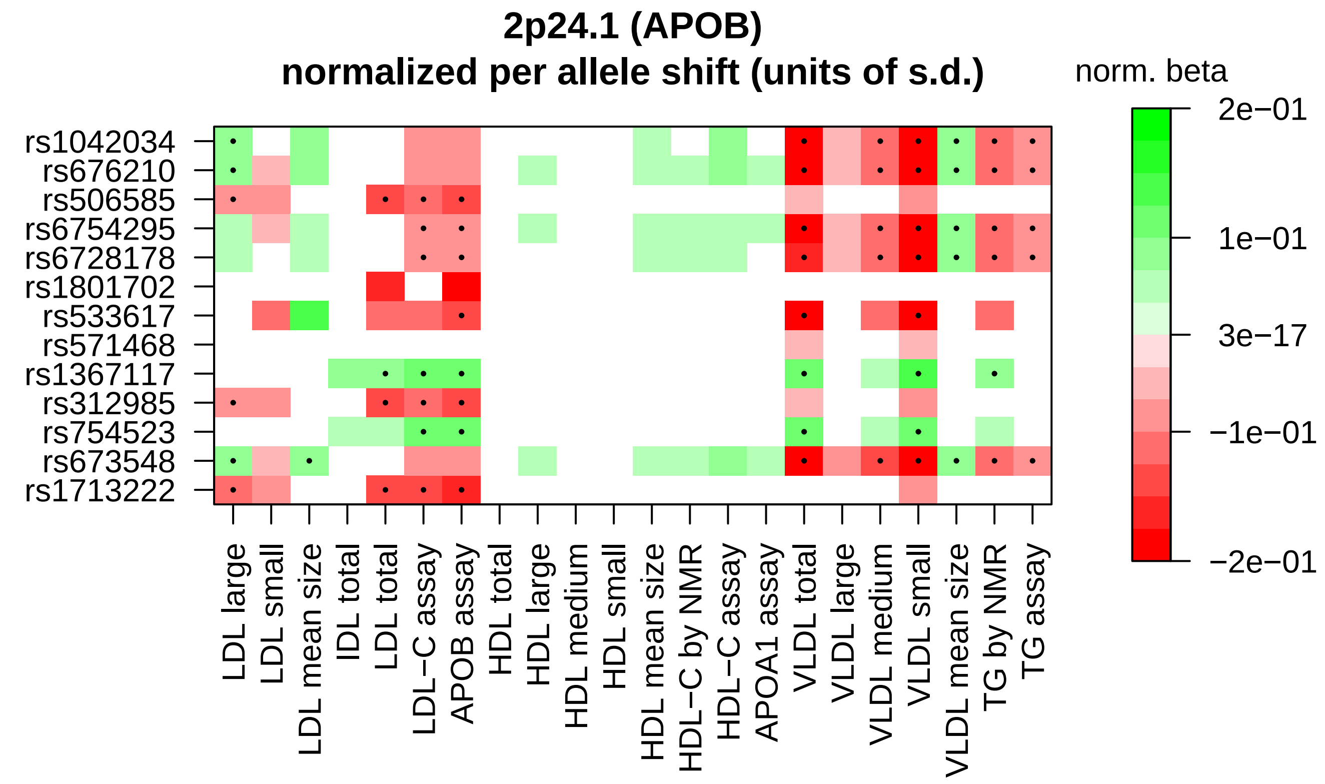

2p24.1 (APOB)
normalized per allele shift (units of s.d.)
norm. beta
2e−01
1e−01
3e−17
−1e−01
−2e−01
rs1042034
rs676210
rs506585
rs6754295
rs6728178
rs1801702
rs533617
rs571468
rs1367117
rs312985
rs754523
rs673548
rs1713222
LDL large
LDL small
LDL mean size
IDL total
LDL total
LDL−C assay
APOB assay
HDL total
HDL large
HDL medium
HDL small
HDL mean size
HDL−C by NMR
HDL−C assay
APOA1 assay
VLDL total
VLDL large
VLDL medium
VLDL small
VLDL mean size
TG by NMR
TG assay

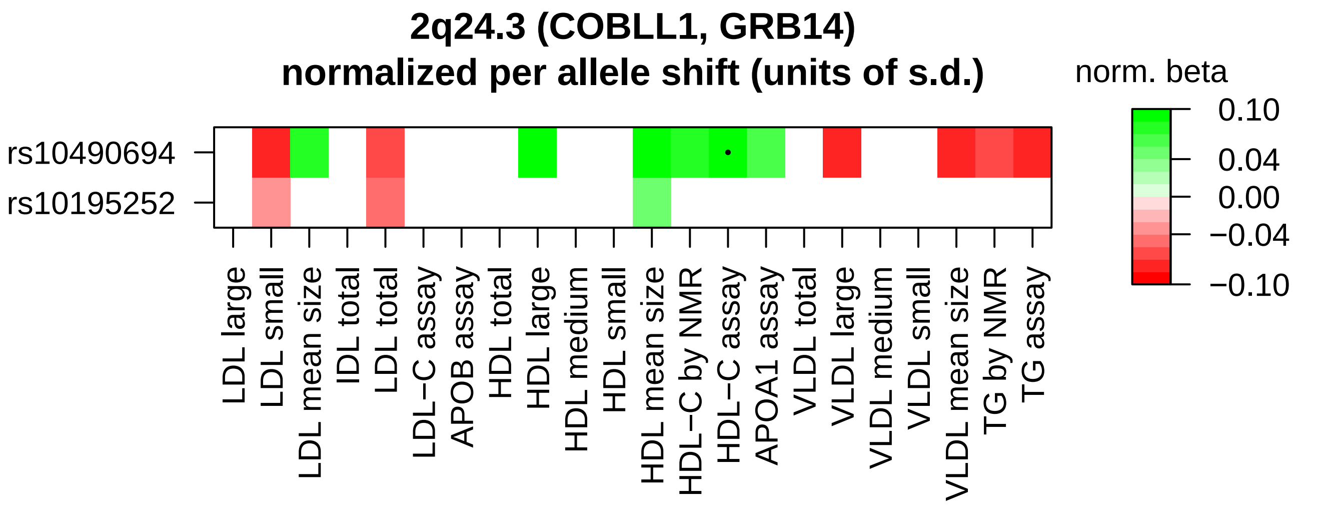

2q24.3 (COBLL1, GRB14)
normalized per allele shift (units of s.d.)
norm. beta
0.10
0.04
0.00
−0.04
−0.10
rs10490694
rs10195252
LDL large
LDL small
LDL mean size
IDL total
LDL total
LDL−C assay
APOB assay
HDL total
HDL large
HDL medium
HDL small
HDL mean size
HDL−C by NMR
HDL−C assay
APOA1 assay
VLDL total
VLDL large
VLDL medium
VLDL small
VLDL mean size
TG by NMR
TG assay

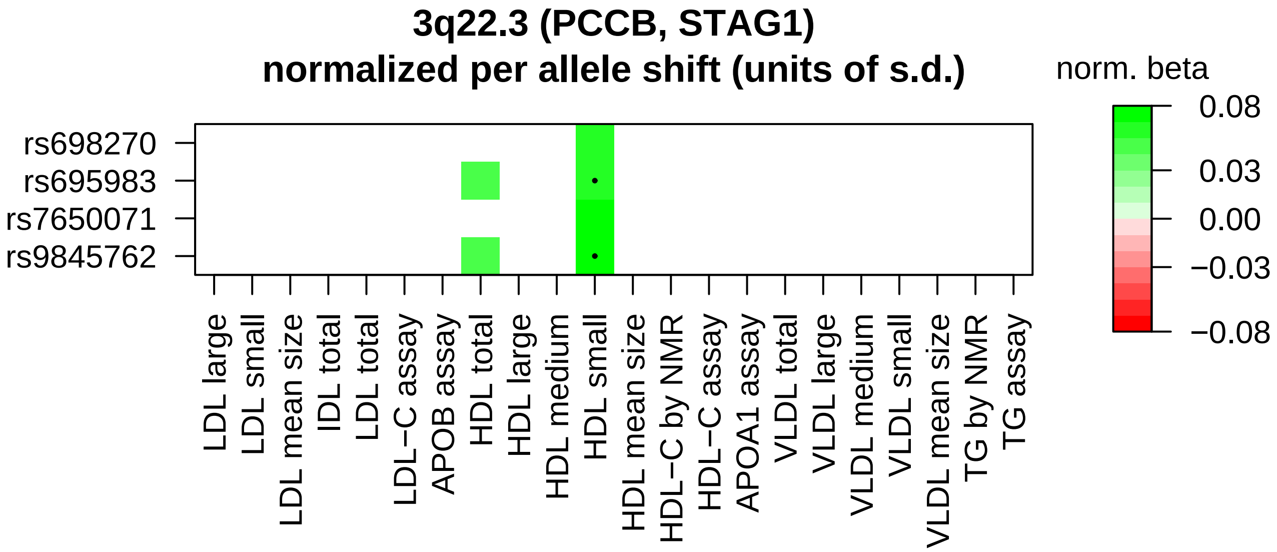
3q22.3 (PCCB, STAG1)
normalized per allele shift (units of s.d.)
norm. beta
0.08
0.03
0.00
−0.03
−0.08
rs698270
rs695983
rs7650071
rs9845762
LDL large
LDL small
LDL mean size
IDL total
LDL total
LDL−C assay
APOB assay
HDL total
HDL large
HDL medium
HDL small
HDL mean size
HDL−C by NMR
HDL−C assay
APOA1 assay
VLDL total
VLDL large
VLDL medium
VLDL small
VLDL mean size
TG by NMR
TG assay

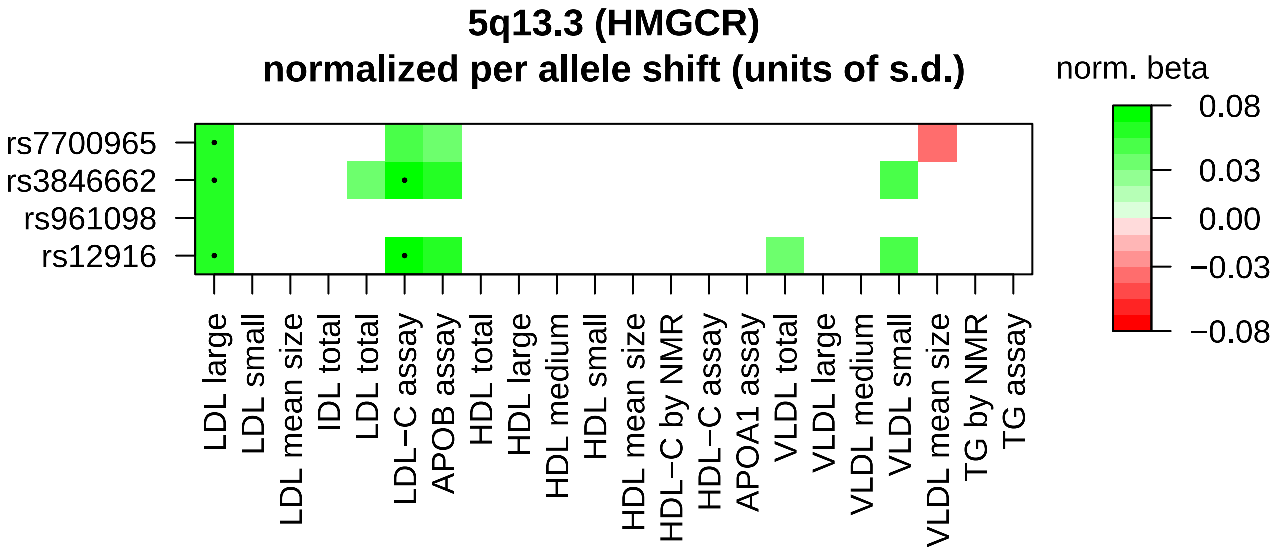
5q13.3 (HMGCR)
normalized per allele shift (units of s.d.)
norm. beta
0.08
0.03
0.00
−0.03
−0.08
rs7700965
rs3846662
rs961098
rs12916
LDL large
LDL small
LDL mean size
IDL total
LDL total
LDL−C assay
APOB assay
HDL total
HDL large
HDL medium
HDL small
HDL mean size
HDL−C by NMR
HDL−C assay
APOA1 assay
VLDL total
VLDL large
VLDL medium
VLDL small
VLDL mean size
TG by NMR
TG assay

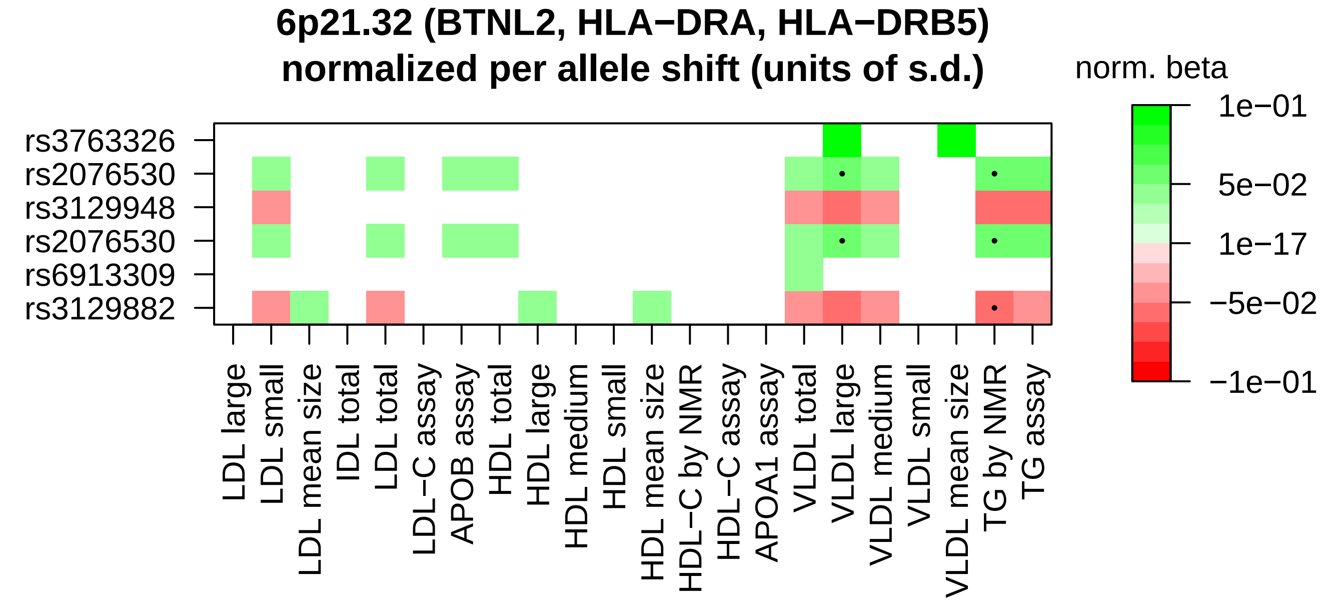
6p21.32 (BTNL2, HLA−DRA, HLA−DRB5)
normalized per allele shift (units of s.d.)
norm. beta
1e−01
5e−02
1e−17
−5e−02
−1e−01
rs3763326
rs2076530
rs3129948
rs2076530
rs6913309
rs3129882
LDL large
LDL small
LDL mean size
IDL total
LDL total
LDL−C assay
APOB assay
HDL total
HDL large
HDL medium
HDL small
HDL mean size
HDL−C by NMR
HDL−C assay
APOA1 assay
VLDL total
VLDL large
VLDL medium
VLDL small
VLDL mean size
TG by NMR
TG assay

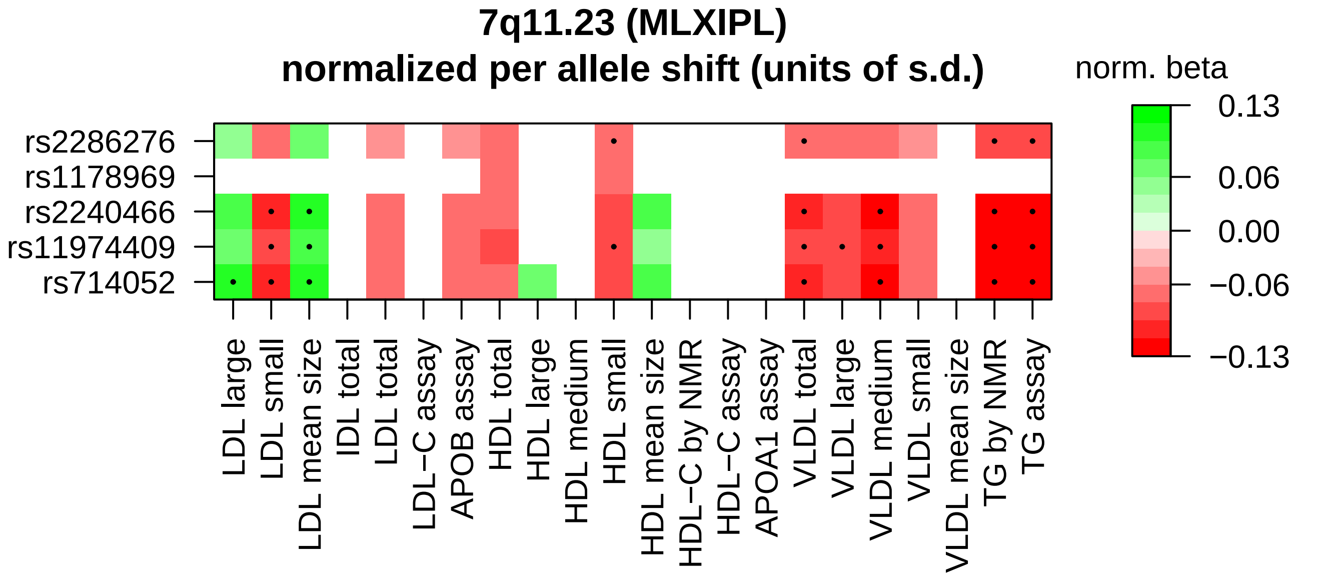
7q11.23 (MLXIPL)
normalized per allele shift (units of s.d.)
norm. beta
0.13
0.06
0.00
−0.06
−0.13
rs2286276
rs1178969
rs2240466
rs11974409
rs714052
LDL large
LDL small
LDL mean size
IDL total
LDL total
LDL−C assay
APOB assay
HDL total
HDL large
HDL medium
HDL small
HDL mean size
HDL−C by NMR
HDL−C assay
APOA1 assay
VLDL total
VLDL large
VLDL medium
VLDL small
VLDL mean size
TG by NMR
TG assay

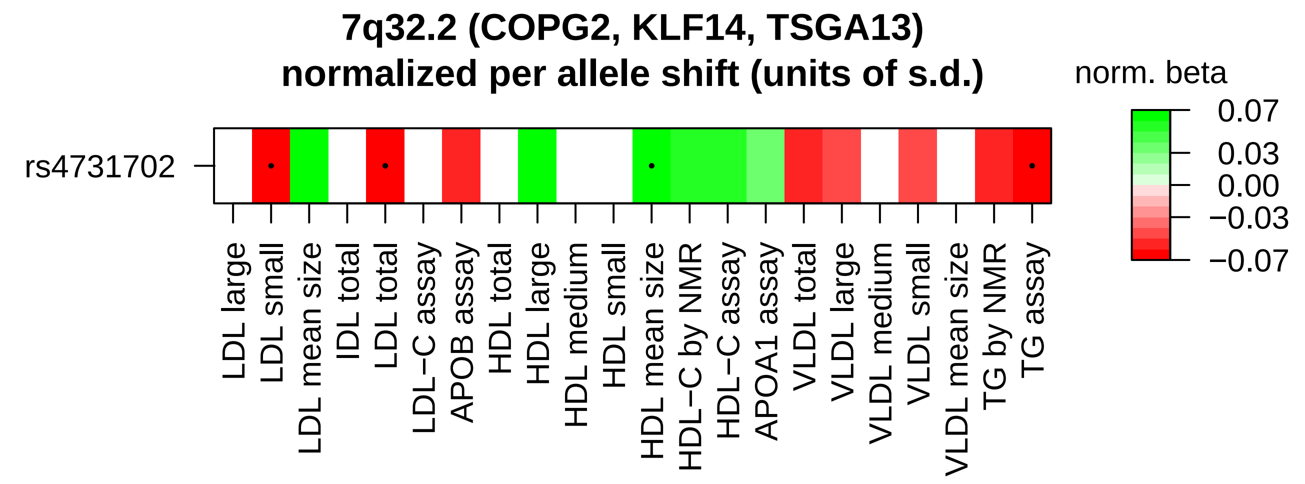

7q32.2 (COPG2, KLF14, TSGA13)
normalized per allele shift (units of s.d.)
norm. beta
0.07
0.03
0.00
−0.03
−0.07
rs4731702
LDL large
LDL small
LDL mean size
IDL total
LDL total
LDL−C assay
APOB assay
HDL total
HDL large
HDL medium
HDL small
HDL mean size
HDL−C by NMR
HDL−C assay
APOA1 assay
VLDL total
VLDL large
VLDL medium
VLDL small
VLDL mean size
TG by NMR
TG assay

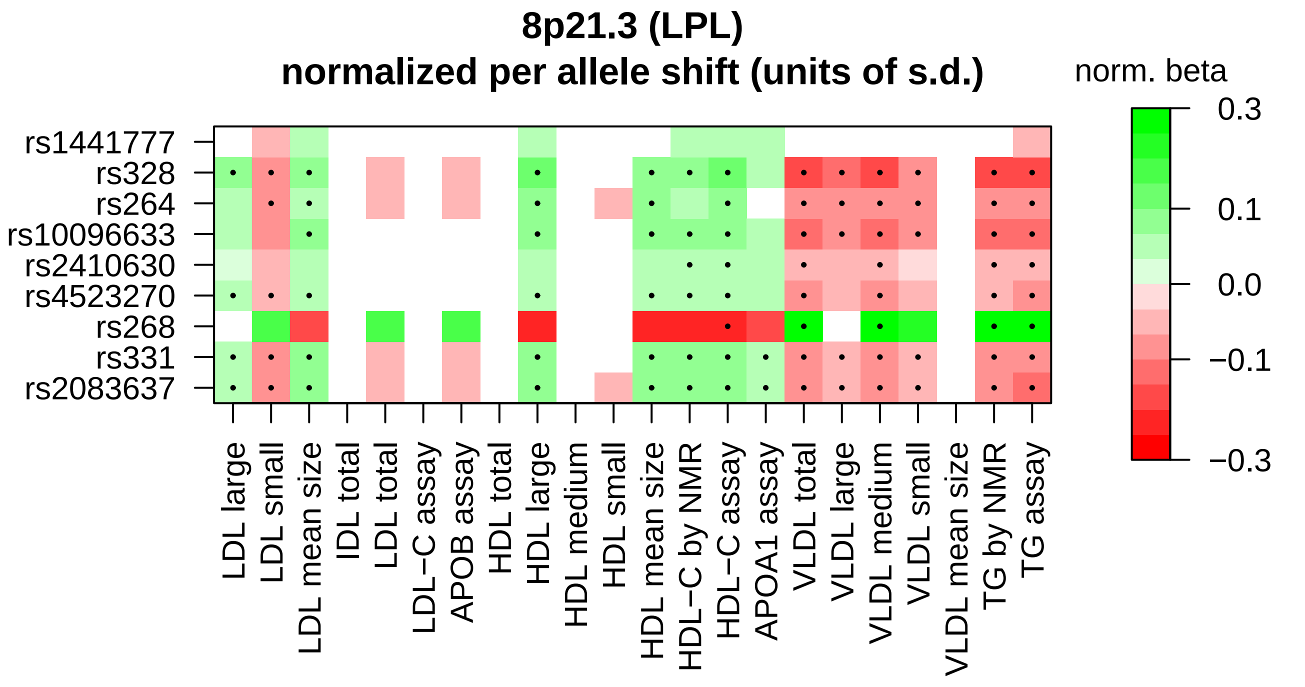

8p21.3 (LPL)
normalized per allele shift (units of s.d.)
norm. beta
0.3
0.1
0.0
−0.1
−0.3
rs1441777
rs328
rs264
rs10096633
rs2410630
rs4523270
rs268
rs331
rs2083637
LDL large
LDL small
LDL mean size
IDL total
LDL total
LDL−C assay
APOB assay
HDL total
HDL large
HDL medium
HDL small
HDL mean size
HDL−C by NMR
HDL−C assay
APOA1 assay
VLDL total
VLDL large
VLDL medium
VLDL small
VLDL mean size
TG by NMR
TG assay

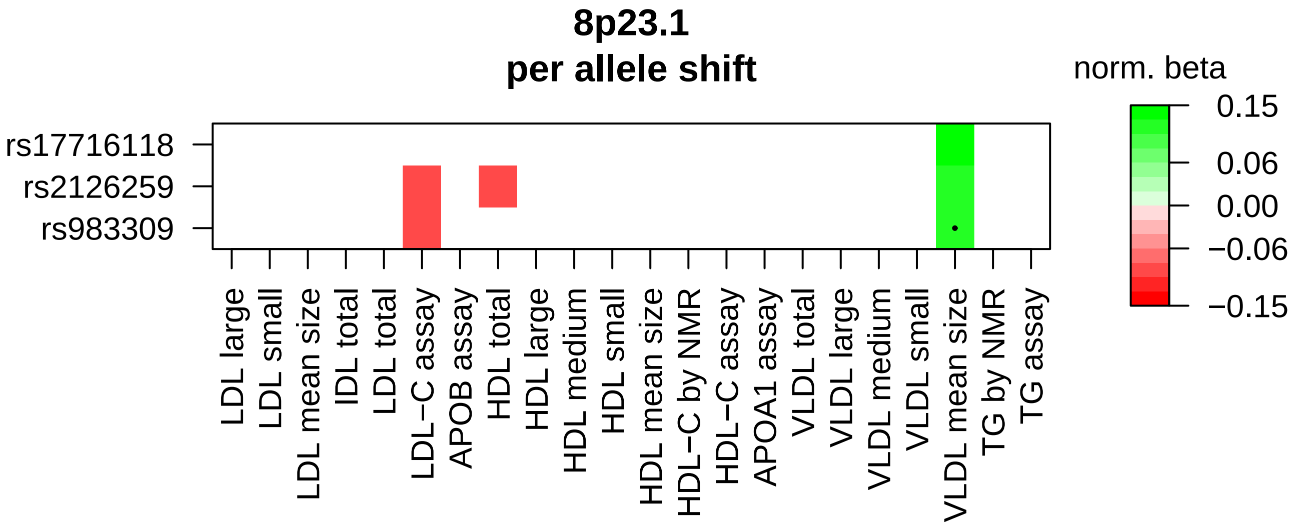
8p23.1
per allele shift
norm. beta
0.15
0.06
0.00
−0.06
−0.15
rs17716118
rs2126259
rs983309
LDL large
LDL small
LDL mean size
IDL total
LDL total
LDL−C assay
APOB assay
HDL total
HDL large
HDL medium
HDL small
HDL mean size
HDL−C by NMR
HDL−C assay
APOA1 assay
VLDL total
VLDL large
VLDL medium
VLDL small
VLDL mean size
TG by NMR
TG assay

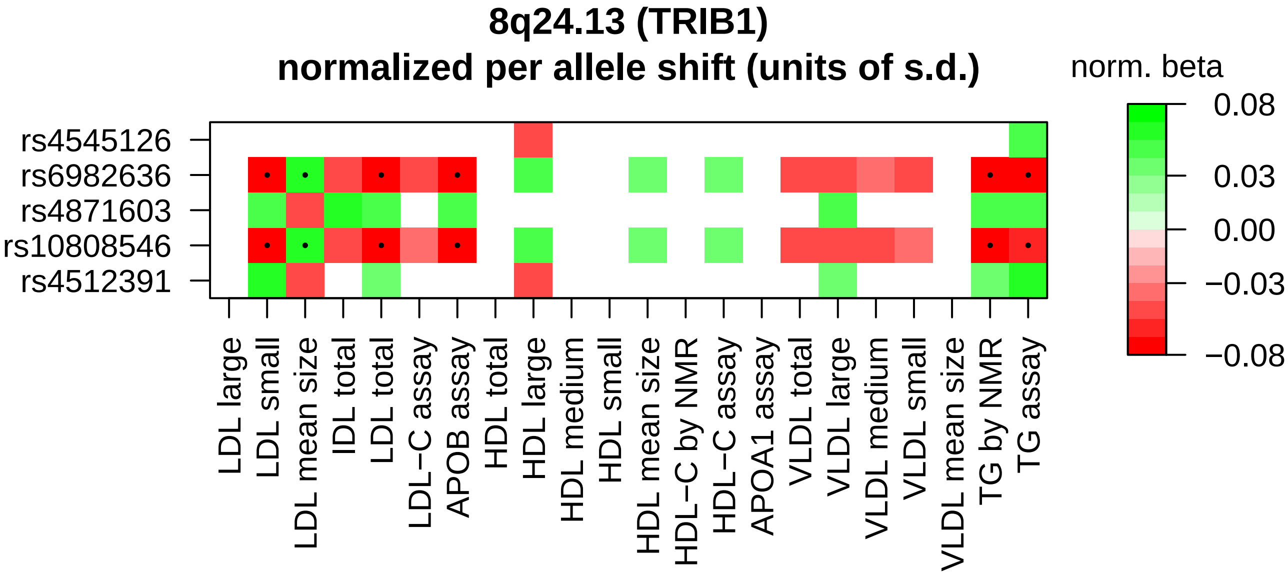
8q24.13 (TRIB1)
normalized per allele shift (units of s.d.)
norm. beta
0.08
0.03
0.00
−0.03
−0.08
rs4545126
rs6982636
rs4871603
rs10808546
rs4512391
LDL large
LDL small
LDL mean size
IDL total
LDL total
LDL−C assay
APOB assay
HDL total
HDL large
HDL medium
HDL small
HDL mean size
HDL−C by NMR
HDL−C assay
APOA1 assay
VLDL total
VLDL large
VLDL medium
VLDL small
VLDL mean size
TG by NMR
TG assay

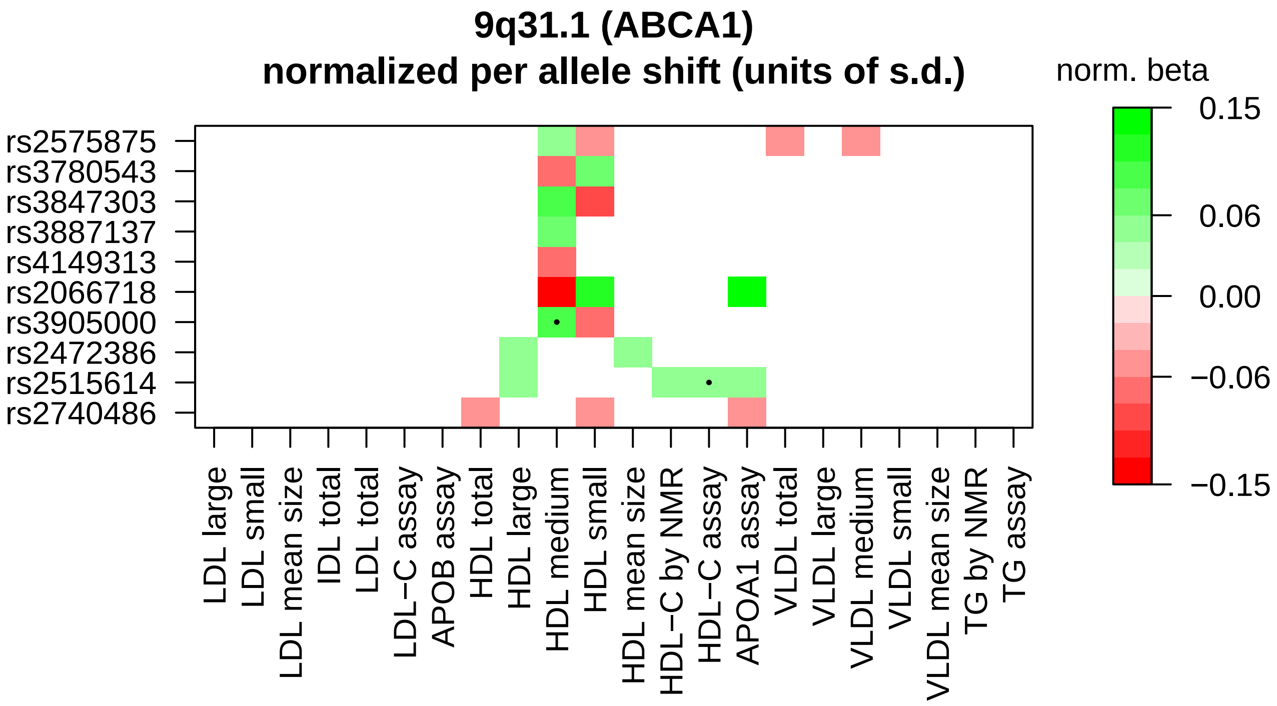
9q31.1 (ABCA1)
normalized per allele shift (units of s.d.)
norm. beta
0.15
0.06
0.00
−0.06
−0.15
rs2575875
rs3780543
rs3847303
rs3887137
rs4149313
rs2066718
rs3905000
rs2472386
rs2515614
rs2740486
LDL large
LDL small
LDL mean size
IDL total
LDL total
LDL−C assay
APOB assay
HDL total
HDL large
HDL medium
HDL small
HDL mean size
HDL−C by NMR
HDL−C assay
APOA1 assay
VLDL total
VLDL large
VLDL medium
VLDL small
VLDL mean size
TG by NMR
TG assay

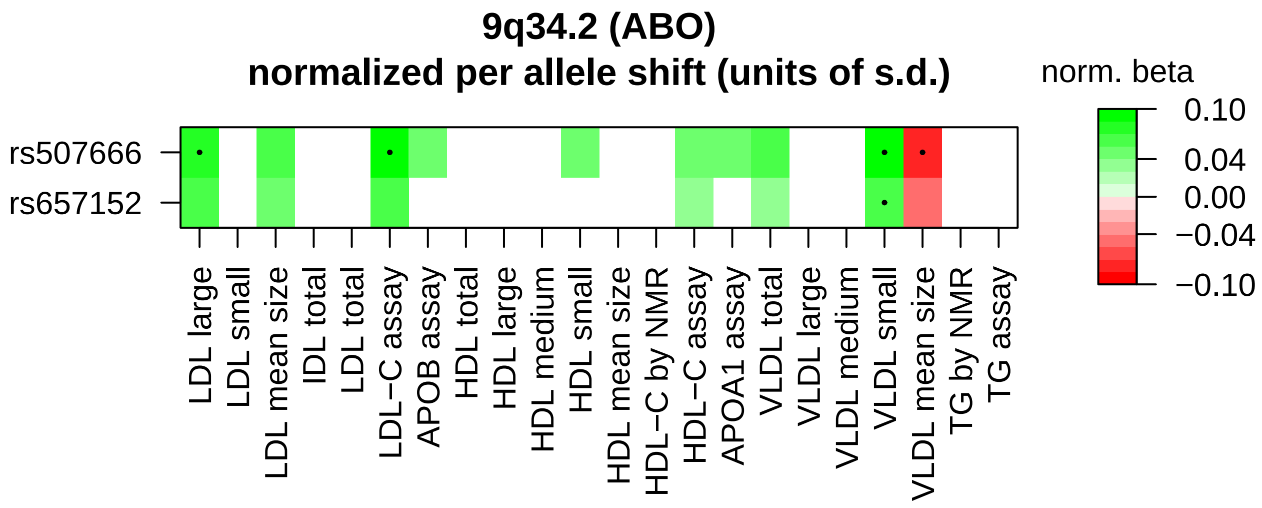
9q34.2 (ABO)
normalized per allele shift (units of s.d.)
norm. beta
0.10
0.04
0.00
−0.04
−0.10
rs507666
rs657152
LDL large
LDL small
LDL mean size
IDL total
LDL total
LDL−C assay
APOB assay
HDL total
HDL large
HDL medium
HDL small
HDL mean size
HDL−C by NMR
HDL−C assay
APOA1 assay
VLDL total
VLDL large
VLDL medium
VLDL small
VLDL mean size
TG by NMR
TG assay

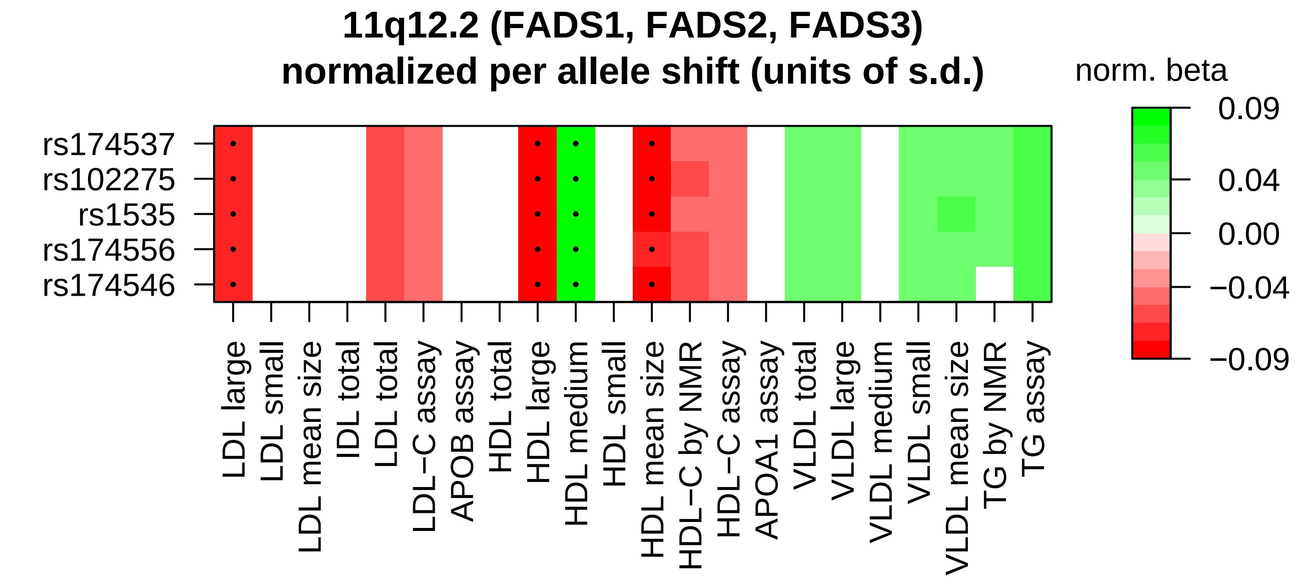

11q12.2 (FADS1, FADS2, FADS3)
normalized per allele shift (units of s.d.)
norm. beta
0.09
0.04
0.00
−0.04
−0.09
rs174537
rs102275
rs1535
rs174556
rs174546
LDL large
LDL small
LDL mean size
IDL total
LDL total
LDL−C assay
APOB assay
HDL total
HDL large
HDL medium
HDL small
HDL mean size
HDL−C by NMR
HDL−C assay
APOA1 assay
VLDL total
VLDL large
VLDL medium
VLDL small
VLDL mean size
TG by NMR
TG assay

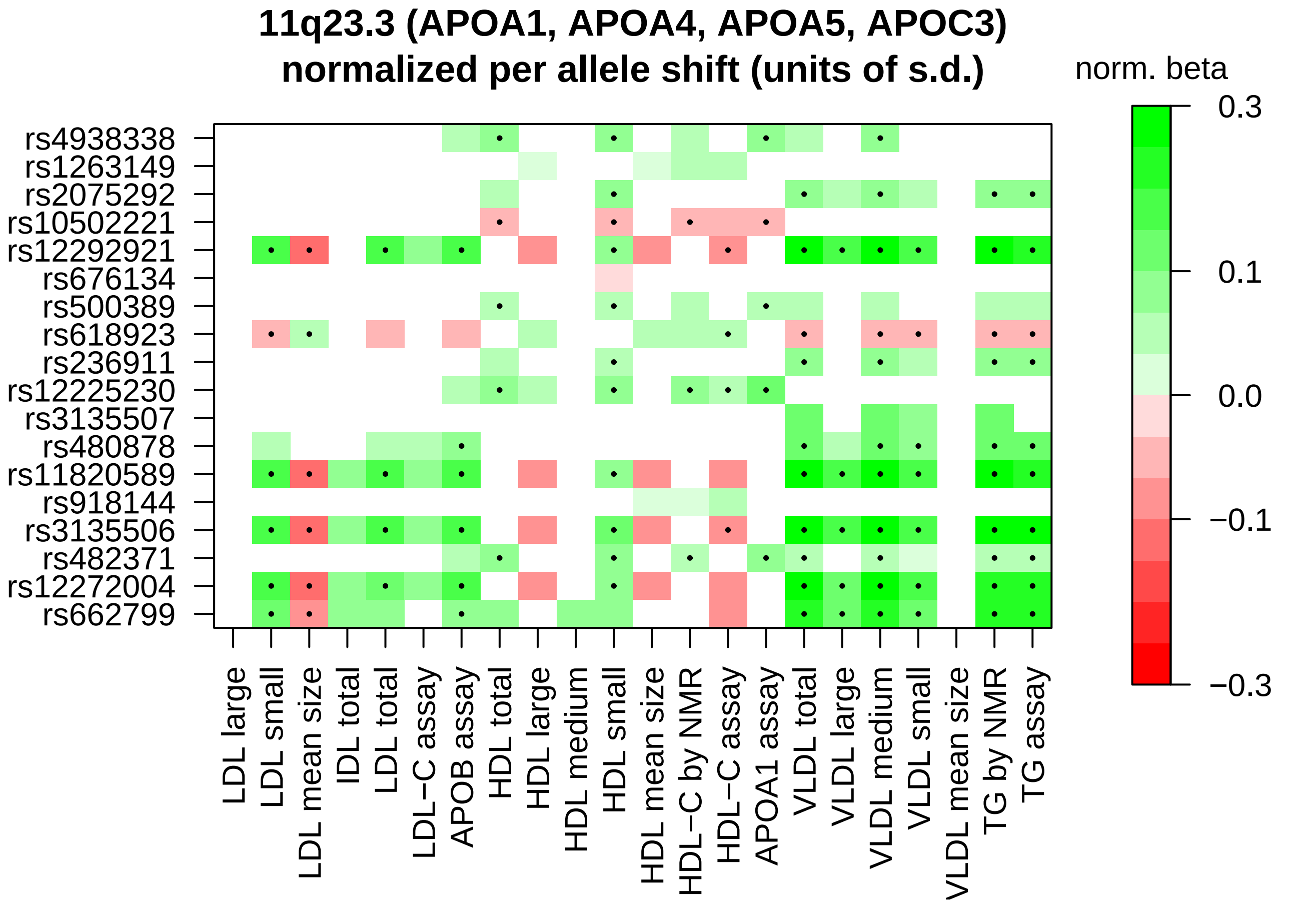

11q23.3 (APOA1, APOA4, APOA5, APOC3)
normalized per allele shift (units of s.d.)
norm. beta
0.3
0.1
0.0
−0.1
−0.3
rs4938338
rs1263149
rs2075292
rs10502221
rs12292921
rs676134
rs500389
rs618923
rs236911
rs12225230
rs3135507
rs480878
rs11820589
rs918144
rs3135506
rs482371
rs12272004
rs662799
LDL large
LDL small
LDL mean size
IDL total
LDL total
LDL−C assay
APOB assay
HDL total
HDL large
HDL medium
HDL small
HDL mean size
HDL−C by NMR
HDL−C assay
APOA1 assay
VLDL total
VLDL large
VLDL medium
VLDL small
VLDL mean size
TG by NMR
TG assay

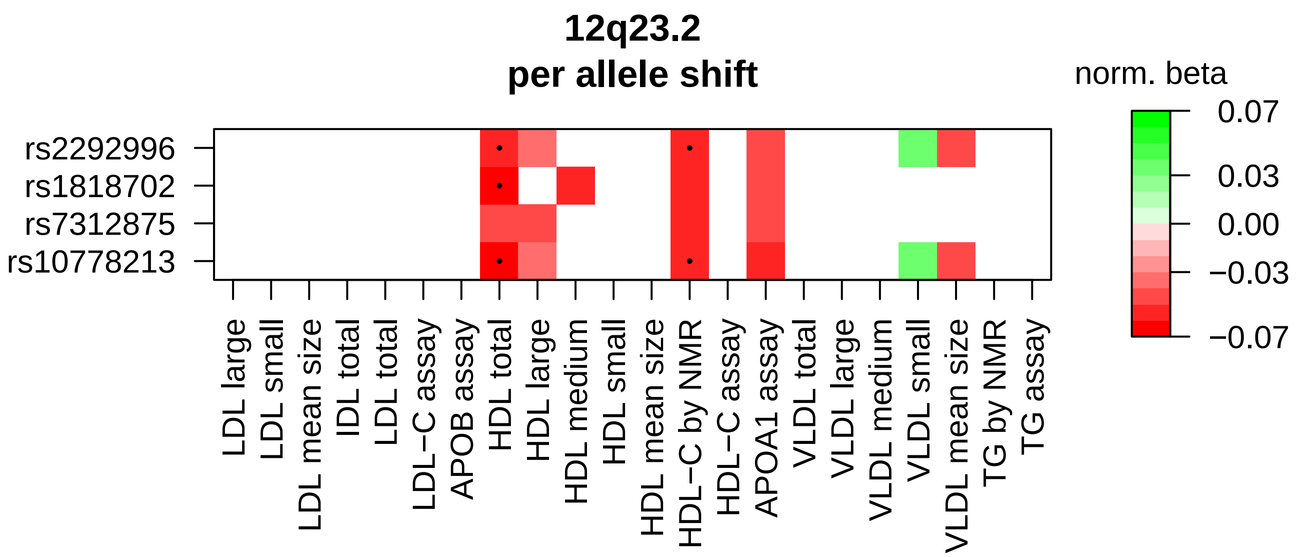
12q23.2
per allele shift
norm. beta
0.07
0.03
0.00
−0.03
−0.07
rs2292996
rs1818702
rs7312875
rs10778213
LDL large
LDL small
LDL mean size
IDL total
LDL total
LDL−C assay
APOB assay
HDL total
HDL large
HDL medium
HDL small
HDL mean size
HDL−C by NMR
HDL−C assay
APOA1 assay
VLDL total
VLDL large
VLDL medium
VLDL small
VLDL mean size
TG by NMR
TG assay

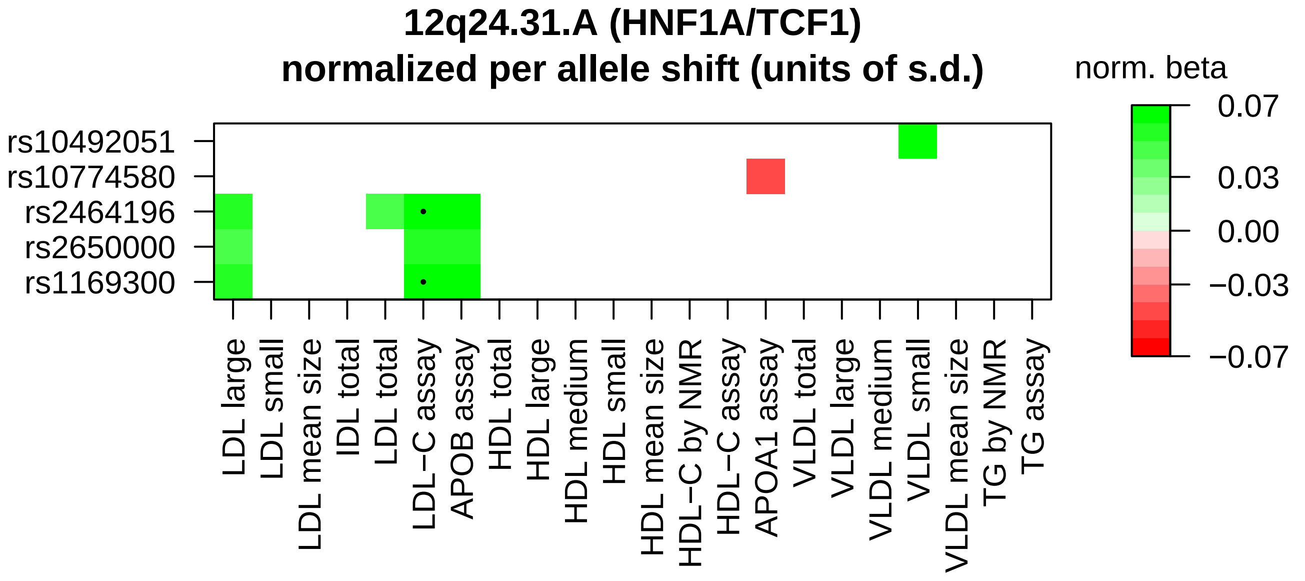
12q24.31.A (HNF1A/TCF1)
normalized per allele shift (units of s.d.)
norm. beta
0.07
0.03
0.00
−0.03
−0.07
rs10492051
rs10774580
rs2464196
rs2650000
rs1169300
LDL large
LDL small
LDL mean size
IDL total
LDL total
LDL−C assay
APOB assay
HDL total
HDL large
HDL medium
HDL small
HDL mean size
HDL−C by NMR
HDL−C assay
APOA1 assay
VLDL total
VLDL large
VLDL medium
VLDL small
VLDL mean size
TG by NMR
TG assay

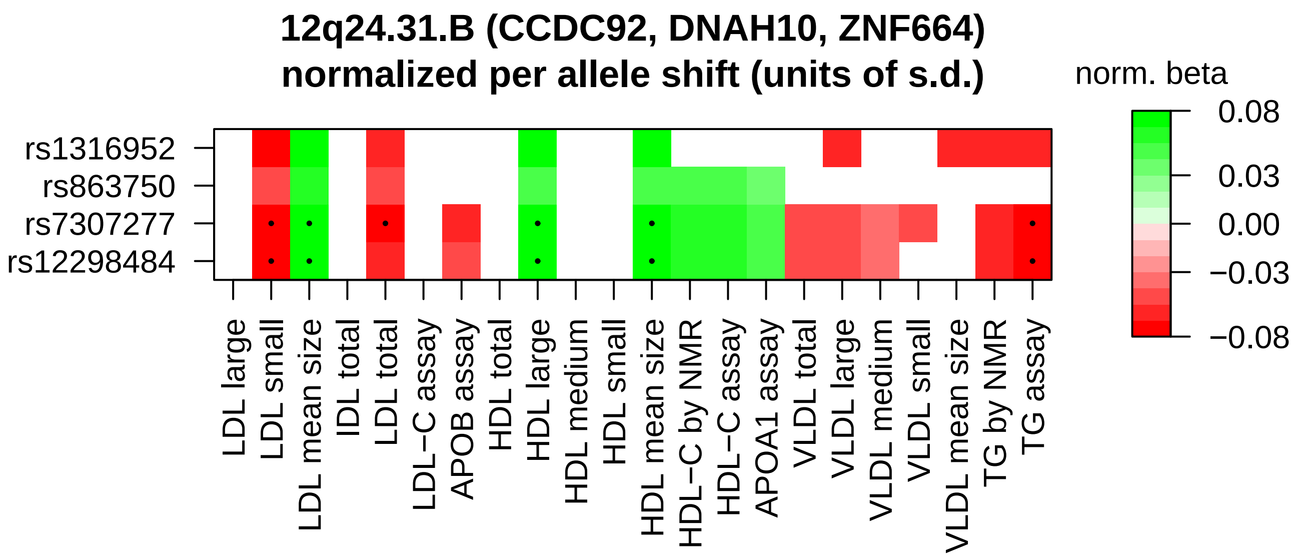

12q24.31.B (CCDC92, DNAH10, ZNF664)
normalized per allele shift (units of s.d.)
norm. beta
0.08
0.03
0.00
−0.03
−0.08
rs1316952
rs863750
rs7307277
rs12298484
LDL large
LDL small
LDL mean size
IDL total
LDL total
LDL−C assay
APOB assay
HDL total
HDL large
HDL medium
HDL small
HDL mean size
HDL−C by NMR
HDL−C assay
APOA1 assay
VLDL total
VLDL large
VLDL medium
VLDL small
VLDL mean size
TG by NMR
TG assay

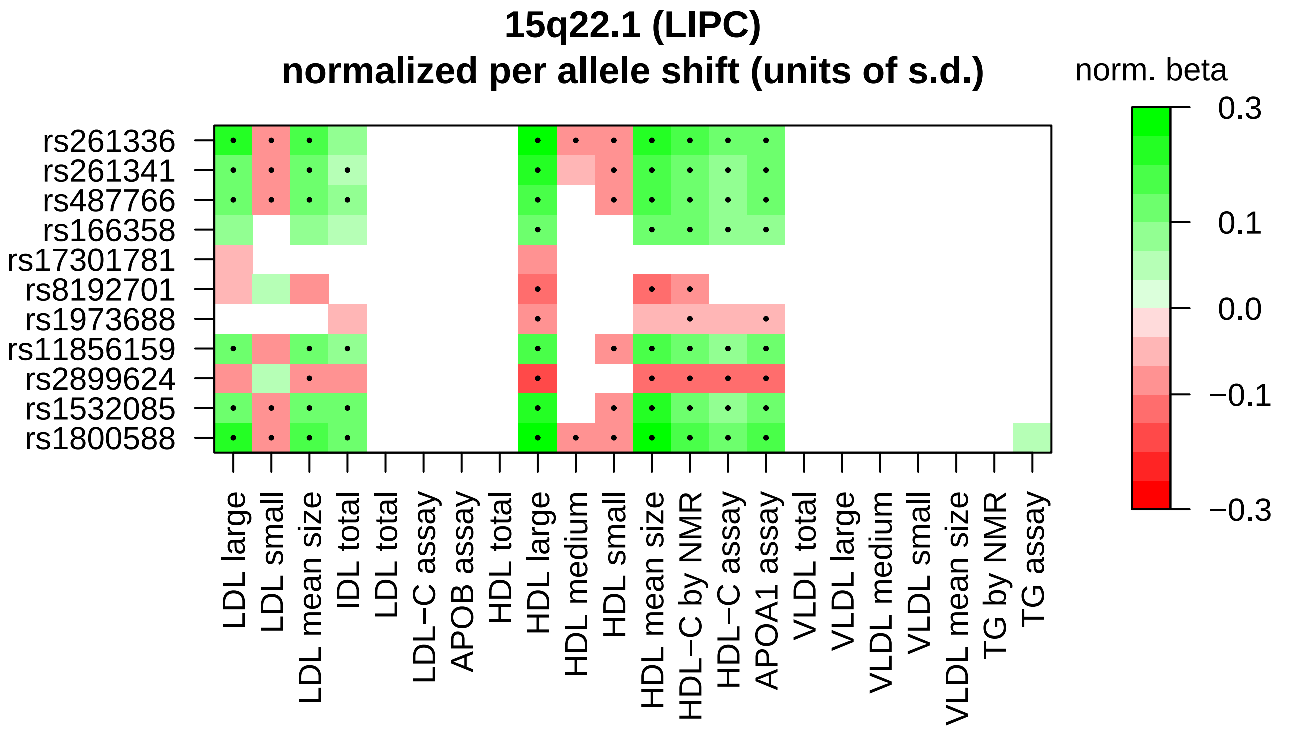

15q22.1 (LIPC)
normalized per allele shift (units of s.d.)
norm. beta
0.3
0.1
0.0
−0.1
−0.3
rs261336
rs261341
rs487766
rs166358
rs17301781
rs8192701
rs1973688
rs11856159
rs2899624
rs1532085
rs1800588
LDL large
LDL small
LDL mean size
IDL total
LDL total
LDL−C assay
APOB assay
HDL total
HDL large
HDL medium
HDL small
HDL mean size
HDL−C by NMR
HDL−C assay
APOA1 assay
VLDL total
VLDL large
VLDL medium
VLDL small
VLDL mean size
TG by NMR
TG assay

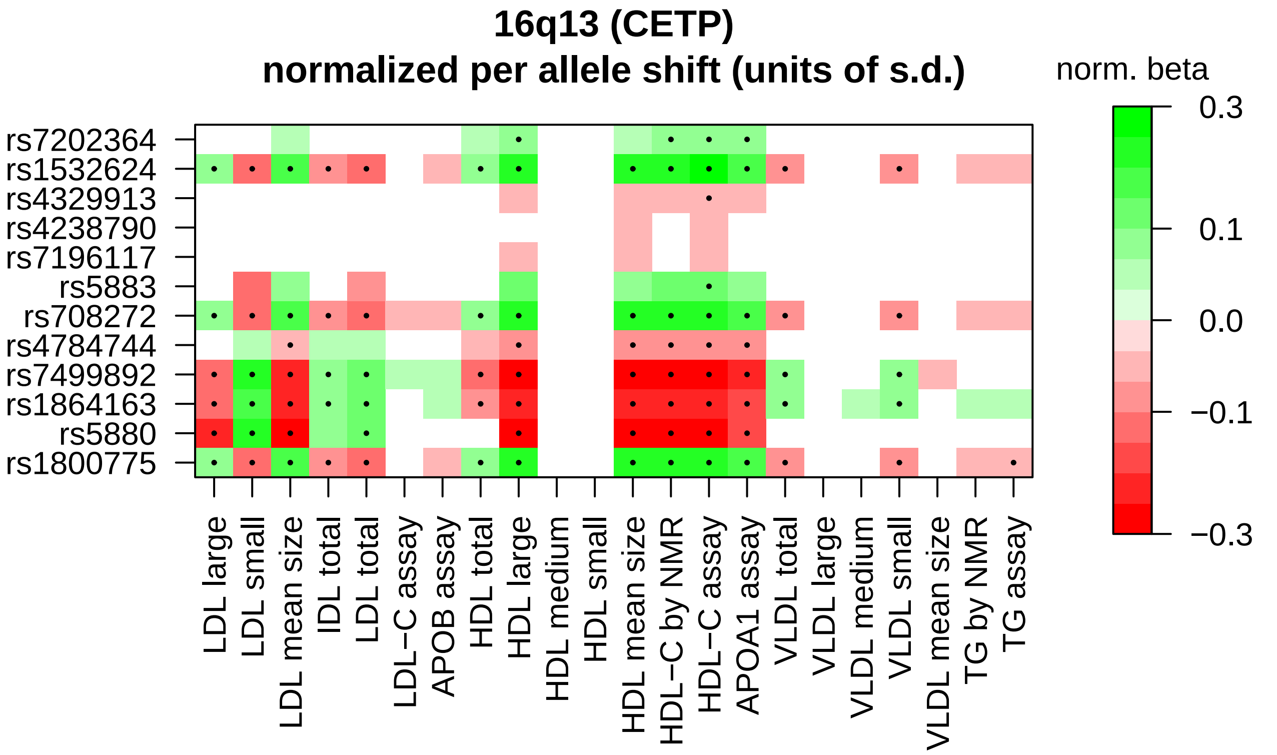
16q13 (CETP)
normalized per allele shift (units of s.d.)
norm. beta
0.3
0.1
0.0
−0.1
−0.3
rs7202364
rs1532624
rs4329913
rs4238790
rs7196117
rs5883
rs708272
rs4784744
rs7499892
rs1864163
rs5880
rs1800775
LDL large
LDL small
LDL mean size
IDL total
LDL total
LDL−C assay
APOB assay
HDL total
HDL large
HDL medium
HDL small
HDL mean size
HDL−C by NMR
HDL−C assay
APOA1 assay
VLDL total
VLDL large
VLDL medium
VLDL small
VLDL mean size
TG by NMR
TG assay

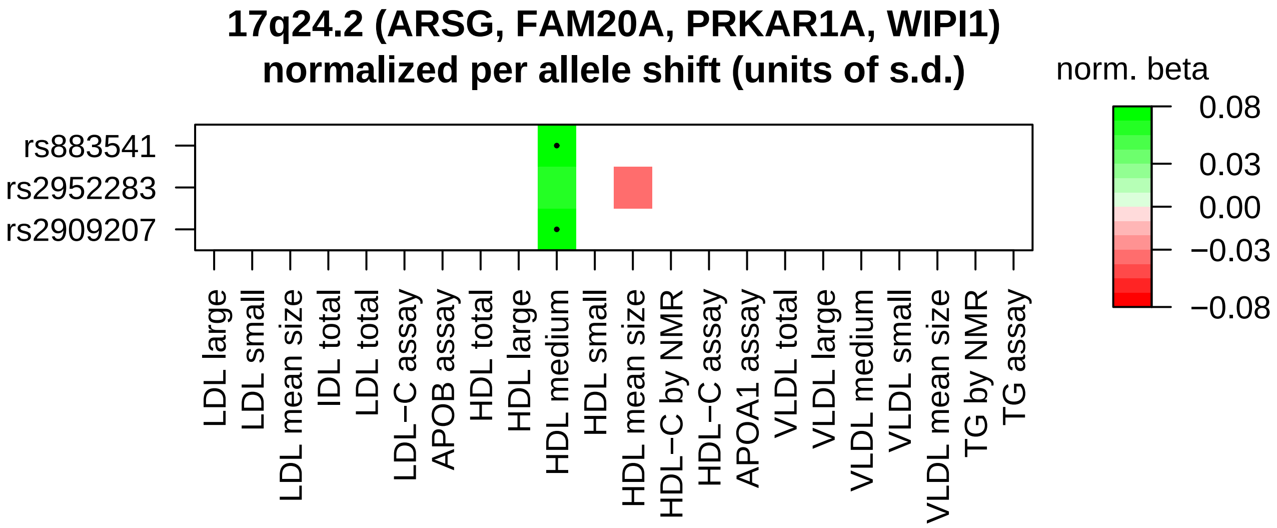
17q24.2 (ARSG, FAM20A, PRKAR1A, WIPI1)
normalized per allele shift (units of s.d.)
norm. beta
0.08
0.03
0.00
−0.03
−0.08
rs883541
rs2952283
rs2909207
LDL large
LDL small
LDL mean size
IDL total
LDL total
LDL−C assay
APOB assay
HDL total
HDL large
HDL medium
HDL small
HDL mean size
HDL−C by NMR
HDL−C assay
APOA1 assay
VLDL total
VLDL large
VLDL medium
VLDL small
VLDL mean size
TG by NMR
TG assay

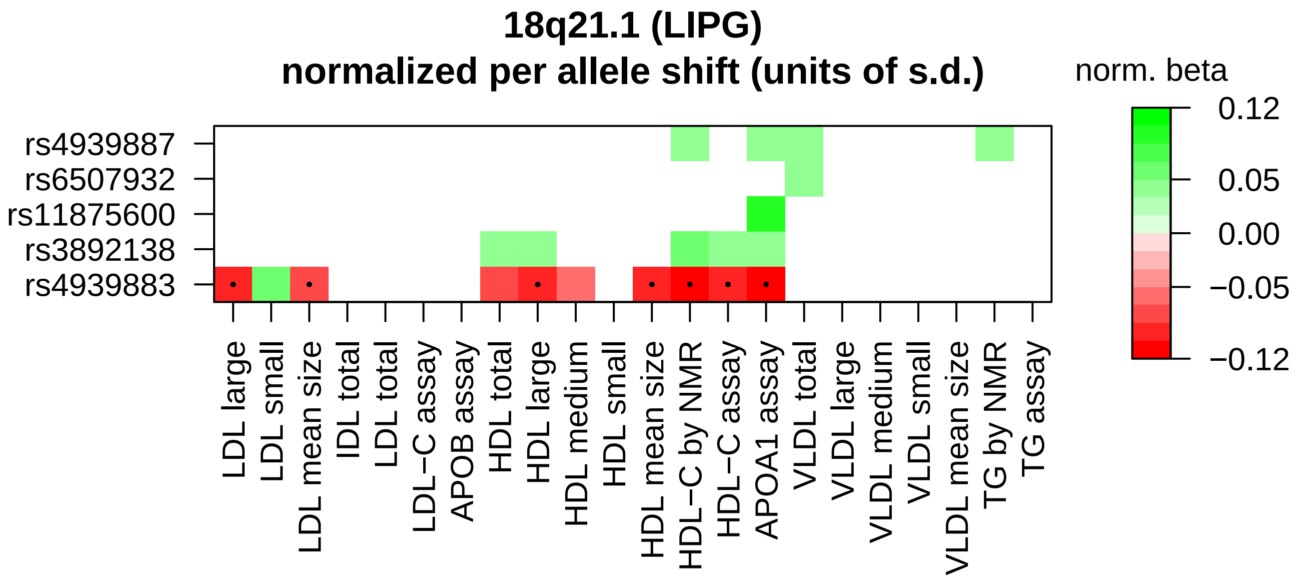
18q21.1 (LIPG)
normalized per allele shift (units of s.d.)
norm. beta
0.12
0.05
0.00
−0.05
−0.12
rs4939887
rs6507932
rs11875600
rs3892138
rs4939883
LDL large
LDL small
LDL mean size
IDL total
LDL total
LDL−C assay
APOB assay
HDL total
HDL large
HDL medium
HDL small
HDL mean size
HDL−C by NMR
HDL−C assay
APOA1 assay
VLDL total
VLDL large
VLDL medium
VLDL small
VLDL mean size
TG by NMR
TG assay

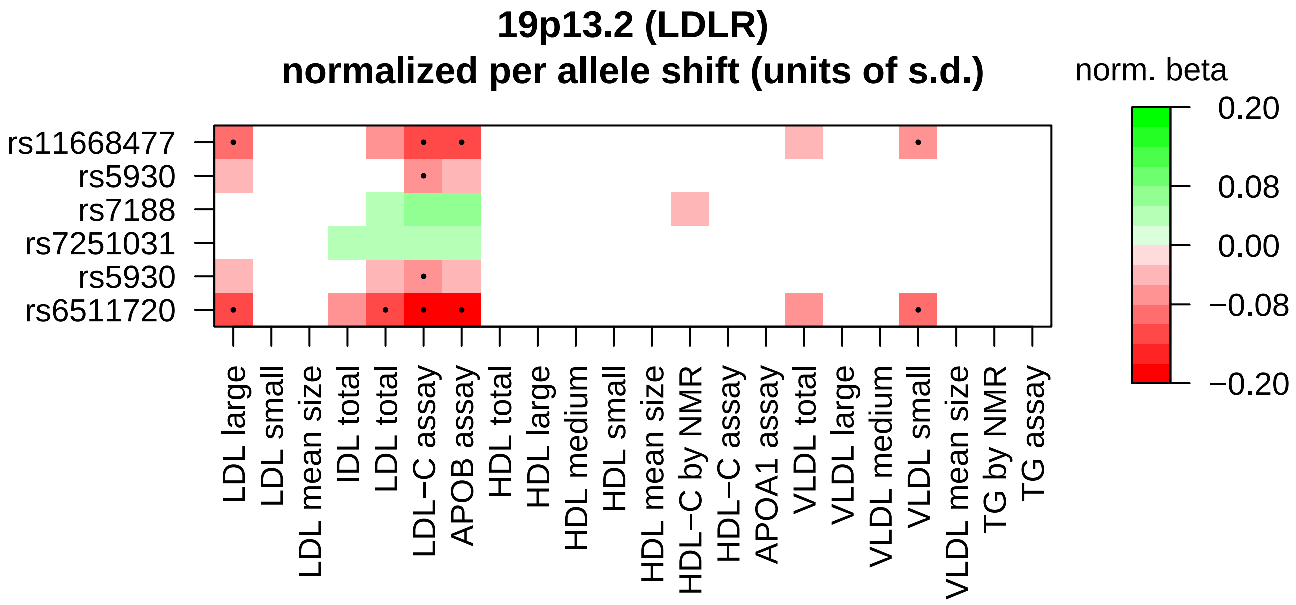
19p13.2 (LDLR)
normalized per allele shift (units of s.d.)
norm. beta
0.20
0.08
0.00
−0.08
−0.20
rs11668477
rs5930
rs7188
rs7251031
rs5930
rs6511720
LDL large
LDL small
LDL mean size
IDL total
LDL total
LDL−C assay
APOB assay
HDL total
HDL large
HDL medium
HDL small
HDL mean size
HDL−C by NMR
HDL−C assay
APOA1 assay
VLDL total
VLDL large
VLDL medium
VLDL small
VLDL mean size
TG by NMR
TG assay

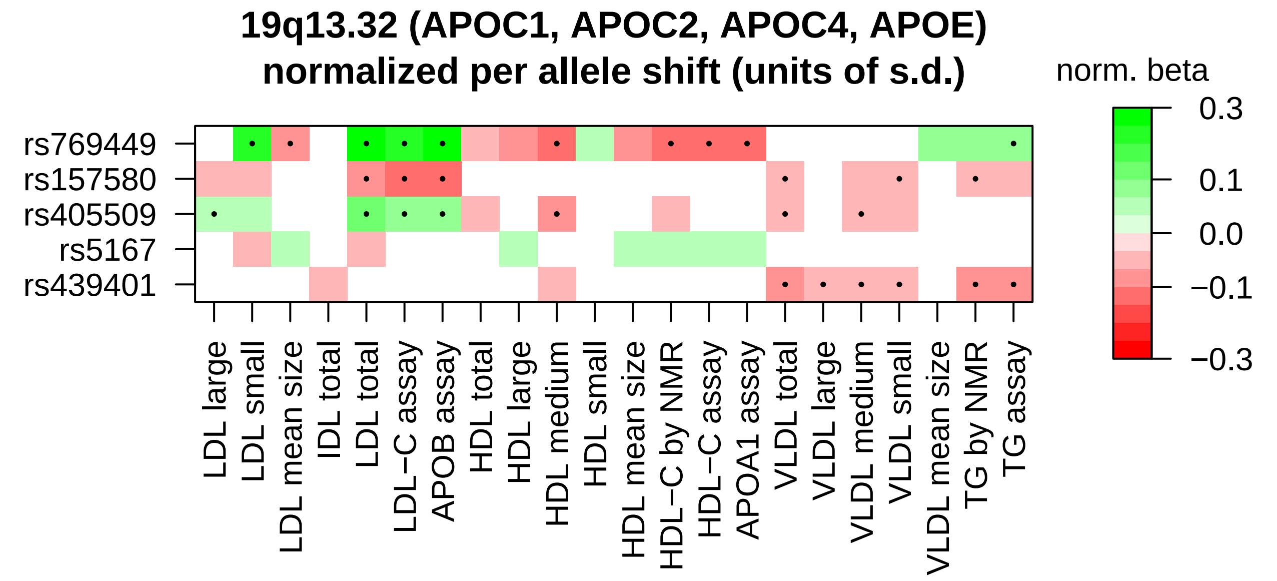

19q13.32 (APOC1, APOC2, APOC4, APOE)
normalized per allele shift (units of s.d.)
norm. beta
0.3
0.1
0.0
−0.1
−0.3
rs769449
rs157580
rs405509
rs5167
rs439401
LDL large
LDL small
LDL mean size
IDL total
LDL total
LDL−C assay
APOB assay
HDL total
HDL large
HDL medium
HDL small
HDL mean size
HDL−C by NMR
HDL−C assay
APOA1 assay
VLDL total
VLDL large
VLDL medium
VLDL small
VLDL mean size
TG by NMR
TG assay

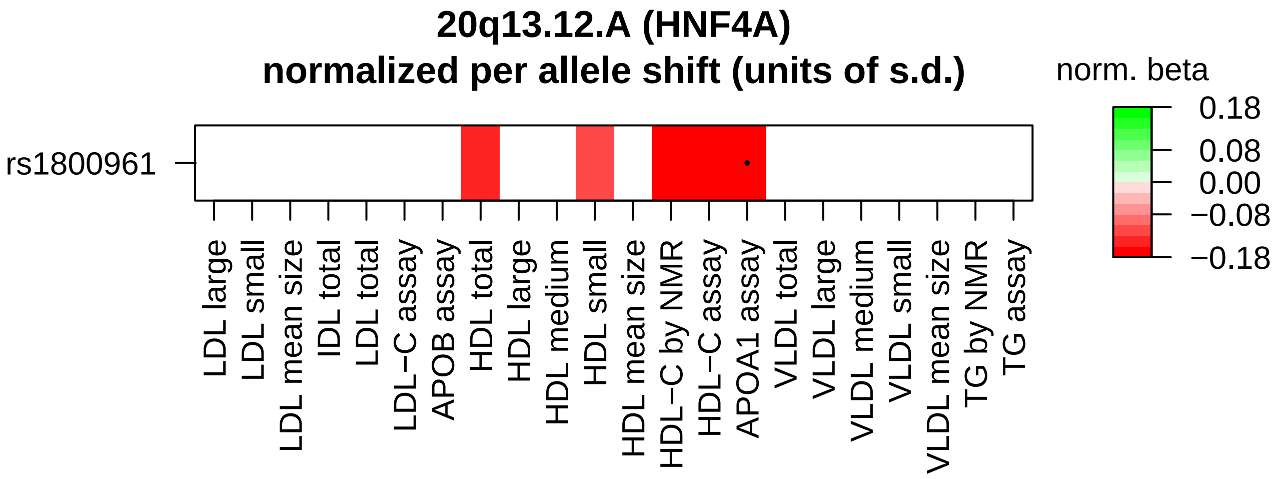

20q13.12.A (HNF4A)
normalized per allele shift (units of s.d.)
norm. beta
0.18
0.08
0.00
−0.08
−0.18
rs1800961
LDL large
LDL small
LDL mean size
IDL total
LDL total
LDL−C assay
APOB assay
HDL total
HDL large
HDL medium
HDL small
HDL mean size
HDL−C by NMR
HDL−C assay
APOA1 assay
VLDL total
VLDL large
VLDL medium
VLDL small
VLDL mean size
TG by NMR
TG assay

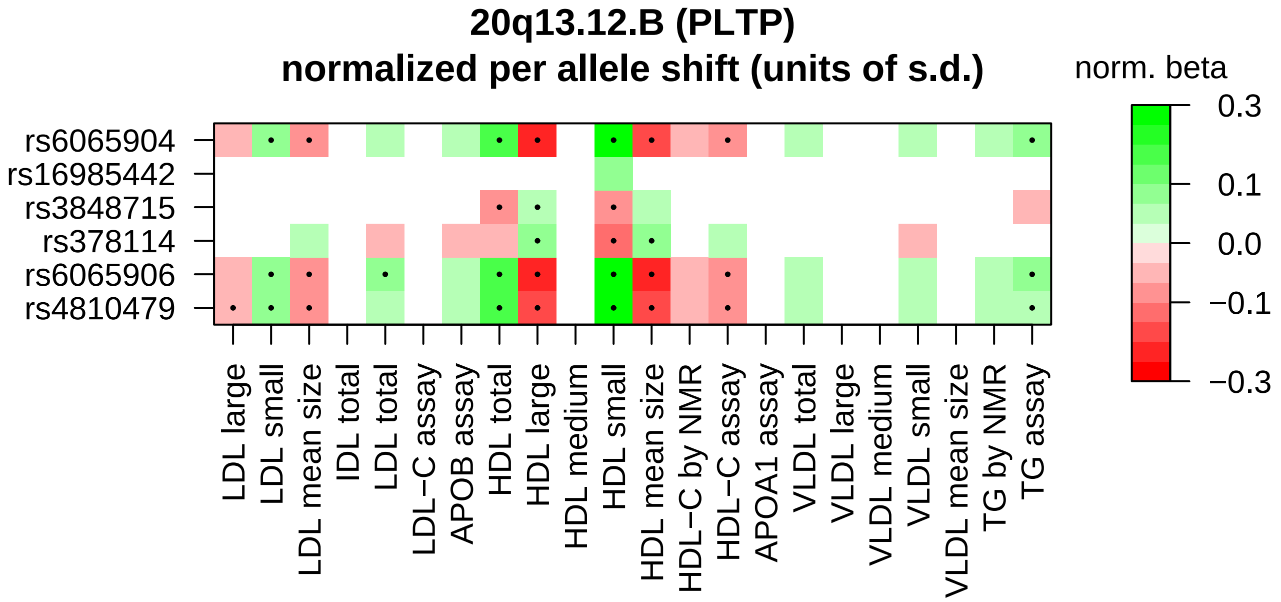
20q13.12.B (PLTP)
normalized per allele shift (units of s.d.)
norm. beta
0.3
0.1
0.0
−0.1
−0.3
rs6065904
rs16985442
rs3848715
rs378114
rs6065906
rs4810479
LDL large
LDL small
LDL mean size
IDL total
LDL total
LDL−C assay
APOB assay
HDL total
HDL large
HDL medium
HDL small
HDL mean size
HDL−C by NMR
HDL−C assay
APOA1 assay
VLDL total
VLDL large
VLDL medium
VLDL small
VLDL mean size
TG by NMR
TG assay
