# Supplementary material for: Forty-Three Loci Associated with Plasma Lipoprotein Size, Concentration, and Cholesterol Content in Genome-Wide Analysis
Source: PLoS Genet. 2009 Nov 20;5(11):e1000730. doi: 10.1371/journal.pgen.1000730 (PMC2777390; doi:10.1371/journal.pgen.1000730)
Supplement: Table S1 — Best genome-wide associations with the lipoprotein fractions at each candidate locus. (1.10 MB DOC) [file pgen.1000730.s005.doc]

Table S1. Best genomewide (*P*<5x10^-8^) associations at each locus among the 22 lipoprotein measures in primary analysis of the whole sample (all) and the fasting sub-sample (fasting).

|  |  |  |  |  |  |  |  |  |  |  |  |  |  |  |  | median (N) | | |
| --- | --- | --- | --- | --- | --- | --- | --- | --- | --- | --- | --- | --- | --- | --- | --- | --- | --- | --- |
| locus | *genes* | SNP | CHR | POS | Lipoprotein  fraction | sample | A1^a^ | A2^a^ | MAF^b^ | HWE  p-value^c^ | N^d^ | BETA^e^ | SE^e^ | P^e^ | R2^f^ | A2A2 | A2A1 | A1A1 |
| 1p32.3 | *PCSK9* | rs11591147 | 1 | 55278234 | LDL large | all | A | C | 0.02 | 0.369 | 16826 | -66.00 | 9.00 | 2.20E-13 | 0.32 | 542 (16029) | 472 (569) | 436 (7) |
| 1p32.3 |  | rs11591147 | 1 | 55278234 | LDL total | all | A | C | 0.02 | 0.369 | 16826 | -0.12 | 0.01 | 7.00E-22 | 0.55 | 1278 (16029) | 1122 (569) | 987 (7) |
| 1p32.3 |  | rs11591147 | 1 | 55278234 | LDL-C assay | all | A | C | 0.02 | 0.369 | 16793 | -16.00 | 1.40 | 1.30E-28 | 0.73 | 122 (15998) | 107 (567) | 90 (7) |
| 1p32.3 |  | rs11591147 | 1 | 55278234 | APOB assay | all | A | C | 0.02 | 0.369 | 16709 | -12.00 | 1.10 | 7.80E-28 | 0.71 | 99 (15916) | 90 (568) | 77 (7) |
| 1p32.3 |  | rs11591147 | 1 | 55278234 | VLDL total | all | A | C | 0.02 | 0.369 | 16826 | -7.70 | 1.30 | 1.10E-09 | 0.22 | 69 (16029) | 63 (569) | 56 (7) |
| 1p32.3 |  | rs11591147 | 1 | 55278234 | VLDL small | all | A | C | 0.02 | 0.369 | 16826 | -5.70 | 0.78 | 2.30E-13 | 0.32 | 45 (16029) | 40 (569) | 36 (7) |
| 1p32.3 |  | rs11591147 | 1 | 55278234 | TG by NMR | all | A | C | 0.02 | 0.369 | 16826 | -0.09 | 0.02 | 3.60E-08 | 0.18 | 109 (16029) | 101 (569) | 81 (7) |
| 1p32.3 |  | rs11591147 | 1 | 55278234 | LDL large | fasting | A | C | 0.02 | 0.369 | 12167 | -67.00 | 10.00 | 1.20E-10 | 0.34 | 548 (11576) | 475 (424) | 387 (5) |
| 1p32.3 |  | rs11591147 | 1 | 55278234 | LDL total | fasting | A | C | 0.02 | 0.369 | 12167 | -0.13 | 0.02 | 8.20E-18 | 0.60 | 1281 (11576) | 1120 (424) | 1046 (5) |
| 1p32.3 |  | rs11591147 | 1 | 55278234 | LDL-C assay | fasting | A | C | 0.02 | 0.369 | 12140 | -16.00 | 1.60 | 1.30E-23 | 0.82 | 123 (11551) | 107 (422) | 90 (5) |
| 1p32.3 |  | rs11591147 | 1 | 55278234 | APOB assay | fasting | A | C | 0.02 | 0.369 | 12082 | -12.00 | 1.30 | 5.30E-23 | 0.80 | 100 (11494) | 89 (423) | 77 (5) |
| 1p32.3 |  | rs11591147 | 1 | 55278234 | VLDL total | fasting | A | C | 0.02 | 0.369 | 12167 | -8.30 | 1.50 | 3.00E-08 | 0.25 | 69 (11576) | 62 (424) | 52 (5) |
| 1p32.3 |  | rs11591147 | 1 | 55278234 | VLDL small | fasting | A | C | 0.02 | 0.369 | 12167 | -6.30 | 0.92 | 4.60E-12 | 0.39 | 45 (11576) | 40 (424) | 36 (5) |
| 1p31.3 | *ANGPTL3* | rs1167998 | 1 | 62704219 | VLDL small | fasting | C | A | 0.34 | 0.656 | 12131 | -1.50 | 0.26 | 4.10E-09 | 0.28 | 46 (5243) | 45 (5360) | 42 (1362) |
| 1p31.3 |  | rs10889353 | 1 | 62890783 | VLDL total | all | C | A | 0.33 | 0.921 | 16831 | -2.70 | 0.35 | 1.30E-14 | 0.35 | 71 (7354) | 67 (7399) | 66 (1855) |
| 1p31.3 |  | rs10889353 | 1 | 62890783 | VLDL medium | all | C | A | 0.33 | 0.921 | 16831 | -1.20 | 0.18 | 2.80E-12 | 0.29 | 22 (7354) | 20 (7399) | 19 (1855) |
| 1p31.3 |  | rs10889353 | 1 | 62890783 | VLDL small | all | C | A | 0.33 | 0.921 | 16831 | -1.30 | 0.21 | 7.30E-10 | 0.23 | 46 (7354) | 44 (7399) | 43 (1855) |
| 1p31.3 |  | rs10889353 | 1 | 62890783 | TG by NMR | all | C | A | 0.33 | 0.921 | 16831 | -0.03 | 0.00 | 1.20E-13 | 0.33 | 112 (7354) | 107 (7399) | 104 (1855) |
| 1p31.3 |  | rs10889353 | 1 | 62890783 | VLDL total | fasting | C | A | 0.33 | 0.921 | 12171 | -3.00 | 0.42 | 1.40E-12 | 0.41 | 71 (5376) | 67 (5329) | 66 (1303) |
| 1p31.3 |  | rs10889353 | 1 | 62890783 | VLDL medium | fasting | C | A | 0.33 | 0.921 | 12171 | -1.30 | 0.21 | 3.00E-10 | 0.33 | 21 (5376) | 20 (5329) | 19 (1303) |
| 1p31.3 |  | rs10889353 | 1 | 62890783 | TG by NMR | fasting | C | A | 0.33 | 0.921 | 12171 | -0.04 | 0.01 | 1.60E-11 | 0.37 | 110 (5376) | 105 (5329) | 103 (1303) |
| 1p13.3 | *CELSR2/PSRC1/SPRT1* | rs646776 | 1 | 109620052 | LDL large | all | G | A | 0.22 | 0.284 | 16791 | -16.00 | 2.80 | 6.00E-09 | 0.20 | 547 (9998) | 529 (5716) | 515 (850) |
| 1p13.3 |  | rs646776 | 1 | 109620052 | LDL small | all | G | A | 0.22 | 0.284 | 16791 | -41.00 | 6.00 | 9.20E-12 | 0.28 | 659 (9998) | 641 (5716) | 602 (850) |
| 1p13.3 |  | rs646776 | 1 | 109620052 | LDL total | all | G | A | 0.22 | 0.284 | 16791 | -0.04 | 0.00 | 2.40E-27 | 0.70 | 1295 (9998) | 1254 (5716) | 1184 (850) |
| 1p13.3 |  | rs646776 | 1 | 109620052 | LDL-C assay | all | G | A | 0.22 | 0.284 | 16758 | -6.20 | 0.43 | 5.00E-46 | 1.20 | 124 (9980) | 118 (5702) | 111 (849) |
| 1p13.3 |  | rs646776 | 1 | 109620052 | APOB assay | all | G | A | 0.22 | 0.284 | 16674 | -5.20 | 0.34 | 1.80E-53 | 1.40 | 104 (9924) | 97 (5683) | 92 (843) |
| 1p13.3 |  | rs646776 | 1 | 109620052 | VLDL total | all | G | A | 0.22 | 0.284 | 16791 | -2.60 | 0.39 | 7.10E-11 | 0.25 | 70 (9998) | 68 (5716) | 64 (850) |
| 1p13.3 |  | rs646776 | 1 | 109620052 | VLDL small | all | G | A | 0.22 | 0.284 | 16791 | -2.20 | 0.24 | 6.30E-19 | 0.47 | 46 (9998) | 44 (5716) | 42 (850) |
| 1p13.3 |  | rs646776 | 1 | 109620052 | LDL small | fasting | G | A | 0.22 | 0.284 | 12139 | -42.00 | 7.20 | 5.00E-09 | 0.28 | 657 (7164) | 636 (4206) | 589 (604) |
| 1p13.3 |  | rs646776 | 1 | 109620052 | LDL total | fasting | G | A | 0.22 | 0.284 | 12139 | -0.04 | 0.00 | 2.20E-20 | 0.70 | 1298 (7164) | 1257 (4206) | 1186 (604) |
| 1p13.3 |  | rs646776 | 1 | 109620052 | LDL-C assay | fasting | G | A | 0.22 | 0.284 | 12112 | -6.10 | 0.51 | 1.20E-32 | 1.20 | 126 (7149) | 120 (4195) | 112 (603) |
| 1p13.3 |  | rs646776 | 1 | 109620052 | APOB assay | fasting | G | A | 0.22 | 0.284 | 12054 | -5.30 | 0.40 | 3.70E-39 | 1.40 | 104 (7114) | 97 (4179) | 92 (598) |
| 1p13.3 |  | rs646776 | 1 | 109620052 | VLDL small | fasting | G | A | 0.22 | 0.284 | 12139 | -2.10 | 0.29 | 1.60E-13 | 0.45 | 46 (7164) | 44 (4206) | 41 (604) |
| 1q23.3 | *APOA2* | rs4073054 | 1 | 159467110 | HDL medium | all | C | A | 0.38 | 0.755 | 14703 | -0.09 | 0.01 | 5.70E-11 | 0.29 | 3 (6382) | 3 (7787) | 2 (2442) |
| 1q23.3 |  | rs4073054 | 1 | 159467110 | HDL medium | fasting | C | A | 0.38 | 0.755 | 10615 | -0.09 | 0.02 | 2.00E-08 | 0.30 | 3 (4587) | 2 (5657) | 2 (1763) |
| 2p24.1 | *APOB* | rs6754295 | 2 | 21059687 | VLDL small | fasting | C | A | 0.23 | 0.386 | 12178 | -4.10 | 0.28 | 3.90E-47 | 1.70 | 47 (7048) | 43 (4328) | 38 (638) |
| 2p24.1 |  | rs676210 | 2 | 21085028 | VLDL total | all | A | G | 0.21 | 0.349 | 16844 | -6.40 | 0.40 | 8.60E-56 | 1.50 | 71 (10351) | 66 (5561) | 57 (707) |
| 2p24.1 |  | rs676210 | 2 | 21085028 | VLDL small | all | A | G | 0.21 | 0.349 | 16844 | -4.20 | 0.25 | 3.70E-64 | 1.70 | 47 (10351) | 43 (5561) | 37 (707) |
| 2p24.1 |  | rs676210 | 2 | 21085028 | VLDL mean size | all | A | G | 0.21 | 0.349 | 16844 | 0.64 | 0.10 | 2.10E-10 | 0.24 | 46 (10351) | 47 (5561) | 48 (707) |
| 2p24.1 |  | rs676210 | 2 | 21085028 | VLDL mean size | fasting | A | G | 0.21 | 0.349 | 12180 | 0.66 | 0.12 | 1.00E-08 | 0.27 | 46 (7500) | 46 (4013) | 48 (503) |
| 2p24.1 |  | rs676210 | 2 | 21085028 | TG assay | fasting | A | G | 0.21 | 0.337 | 12152 | -0.05 | 0.01 | 1.50E-09 | 0.30 | 116 (7484) | 110 (4003) | 107 (501) |
| 2p24.1 |  | rs673548 | 2 | 21091048 | LDL mean size | all | A | G | 0.21 | 0.372 | 16830 | 0.05 | 0.01 | 4.40E-08 | 0.18 | 21 (10345) | 21 (5555) | 22 (706) |
| 2p24.1 |  | rs673548 | 2 | 21091048 | VLDL medium | all | A | G | 0.21 | 0.372 | 16830 | -2.00 | 0.20 | 1.60E-22 | 0.56 | 22 (10345) | 20 (5555) | 18 (706) |
| 2p24.1 |  | rs673548 | 2 | 21091048 | TG by NMR | all | A | G | 0.21 | 0.372 | 16830 | -0.05 | 0.01 | 1.70E-18 | 0.46 | 111 (10345) | 106 (5555) | 100 (706) |
| 2p24.1 |  | rs673548 | 2 | 21091048 | TG assay | all | A | G | 0.21 | 0.372 | 16797 | -0.04 | 0.01 | 4.30E-10 | 0.23 | 119 (10324) | 115 (5545) | 109 (704) |
| 2p24.1 |  | rs673548 | 2 | 21091048 | VLDL total | fasting | A | G | 0.21 | 0.372 | 12170 | -6.60 | 0.49 | 4.80E-42 | 1.50 | 71 (7497) | 65 (4009) | 56 (501) |
| 2p24.1 |  | rs673548 | 2 | 21091048 | VLDL medium | fasting | A | G | 0.21 | 0.372 | 12170 | -2.20 | 0.24 | 4.00E-19 | 0.65 | 21 (7497) | 19 (4009) | 17 (501) |
| 2p24.1 |  | rs673548 | 2 | 21091048 | TG by NMR | fasting | A | G | 0.21 | 0.372 | 12170 | -0.05 | 0.01 | 1.70E-14 | 0.48 | 110 (7497) | 105 (4009) | 100 (501) |
| 2p24.1 |  | rs1367117 | 2 | 21117404 | LDL-C assay | all | A | G | 0.32 | 0.812 | 16812 | 4.00 | 0.39 | 6.50E-25 | 0.63 | 119 (7692) | 123 (7228) | 127 (1667) |
| 2p24.1 |  | rs1367117 | 2 | 21117404 | LDL-C assay | fasting | A | G | 0.32 | 0.812 | 12153 | 3.90 | 0.46 | 2.10E-17 | 0.59 | 120 (5556) | 124 (5219) | 128 (1214) |
| 2p24.1 |  | rs1713222 | 2 | 21124827 | LDL large | all | A | G | 0.15 | 0.185 | 16789 | -24.00 | 3.20 | 1.80E-13 | 0.32 | 548 (11961) | 520 (4202) | 504 (403) |
| 2p24.1 |  | rs1713222 | 2 | 21124827 | LDL large | fasting | A | G | 0.15 | 0.185 | 12141 | -25.00 | 3.80 | 7.10E-11 | 0.35 | 554 (8664) | 529 (3017) | 506 (299) |
| 2p24.1 |  | rs312985 | 2 | 21232309 | LDL total | fasting | A | G | 0.20 | 0.195 | 12162 | -0.04 | 0.00 | 1.20E-17 | 0.60 | 1296 (7759) | 1251 (3746) | 1204 (493) |
| 2p24.1 |  | rs506585 | 2 | 21250686 | LDL total | all | G | A | 0.20 | 0.166 | 16842 | -0.04 | 0.00 | 1.50E-22 | 0.56 | 1292 (10705) | 1247 (5232) | 1198 (680) |
| 2p24.1 |  | rs506585 | 2 | 21250686 | APOB assay | all | G | A | 0.20 | 0.166 | 16725 | -4.10 | 0.35 | 2.80E-31 | 0.80 | 103 (10638) | 97 (5189) | 92 (676) |
| 2p24.1 |  | rs506585 | 2 | 21250686 | APOB assay | fasting | G | A | 0.20 | 0.166 | 12093 | -4.40 | 0.42 | 1.20E-25 | 0.90 | 104 (7718) | 97 (3718) | 94 (495) |
| 2p23.3 | *GCKR* | rs1260326 | 2 | 27584443 | HDL total | all | A | G | 0.41 | 0.550 | 16682 | 0.80 | 0.06 | 6.30E-36 | 0.93 | 34 (5735) | 35 (7915) | 36 (2812) |
| 2p23.3 |  | rs1260326 | 2 | 27584443 | HDL small | All | A | G | 0.41 | 0.550 | 16682 | 0.56 | 0.06 | 8.40E-21 | 0.52 | 23 (5735) | 24 (7915) | 24 (2812) |
| 2p23.3 |  | rs1260326 | 2 | 27584443 | HDL mean size | all | A | G | 0.41 | 0.550 | 16682 | -0.03 | 0.00 | 7.20E-10 | 0.23 | 9 (5735) | 9 (7915) | 9 (2812) |
| 2p23.3 |  | rs1260326 | 2 | 27584443 | APOA1 assay | all | A | G | 0.41 | 0.550 | 16571 | 1.70 | 0.25 | 2.90E-11 | 0.27 | 149 (5696) | 150 (7869) | 152 (2789) |
| 2p23.3 |  | rs1260326 | 2 | 27584443 | VLDL total | all | A | G | 0.41 | 0.550 | 16682 | 2.80 | 0.34 | 7.80E-17 | 0.42 | 67 (5735) | 69 (7915) | 72 (2812) |
| 2p23.3 |  | rs1260326 | 2 | 27584443 | VLDL large | all | A | G | 0.41 | 0.550 | 16682 | 0.34 | 0.03 | 2.80E-28 | 0.73 | 1 (5735) | 2 (7915) | 2 (2812) |
| 2p23.3 |  | rs1260326 | 2 | 27584443 | VLDL medium | all | A | G | 0.41 | 0.550 | 16682 | 1.40 | 0.17 | 3.70E-17 | 0.42 | 20 (5735) | 21 (7915) | 22 (2812) |
| 2p23.3 |  | rs1260326 | 2 | 27584443 | VLDL mean size | all | A | G | 0.41 | 0.550 | 16682 | 0.67 | 0.08 | 7.60E-16 | 0.39 | 46 (5735) | 47 (7915) | 47 (2812) |
| 2p23.3 |  | rs1260326 | 2 | 27584443 | TG by NMR | all | A | G | 0.41 | 0.550 | 16682 | 0.05 | 0.00 | 2.90E-35 | 0.92 | 104 (5735) | 110 (7915) | 116 (2812) |
| 2p23.3 |  | rs1260326 | 2 | 27584443 | TG assay | all | A | G | 0.41 | 0.550 | 16650 | 0.07 | 0.01 | 1.00E-37 | 0.98 | 112 (5722) | 119 (7901) | 129 (2807) |
| 2p23.3 |  | rs1260326 | 2 | 27584443 | HDL total | fasting | A | G | 0.41 | 0.550 | 12067 | 0.82 | 0.08 | 8.00E-28 | 0.99 | 34 (4116) | 35 (5739) | 36 (2052) |
| 2p23.3 |  | rs1260326 | 2 | 27584443 | APOA1 assay | fasting | A | G | 0.41 | 0.550 | 11985 | 1.80 | 0.30 | 3.40E-09 | 0.29 | 149 (4088) | 150 (5703) | 151 (2036) |
| 2p23.3 |  | rs1260326 | 2 | 27584443 | VLDL large | fasting | A | G | 0.41 | 0.550 | 12067 | 0.36 | 0.04 | 3.60E-24 | 0.85 | 1 (4116) | 1 (5739) | 2 (2052) |
| 2p23.3 |  | rs1260326 | 2 | 27584443 | VLDL medium | fasting | A | G | 0.41 | 0.550 | 12067 | 1.60 | 0.20 | 6.10E-15 | 0.50 | 19 (4116) | 21 (5739) | 22 (2052) |
| 2p23.3 |  | rs1260326 | 2 | 27584443 | VLDL mean size | fasting | A | G | 0.41 | 0.550 | 12067 | 0.71 | 0.10 | 6.20E-14 | 0.47 | 45 (4116) | 46 (5739) | 47 (2052) |
| 2p23.3 |  | rs1260326 | 2 | 27584443 | TG by NMR | fasting | A | G | 0.41 | 0.550 | 12067 | 0.06 | 0.01 | 3.50E-29 | 1.00 | 102 (4116) | 108 (5739) | 114 (2052) |
| 2p23.3 |  | rs1260326 | 2 | 27584443 | TG assay | fasting | A | G | 0.41 | 0.550 | 12040 | 0.08 | 0.01 | 4.50E-32 | 1.10 | 107 (4103) | 115 (5730) | 125 (2047) |
| 2p23.3 |  | rs780094 | 2 | 27594740 | LDL small | all | A | G | 0.40 | 0.224 | 16841 | 46.00 | 5.10 | 2.60E-19 | 0.48 | 627 (6066) | 665 (7863) | 664 (2687) |
| 2p23.3 |  | rs780094 | 2 | 27594740 | LDL mean size | all | A | G | 0.40 | 0.224 | 16841 | -0.05 | 0.01 | 3.30E-12 | 0.29 | 21 (6066) | 21 (7863) | 21 (2687) |
| 2p23.3 |  | rs780094 | 2 | 27594740 | IDL total | all | A | G | 0.40 | 0.224 | 14345 | 0.07 | 0.01 | 7.00E-10 | 0.26 | 30 (6066) | 32 (7863) | 36 (2687) |
| 2p23.3 |  | rs780094 | 2 | 27594740 | LDL total | all | A | G | 0.40 | 0.224 | 16841 | 0.03 | 0.00 | 3.80E-19 | 0.47 | 1246 (6066) | 1282 (7863) | 1314 (2687) |
| 2p23.3 |  | rs780094 | 2 | 27594740 | APOB assay | all | A | G | 0.40 | 0.224 | 16724 | 2.50 | 0.29 | 1.10E-17 | 0.44 | 97 (6020) | 99 (7818) | 104 (2664) |
| 2p23.3 |  | rs780094 | 2 | 27594740 | LDL small | fasting | A | G | 0.40 | 0.224 | 12176 | 52.00 | 6.00 | 8.60E-18 | 0.60 | 619 (4349) | 663 (5698) | 671 (1965) |
| 2p23.3 |  | rs780094 | 2 | 27594740 | LDL mean size | fasting | A | G | 0.40 | 0.224 | 12176 | -0.07 | 0.01 | 9.80E-13 | 0.42 | 21 (4349) | 21 (5698) | 21 (1965) |
| 2p23.3 |  | rs780094 | 2 | 27594740 | IDL total | fasting | A | G | 0.40 | 0.224 | 10525 | 0.07 | 0.01 | 4.50E-08 | 0.28 | 32 (4349) | 35 (5698) | 39 (1965) |
| 2p23.3 |  | rs780094 | 2 | 27594740 | LDL total | fasting | A | G | 0.40 | 0.224 | 12176 | 0.03 | 0.00 | 2.90E-16 | 0.55 | 1246 (4349) | 1285 (5698) | 1323 (1965) |
| 2p23.3 |  | rs780094 | 2 | 27594740 | APOB assay | fasting | A | G | 0.40 | 0.224 | 12091 | 2.50 | 0.34 | 2.70E-13 | 0.44 | 98 (4316) | 100 (5664) | 105 (1949) |
| 2p23.3 |  | rs780094 | 2 | 27594740 | HDL small | fasting | A | G | 0.40 | 0.224 | 12176 | 0.60 | 0.07 | 3.10E-17 | 0.58 | 24 (4349) | 24 (5698) | 24 (1965) |
| 2p23.3 |  | rs780094 | 2 | 27594740 | HDL mean size | fasting | A | G | 0.40 | 0.224 | 12176 | -0.03 | 0.01 | 1.70E-09 | 0.30 | 9 (4349) | 9 (5698) | 9 (1965) |
| 2p23.3 |  | rs780094 | 2 | 27594740 | VLDL total | fasting | A | G | 0.40 | 0.224 | 12176 | 3.00 | 0.40 | 1.60E-13 | 0.45 | 67 (4349) | 69 (5698) | 71 (1965) |
| 2p21 | *ABCG5/8* | rs11887534 | 2 | 43919750 | LDL-C assay | all | G | C | 0.07 | 0.632 | 16810 | -4.90 | 0.73 | 2.80E-11 | 0.26 | 122 (14488) | 117 (2025) | 117 (72) |
| 2p21 |  | rs11887534 | 2 | 43919750 | APOB assay | all | G | C | 0.07 | 0.632 | 16726 | -3.60 | 0.57 | 3.50E-10 | 0.24 | 99 (14418) | 96 (2015) | 94 (71) |
| 2p21 |  | rs4299376 | 2 | 43926079 | LDL-C assay | fasting | C | A | 0.32 | 0.290 | 12148 | 2.60 | 0.46 | 2.20E-08 | 0.26 | 121 (5534) | 124 (5270) | 127 (1180) |
| 2q24.3 | *COBLL1/GRB14* | rs10490694 | 2 | 165256814 | HDL-C assay | all | A | G | 0.12 | 0.338 | 16777 | 1.30 | 0.23 | 4.80E-09 | 0.20 | 52 (12741) | 53 (3551) | 53 (262) |
| 3q22.3 | *PCCB* | rs3856637 | 3 | 137873884 | HDL small | all | G | A | 0.28 | 0.042 | 16838 | 0.37 | 0.07 | 1.30E-08 | 0.19 | 24 (8540) | 24 (6684) | 24 (1390) |
| 5q13.3 | *HMGCR* | rs3846662 | 5 | 74686839 | LDL-C assay | all | G | A | 0.43 | 0.752 | 16756 | 2.60 | 0.37 | 1.60E-12 | 0.30 | 119 (5407) | 122 (8038) | 123 (3084) |
| 5q13.3 |  | rs3846662 | 5 | 74686839 | LDL-C assay | fasting | G | A | 0.43 | 0.752 | 12116 | 2.70 | 0.43 | 3.60E-10 | 0.32 | 120 (3942) | 124 (5769) | 125 (2239) |
| 5q13.3 |  | rs5744680 | 5 | 74915645 | LDL large | all | A | G | 0.38 | 0.560 | 16722 | 14.00 | 2.40 | 8.80E-09 | 0.20 | 525 (6394) | 548 (7764) | 552 (2340) |
| 5q13.3 |  | rs5744680 | 5 | 74915645 | LDL large | fasting | A | G | 0.38 | 0.560 | 12084 | 17.00 | 2.80 | 3.70E-09 | 0.29 | 530 (4641) | 556 (5561) | 560 (1720) |
| 6p21.32 | *BTNL2* | rs2076530 | 6 | 32471793 | VLDL large | all | G | A | 0.43 | 0.588 | 16829 | 0.17 | 0.03 | 3.40E-08 | 0.18 | 1 (5310) | 1 (8224) | 2 (3070) |
| 6p21.32 |  | rs2076530 | 6 | 32471793 | TG by NMR | all | G | A | 0.43 | 0.588 | 16829 | 0.03 | 0.00 | 2.40E-09 | 0.21 | 106 (5310) | 110 (8224) | 112 (3070) |
| 6p21.32 |  | rs3129882 | 6 | 32517507 | VLDL large | fasting | G | A | 0.42 | 0.449 | 12176 | -0.20 | 0.04 | 3.60E-08 | 0.25 | 2 (4007) | 1 (5885) | 1 (2120) |
| 6p21.32 |  | rs3129882 | 6 | 32517507 | TG by NMR | fasting | G | A | 0.42 | 0.449 | 12176 | -0.03 | 0.01 | 1.70E-09 | 0.30 | 110 (4007) | 107 (5885) | 103 (2120) |
| 7q11.23 | *MLXIPL* | rs2240466 | 7 | 72494204 | VLDL medium | fasting | A | G | 0.12 | 0.774 | 12113 | -1.70 | 0.31 | 3.30E-08 | 0.25 | 21 (9319) | 19 (2454) | 17 (176) |
| 7q11.23 |  | rs714052 | 7 | 72502804 | LDL large | all | G | A | 0.12 | 0.751 | 16830 | 20.00 | 3.60 | 3.00E-08 | 0.18 | 534 (12888) | 555 (3485) | 574 (232) |
| 7q11.23 |  | rs714052 | 7 | 72502804 | VLDL total | all | G | A | 0.12 | 0.751 | 16830 | -3.30 | 0.51 | 1.60E-10 | 0.24 | 70 (12888) | 66 (3485) | 62 (232) |
| 7q11.23 |  | rs11974409 | 7 | 72627325 | LDL small | all | G | A | 0.19 | 0.629 | 16839 | -41.00 | 6.50 | 2.60E-10 | 0.24 | 663 (10922) | 628 (5119) | 621 (573) |
| 7q11.23 |  | rs11974409 | 7 | 72627325 | HDL small | all | G | A | 0.19 | 0.629 | 16839 | -0.44 | 0.08 | 5.70E-09 | 0.20 | 24 (10922) | 23 (5119) | 23 (573) |
| 7q11.23 |  | rs11974409 | 7 | 72627325 | VLDL large | all | G | A | 0.19 | 0.629 | 16839 | -0.23 | 0.04 | 6.30E-09 | 0.20 | 2 (10922) | 1 (5119) | 1 (573) |
| 7q11.23 |  | rs11974409 | 7 | 72627325 | VLDL medium | all | G | A | 0.19 | 0.629 | 16839 | -1.40 | 0.21 | 3.80E-11 | 0.26 | 21 (10922) | 20 (5119) | 18 (573) |
| 7q11.23 |  | rs11974409 | 7 | 72627325 | TG by NMR | all | G | A | 0.19 | 0.629 | 16839 | -0.04 | 0.01 | 2.10E-15 | 0.37 | 111 (10922) | 106 (5119) | 101 (573) |
| 7q11.23 |  | rs11974409 | 7 | 72627325 | TG assay | all | G | A | 0.19 | 0.629 | 16806 | -0.06 | 0.01 | 1.50E-18 | 0.46 | 120 (10899) | 114 (5109) | 104 (573) |
| 7q11.23 |  | rs11974409 | 7 | 72627325 | TG by NMR | fasting | G | A | 0.19 | 0.629 | 12178 | -0.04 | 0.01 | 8.40E-11 | 0.35 | 109 (7909) | 104 (3682) | 99 (422) |
| 7q11.23 |  | rs11974409 | 7 | 72627325 | TG assay | fasting | G | A | 0.19 | 0.629 | 12151 | -0.06 | 0.01 | 3.20E-13 | 0.44 | 116 (7888) | 110 (3676) | 98 (422) |
| 7q11.23 |  | rs7777102 | 7 | 72695952 | LDL mean size | all | G | A | 0.13 | 0.974 | 16834 | 0.08 | 0.01 | 3.50E-11 | 0.26 | 21 (12652) | 21 (3691) | 21 (267) |
| 7q32.2 | *KLF14* | rs4731702 | 7 | 130083923 | LDL small | all | A | G | 0.49 | 0.203 | 16747 | -30.00 | 5.00 | 1.50E-09 | 0.22 | 672 (4335) | 648 (8167) | 635 (4023) |
| 7q32.2 |  | rs4731702 | 7 | 130083923 | LDL total | all | A | G | 0.49 | 0.203 | 16747 | -0.02 | 0.00 | 6.60E-09 | 0.20 | 1297 (4335) | 1269 (8167) | 1255 (4023) |
| 7q32.2 |  | rs4731702 | 7 | 130083923 | HDL mean size | all | A | G | 0.49 | 0.203 | 16747 | 0.03 | 0.00 | 3.90E-09 | 0.21 | 9 (4335) | 9 (8167) | 9 (4023) |
| 7q32.2 |  | rs4731702 | 7 | 130083923 | TG assay | all | A | G | 0.49 | 0.203 | 16714 | -0.03 | 0.01 | 2.20E-09 | 0.21 | 121 (4323) | 117 (8147) | 116 (4022) |
| 8p23.1 | *intergenic, PPP1R3B* | rs983309 | 8 | 9215141 | VLDL mean size | fasting | A | C | 0.11 | 0.145 | 12177 | 0.87 | 0.15 | 6.40E-09 | 0.28 | 46 (9464) | 47 (2421) | 47 (128) |
| 8p21.3 | *LPL* | rs328 | 8 | 19864003 | VLDL total | all | G | C | 0.11 | 1.000 | 16845 | -5.80 | 0.53 | 2.20E-27 | 0.70 | 70 (13239) | 65 (3189) | 55 (192) |
| 8p21.3 |  | rs328 | 8 | 19864003 | VLDL large | all | G | C | 0.11 | 1.000 | 16845 | -0.39 | 0.05 | 2.80E-15 | 0.37 | 2 (13239) | 1 (3189) | 0.9 (192) |
| 8p21.3 |  | rs328 | 8 | 19864003 | VLDL medium | all | G | C | 0.11 | 1.000 | 16845 | -3.10 | 0.27 | 2.90E-30 | 0.77 | 22 (13239) | 18 (3189) | 16 (192) |
| 8p21.3 |  | rs328 | 8 | 19864003 | VLDL small | all | G | C | 0.11 | 1.000 | 16845 | -2.30 | 0.33 | 1.40E-12 | 0.30 | 46 (13239) | 43 (3189) | 38 (192) |
| 8p21.3 |  | rs328 | 8 | 19864003 | TG by NMR | all | G | C | 0.11 | 1.000 | 16845 | -0.07 | 0.01 | 1.40E-25 | 0.65 | 111 (13239) | 103 (3189) | 95 (192) |
| 8p21.3 |  | rs328 | 8 | 19864003 | TG assay | all | G | C | 0.11 | 1.000 | 16812 | -0.09 | 0.01 | 2.40E-26 | 0.67 | 119 (13215) | 111 (3180) | 101 (192) |
| 8p21.3 |  | rs328 | 8 | 19864003 | VLDL total | fasting | G | C | 0.11 | 1.000 | 12180 | -5.60 | 0.63 | 1.80E-18 | 0.63 | 70 (9535) | 64 (2338) | 57 (143) |
| 8p21.3 |  | rs328 | 8 | 19864003 | VLDL small | fasting | G | C | 0.11 | 1.000 | 12180 | -2.20 | 0.39 | 9.20E-09 | 0.27 | 46 (9535) | 43 (2338) | 40 (143) |
| 8p21.3 |  | rs331 | 8 | 19864684 | LDL small | all | A | G | 0.27 | 0.751 | 16842 | -45.00 | 5.70 | 2.50E-15 | 0.37 | 673 (8803) | 632 (6593) | 599 (1221) |
| 8p21.3 |  | rs331 | 8 | 19864684 | HDL large | all | A | G | 0.27 | 0.751 | 16842 | 0.34 | 0.04 | 1.50E-16 | 0.40 | 7 (8803) | 8 (6593) | 8 (1221) |
| 8p21.3 |  | rs331 | 8 | 19864684 | HDL mean size | all | A | G | 0.27 | 0.751 | 16842 | 0.04 | 0.01 | 2.50E-14 | 0.34 | 9 (8803) | 9 (6593) | 9 (1221) |
| 8p21.3 |  | rs331 | 8 | 19864684 | HDL-C by NMR | all | A | G | 0.27 | 0.751 | 16842 | 1.20 | 0.16 | 1.10E-14 | 0.35 | 53 (8803) | 54 (6593) | 56 (1221) |
| 8p21.3 |  | rs331 | 8 | 19864684 | HDL-C assay | all | A | G | 0.27 | 0.751 | 16809 | 1.50 | 0.17 | 1.20E-17 | 0.43 | 51 (8786) | 53 (6578) | 55 (1220) |
| 8p21.3 |  | rs331 | 8 | 19864684 | APOA1 assay | all | A | G | 0.27 | 0.751 | 16729 | 1.80 | 0.28 | 1.50E-10 | 0.24 | 149 (8744) | 151 (6549) | 152 (1214) |
| 8p21.3 |  | rs331 | 8 | 19864684 | HDL large | fasting | A | G | 0.27 | 0.751 | 12179 | 0.37 | 0.05 | 3.60E-14 | 0.47 | 7 (6353) | 8 (4783) | 8 (879) |
| 8p21.3 |  | rs331 | 8 | 19864684 | HDL mean size | fasting | A | G | 0.27 | 0.751 | 12179 | 0.04 | 0.01 | 2.10E-11 | 0.37 | 9 (6353) | 9 (4783) | 9 (879) |
| 8p21.3 |  | rs331 | 8 | 19864684 | HDL-C by NMR | fasting | A | G | 0.27 | 0.751 | 12179 | 1.30 | 0.18 | 1.00E-11 | 0.38 | 53 (6353) | 54 (4783) | 56 (879) |
| 8p21.3 |  | rs331 | 8 | 19864684 | HDL-C assay | fasting | A | G | 0.27 | 0.751 | 12152 | 1.50 | 0.20 | 2.70E-14 | 0.48 | 52 (6339) | 53 (4771) | 56 (878) |
| 8p21.3 |  | rs331 | 8 | 19864684 | APOA1 assay | fasting | A | G | 0.27 | 0.751 | 12096 | 1.90 | 0.33 | 3.10E-09 | 0.29 | 148 (6314) | 151 (4746) | 152 (874) |
| 8p21.3 |  | rs1059611 | 8 | 19868842 | VLDL large | fasting | G | A | 0.11 | 0.942 | 12178 | -0.39 | 0.06 | 1.20E-12 | 0.41 | 1 (9394) | 1 (2463) | 0.7 (157) |
| 8p21.3 |  | rs1059611 | 8 | 19868842 | VLDL medium | fasting | G | A | 0.11 | 0.942 | 12178 | -2.90 | 0.31 | 1.50E-20 | 0.70 | 21 (9394) | 18 (2463) | 16 (157) |
| 8p21.3 |  | rs1059611 | 8 | 19868842 | TG by NMR | fasting | G | A | 0.11 | 0.942 | 12178 | -0.07 | 0.01 | 1.60E-17 | 0.59 | 109 (9394) | 102 (2463) | 95 (157) |
| 8p21.3 |  | rs2083637 | 8 | 19909454 | LDL large | all | G | A | 0.27 | 0.838 | 16839 | 18.00 | 2.60 | 5.00E-12 | 0.28 | 528 (8755) | 547 (6644) | 565 (1215) |
| 8p21.3 |  | rs2083637 | 8 | 19909454 | LDL mean size | all | G | A | 0.27 | 0.838 | 16839 | 0.07 | 0.01 | 1.40E-15 | 0.38 | 21 (8755) | 21 (6644) | 22 (1215) |
| 8p21.3 |  | rs2083637 | 8 | 19909454 | LDL large | fasting | G | A | 0.27 | 0.838 | 12174 | 19.00 | 3.10 | 9.40E-10 | 0.31 | 538 (6324) | 549 (4810) | 578 (876) |
| 8p21.3 |  | rs2083637 | 8 | 19909454 | LDL small | fasting | G | A | 0.27 | 0.838 | 12174 | -47.00 | 6.70 | 3.00E-12 | 0.40 | 667 (6324) | 636 (4810) | 584 (876) |
| 8p21.3 |  | rs2083637 | 8 | 19909454 | LDL mean size | fasting | G | A | 0.27 | 0.838 | 12174 | 0.07 | 0.01 | 6.30E-12 | 0.39 | 21 (6324) | 21 (4810) | 22 (876) |
| 8p21.3 |  | rs2083637 | 8 | 19909454 | TG assay | fasting | G | A | 0.27 | 0.838 | 12147 | -0.06 | 0.01 | 2.50E-19 | 0.66 | 117 (6312) | 110 (4796) | 104 (875) |
| 8q24.13 | *TRIB1* | rs6982636 | 8 | 126548496 | LDL total | all | A | G | 0.47 | 0.278 | 16798 | -0.02 | 0.00 | 2.40E-12 | 0.29 | 1293 (4643) | 1275 (8296) | 1242 (3635) |
| 8q24.13 |  | rs6982636 | 8 | 126548496 | APOB assay | all | A | G | 0.47 | 0.278 | 16682 | -1.90 | 0.28 | 7.50E-12 | 0.28 | 103 (4613) | 99 (8234) | 97 (3614) |
| 8q24.13 |  | rs6982636 | 8 | 126548496 | TG by NMR | all | A | G | 0.47 | 0.278 | 16798 | -0.03 | 0.00 | 8.50E-10 | 0.22 | 111 (4643) | 109 (8296) | 105 (3635) |
| 8q24.13 |  | rs6982636 | 8 | 126548496 | TG assay | all | A | G | 0.47 | 0.278 | 16765 | -0.03 | 0.01 | 1.20E-09 | 0.22 | 120 (4629) | 118 (8283) | 113 (3629) |
| 8q24.13 |  | rs6982636 | 8 | 126548496 | LDL small | fasting | A | G | 0.47 | 0.278 | 12148 | -37.00 | 6.00 | 6.00E-10 | 0.32 | 663 (3362) | 655 (5981) | 617 (2641) |
| 8q24.13 |  | rs6982636 | 8 | 126548496 | LDL total | fasting | A | G | 0.47 | 0.278 | 12148 | -0.03 | 0.00 | 1.20E-11 | 0.38 | 1302 (3362) | 1276 (5981) | 1233 (2641) |
| 8q24.13 |  | rs6982636 | 8 | 126548496 | APOB assay | fasting | A | G | 0.47 | 0.278 | 12064 | -2.10 | 0.34 | 4.20E-10 | 0.32 | 104 (3339) | 100 (5941) | 97 (2622) |
| 8q24.13 |  | rs10808546 | 8 | 126564999 | LDL small | all | A | G | 0.44 | 0.661 | 16516 | -36.00 | 5.10 | 2.40E-12 | 0.30 | 662 (5114) | 654 (7989) | 620 (3191) |
| 8q24.13 |  | rs10808546 | 8 | 126564999 | LDL mean size | all | A | G | 0.44 | 0.661 | 16516 | 0.05 | 0.01 | 5.20E-09 | 0.21 | 21 (5114) | 21 (7989) | 21 (3191) |
| 9q31.1 | *ABCA1* | rs3905000 | 9 | 106696890 | HDL medium | all | A | G | 0.14 | 0.975 | 14709 | 0.10 | 0.02 | 2.80E-08 | 0.21 | 3 (12295) | 3 (4008) | 3 (312) |
| 9q31.1 |  | rs2740486 | 9 | 106706333 | APOA1 assay | fasting | C | A | 0.47 | 0.824 | 12092 | -1.70 | 0.29 | 2.80E-09 | 0.29 | 152 (3372) | 149 (5977) | 148 (2581) |
| 9q31.1 |  | rs2515614 | 9 | 106724138 | HDL-C assay | all | C | A | 0.34 | 0.379 | 16789 | 0.90 | 0.16 | 2.30E-08 | 0.19 | 52 (7362) | 53 (7310) | 54 (1893) |
| 9q31.1 |  | rs2515614 | 9 | 106724138 | HDL-C assay | fasting | C | A | 0.34 | 0.379 | 12137 | 1.00 | 0.19 | 3.40E-08 | 0.25 | 52 (5316) | 53 (5305) | 53 (1352) |
| 9q34.2 | *ABO* | rs507666 | 9 | 135139219 | LDL large | all | A | G | 0.20 | 0.091 | 16669 | 18.00 | 2.90 | 1.00E-09 | 0.22 | 532 (10617) | 553 (5157) | 555 (673) |
| 9q34.2 |  | rs507666 | 9 | 135139219 | LDL-C assay | all | A | G | 0.20 | 0.091 | 16636 | 3.30 | 0.46 | 5.30E-13 | 0.31 | 120 (10594) | 124 (5147) | 123 (673) |
| 9q34.2 |  | rs507666 | 9 | 135139219 | VLDL small | all | A | G | 0.20 | 0.091 | 16669 | 1.90 | 0.26 | 2.50E-13 | 0.32 | 44 (10617) | 46 (5157) | 45 (673) |
| 9q34.2 |  | rs507666 | 9 | 135139219 | VLDL mean size | all | A | G | 0.20 | 0.091 | 16669 | -0.56 | 0.10 | 4.40E-08 | 0.18 | 47 (10617) | 46 (5157) | 46 (673) |
| 9q34.2 |  | rs507666 | 9 | 135139219 | LDL-C assay | fasting | A | G | 0.20 | 0.091 | 12018 | 3.30 | 0.54 | 7.70E-10 | 0.31 | 121 (7644) | 126 (3717) | 124 (494) |
| 9q34.2 |  | rs507666 | 9 | 135139219 | VLDL small | fasting | A | G | 0.20 | 0.091 | 12045 | 1.90 | 0.30 | 4.50E-10 | 0.32 | 44 (7663) | 47 (3725) | 45 (494) |
| 11q12.2 | *FADS1-3* | rs174537 | 11 | 61309255 | HDL medium | all | A | C | 0.33 | 0.436 | 14708 | 0.10 | 0.01 | 7.60E-14 | 0.38 | 2 (7373) | 3 (7375) | 3 (1867) |
| 11q12.2 |  | rs174537 | 11 | 61309255 | HDL medium | fasting | A | C | 0.33 | 0.436 | 10622 | 0.09 | 0.02 | 4.50E-09 | 0.32 | 2 (5346) | 3 (5304) | 3 (1363) |
| 11q12.2 |  | rs102275 | 11 | 61314378 | HDL large | all | G | A | 0.34 | 0.513 | 16835 | -0.30 | 0.04 | 1.40E-14 | 0.35 | 8 (7166) | 8 (7477) | 7 (1968) |
| 11q12.2 |  | rs174546 | 11 | 61326405 | HDL large | fasting | A | G | 0.34 | 0.655 | 12176 | -0.30 | 0.05 | 9.80E-11 | 0.34 | 8 (5322) | 7 (5327) | 7 (1363) |
| 11q12.2 |  | rs1535 | 11 | 61354547 | LDL large | all | G | A | 0.34 | 0.520 | 16826 | -15.00 | 2.50 | 1.00E-09 | 0.22 | 550 (7280) | 535 (7420) | 518 (1901) |
| 11q12.2 |  | rs1535 | 11 | 61354547 | HDL mean size | all | G | A | 0.34 | 0.520 | 16826 | -0.03 | 0.00 | 3.50E-12 | 0.29 | 9 (7280) | 9 (7420) | 9 (1901) |
| 11q12.2 |  | rs1535 | 11 | 61354547 | LDL large | fasting | G | A | 0.34 | 0.520 | 12164 | -16.00 | 2.90 | 4.40E-08 | 0.25 | 557 (5283) | 539 (5334) | 527 (1383) |
| 11q12.2 |  | rs1535 | 11 | 61354547 | HDL mean size | fasting | G | A | 0.34 | 0.520 | 12164 | -0.03 | 0.01 | 1.00E-08 | 0.27 | 9 (5283) | 9 (5334) | 9 (1383) |
| 11q23.3 | *APOA1-A5* | rs618923 | 11 | 116159368 | HDL-C assay | all | G | A | 0.25 | 0.829 | 16754 | 1.20 | 0.17 | 2.40E-12 | 0.29 | 52 (9229) | 53 (6235) | 54 (1064) |
| 11q23.3 |  | rs618923 | 11 | 116159368 | HDL-C assay | fasting | G | A | 0.25 | 0.829 | 12111 | 1.20 | 0.20 | 3.70E-09 | 0.29 | 52 (6662) | 53 (4536) | 54 (747) |
| 11q23.3 |  | rs3135506 | 11 | 116167616 | LDL small | all | G | C | 0.06 | 0.005 | 16837 | 105.00 | 11.00 | 5.70E-23 | 0.58 | 643 (14610) | 720 (1959) | 1095 (43) |
| 11q23.3 |  | rs3135506 | 11 | 116167616 | LDL mean size | all | G | C | 0.06 | 0.005 | 16837 | -0.13 | 0.02 | 7.40E-16 | 0.39 | 21 (14610) | 21 (1959) | 21 (43) |
| 11q23.3 |  | rs3135506 | 11 | 116167616 | LDL total | all | G | C | 0.06 | 0.005 | 16837 | 0.06 | 0.01 | 1.20E-18 | 0.46 | 1262 (14610) | 1357 (1959) | 1428 (43) |
| 11q23.3 |  | rs3135506 | 11 | 116167616 | APOB assay | all | G | C | 0.06 | 0.005 | 16720 | 5.80 | 0.60 | 2.30E-22 | 0.56 | 98 (14516) | 107 (1939) | 115 (43) |
| 11q23.3 |  | rs3135506 | 11 | 116167616 | VLDL total | all | G | C | 0.06 | 0.005 | 16837 | 9.50 | 0.69 | 7.00E-43 | 1.10 | 68 (14610) | 77 (1959) | 86 (43) |
| 11q23.3 |  | rs3135506 | 11 | 116167616 | VLDL large | all | G | C | 0.06 | 0.005 | 16837 | 0.57 | 0.06 | 3.60E-19 | 0.47 | 1 (14610) | 2 (1959) | 4 (43) |
| 11q23.3 |  | rs3135506 | 11 | 116167616 | VLDL medium | all | G | C | 0.06 | 0.005 | 16837 | 5.00 | 0.35 | 5.10E-46 | 1.20 | 20 (14610) | 25 (1959) | 34 (43) |
| 11q23.3 |  | rs3135506 | 11 | 116167616 | VLDL small | all | G | C | 0.06 | 0.005 | 16837 | 4.00 | 0.43 | 1.10E-20 | 0.52 | 44 (14610) | 48 (1959) | 50 (43) |
| 11q23.3 |  | rs3135506 | 11 | 116167616 | TG by NMR | all | G | C | 0.06 | 0.005 | 16837 | 0.12 | 0.01 | 7.80E-42 | 1.10 | 107 (14610) | 123 (1959) | 164 (43) |
| 11q23.3 |  | rs3135506 | 11 | 116167616 | TG assay | all | G | C | 0.06 | 0.005 | 16804 | 0.14 | 0.01 | 4.00E-36 | 0.93 | 116 (14582) | 132 (1955) | 178 (42) |
| 11q23.3 |  | rs3135506 | 11 | 116167616 | LDL small | fasting | G | C | 0.06 | 0.005 | 12177 | 101.00 | 13.00 | 1.40E-15 | 0.52 | 639 (10563) | 716 (1420) | 1095 (29) |
| 11q23.3 |  | rs3135506 | 11 | 116167616 | LDL mean size | fasting | G | C | 0.06 | 0.005 | 12177 | -0.13 | 0.02 | 7.20E-12 | 0.39 | 21 (10563) | 21 (1420) | 21 (29) |
| 11q23.3 |  | rs3135506 | 11 | 116167616 | LDL total | fasting | G | C | 0.06 | 0.005 | 12177 | 0.06 | 0.01 | 8.30E-12 | 0.38 | 1265 (10563) | 1348 (1420) | 1377 (29) |
| 11q23.3 |  | rs3135506 | 11 | 116167616 | APOB assay | fasting | G | C | 0.06 | 0.005 | 12092 | 5.20 | 0.71 | 1.80E-13 | 0.45 | 99 (10495) | 107 (1405) | 113 (29) |
| 11q23.3 |  | rs3135506 | 11 | 116167616 | VLDL total | fasting | G | C | 0.06 | 0.005 | 12177 | 9.70 | 0.83 | 2.90E-31 | 1.10 | 67 (10563) | 77 (1420) | 87 (29) |
| 11q23.3 |  | rs3135506 | 11 | 116167616 | VLDL large | fasting | G | C | 0.06 | 0.005 | 12177 | 0.53 | 0.07 | 1.10E-12 | 0.41 | 1 (10563) | 2 (1420) | 4 (29) |
| 11q23.3 |  | rs3135506 | 11 | 116167616 | VLDL medium | fasting | G | C | 0.06 | 0.005 | 12177 | 5.00 | 0.42 | 1.20E-33 | 1.20 | 20 (10563) | 25 (1420) | 35 (29) |
| 11q23.3 |  | rs3135506 | 11 | 116167616 | VLDL small | fasting | G | C | 0.06 | 0.005 | 12177 | 4.10 | 0.51 | 5.60E-16 | 0.54 | 45 (10563) | 49 (1420) | 50 (29) |
| 11q23.3 |  | rs3135506 | 11 | 116167616 | TG by NMR | fasting | G | C | 0.06 | 0.005 | 12177 | 0.12 | 0.01 | 9.20E-29 | 1.00 | 105 (10563) | 121 (1420) | 160 (29) |
| 11q23.3 |  | rs662799 | 11 | 116168916 | TG assay | fasting | G | A | 0.06 | 0.141 | 12152 | 0.14 | 0.01 | 3.50E-27 | 0.95 | 112 (10565) | 126 (1365) | 140 (57) |
| 11q23.3 |  | rs12225230 | 11 | 116233839 | HDL-C by NMR | all | C | G | 0.18 | 0.146 | 16840 | 1.40 | 0.18 | 9.00E-15 | 0.36 | 53 (11162) | 54 (4946) | 56 (507) |
| 11q23.3 |  | rs12225230 | 11 | 116233839 | APOA1 assay | all | C | G | 0.18 | 0.146 | 16727 | 3.20 | 0.33 | 6.40E-23 | 0.58 | 149 (11091) | 152 (4914) | 155 (500) |
| 11q23.3 |  | rs12225230 | 11 | 116233839 | HDL-C by NMR | fasting | C | G | 0.18 | 0.146 | 12175 | 1.50 | 0.21 | 1.30E-11 | 0.38 | 53 (8036) | 54 (3610) | 56 (365) |
| 11q23.3 |  | rs12225230 | 11 | 116233839 | APOA1 assay | fasting | C | G | 0.18 | 0.146 | 12092 | 3.30 | 0.38 | 1.90E-17 | 0.60 | 149 (7985) | 151 (3586) | 154 (359) |
| 11q23.3 |  | rs518181 | 11 | 116277996 | HDL total | all | C | A | 0.37 | 0.588 | 16837 | 0.61 | 0.07 | 3.90E-21 | 0.53 | 34 (6734) | 35 (7660) | 36 (2219) |
| 11q23.3 |  | rs518181 | 11 | 116277996 | HDL small | all | C | A | 0.37 | 0.588 | 16837 | 0.68 | 0.06 | 1.50E-28 | 0.73 | 23 (6734) | 24 (7660) | 24 (2219) |
| 11q23.3 |  | rs518181 | 11 | 116277996 | HDL total | fasting | C | A | 0.37 | 0.588 | 12173 | 0.62 | 0.08 | 8.10E-16 | 0.53 | 34 (4862) | 35 (5553) | 36 (1595) |
| 11q23.3 |  | rs518181 | 11 | 116277996 | HDL small | fasting | C | A | 0.37 | 0.588 | 12173 | 0.67 | 0.07 | 6.80E-21 | 0.72 | 23 (4862) | 24 (5553) | 25 (1595) |
| 12q23.2 | *intergenic, ASCL1, PAH* | rs10778213 | 12 | 102019280 | HDL-C by NMR | all | G | A | 0.47 | 0.778 | 16843 | -0.79 | 0.14 | 1.80E-08 | 0.19 | 54 (4701) | 53 (8281) | 53 (3636) |
| 12q23.2 |  | rs1818702 | 12 | 102047684 | HDL total | all | A | G | 0.29 | 0.460 | 16844 | -0.42 | 0.07 | 9.40E-10 | 0.22 | 35 (8436) | 35 (6783) | 35 (1400) |
| 12q24.31.A | *HNF1A* | rs1169300 | 12 | 119915607 | LDL-C assay | all | A | G | 0.30 | 0.645 | 16807 | 2.40 | 0.40 | 1.40E-09 | 0.22 | 120 (8260) | 122 (6872) | 124 (1448) |
| 12q24.31.B | *CCDC92/DNAH10/ZNF664* | rs7307277 | 12 | 123041108 | LDL small | all | G | A | 0.34 | 0.974 | 16804 | -33.00 | 5.30 | 5.20E-10 | 0.23 | 667 (7198) | 638 (7442) | 623 (1938) |
| 12q24.31.B |  | rs7307277 | 12 | 123041108 | LDL mean size | all | G | A | 0.34 | 0.974 | 16804 | 0.05 | 0.01 | 1.00E-08 | 0.19 | 21 (7198) | 21 (7442) | 21 (1938) |
| 12q24.31.B |  | rs7307277 | 12 | 123041108 | LDL total | all | G | A | 0.34 | 0.974 | 16804 | -0.02 | 0.00 | 1.10E-08 | 0.19 | 1293 (7198) | 1262 (7442) | 1253 (1938) |
| 12q24.31.B |  | rs7307277 | 12 | 123041108 | HDL large | all | G | A | 0.34 | 0.974 | 16804 | 0.26 | 0.04 | 4.90E-11 | 0.26 | 7 (7198) | 8 (7442) | 8 (1938) |
| 12q24.31.B |  | rs7307277 | 12 | 123041108 | HDL mean size | all | G | A | 0.34 | 0.974 | 16804 | 0.03 | 0.00 | 2.90E-10 | 0.24 | 9 (7198) | 9 (7442) | 9 (1938) |
| 12q24.31.B |  | rs7307277 | 12 | 123041108 | TG assay | all | G | A | 0.34 | 0.974 | 16771 | -0.04 | 0.01 | 6.10E-10 | 0.23 | 120 (7183) | 117 (7427) | 113 (1935) |
| 12q24.31.B |  | rs7307277 | 12 | 123041108 | HDL large | fasting | G | A | 0.34 | 0.974 | 12149 | 0.25 | 0.05 | 2.90E-08 | 0.25 | 7 (5221) | 8 (5354) | 8 (1409) |
| 15q22.1 | *LIPC* | rs1532085 | 15 | 56470657 | IDL total | all | A | G | 0.37 | 0.987 | 14337 | 0.11 | 0.01 | 1.50E-19 | 0.57 | 29 (6531) | 33 (7751) | 39 (2324) |
| 15q22.1 |  | rs1532085 | 15 | 56470657 | HDL small | all | A | G | 0.37 | 0.987 | 16830 | -0.51 | 0.06 | 6.80E-17 | 0.41 | 24 (6531) | 24 (7751) | 23 (2324) |
| 15q22.1 |  | rs1532085 | 15 | 56470657 | IDL total | fasting | A | G | 0.37 | 0.987 | 10519 | 0.11 | 0.01 | 3.70E-16 | 0.63 | 31 (4772) | 36 (5605) | 42 (1629) |
| 15q22.1 |  | rs1532085 | 15 | 56470657 | HDL-C assay | fasting | A | G | 0.37 | 0.987 | 12142 | 1.40 | 0.18 | 9.00E-15 | 0.49 | 52 (4762) | 53 (5590) | 55 (1627) |
| 15q22.1 |  | rs1800588 | 15 | 56510966 | LDL large | all | A | G | 0.22 | 0.729 | 16844 | 44.00 | 2.80 | 9.40E-56 | 1.50 | 521 (10146) | 562 (5682) | 635 (791) |
| 15q22.1 |  | rs1800588 | 15 | 56510966 | LDL small | all | A | G | 0.22 | 0.729 | 16844 | -45.00 | 6.10 | 1.30E-13 | 0.33 | 671 (10146) | 622 (5682) | 580 (791) |
| 15q22.1 |  | rs1800588 | 15 | 56510966 | LDL mean size | all | A | G | 0.22 | 0.729 | 16844 | 0.12 | 0.01 | 1.50E-37 | 0.97 | 21 (10146) | 21 (5682) | 22 (791) |
| 15q22.1 |  | rs1800588 | 15 | 56510966 | HDL large | all | A | G | 0.22 | 0.729 | 16844 | 0.85 | 0.04 | 1.20E-81 | 2.20 | 7 (10146) | 8 (5682) | 9 (791) |
| 15q22.1 |  | rs1800588 | 15 | 56510966 | HDL medium | all | A | G | 0.22 | 0.729 | 14713 | -0.10 | 0.02 | 3.40E-10 | 0.27 | 3 (10146) | 3 (5682) | 2 (791) |
| 15q22.1 |  | rs1800588 | 15 | 56510966 | HDL mean size | all | A | G | 0.22 | 0.729 | 16844 | 0.10 | 0.01 | 8.00E-69 | 1.80 | 9 (10146) | 9 (5682) | 9 (791) |
| 15q22.1 |  | rs1800588 | 15 | 56510966 | HDL-C by NMR | all | A | G | 0.22 | 0.729 | 16844 | 2.30 | 0.17 | 7.90E-42 | 1.10 | 53 (10146) | 55 (5682) | 57 (791) |
| 15q22.1 |  | rs1800588 | 15 | 56510966 | HDL-C assay | all | A | G | 0.22 | 0.729 | 16811 | 1.80 | 0.18 | 3.60E-23 | 0.58 | 51 (10126) | 54 (5671) | 55 (789) |
| 15q22.1 |  | rs1800588 | 15 | 56510966 | APOA1 assay | all | A | G | 0.22 | 0.729 | 16731 | 3.80 | 0.30 | 8.20E-37 | 0.96 | 148 (10085) | 152 (5641) | 156 (783) |
| 15q22.1 |  | rs1800588 | 15 | 56510966 | LDL large | fasting | A | G | 0.22 | 0.729 | 12179 | 43.00 | 3.30 | 7.40E-39 | 1.40 | 527 (7404) | 568 (4012) | 640 (599) |
| 15q22.1 |  | rs1800588 | 15 | 56510966 | LDL small | fasting | A | G | 0.22 | 0.729 | 12179 | -43.00 | 7.20 | 1.50E-09 | 0.30 | 666 (7404) | 621 (4012) | 567 (599) |
| 15q22.1 |  | rs1800588 | 15 | 56510966 | LDL mean size | fasting | A | G | 0.22 | 0.729 | 12179 | 0.11 | 0.01 | 3.50E-25 | 0.88 | 21 (7404) | 22 (4012) | 22 (599) |
| 15q22.1 |  | rs1800588 | 15 | 56510966 | HDL large | fasting | A | G | 0.22 | 0.729 | 12179 | 0.80 | 0.05 | 4.00E-54 | 2.00 | 7 (7404) | 8 (4012) | 9 (599) |
| 15q22.1 |  | rs1800588 | 15 | 56510966 | HDL small | fasting | A | G | 0.22 | 0.729 | 12179 | -0.60 | 0.08 | 3.90E-13 | 0.43 | 24 (7404) | 24 (4012) | 22 (599) |
| 15q22.1 |  | rs1800588 | 15 | 56510966 | HDL mean size | fasting | A | G | 0.22 | 0.729 | 12179 | 0.09 | 0.01 | 1.50E-46 | 1.70 | 9 (7404) | 9 (4012) | 9 (599) |
| 15q22.1 |  | rs1800588 | 15 | 56510966 | HDL-C by NMR | fasting | A | G | 0.22 | 0.729 | 12179 | 2.10 | 0.20 | 3.30E-26 | 0.92 | 52 (7404) | 55 (4012) | 57 (599) |
| 15q22.1 |  | rs1800588 | 15 | 56510966 | APOA1 assay | fasting | A | G | 0.22 | 0.729 | 12096 | 3.60 | 0.35 | 2.10E-24 | 0.86 | 148 (7358) | 152 (3983) | 157 (593) |
| 16q13 | *CETP* | rs1800775 | 16 | 55552736 | LDL small | all | A | C | 0.49 | 0.442 | 16812 | -69.00 | 5.00 | 4.50E-42 | 1.10 | 703 (4365) | 653 (8315) | 590 (3910) |
| 16q13 |  | rs1800775 | 16 | 55552736 | LDL mean size | all | A | C | 0.49 | 0.442 | 16812 | 0.12 | 0.01 | 4.30E-51 | 1.30 | 21 (4365) | 21 (8315) | 22 (3910) |
| 16q13 |  | rs1800775 | 16 | 55552736 | IDL total | all | A | C | 0.49 | 0.442 | 14322 | -0.10 | 0.01 | 2.80E-19 | 0.56 | 37 (4365) | 32 (8315) | 26 (3910) |
| 16q13 |  | rs1800775 | 16 | 55552736 | HDL large | all | A | C | 0.49 | 0.442 | 16812 | 0.73 | 0.04 | 5.00E-87 | 2.30 | 7 (4365) | 8 (8315) | 8 (3910) |
| 16q13 |  | rs1800775 | 16 | 55552736 | HDL mean size | all | A | C | 0.49 | 0.442 | 16812 | 0.09 | 0.00 | 8.00E-78 | 2.10 | 9 (4365) | 9 (8315) | 9 (3910) |
| 16q13 |  | rs1800775 | 16 | 55552736 | HDL-C by NMR | all | A | C | 0.49 | 0.442 | 16812 | 2.70 | 0.14 | 1.40E-81 | 2.20 | 51 (4365) | 53 (8315) | 56 (3910) |
| 16q13 |  | rs1800775 | 16 | 55552736 | HDL-C assay | all | A | C | 0.49 | 0.442 | 16779 | 3.10 | 0.15 | 3.30E-93 | 2.50 | 50 (4353) | 52 (8297) | 55 (3907) |
| 16q13 |  | rs1800775 | 16 | 55552736 | APOA1 assay | all | A | C | 0.49 | 0.442 | 16699 | 4.10 | 0.25 | 1.40E-59 | 1.60 | 146 (4332) | 150 (8262) | 154 (3886) |
| 16q13 |  | rs1800775 | 16 | 55552736 | VLDL total | all | A | C | 0.49 | 0.442 | 16812 | -2.50 | 0.33 | 4.60E-14 | 0.34 | 71 (4365) | 69 (8315) | 65 (3910) |
| 16q13 |  | rs1800775 | 16 | 55552736 | VLDL small | all | A | C | 0.49 | 0.442 | 16812 | -1.90 | 0.20 | 4.70E-20 | 0.50 | 47 (4365) | 45 (8315) | 42 (3910) |
| 16q13 |  | rs1800775 | 16 | 55552736 | TG assay | all | A | C | 0.49 | 0.442 | 16779 | -0.03 | 0.01 | 4.30E-08 | 0.18 | 120 (4353) | 118 (8297) | 113 (3907) |
| 16q13 |  | rs1800775 | 16 | 55552736 | LDL small | fasting | A | C | 0.49 | 0.442 | 12156 | -69.00 | 6.00 | 9.40E-31 | 1.10 | 696 (3154) | 651 (6018) | 586 (2823) |
| 16q13 |  | rs1800775 | 16 | 55552736 | LDL mean size | fasting | A | C | 0.49 | 0.442 | 12156 | 0.12 | 0.01 | 1.00E-38 | 1.40 | 21 (3154) | 21 (6018) | 22 (2823) |
| 16q13 |  | rs1800775 | 16 | 55552736 | IDL total | fasting | A | C | 0.49 | 0.442 | 10510 | -0.11 | 0.01 | 4.50E-16 | 0.63 | 41 (3154) | 34 (6018) | 29 (2823) |
| 16q13 |  | rs1800775 | 16 | 55552736 | LDL total | fasting | A | C | 0.49 | 0.442 | 12156 | -0.03 | 0.00 | 1.20E-18 | 0.64 | 1318 (3154) | 1282 (6018) | 1226 (2823) |
| 16q13 |  | rs1800775 | 16 | 55552736 | HDL large | fasting | A | C | 0.49 | 0.442 | 12156 | 0.70 | 0.04 | 1.90E-59 | 2.20 | 7 (3154) | 8 (6018) | 8 (2823) |
| 16q13 |  | rs1800775 | 16 | 55552736 | HDL mean size | fasting | A | C | 0.49 | 0.442 | 12156 | 0.08 | 0.01 | 1.10E-53 | 1.90 | 9 (3154) | 9 (6018) | 9 (2823) |
| 16q13 |  | rs1800775 | 16 | 55552736 | VLDL total | fasting | A | C | 0.49 | 0.442 | 12156 | -2.40 | 0.40 | 2.90E-09 | 0.29 | 71 (3154) | 69 (6018) | 65 (2823) |
| 16q13 |  | rs1800775 | 16 | 55552736 | VLDL small | fasting | A | C | 0.49 | 0.442 | 12156 | -1.90 | 0.24 | 8.80E-15 | 0.49 | 47 (3154) | 46 (6018) | 42 (2823) |
| 16q13 |  | rs708272 | 16 | 55553788 | LDL total | all | A | G | 0.43 | 0.284 | 16843 | -0.04 | 0.00 | 1.90E-26 | 0.67 | 1313 (5424) | 1269 (8186) | 1212 (3008) |
| 16q13 |  | rs1864163 | 16 | 55554733 | LDL large | all | A | G | 0.25 | 0.266 | 16761 | -28.00 | 2.70 | 3.30E-25 | 0.64 | 555 (9154) | 527 (6329) | 500 (1054) |
| 16q13 |  | rs1864163 | 16 | 55554733 | LDL large | fasting | A | G | 0.25 | 0.266 | 12120 | -30.00 | 3.20 | 7.40E-22 | 0.76 | 562 (6595) | 532 (4589) | 500 (771) |
| 16q13 |  | rs1532624 | 16 | 55562979 | HDL-C by NMR | fasting | A | C | 0.43 | 0.572 | 11820 | 2.60 | 0.17 | 3.00E-55 | 2.10 | 51 (3811) | 54 (5660) | 56 (2182) |
| 16q13 |  | rs1532624 | 16 | 55562979 | HDL-C assay | fasting | A | C | 0.43 | 0.572 | 11793 | 3.10 | 0.18 | 1.30E-66 | 2.50 | 50 (3800) | 53 (5646) | 56 (2180) |
| 16q13 |  | rs1532624 | 16 | 55562979 | APOA1 assay | fasting | A | C | 0.43 | 0.572 | 11742 | 3.90 | 0.30 | 1.10E-39 | 1.50 | 146 (3782) | 150 (5624) | 154 (2171) |
| 16q13 |  | rs7499892 | 16 | 55564090 | HDL total | all | A | G | 0.18 | 0.960 | 16831 | -0.76 | 0.08 | 1.40E-20 | 0.51 | 35 (11226) | 34 (4853) | 34 (527) |
| 16q13 |  | rs7499892 | 16 | 55564090 | HDL total | fasting | A | G | 0.18 | 0.960 | 12170 | -0.74 | 0.10 | 1.70E-14 | 0.48 | 35 (8106) | 35 (3525) | 34 (375) |
| 17q24.2 | *WIPI1* | rs2909207 | 17 | 63951199 | HDL medium | all | A | G | 0.22 | 0.915 | 14704 | 0.09 | 0.02 | 1.00E-08 | 0.22 | 3 (10031) | 3 (5752) | 3 (829) |
| 18q21.1 | *LIPG* | rs8090363 | 18 | 45387825 | LDL large | fasting | A | G | 0.39 | 0.492 | 12102 | -16.00 | 2.80 | 2.00E-08 | 0.26 | 559 (4457) | 545 (5679) | 517 (1803) |
| 18q21.1 |  | rs4939883 | 18 | 45421211 | LDL large | all | A | G | 0.16 | 0.131 | 16680 | -19.00 | 3.10 | 2.60E-09 | 0.21 | 545 (11513) | 527 (4473) | 513 (474) |
| 18q21.1 |  | rs4939883 | 18 | 45421211 | LDL mean size | all | A | G | 0.16 | 0.131 | 16680 | -0.06 | 0.01 | 1.90E-08 | 0.19 | 21 (11513) | 21 (4473) | 21 (474) |
| 18q21.1 |  | rs4939883 | 18 | 45421211 | HDL large | all | A | G | 0.16 | 0.131 | 16680 | -0.31 | 0.05 | 4.00E-10 | 0.23 | 8 (11513) | 8 (4473) | 7 (474) |
| 18q21.1 |  | rs4939883 | 18 | 45421211 | HDL mean size | all | A | G | 0.16 | 0.131 | 16680 | -0.04 | 0.01 | 2.70E-09 | 0.21 | 9 (11513) | 9 (4473) | 9 (474) |
| 18q21.1 |  | rs4939883 | 18 | 45421211 | HDL-C by NMR | all | A | G | 0.16 | 0.131 | 16680 | -1.50 | 0.19 | 7.40E-16 | 0.39 | 54 (11513) | 53 (4473) | 50 (474) |
| 18q21.1 |  | rs4939883 | 18 | 45421211 | HDL-C assay | all | A | G | 0.16 | 0.131 | 16648 | -1.20 | 0.20 | 1.40E-09 | 0.22 | 53 (11488) | 52 (4467) | 49 (473) |
| 18q21.1 |  | rs4939883 | 18 | 45421211 | APOA1 assay | all | A | G | 0.16 | 0.131 | 16570 | -2.50 | 0.34 | 5.60E-14 | 0.34 | 151 (11433) | 148 (4450) | 145 (470) |
| 18q21.1 |  | rs4939883 | 18 | 45421211 | LDL mean size | fasting | A | G | 0.16 | 0.131 | 12057 | -0.07 | 0.01 | 6.10E-09 | 0.28 | 21 (8295) | 21 (3257) | 21 (345) |
| 18q21.1 |  | rs4939883 | 18 | 45421211 | HDL large | fasting | A | G | 0.16 | 0.131 | 12057 | -0.35 | 0.06 | 1.30E-09 | 0.31 | 8 (8295) | 7 (3257) | 6 (345) |
| 18q21.1 |  | rs4939883 | 18 | 45421211 | HDL mean size | fasting | A | G | 0.16 | 0.131 | 12057 | -0.04 | 0.01 | 1.90E-09 | 0.30 | 9 (8295) | 9 (3257) | 9 (345) |
| 18q21.1 |  | rs4939883 | 18 | 45421211 | HDL-C by NMR | fasting | A | G | 0.16 | 0.131 | 12057 | -1.60 | 0.22 | 5.30E-13 | 0.43 | 54 (8295) | 53 (3257) | 49 (345) |
| 18q21.1 |  | rs4939883 | 18 | 45421211 | HDL-C assay | fasting | A | G | 0.16 | 0.131 | 12030 | -1.30 | 0.24 | 1.90E-08 | 0.26 | 53 (8274) | 52 (3251) | 49 (345) |
| 18q21.1 |  | rs4939883 | 18 | 45421211 | APOA1 assay | fasting | A | G | 0.16 | 0.131 | 11976 | -2.60 | 0.39 | 2.40E-11 | 0.37 | 151 (8235) | 148 (3242) | 145 (341) |
| 19p13.2 | *LDLR* | rs6511720 | 19 | 11063305 | LDL large | all | A | C | 0.12 | 0.774 | 16843 | -28.00 | 3.60 | 4.30E-15 | 0.36 | 547 (12917) | 518 (3462) | 488 (239) |
| 19p13.2 |  | rs6511720 | 19 | 11063305 | LDL total | all | A | C | 0.12 | 0.774 | 16843 | -0.04 | 0.01 | 1.90E-13 | 0.32 | 1286 (12917) | 1229 (3462) | 1257 (239) |
| 19p13.2 |  | rs6511720 | 19 | 11063305 | LDL-C assay | all | A | C | 0.12 | 0.774 | 16810 | -6.50 | 0.56 | 2.30E-31 | 0.80 | 123 (12888) | 116 (3459) | 112 (238) |
| 19p13.2 |  | rs6511720 | 19 | 11063305 | APOB assay | all | A | C | 0.12 | 0.774 | 16726 | -4.50 | 0.44 | 4.80E-25 | 0.64 | 100 (12829) | 95 (3437) | 96 (238) |
| 19p13.2 |  | rs6511720 | 19 | 11063305 | VLDL small | all | A | C | 0.12 | 0.774 | 16843 | -2.00 | 0.31 | 1.60E-10 | 0.24 | 45 (12917) | 44 (3462) | 42 (239) |
| 19p13.2 |  | rs6511720 | 19 | 11063305 | LDL large | fasting | A | C | 0.12 | 0.774 | 12179 | -29.00 | 4.20 | 3.00E-12 | 0.40 | 553 (9285) | 524 (2561) | 488 (169) |
| 19p13.2 |  | rs6511720 | 19 | 11063305 | LDL total | fasting | A | C | 0.12 | 0.774 | 12179 | -0.04 | 0.01 | 2.10E-09 | 0.29 | 1287 (9285) | 1241 (2561) | 1211 (169) |
| 19p13.2 |  | rs6511720 | 19 | 11063305 | LDL-C assay | fasting | A | C | 0.12 | 0.774 | 12152 | -6.90 | 0.66 | 1.50E-25 | 0.90 | 124 (9261) | 118 (2559) | 111 (168) |
| 19p13.2 |  | rs6511720 | 19 | 11063305 | APOB assay | fasting | A | C | 0.12 | 0.774 | 12094 | -4.50 | 0.51 | 2.80E-18 | 0.63 | 103 (9221) | 96 (2543) | 95 (168) |
| 19p13.2 |  | rs6511720 | 19 | 11063305 | VLDL small | fasting | A | C | 0.12 | 0.774 | 12179 | -2.10 | 0.37 | 1.90E-08 | 0.26 | 46 (9285) | 44 (2561) | 42 (169) |
| 19q13.32 | *APOC1-APOE* | rs4803750 | 19 | 49939466 | LDL large | all | G | A | 0.07 | 0.006 | 16649 | -50.00 | 4.60 | 1.20E-26 | 0.68 | 548 (14338) | 489 (1989) | 399 (95) |
| 19q13.32 |  | rs4803750 | 19 | 49939466 | LDL-C assay | all | G | A | 0.07 | 0.006 | 16616 | -9.30 | 0.72 | 1.50E-37 | 0.98 | 123 (14306) | 113 (1988) | 105 (95) |
| 19q13.32 |  | rs4803750 | 19 | 49939466 | APOB assay | all | G | A | 0.07 | 0.006 | 16532 | -8.50 | 0.56 | 3.50E-51 | 1.40 | 100 (14236) | 92 (1978) | 81 (94) |
| 19q13.32 |  | rs4803750 | 19 | 49939466 | LDL large | fasting | G | A | 0.07 | 0.006 | 12037 | -49.00 | 5.50 | 2.40E-19 | 0.67 | 555 (10330) | 493 (1472) | 402 (67) |
| 19q13.32 |  | rs4803750 | 19 | 49939466 | LDL-C assay | fasting | G | A | 0.07 | 0.006 | 12010 | -9.30 | 0.85 | 1.30E-27 | 0.98 | 124 (10304) | 114 (1471) | 106 (67) |
| 19q13.32 |  | rs157580 | 19 | 50087105 | VLDL small | all | G | A | 0.39 | 0.436 | 16825 | -1.30 | 0.21 | 3.80E-10 | 0.23 | 46 (6224) | 45 (7858) | 43 (2520) |
| 19q13.32 |  | rs405509 | 19 | 50100675 | HDL medium | all | A | C | 0.48 | 0.712 | 14698 | -0.08 | 0.01 | 1.70E-10 | 0.28 | 3 (4466) | 3 (8283) | 2 (3854) |
| 19q13.32 |  | rs405509 | 19 | 50100675 | VLDL medium | all | A | C | 0.48 | 0.712 | 16827 | -0.92 | 0.17 | 2.70E-08 | 0.18 | 22 (4466) | 21 (8283) | 20 (3854) |
| 19q13.32 |  | rs405509 | 19 | 50100675 | HDL medium | fasting | A | C | 0.48 | 0.712 | 10613 | -0.10 | 0.02 | 2.50E-10 | 0.38 | 3 (3217) | 3 (6017) | 2 (2768) |
| 19q13.32 |  | rs769449 | 19 | 50101841 | LDL small | all | A | G | 0.12 | 0.568 | 16761 | 84.00 | 7.80 | 7.00E-27 | 0.69 | 635 (12899) | 713 (3413) | 736 (230) |
| 19q13.32 |  | rs769449 | 19 | 50101841 | LDL mean size | all | A | G | 0.12 | 0.568 | 16761 | -0.07 | 0.01 | 1.50E-09 | 0.22 | 21 (12899) | 21 (3413) | 21 (230) |
| 19q13.32 |  | rs769449 | 19 | 50101841 | LDL total | all | A | G | 0.12 | 0.568 | 16761 | 0.07 | 0.01 | 1.10E-44 | 1.20 | 1252 (12899) | 1355 (3413) | 1416 (230) |
| 19q13.32 |  | rs769449 | 19 | 50101841 | HDL-C by NMR | all | A | G | 0.12 | 0.568 | 16761 | -1.50 | 0.22 | 2.20E-12 | 0.29 | 54 (12899) | 52 (3413) | 52 (230) |
| 19q13.32 |  | rs769449 | 19 | 50101841 | HDL-C assay | all | A | G | 0.12 | 0.568 | 16728 | -1.60 | 0.24 | 2.20E-11 | 0.27 | 53 (12876) | 51 (3403) | 51 (230) |
| 19q13.32 |  | rs769449 | 19 | 50101841 | APOA1 assay | all | A | G | 0.12 | 0.568 | 16649 | -2.60 | 0.39 | 2.80E-11 | 0.27 | 151 (12810) | 148 (3393) | 146 (230) |
| 19q13.32 |  | rs769449 | 19 | 50101841 | LDL small | fasting | A | G | 0.12 | 0.568 | 12115 | 89.00 | 9.30 | 1.40E-21 | 0.75 | 631 (9347) | 708 (2444) | 719 (164) |
| 19q13.32 |  | rs769449 | 19 | 50101841 | LDL total | fasting | A | G | 0.12 | 0.568 | 12115 | 0.07 | 0.01 | 1.70E-33 | 1.20 | 1255 (9347) | 1365 (2444) | 1424 (164) |
| 19q13.32 |  | rs769449 | 19 | 50101841 | APOB assay | fasting | A | G | 0.12 | 0.568 | 12030 | 6.60 | 0.52 | 1.10E-36 | 1.30 | 98 (9280) | 107 (2428) | 114 (164) |
| 19q13.32 |  | rs769449 | 19 | 50101841 | HDL-C by NMR | fasting | A | G | 0.12 | 0.568 | 12115 | -1.70 | 0.26 | 2.40E-11 | 0.37 | 54 (9347) | 52 (2444) | 52 (164) |
| 19q13.32 |  | rs769449 | 19 | 50101841 | HDL-C assay | fasting | A | G | 0.12 | 0.568 | 12088 | -1.70 | 0.28 | 2.30E-10 | 0.33 | 53 (9328) | 51 (2436) | 51 (164) |
| 19q13.32 |  | rs769449 | 19 | 50101841 | APOA1 assay | fasting | A | G | 0.12 | 0.568 | 12032 | -3.10 | 0.45 | 1.10E-11 | 0.38 | 151 (9281) | 147 (2429) | 146 (164) |
| 19q13.32 |  | rs439401 | 19 | 50106290 | VLDL total | all | A | G | 0.35 | 0.809 | 16671 | -2.40 | 0.34 | 2.10E-12 | 0.30 | 71 (6639) | 68 (7570) | 65 (2238) |
| 19q13.32 |  | rs439401 | 19 | 50106290 | VLDL large | all | A | G | 0.35 | 0.809 | 16671 | -0.20 | 0.03 | 6.00E-10 | 0.23 | 2 (6639) | 1 (7570) | 1 (2238) |
| 19q13.32 |  | rs439401 | 19 | 50106290 | TG by NMR | all | A | G | 0.35 | 0.809 | 16671 | -0.04 | 0.00 | 7.30E-16 | 0.39 | 112 (6639) | 107 (7570) | 104 (2238) |
| 19q13.32 |  | rs439401 | 19 | 50106290 | TG assay | all | A | G | 0.35 | 0.809 | 16638 | -0.04 | 0.01 | 1.70E-15 | 0.38 | 122 (6620) | 116 (7560) | 112 (2234) |
| 19q13.32 |  | rs439401 | 19 | 50106290 | VLDL total | fasting | A | G | 0.35 | 0.809 | 12037 | -2.50 | 0.41 | 6.20E-10 | 0.32 | 70 (4806) | 68 (5452) | 64 (1618) |
| 19q13.32 |  | rs439401 | 19 | 50106290 | VLDL large | fasting | A | G | 0.35 | 0.809 | 12037 | -0.20 | 0.04 | 4.70E-08 | 0.25 | 2 (4806) | 1 (5452) | 1 (1618) |
| 19q13.32 |  | rs439401 | 19 | 50106290 | VLDL small | fasting | A | G | 0.35 | 0.809 | 12037 | -1.40 | 0.25 | 1.40E-08 | 0.27 | 46 (4806) | 45 (5452) | 43 (1618) |
| 19q13.32 |  | rs439401 | 19 | 50106290 | TG by NMR | fasting | A | G | 0.35 | 0.809 | 12037 | -0.04 | 0.01 | 3.60E-12 | 0.40 | 110 (4806) | 106 (5452) | 103 (1618) |
| 19q13.32 |  | rs439401 | 19 | 50106290 | TG assay | fasting | A | G | 0.35 | 0.809 | 12010 | -0.04 | 0.01 | 1.80E-11 | 0.38 | 117 (4791) | 112 (5443) | 109 (1615) |
| 20q13.12.A | *HNF4A* | rs1800961 | 20 | 42475777 | APOA1 assay | all | A | G | 0.42 | 0.820 | 16730 | -4.10 | 0.74 | 3.60E-08 | 0.18 | 150 (15545) | 147 (945) | 151 (18) |
| 20q13.12.B | *PLTP* | rs6065904 | 20 | 43968057 | HDL large | fasting | A | G | 0.45 | 0.765 | 12180 | -0.69 | 0.05 | 3.60E-40 | 1.40 | 8 (7273) | 7 (4130) | 6 (613) |
| 20q13.12.B |  | rs4810479 | 20 | 43978454 | LDL large | all | G | A | 0.05 | 0.876 | 16720 | -15.00 | 2.70 | 2.80E-08 | 0.18 | 548 (9157) | 529 (6245) | 526 (1095) |
| 20q13.12.B |  | rs4810479 | 20 | 43978454 | LDL large | fasting | G | A | 0.05 | 0.876 | 12085 | -18.00 | 3.20 | 1.20E-08 | 0.27 | 556 (6598) | 534 (4531) | 528 (793) |
| 20q13.12.B |  | rs4810479 | 20 | 43978454 | HDL small | fasting | G | A | 0.05 | 0.876 | 12085 | 1.20 | 0.08 | 1.00E-55 | 2.00 | 23 (6598) | 25 (4531) | 25 (793) |
| 20q13.12.B |  | rs6065906 | 20 | 43987421 | LDL small | all | G | A | 0.48 | 0.906 | 16843 | 47.00 | 6.40 | 3.90E-13 | 0.31 | 634 (10914) | 682 (5084) | 692 (620) |
| 20q13.12.B |  | rs6065906 | 20 | 43987421 | LDL mean size | all | G | A | 0.48 | 0.906 | 16843 | -0.08 | 0.01 | 1.30E-15 | 0.38 | 21 (10914) | 21 (5084) | 21 (620) |
| 20q13.12.B |  | rs6065906 | 20 | 43987421 | LDL total | all | G | A | 0.48 | 0.906 | 16843 | 0.02 | 0.00 | 4.40E-08 | 0.18 | 1261 (10914) | 1295 (5084) | 1320 (620) |
| 20q13.12.B |  | rs6065906 | 20 | 43987421 | HDL total | all | G | A | 0.48 | 0.906 | 16843 | 1.00 | 0.08 | 7.00E-38 | 0.98 | 34 (10914) | 36 (5084) | 36 (620) |
| 20q13.12.B |  | rs6065906 | 20 | 43987421 | HDL large | all | G | A | 0.48 | 0.906 | 16843 | -0.69 | 0.05 | 3.30E-49 | 1.30 | 8 (10914) | 7 (5084) | 6 (620) |
| 20q13.12.B |  | rs6065906 | 20 | 43987421 | HDL small | all | G | A | 0.48 | 0.906 | 16843 | 1.40 | 0.08 | 6.00E-79 | 2.10 | 23 (10914) | 24 (5084) | 26 (620) |
| 20q13.12.B |  | rs6065906 | 20 | 43987421 | HDL mean size | all | G | A | 0.48 | 0.906 | 16843 | -0.08 | 0.01 | 1.50E-45 | 1.20 | 9 (10914) | 9 (5084) | 9 (620) |
| 20q13.12.B |  | rs6065906 | 20 | 43987421 | HDL-C assay | all | G | A | 0.48 | 0.906 | 16810 | -1.50 | 0.19 | 1.80E-14 | 0.35 | 53 (10890) | 52 (5075) | 50 (620) |
| 20q13.12.B |  | rs6065906 | 20 | 43987421 | TG assay | all | G | A | 0.48 | 0.906 | 16810 | 0.04 | 0.01 | 5.00E-10 | 0.23 | 116 (10890) | 120 (5075) | 128 (620) |
| 20q13.12.B |  | rs6065906 | 20 | 43987421 | LDL small | fasting | G | A | 0.48 | 0.906 | 12179 | 53.00 | 7.60 | 3.10E-12 | 0.40 | 632 (7891) | 678 (3668) | 694 (456) |
| 20q13.12.B |  | rs6065906 | 20 | 43987421 | LDL mean size | fasting | G | A | 0.48 | 0.906 | 12179 | -0.09 | 0.01 | 1.00E-14 | 0.49 | 21 (7891) | 21 (3668) | 21 (456) |
| 20q13.12.B |  | rs6065906 | 20 | 43987421 | HDL total | fasting | G | A | 0.48 | 0.906 | 12179 | 1.00 | 0.09 | 3.20E-28 | 1.00 | 35 (7891) | 36 (3668) | 36 (456) |
| 20q13.12.B |  | rs6065906 | 20 | 43987421 | HDL mean size | fasting | G | A | 0.48 | 0.906 | 12179 | -0.09 | 0.01 | 8.30E-37 | 1.30 | 9 (7891) | 9 (3668) | 9 (456) |
| 20q13.12.B |  | rs6065906 | 20 | 43987421 | HDL-C assay | fasting | G | A | 0.48 | 0.906 | 12152 | -1.50 | 0.22 | 2.00E-11 | 0.37 | 53 (7873) | 52 (3659) | 50 (456) |
| 20q13.12.B |  | rs6065906 | 20 | 43987421 | TG assay | fasting | G | A | 0.48 | 0.906 | 12152 | 0.05 | 0.01 | 1.40E-08 | 0.26 | 112 (7873) | 115 (3659) | 126 (456) |

^a^A1, A2 = the minor and major alleles, respectively.

^b^MAF = minor allele frequency

^c^HWE p-value = significance of an exact test for Hardy-Weinberg equilibrium

^d^N = number of samples with genotype information for SNP

^e^BETA, SE, P = estimated mean, standard error, and significance of shift in adjusted lipoprotein fraction per copy of the minor allele

^f^R2 = proportion (%) variance explained by additive model
