# Supplementary material for: Forty-Three Loci Associated with Plasma Lipoprotein Size, Concentration, and Cholesterol Content in Genome-Wide Analysis
Source: PLoS Genet. 2009 Nov 20;5(11):e1000730. doi: 10.1371/journal.pgen.1000730 (PMC2777390; doi:10.1371/journal.pgen.1000730)
Supplement: Table S2 — Correlations between all pairs of lipoprotein fractions. (0.12 MB DOC) [file pgen.1000730.s006.doc]

Table S2. Spearman correlation coeffecients between lipoprotein fractions together with CRP in whole sample (lower left) and fasting sub-sample (upper right)

|  | LDL:L | LDL:S | LDL:Z | IDL | LDL:T | LDL-C | ApoB | HDL:T | HDL:L | HDL:M | HDL:S | HDL:Z | HDL:N | HDL-C | ApoA1 | VLDL:T | VLDL:L | VLDL:M | VLDL:S | VLDL:Z | TG:N | TG | CRP |
| --- | --- | --- | --- | --- | --- | --- | --- | --- | --- | --- | --- | --- | --- | --- | --- | --- | --- | --- | --- | --- | --- | --- | --- |
| LDL:L | - | -0.52 | 0.75 | -0.17 | -0.1 | 0.33 | 0.17 | 0.14 | 0.53 | -0.21 | -0.09 | 0.48 | 0.42 | 0.45 | 0.37 | -0.11 | -0.3 | -0.22 | 0.02 | -0.25 | -0.17 | -0.26 | -0.13 |
| LDL:S | -0.51 | - | -0.94 | 0.41 | 0.87 | 0.35 | 0.58 | 0.02 | -0.67 | 0.14 | 0.39 | -0.79 | -0.53 | -0.62 | -0.38 | 0.5 | 0.54 | 0.41 | 0.4 | 0.3 | 0.6 | 0.6 | 0.33 |
| LDL:Z | 0.74 | -0.93 | - | -0.38 | -0.68 | -0.14 | -0.37 | 0.04 | 0.7 | -0.16 | -0.32 | 0.78 | 0.56 | 0.63 | 0.43 | -0.41 | -0.51 | -0.38 | -0.29 | -0.31 | -0.5 | -0.53 | -0.3 |
| IDL | -0.16 | 0.4 | -0.37 | - | 0.5 | 0.34 | 0.44 | 0.14 | -0.24 | 0.25 | 0.15 | -0.35 | -0.13 | -0.21 | -0.01 | 0.3 | 0.43 | 0.23 | 0.26 | 0.23 | 0.48 | 0.41 | 0.27 |
| LDL:T | -0.07 | 0.86 | -0.66 | 0.48 | - | 0.62 | 0.83 | 0.11 | -0.5 | 0.06 | 0.4 | -0.66 | -0.38 | -0.48 | -0.23 | 0.55 | 0.51 | 0.4 | 0.5 | 0.25 | 0.66 | 0.61 | 0.34 |
| LDL-C | 0.35 | 0.34 | -0.12 | 0.33 | 0.62 | - | 0.79 | -0.01 | -0.25 | -0.15 | 0.25 | -0.32 | -0.24 | -0.07 | -0.09 | 0.51 | 0.15 | 0.23 | 0.62 | -0.14 | 0.4 | 0.3 | 0.09 |
| ApoB | 0.19 | 0.58 | -0.35 | 0.43 | 0.83 | 0.79 | - | 0.09 | -0.37 | -0.03 | 0.35 | -0.47 | -0.28 | -0.33 | -0.1 | 0.62 | 0.41 | 0.4 | 0.62 | 0.1 | 0.63 | 0.54 | 0.25 |
| HDL:T | 0.13 | 0.02 | 0.04 | 0.16 | 0.11 | 0 | 0.09 | - | 0.35 | 0.36 | 0.59 | 0.09 | 0.68 | 0.42 | 0.7 | 0.09 | 0.28 | 0.19 | -0.03 | 0.28 | 0.3 | 0.26 | 0.21 |
| HDL:L | 0.52 | -0.66 | 0.69 | -0.24 | -0.49 | -0.24 | -0.37 | 0.34 | - | -0.14 | -0.2 | 0.87 | 0.87 | 0.83 | 0.73 | -0.45 | -0.41 | -0.32 | -0.42 | -0.16 | -0.4 | -0.42 | -0.17 |
| HDL:M | -0.22 | 0.15 | -0.18 | 0.25 | 0.07 | -0.15 | -0.03 | 0.35 | -0.15 | - | -0.16 | -0.14 | 0.15 | -0.05 | 0.11 | 0.08 | 0.38 | 0.18 | -0.04 | 0.34 | 0.26 | 0.27 | 0.21 |
| HDL:S | -0.08 | 0.38 | -0.31 | 0.16 | 0.4 | 0.26 | 0.35 | 0.59 | -0.2 | -0.16 | - | -0.41 | 0.03 | -0.08 | 0.17 | 0.35 | 0.32 | 0.3 | 0.28 | 0.17 | 0.41 | 0.36 | 0.2 |
| HDL:Z | 0.47 | -0.79 | 0.77 | -0.34 | -0.65 | -0.32 | -0.47 | 0.08 | 0.87 | -0.15 | -0.41 | - | 0.75 | 0.76 | 0.58 | -0.53 | -0.52 | -0.41 | -0.47 | -0.24 | -0.54 | -0.54 | -0.27 |
| HDL:N | 0.41 | -0.53 | 0.56 | -0.12 | -0.37 | -0.23 | -0.28 | 0.68 | 0.87 | 0.14 | 0.03 | 0.74 | - | 0.83 | 0.88 | -0.33 | -0.18 | -0.17 | -0.36 | 0.03 | -0.18 | -0.21 | -0.05 |
| HDL-C | 0.44 | -0.62 | 0.63 | -0.19 | -0.47 | -0.06 | -0.32 | 0.41 | 0.83 | -0.06 | -0.08 | 0.76 | 0.83 | - | 0.78 | -0.41 | -0.38 | -0.3 | -0.37 | -0.17 | -0.38 | -0.39 | -0.17 |
| ApoA1 | 0.36 | -0.38 | 0.43 | 0 | -0.23 | -0.09 | -0.1 | 0.7 | 0.73 | 0.1 | 0.17 | 0.58 | 0.88 | 0.79 | - | -0.21 | -0.05 | -0.08 | -0.26 | 0.09 | -0.05 | -0.07 | 0.05 |
| VLDL:T | -0.1 | 0.49 | -0.4 | 0.28 | 0.54 | 0.51 | 0.61 | 0.09 | -0.44 | 0.09 | 0.34 | -0.52 | -0.32 | -0.4 | -0.21 | - | 0.53 | 0.82 | 0.87 | 0 | 0.83 | 0.67 | 0.16 |
| VLDL:L | -0.29 | 0.54 | -0.5 | 0.41 | 0.51 | 0.15 | 0.41 | 0.28 | -0.39 | 0.39 | 0.3 | -0.52 | -0.17 | -0.39 | -0.06 | 0.52 | - | 0.61 | 0.27 | 0.75 | 0.85 | 0.85 | 0.37 |
| VLDL:M | -0.2 | 0.41 | -0.37 | 0.21 | 0.39 | 0.23 | 0.39 | 0.18 | -0.32 | 0.18 | 0.29 | -0.4 | -0.16 | -0.3 | -0.09 | 0.82 | 0.61 | - | 0.47 | 0.21 | 0.84 | 0.7 | 0.17 |
| VLDL:S | 0.04 | 0.39 | -0.27 | 0.25 | 0.48 | 0.61 | 0.61 | -0.02 | -0.4 | -0.04 | 0.28 | -0.45 | -0.35 | -0.35 | -0.25 | 0.87 | 0.24 | 0.46 | - | -0.24 | 0.56 | 0.42 | 0.08 |
| VLDL:Z | -0.24 | 0.3 | -0.31 | 0.2 | 0.25 | -0.14 | 0.1 | 0.27 | -0.15 | 0.34 | 0.15 | -0.23 | 0.03 | -0.18 | 0.07 | 0 | 0.75 | 0.21 | -0.25 | - | 0.45 | 0.56 | 0.28 |
| TG:N | -0.16 | 0.6 | -0.5 | 0.45 | 0.66 | 0.39 | 0.63 | 0.3 | -0.39 | 0.28 | 0.39 | -0.54 | -0.18 | -0.38 | -0.06 | 0.81 | 0.85 | 0.83 | 0.53 | 0.47 | - | 0.89 | 0.32 |
| TG | -0.24 | 0.6 | -0.53 | 0.37 | 0.6 | 0.29 | 0.53 | 0.25 | -0.4 | 0.27 | 0.33 | -0.53 | -0.21 | -0.4 | -0.08 | 0.64 | 0.85 | 0.68 | 0.38 | 0.59 | 0.89 | - | 0.43 |
| CRP | -0.11 | 0.33 | -0.29 | 0.26 | 0.33 | 0.09 | 0.25 | 0.22 | -0.15 | 0.21 | 0.19 | -0.27 | -0.04 | -0.16 | 0.05 | 0.15 | 0.36 | 0.16 | 0.07 | 0.28 | 0.32 | 0.42 | - |
