# Supplementary material for: Forty-Three Loci Associated with Plasma Lipoprotein Size, Concentration, and Cholesterol Content in Genome-Wide Analysis
Source: PLoS Genet. 2009 Nov 20;5(11):e1000730. doi: 10.1371/journal.pgen.1000730 (PMC2777390; doi:10.1371/journal.pgen.1000730)
Supplement: Table S3 — Replication of WGHS candidate associations from whole sample in PROCARDIS and the Framingham Heart Study. (0.56 MB DOC) [file pgen.1000730.s007.doc]

Table S3. Replication of genomewide associations from whole WGHS sample in the FHS and PROCARDIS samples

|  |  |  | WGHS | | | FHS | | | | PROCARDIS | | | | combined |
| --- | --- | --- | --- | --- | --- | --- | --- | --- | --- | --- | --- | --- | --- | --- |
| Locus | lipid fraction | snp | beta^a^ | p^a^ | r2^a^ | N | beta^a^ | p^a^ | power^b^ | N | beta^a^ | p^a^ | power^b^ | p^c^ |
| 1p32.3 | APOB.assay | rs11591147 | -11.880 | 7.75E-28 | 0.71 | - | - | - | - | 2004 | -16.660 | 4.28E-02 | 0.97 | - |
| 1p32.3 | LDL-C.assay | rs11591147 | -15.520 | 1.29E-28 | 0.73 | - | - | - | - | 2008 | -0.657 | 3.44E-02 | 0.97 | - |
| 1p32.3 | LDL.total | rs11591147 | -0.121 | 7.05E-22 | 0.55 | - | - | - | - | 1962 | -0.148 | 1.74E-01 | 0.91 | - |
| 1p32.3 | LDL.large | rs11591147 | -65.710 | 2.24E-13 | 0.32 | - | - | - | - | 1928 | -119.800 | 1.30E-01 | 0.70 | - |
| 1p32.3 | TG.by.NMR | rs11591147 | -0.088 | 3.57E-08 | 0.18 | - | - | - | - | 1948 | -0.122 | 5.16E-01 | 0.47 | - |
| 1p32.3 | VLDL.total | rs11591147 | -7.734 | 1.14E-09 | 0.22 | - | - | - | - | 1974 | -14.960 | 2.45E-01 | 0.55 | - |
| 1p32.3 | VLDL.small | rs11591147 | -5.728 | 2.31E-13 | 0.32 | - | - | - | - | 1975 | -13.160 | 6.49E-02 | 0.71 | - |
| 1p31.3 | TG.by.NMR | rs10889353 | -0.033 | 1.19E-13 | 0.33 | 3229 | -0.052 | 5.47E-02 | 0.90 | 1948 | -0.068 | 1.12E-05 | 0.71 | 9.34E-06 |
| 1p31.3 | VLDL.total | rs10889353 | -2.688 | 1.34E-14 | 0.35 | 2742 | -0.100 | 5.43E-04 | 0.88 | 1974 | -5.472 | 1.79E-07 | 0.75 | 2.34E-09 |
| 1p31.3 | VLDL.medium | rs10889353 | -1.226 | 2.77E-12 | 0.29 | 2742 | -0.059 | 4.08E-02 | 0.81 | - | - | - | - | - |
| 1p31.3 | VLDL.small | rs10889353 | -1.323 | 7.35E-10 | 0.23 | 2742 | -0.105 | 2.67E-04 | 0.70 | 1975 | -2.273 | 9.07E-05 | 0.56 | 4.49E-07 |
| 1p13.3 | APOB.assay | rs646776 | -5.205 | 1.77E-53 | 1.41 | 2821 | -0.192 | 2.15E-08 | 1.00 | 2004 | -3.544 | 4.31E-06 | 1.00 | 2.87E-12 |
| 1p13.3 | LDL-C.assay | rs646776 | -6.182 | 4.97E-46 | 1.20 | 7344 | -0.233 | 2.00E-25 | 1.00 | 2008 | -0.095 | 1.13E-03 | 1.00 | 0.00E+00 |
| 1p13.3 | LDL.total | rs646776 | -0.042 | 2.42E-27 | 0.70 | 2742 | -0.171 | 6.31E-07 | 0.99 | 1962 | -0.030 | 3.71E-03 | 0.96 | 4.88E-08 |
| 1p13.3 | LDL.large | rs646776 | -16.210 | 6.05E-09 | 0.20 | 2742 | -0.095 | 5.94E-03 | 0.65 | 1928 | -16.470 | 2.96E-02 | 0.50 | 1.69E-03 |
| 1p13.3 | LDL.small | rs646776 | -41.260 | 9.25E-12 | 0.28 | 2742 | -0.086 | 1.25E-02 | 0.79 | 1974 | -31.800 | 2.78E-02 | 0.65 | 3.12E-03 |
| 1p13.3 | VLDL.total | rs646776 | -2.575 | 7.11E-11 | 0.25 | 2742 | -0.006 | 8.51E-01 | 0.75 | 1974 | 0.167 | 8.91E-01 | 0.61 | - |
| 1p13.3 | VLDL.small | rs646776 | -2.159 | 6.29E-19 | 0.47 | 2742 | -0.098 | 4.56E-03 | 0.95 | 1975 | 0.081 | 9.05E-01 | 0.86 | - |
| 1q23.3 | HDL.medium | rs4073054 | -0.087 | 5.75E-11 | 0.29 | 2742 | -0.051 | 7.32E-02 | 0.81 | 1772 | -0.152 | 4.45E-04 | 0.62 | 3.69E-04 |
| 2p24.1 | APOB.assay | rs506585 | -4.093 | 2.84E-31 | 0.81 | 2821 | -0.130 | 9.20E-05 | 1.00 | 2004 | -3.757 | 5.45E-06 | 0.98 | 1.12E-08 |
| 2p24.1 | LDL-C.assay | rs1367117 | 4.027 | 6.46E-25 | 0.63 | 7344 | 0.142 | 3.69E-13 | 1.00 | 2008 | 0.105 | 4.81E-05 | 0.95 | 6.66E-16 |
| 2p24.1 | LDL.total | rs506585 | -0.040 | 1.55E-22 | 0.57 | 2742 | -0.162 | 1.33E-06 | 0.98 | 1962 | -0.040 | 3.24E-04 | 0.92 | 9.69E-09 |
| 2p24.1 | LDL.large | rs1713222 | -23.810 | 1.84E-13 | 0.32 | 2742 | -0.136 | 1.98E-04 | 0.85 | 1928 | -17.860 | 4.77E-02 | 0.70 | 1.19E-04 |
| 2p24.1 | LDL.mean.size | rs673548 | 0.052 | 4.40E-08 | 0.18 | - | - | - | - | - | - | - | - | - |
| 2p24.1 | TG.by.NMR | rs673548 | -0.045 | 1.74E-18 | 0.46 | 3229 | -0.087 | 7.30E-03 | 0.97 | 1948 | -0.048 | 6.60E-03 | 0.85 | 5.27E-04 |
| 2p24.1 | TG.assay | rs673548 | -0.041 | 4.28E-10 | 0.23 | 7423 | -0.106 | 1.83E-06 | 0.99 | 1948 | -0.041 | 2.36E-02 | 0.57 | 7.76E-07 |
| 2p24.1 | VLDL.total | rs676210 | -6.384 | 8.61E-56 | 1.46 | 2742 | -0.124 | 3.09E-04 | 1.00 | 1974 | -5.728 | 1.35E-06 | 1.00 | 9.40E-09 |
| 2p24.1 | VLDL.medium | rs673548 | -1.996 | 1.61E-22 | 0.56 | 2742 | -0.096 | 5.98E-03 | 0.98 | 2004 | -2.308 | 1.06E-03 | 0.92 | 8.20E-05 |
| 2p24.1 | VLDL.small | rs676210 | -4.219 | 3.75E-64 | 1.68 | 2742 | -0.110 | 1.41E-03 | 1.00 | 1975 | -2.938 | 9.12E-06 | 1.00 | 2.46E-07 |
| 2p24.1 | VLDL.mean.size | rs676210 | 0.642 | 2.05E-10 | 0.24 | 2742 | 0.020 | 5.70E-01 | 0.73 | 2030 | 0.664 | 7.49E-02 | 0.60 | 1.77E-01 |
| 2p23.3 | APOA1.assay | rs1260326 | 1.695 | 2.87E-11 | 0.27 | 2885 | 0.051 | 5.91E-02 | 0.79 | 2018 | 1.272 | 8.25E-02 | 0.64 | 3.08E-02 |
| 2p23.3 | APOB.assay | rs780094 | 2.465 | 1.07E-17 | 0.44 | 2821 | 0.063 | 1.94E-02 | 0.94 | 2004 | 1.646 | 1.26E-02 | 0.84 | 2.27E-03 |
| 2p23.3 | HDL.total | rs1260326 | 0.794 | 6.34E-36 | 0.93 | 2742 | 0.054 | 5.29E-02 | 1.00 | 2031 | 0.365 | 2.37E-02 | 0.99 | 9.63E-03 |
| 2p23.3 | HDL.small | rs1260326 | 0.565 | 8.39E-21 | 0.52 | 2742 | -0.033 | 2.26E-01 | 0.97 | 2024 | 0.359 | 2.11E-02 | 0.90 | - |
| 2p23.3 | HDL.mean.size | rs1260326 | -0.029 | 7.21E-10 | 0.23 | - | - | - | - | - | - | - | - | - |
| 2p23.3 | IDL.total | rs780094 | 0.072 | 6.98E-10 | 0.26 | 2742 | -0.009 | 7.40E-01 | 0.77 | 1765 | 0.060 | 8.56E-02 | 0.58 | - |
| 2p23.3 | LDL.total | rs780094 | 0.030 | 3.79E-19 | 0.47 | 2742 | 0.029 | 2.98E-01 | 0.95 | 1962 | 0.016 | 7.17E-02 | 0.86 | 1.03E-01 |
| 2p23.3 | LDL.small | rs780094 | 46.100 | 2.62E-19 | 0.48 | 2742 | 0.031 | 2.54E-01 | 0.95 | 1974 | 32.310 | 8.68E-03 | 0.87 | 1.57E-02 |
| 2p23.3 | LDL.mean.size | rs780094 | -0.054 | 3.34E-12 | 0.29 | 2742 | -0.004 | 8.87E-01 | 0.80 | 2011 | -0.059 | 5.38E-03 | 0.67 | 3.03E-02 |
| 2p23.3 | TG.by.NMR | rs1260326 | 0.052 | 2.93E-35 | 0.92 | 3229 | 0.123 | 1.82E-06 | 1.00 | 1948 | 0.065 | 1.88E-05 | 0.99 | 8.59E-10 |
| 2p23.3 | TG.assay | rs1260326 | 0.070 | 1.03E-37 | 0.98 | 7423 | 0.119 | 2.86E-11 | 1.00 | 1948 | 0.079 | 4.32E-07 | 0.99 | 4.44E-16 |
| 2p23.3 | VLDL.large | rs1260326 | 0.342 | 2.79E-28 | 0.73 | 2742 | 0.120 | 1.51E-05 | 0.99 | 1992 | 0.866 | 4.08E-06 | 0.97 | 1.51E-09 |
| 2p23.3 | VLDL.total | rs1260326 | 2.796 | 7.78E-17 | 0.42 | - | - | - | - | - | - | - | - | - |
| 2p23.3 | VLDL.medium | rs1260326 | 1.420 | 3.67E-17 | 0.42 | 2742 | 0.086 | 1.98E-03 | 0.93 | - | - | - | - | - |
| 2p23.3 | VLDL.mean.size | rs1260326 | 0.671 | 7.60E-16 | 0.39 | 2742 | 0.108 | 9.80E-05 | 0.90 | 2030 | 0.875 | 6.94E-03 | 0.80 | 1.03E-05 |
| 2p21 | APOB.assay | rs11887534 | -3.574 | 3.48E-10 | 0.24 | 2821 | -0.177 | 3.03E-02 | 0.73 | - | - | - | - | - |
| 2p21 | LDL-C.assay | rs11887534 | -4.864 | 2.78E-11 | 0.26 | 7344 | -0.231 | 1.35E-05 | 0.99 | - | - | - | - | - |
| 2q24.3 | HDL-C.assay | rs10490694 | 1.349 | 4.78E-09 | 0.20 | 7423 | 0.030 | 2.63E-01 | 0.97 | 2049 | -0.010 | 4.70E-01 | 0.53 | - |
| 3q22.3 | HDL.small | rs3856637 | 0.371 | 1.28E-08 | 0.19 | 2742 | 0.056 | 6.01E-02 | 0.63 | 2024 | 0.097 | 5.58E-01 | 0.50 | 1.47E-01 |
| 5q13.3 | LDL-C.assay | rs3846662 | 2.589 | 1.61E-12 | 0.30 | 7344 | 0.071 | 1.13E-04 | 1.00 | 2008 | 0.044 | 6.98E-02 | 0.69 | 1.00E-04 |
| 5q13.3 | LDL.large | rs5744680 | 13.860 | 8.76E-09 | 0.20 | 2742 | 0.056 | 5.47E-02 | 0.64 | 1928 | 13.540 | 3.38E-02 | 0.50 | 1.35E-02 |
| 6p21.32 | TG.by.NMR | rs2076530 | 0.025 | 2.41E-09 | 0.21 | 3229 | 0.010 | 6.97E-01 | 0.74 | 1948 | 0.017 | 2.57E-01 | 0.53 | 4.87E-01 |
| 6p21.32 | VLDL.large | rs2076530 | 0.170 | 3.43E-08 | 0.18 | - | - | - | - | - | - | - | - | - |
| 7q11.23 | HDL.small | rs11974409 | -0.443 | 5.71E-09 | 0.20 | - | - | - | - | - | - | - | - | - |
| 7q11.23 | LDL.large | rs714052 | 19.950 | 2.98E-08 | 0.18 | 2742 | 0.077 | 7.51E-02 | 0.61 | 1928 | 27.270 | 4.20E-03 | 0.47 | 2.86E-03 |
| 7q11.23 | LDL.small | rs11974409 | -40.960 | 2.60E-10 | 0.24 | 2742 | -0.069 | 5.02E-02 | 0.72 | 1974 | -33.650 | 2.71E-02 | 0.58 | 1.03E-02 |
| 7q11.23 | LDL.mean.size | rs7777102 | 0.076 | 3.54E-11 | 0.26 | 2742 | 0.096 | 4.69E-02 | 0.76 | 2011 | 0.079 | 1.02E-02 | 0.63 | 4.12E-03 |
| 7q11.23 | TG.by.NMR | rs11974409 | -0.042 | 2.10E-15 | 0.37 | 3229 | -0.110 | 8.85E-04 | 0.94 | 1948 | -0.001 | 9.46E-01 | 0.77 | 6.77E-03 |
| 7q11.23 | TG.assay | rs11974409 | -0.061 | 1.49E-18 | 0.46 | 7423 | -0.118 | 3.28E-07 | 1.00 | 1948 | 0.002 | 9.18E-01 | 0.85 | - |
| 7q11.23 | VLDL.large | rs11974409 | -0.227 | 6.29E-09 | 0.20 | 2742 | -0.095 | 7.75E-03 | 0.65 | 1992 | -0.179 | 4.42E-01 | 0.52 | 2.29E-02 |
| 7q11.23 | VLDL.total | rs714052 | -3.262 | 1.58E-10 | 0.24 | 2742 | -0.115 | 7.76E-03 | 0.73 | 1974 | -0.599 | 6.97E-01 | 0.59 | 3.37E-02 |
| 7q11.23 | VLDL.medium | rs11974409 | -1.405 | 3.83E-11 | 0.26 | 2742 | -0.124 | 4.44E-04 | 0.76 | 2004 | -0.949 | 2.13E-01 | 0.63 | 9.69E-04 |
| 7q32.2 | HDL.mean.size | rs4731702 | 0.027 | 3.93E-09 | 0.21 | 2742 | 0.038 | 1.71E-01 | 0.66 | 1998 | 0.006 | 6.14E-01 | 0.53 | 3.42E-01 |
| 7q32.2 | LDL.total | rs4731702 | -0.019 | 6.60E-09 | 0.20 | 2742 | -0.015 | 5.91E-01 | 0.65 | 1962 | -0.017 | 4.28E-02 | 0.51 | 1.18E-01 |
| 7q32.2 | LDL.small | rs4731702 | -30.490 | 1.51E-09 | 0.22 | 2742 | -0.020 | 4.55E-01 | 0.69 | 1974 | -12.270 | 3.00E-01 | 0.55 | 4.08E-01 |
| 7q32.2 | TG.assay | rs4731702 | -0.032 | 2.24E-09 | 0.21 | 7423 | -0.054 | 2.19E-03 | 0.98 | 1948 | 0.003 | 8.39E-01 | 0.53 | - |
| 8p21.3 | APOA1.assay | rs331 | 1.798 | 1.54E-10 | 0.24 | 2885 | 0.074 | 1.44E-02 | 0.76 | 2018 | 1.778 | 2.98E-02 | 0.60 | 3.76E-03 |
| 8p21.3 | HDL-C.assay | rs331 | 1.459 | 1.22E-17 | 0.43 | 7423 | 0.148 | 2.01E-13 | 1.00 | 2049 | 0.029 | 7.34E-03 | 0.85 | 5.17E-14 |
| 8p21.3 | HDL.large | rs331 | 0.343 | 1.52E-16 | 0.40 | 2742 | 0.116 | 1.94E-04 | 0.92 | 2013 | 0.164 | 1.16E-01 | 0.81 | 2.64E-04 |
| 8p21.3 | HDL.mean.size | rs331 | 0.039 | 2.51E-14 | 0.34 | 2742 | 0.089 | 4.21E-03 | 0.87 | 1998 | 0.019 | 1.78E-01 | 0.75 | 6.15E-03 |
| 8p21.3 | LDL.large | rs2083637 | 18.080 | 4.98E-12 | 0.28 | 2742 | 0.056 | 6.96E-02 | 0.80 | 1928 | 13.430 | 6.31E-02 | 0.65 | 2.82E-02 |
| 8p21.3 | LDL.small | rs331 | -44.920 | 2.46E-15 | 0.37 | 2742 | -0.038 | 2.27E-01 | 0.89 | 1974 | -22.740 | 9.73E-02 | 0.77 | 1.06E-01 |
| 8p21.3 | LDL.mean.size | rs2083637 | 0.069 | 1.36E-15 | 0.38 | 2742 | 0.067 | 3.14E-02 | 0.90 | - | - | - | - | - |
| 8p21.3 | HDL-C.by.NMR | rs331 | 1.210 | 1.10E-14 | 0.35 | 3225 | 0.151 | 1.44E-07 | 0.92 | 2037 | 0.634 | 1.18E-01 | 0.77 | 3.20E-07 |
| 8p21.3 | TG.by.NMR | rs328 | -0.070 | 1.35E-25 | 0.65 | 3229 | -0.141 | 1.22E-03 | 1.00 | - | - | - | - | - |
| 8p21.3 | TG.assay | rs328 | -0.092 | 2.37E-26 | 0.67 | - | - | - | - | - | - | - | - | - |
| 8p21.3 | VLDL.large | rs328 | -0.390 | 2.80E-15 | 0.37 | 2742 | -0.064 | 1.68E-01 | 0.89 | - | - | - | - | - |
| 8p21.3 | VLDL.total | rs328 | -5.780 | 2.24E-27 | 0.70 | 2742 | -0.164 | 3.72E-04 | 0.99 | - | - | - | - | - |
| 8p21.3 | VLDL.medium | rs328 | -3.062 | 2.91E-30 | 0.77 | 2742 | -0.179 | 1.09E-04 | 1.00 | - | - | - | - | - |
| 8p21.3 | VLDL.small | rs328 | -2.328 | 1.36E-12 | 0.30 | 2742 | -0.019 | 6.80E-01 | 0.82 | - | - | - | - | - |
| 8q24.13 | APOB.assay | rs6982636 | -1.950 | 7.45E-12 | 0.28 | 2821 | -0.056 | 3.84E-02 | 0.80 | 2004 | -2.761 | 1.37E-05 | 0.66 | 8.14E-06 |
| 8q24.13 | LDL.total | rs6982636 | -0.023 | 2.40E-12 | 0.29 | 2742 | -0.061 | 2.71E-02 | 0.81 | 1962 | -0.036 | 2.39E-05 | 0.67 | 9.89E-06 |
| 8q24.13 | LDL.small | rs10808546 | -35.910 | 2.38E-12 | 0.30 | 2742 | -0.068 | 1.60E-02 | 0.82 | 1974 | -34.330 | 4.00E-03 | 0.68 | 6.82E-04 |
| 8q24.13 | LDL.mean.size | rs10808546 | 0.045 | 5.20E-09 | 0.21 | 2742 | 0.067 | 1.86E-02 | 0.66 | 2011 | 0.039 | 6.12E-02 | 0.53 | 8.87E-03 |
| 8q24.13 | TG.by.NMR | rs6982636 | -0.026 | 8.46E-10 | 0.22 | 3229 | -0.069 | 6.73E-03 | 0.77 | 1948 | -0.052 | 4.33E-04 | 0.55 | 4.01E-05 |
| 8q24.13 | TG.assay | rs6982636 | -0.033 | 1.20E-09 | 0.22 | 7423 | -0.101 | 1.36E-08 | 0.98 | 1948 | -0.045 | 2.76E-03 | 0.54 | 9.35E-10 |
| 9q31.1 | HDL-C.assay | rs2515614 | 0.896 | 2.35E-08 | 0.19 | 7423 | -0.002 | 9.24E-01 | 0.96 | 2049 | -0.001 | 9.51E-01 | 0.50 | - |
| 9q31.1 | HDL.medium | rs3905000 | 0.104 | 2.76E-08 | 0.21 | - | - | - | - | - | - | - | - | - |
| 9q34.2 | LDL-C.assay | rs507666 | 3.299 | 5.31E-13 | 0.31 | - | - | - | - | - | - | - | - | - |
| 9q34.2 | LDL.large | rs507666 | 17.850 | 1.04E-09 | 0.22 | - | - | - | - | - | - | - | - | - |
| 9q34.2 | VLDL.small | rs507666 | 1.869 | 2.49E-13 | 0.32 | - | - | - | - | - | - | - | - | - |
| 9q34.2 | VLDL.mean.size | rs507666 | -0.564 | 4.40E-08 | 0.18 | - | - | - | - | - | - | - | - | - |
| 11q12.2 | HDL.large | rs102275 | -0.299 | 1.45E-14 | 0.35 | 2742 | -0.080 | 7.14E-03 | 0.87 | 2013 | -0.373 | 9.41E-05 | 0.76 | 1.02E-05 |
| 11q12.2 | HDL.medium | rs174537 | 0.102 | 7.57E-14 | 0.38 | 2742 | 0.085 | 4.24E-03 | 0.90 | 1772 | 0.052 | 2.32E-01 | 0.74 | 7.80E-03 |
| 11q12.2 | HDL.mean.size | rs1535 | -0.034 | 3.54E-12 | 0.29 | 2742 | -0.070 | 1.89E-02 | 0.80 | - | - | - | - | - |
| 11q12.2 | LDL.large | rs1535 | -15.020 | 1.00E-09 | 0.22 | 2742 | -0.051 | 8.79E-02 | 0.69 | 1928 | -5.670 | 3.93E-01 | 0.54 | 1.51E-01 |
| 11q23.3 | APOA1.assay | rs12225230 | 3.234 | 6.41E-23 | 0.58 | 2885 | 0.055 | 1.21E-01 | 0.98 | 2018 | 2.609 | 6.71E-03 | 0.93 | 6.59E-03 |
| 11q23.3 | APOB.assay | rs3135506 | 5.808 | 2.28E-22 | 0.56 | - | - | - | - | - | - | - | - | - |
| 11q23.3 | HDL-C.assay | rs618923 | 1.224 | 2.44E-12 | 0.29 | 7423 | 0.020 | 3.38E-01 | 1.00 | 2049 | 0.011 | 3.18E-01 | 0.69 | 3.47E-01 |
| 11q23.3 | HDL.total | rs518181 | 0.610 | 3.88E-21 | 0.53 | 2742 | 0.005 | 8.56E-01 | 0.97 | 2031 | 0.372 | 2.34E-02 | 0.91 | 9.85E-02 |
| 11q23.3 | HDL.small | rs518181 | 0.681 | 1.48E-28 | 0.73 | 2742 | 0.031 | 2.76E-01 | 0.99 | 2024 | 0.197 | 2.14E-01 | 0.97 | 2.26E-01 |
| 11q23.3 | LDL.total | rs3135506 | 0.061 | 1.15E-18 | 0.46 | - | - | - | - | - | - | - | - | - |
| 11q23.3 | LDL.small | rs3135506 | 105.100 | 5.66E-23 | 0.58 | - | - | - | - | - | - | - | - | - |
| 11q23.3 | LDL.mean.size | rs3135506 | -0.130 | 7.36E-16 | 0.39 | - | - | - | - | - | - | - | - | - |
| 11q23.3 | HDL-C.by.NMR | rs12225230 | 1.418 | 8.95E-15 | 0.36 | 3225 | 0.039 | 2.41E-01 | 0.92 | 2037 | 0.661 | 1.65E-01 | 0.77 | 1.68E-01 |
| 11q23.3 | TG.by.NMR | rs3135506 | 0.119 | 7.77E-42 | 1.09 | - | - | - | - | - | - | - | - | - |
| 11q23.3 | TG.assay | rs3135506 | 0.142 | 4.05E-36 | 0.93 | - | - | - | - | - | - | - | - | - |
| 11q23.3 | VLDL.large | rs3135506 | 0.575 | 3.55E-19 | 0.47 | - | - | - | - | - | - | - | - | - |
| 11q23.3 | VLDL.total | rs3135506 | 9.526 | 7.04E-43 | 1.11 | - | - | - | - | - | - | - | - | - |
| 11q23.3 | VLDL.medium | rs3135506 | 4.965 | 5.06E-46 | 1.20 | - | - | - | - | - | - | - | - | - |
| 11q23.3 | VLDL.small | rs3135506 | 3.988 | 1.11E-20 | 0.52 | - | - | - | - | - | - | - | - | - |
| 12q23.2 | HDL.total | rs1818702 | -0.421 | 9.42E-10 | 0.22 | - | - | - | - | - | - | - | - | - |
| 12q23.2 | HDL-C.by.NMR | rs10778213 | -0.787 | 1.76E-08 | 0.19 | 3225 | 0.032 | 2.33E-01 | 0.69 | 2037 | 0.214 | 5.49E-01 | 0.50 | - |
| 12q24.31.A | LDL-C.assay | rs1169300 | 2.404 | 1.45E-09 | 0.22 | 7344 | 0.042 | 2.59E-02 | 0.98 | 2008 | 0.031 | 2.35E-01 | 0.55 | 3.72E-02 |
| 12q24.31.B | HDL.large | rs7307277 | 0.257 | 4.86E-11 | 0.26 | 2742 | -0.018 | 5.28E-01 | 0.76 | 2013 | 0.235 | 1.33E-02 | 0.62 | - |
| 12q24.31.B | HDL.mean.size | rs7307277 | 0.031 | 2.91E-10 | 0.24 | 2742 | 0.013 | 6.44E-01 | 0.72 | 1998 | 0.015 | 2.35E-01 | 0.58 | 4.37E-01 |
| 12q24.31.B | LDL.total | rs7307277 | -0.020 | 1.09E-08 | 0.19 | 2742 | 0.015 | 5.99E-01 | 0.64 | 1962 | -0.009 | 3.37E-01 | 0.50 | - |
| 12q24.31.B | LDL.small | rs7307277 | -33.120 | 5.16E-10 | 0.23 | 2742 | 0.011 | 6.97E-01 | 0.71 | 1974 | -12.020 | 3.34E-01 | 0.57 | - |
| 12q24.31.B | LDL.mean.size | rs7307277 | 0.046 | 1.05E-08 | 0.19 | 2742 | 0.024 | 4.02E-01 | 0.64 | 2011 | 0.045 | 3.82E-02 | 0.51 | 7.94E-02 |
| 12q24.31.B | TG.assay | rs7307277 | -0.035 | 6.10E-10 | 0.23 | 7423 | 0.002 | 9.04E-01 | 0.98 | 1948 | -0.014 | 3.90E-01 | 0.56 | - |
| 15q22.1 | APOA1.assay | rs1800588 | 3.829 | 8.23E-37 | 0.96 | 2885 | 0.133 | 4.71E-05 | 1.00 | - | - | - | - | - |
| 15q22.1 | HDL-C.assay | rs1800588 | 1.821 | 3.63E-23 | 0.58 | 7423 | 0.120 | 1.98E-08 | 1.00 | - | - | - | - | - |
| 15q22.1 | HDL.large | rs1800588 | 0.853 | 1.22E-81 | 2.15 | 2742 | 0.124 | 1.92E-04 | 1.00 | - | - | - | - | - |
| 15q22.1 | HDL.medium | rs1800588 | -0.099 | 3.41E-10 | 0.27 | 2742 | -0.028 | 4.04E-01 | 0.77 | - | - | - | - | - |
| 15q22.1 | HDL.small | rs1532085 | -0.511 | 6.79E-17 | 0.41 | - | - | - | - | - | - | - | - | - |
| 15q22.1 | HDL.mean.size | rs1800588 | 0.097 | 8.04E-69 | 1.81 | 2742 | 0.135 | 4.84E-05 | 1.00 | - | - | - | - | - |
| 15q22.1 | IDL.total | rs1532085 | 0.107 | 1.45E-19 | 0.57 | 2742 | 0.102 | 3.84E-04 | 0.98 | 1765 | 0.148 | 1.23E-05 | 0.89 | 9.54E-08 |
| 15q22.1 | LDL.large | rs1800588 | 44.160 | 9.37E-56 | 1.46 | 2742 | 0.086 | 1.01E-02 | 1.00 | - | - | - | - | - |
| 15q22.1 | LDL.small | rs1800588 | -45.290 | 1.28E-13 | 0.33 | 2742 | -0.115 | 5.74E-04 | 0.85 | - | - | - | - | - |
| 15q22.1 | LDL.mean.size | rs1800588 | 0.119 | 1.49E-37 | 0.97 | 2742 | 0.077 | 2.08E-02 | 1.00 | - | - | - | - | - |
| 15q22.1 | HDL-C.by.NMR | rs1800588 | 2.281 | 7.92E-42 | 1.08 | 3225 | 0.109 | 4.36E-04 | 1.00 | - | - | - | - | - |
| 16q13 | APOA1.assay | rs1800775 | 4.062 | 1.35E-59 | 1.58 | 2885 | -0.176 | 4.34E-08 | 1.00 | 2018 | 2.749 | 8.13E-05 | 1.00 | - |
| 16q13 | HDL-C.assay | rs1800775 | 3.096 | 3.28E-93 | 2.47 | 7423 | -0.244 | 5.78E-32 | 1.00 | 2049 | 0.054 | 3.68E-09 | 1.00 | - |
| 16q13 | HDL.total | rs7499892 | -0.761 | 1.43E-20 | 0.51 | 2742 | -0.299 | 1.84E-08 | 0.96 | 2031 | -0.724 | 2.15E-04 | 0.90 | 1.08E-10 |
| 16q13 | HDL.large | rs1800775 | 0.729 | 4.96E-87 | 2.30 | 2742 | -0.209 | 1.74E-10 | 1.00 | 2013 | 0.573 | 1.44E-10 | 1.00 | - |
| 16q13 | HDL.mean.size | rs1800775 | 0.086 | 7.95E-78 | 2.05 | 2742 | -0.157 | 1.79E-06 | 1.00 | 1998 | 0.065 | 4.78E-08 | 1.00 | - |
| 16q13 | IDL.total | rs1800775 | -0.103 | 2.79E-19 | 0.56 | 2742 | 0.166 | 4.05E-07 | 0.98 | 1765 | -0.086 | 9.89E-03 | 0.88 | - |
| 16q13 | LDL.total | rs708272 | -0.035 | 1.87E-26 | 0.67 | - | - | - | - | - | - | - | - | - |
| 16q13 | LDL.large | rs1864163 | -27.870 | 3.30E-25 | 0.64 | 2742 | -0.159 | 8.17E-05 | 0.99 | 1928 | -25.860 | 2.40E-04 | 0.94 | 3.68E-07 |
| 16q13 | LDL.small | rs1800775 | -68.570 | 4.50E-42 | 1.09 | 2742 | 0.039 | 2.31E-01 | 1.00 | 1974 | -33.650 | 4.11E-03 | 1.00 | - |
| 16q13 | LDL.mean.size | rs1800775 | 0.115 | 4.31E-51 | 1.34 | 2742 | -0.129 | 9.29E-05 | 1.00 | 2011 | 0.067 | 9.48E-04 | 1.00 | - |
| 16q13 | HDL-C.by.NMR | rs1800775 | 2.658 | 1.37E-81 | 2.15 | 3225 | -0.234 | 9.92E-15 | 1.00 | 2037 | 1.941 | 1.90E-08 | 1.00 | - |
| 16q13 | TG.assay | rs1800775 | -0.029 | 4.27E-08 | 0.18 | 7423 | 0.028 | 1.88E-01 | 0.95 | 1948 | 0.006 | 6.90E-01 | 0.46 | - |
| 16q13 | VLDL.total | rs1800775 | -2.490 | 4.56E-14 | 0.34 | 2742 | 0.014 | 6.74E-01 | 0.86 | 1974 | -2.142 | 3.03E-02 | 0.73 | - |
| 16q13 | VLDL.small | rs1800775 | -1.861 | 4.74E-20 | 0.50 | 2742 | 0.064 | 5.17E-02 | 0.96 | 1975 | -1.714 | 1.78E-03 | 0.88 | - |
| 17q24.2.B | HDL.medium | rs2909207 | 0.089 | 1.02E-08 | 0.22 | 2742 | 0.061 | 6.02E-02 | 0.70 | 1772 | 0.047 | 3.48E-01 | 0.51 | 1.02E-01 |
| 18q21.1 | APOA1.assay | rs4939883 | -2.529 | 5.58E-14 | 0.34 | 2885 | -0.190 | 1.98E-07 | 0.88 | 2018 | -2.498 | 5.92E-03 | 0.75 | 2.52E-08 |
| 18q21.1 | HDL-C.assay | rs4939883 | -1.238 | 1.40E-09 | 0.22 | 7423 | -0.147 | 7.08E-10 | 0.98 | 2049 | -0.032 | 6.39E-03 | 0.57 | 1.23E-10 |
| 18q21.1 | HDL.large | rs4939883 | -0.312 | 4.02E-10 | 0.23 | 2742 | -0.151 | 5.51E-05 | 0.72 | 2013 | -0.364 | 1.76E-03 | 0.58 | 1.66E-06 |
| 18q21.1 | HDL.mean.size | rs4939883 | -0.037 | 2.72E-09 | 0.21 | 2742 | -0.124 | 9.21E-04 | 0.67 | 1998 | -0.036 | 2.00E-02 | 0.54 | 2.19E-04 |
| 18q21.1 | LDL.large | rs4939883 | -18.640 | 2.60E-09 | 0.21 | 2742 | -0.013 | 7.33E-01 | 0.68 | 1928 | -3.354 | 6.77E-01 | 0.53 | 8.44E-01 |
| 18q21.1 | LDL.mean.size | rs4939883 | -0.058 | 1.87E-08 | 0.19 | 2742 | -0.058 | 1.20E-01 | 0.63 | 2011 | -0.037 | 1.67E-01 | 0.50 | 9.85E-02 |
| 18q21.1 | HDL-C.by.NMR | rs4939883 | -1.513 | 7.37E-16 | 0.39 | 3225 | -0.151 | 1.25E-05 | 0.94 | 2037 | -1.550 | 5.49E-04 | 0.81 | 1.36E-07 |
| 19p13.2 | APOB.assay | rs6511720 | -4.516 | 4.84E-25 | 0.64 | 2821 | -0.205 | 3.00E-03 | 0.99 | 2004 | -3.975 | 1.45E-04 | 0.95 | 6.78E-06 |
| 19p13.2 | LDL-C.assay | rs6511720 | -6.532 | 2.27E-31 | 0.80 | 7344 | -0.262 | 4.39E-09 | 1.00 | 2008 | -0.151 | 1.32E-04 | 0.98 | 1.69E-11 |
| 19p13.2 | LDL.total | rs6511720 | -0.037 | 1.88E-13 | 0.32 | 2742 | -0.178 | 1.17E-02 | 0.84 | 1962 | -0.044 | 1.72E-03 | 0.71 | 2.37E-04 |
| 19p13.2 | LDL.large | rs6511720 | -28.200 | 4.27E-15 | 0.37 | 2742 | -0.080 | 2.57E-01 | 0.89 | 1928 | -26.340 | 1.08E-02 | 0.76 | 1.90E-02 |
| 19p13.2 | VLDL.small | rs6511720 | -2.005 | 1.62E-10 | 0.24 | 2742 | 0.007 | 9.21E-01 | 0.73 | 1975 | -2.004 | 2.83E-02 | 0.59 | - |
| 19q13.32 | APOA1.assay | rs769449 | -2.584 | 2.85E-11 | 0.27 | - | - | - | - | - | - | - | - | - |
| 19q13.32 | APOB.assay | rs4803750 | -8.495 | 3.46E-51 | 1.36 | 2821 | -0.327 | 3.42E-05 | 1.00 | 2004 | -5.509 | 1.95E-05 | 1.00 | 1.48E-08 |
| 19q13.32 | HDL-C.assay | rs769449 | -1.581 | 2.19E-11 | 0.27 | - | - | - | - | - | - | - | - | - |
| 19q13.32 | HDL.medium | rs405509 | -0.083 | 1.73E-10 | 0.28 | 2742 | -0.052 | 3.21E-01 | 0.79 | 1772 | -0.176 | 2.25E-05 | 0.60 | 9.26E-05 |
| 19q13.32 | LDL-C.assay | rs4803750 | -9.284 | 1.48E-37 | 0.98 | 7344 | -0.285 | 1.41E-08 | 1.00 | 2008 | -0.217 | 8.11E-06 | 0.99 | 3.52E-12 |
| 19q13.32 | LDL.total | rs769449 | 0.071 | 1.10E-44 | 1.17 | - | - | - | - | - | - | - | - | - |
| 19q13.32 | LDL.large | rs4803750 | -49.740 | 1.25E-26 | 0.68 | 2742 | -0.184 | 2.38E-02 | 0.99 | 1928 | -38.250 | 2.32E-03 | 0.95 | 5.98E-04 |
| 19q13.32 | LDL.small | rs769449 | 84.200 | 7.00E-27 | 0.69 | - | - | - | - | - | - | - | - | - |
| 19q13.32 | LDL.mean.size | rs769449 | -0.072 | 1.49E-09 | 0.22 | - | - | - | - | - | - | - | - | - |
| 19q13.32 | HDL-C.by.NMR | rs769449 | -1.521 | 2.25E-12 | 0.29 | - | - | - | - | - | - | - | - | - |
| 19q13.32 | TG.by.NMR | rs439401 | -0.035 | 7.32E-16 | 0.39 | 3229 | -0.103 | 7.11E-02 | 0.94 | 1948 | -0.045 | 3.25E-03 | 0.79 | 2.17E-03 |
| 19q13.32 | TG.assay | rs439401 | -0.044 | 1.72E-15 | 0.38 | 7423 | -0.059 | 1.35E-01 | 1.00 | 1948 | -0.043 | 6.57E-03 | 0.78 | 7.12E-03 |
| 19q13.32 | VLDL.large | rs439401 | -0.196 | 5.94E-10 | 0.23 | 2742 | -0.141 | 2.06E-02 | 0.71 | 1992 | -0.451 | 1.66E-02 | 0.57 | 3.07E-03 |
| 19q13.32 | VLDL.total | rs439401 | -2.402 | 2.09E-12 | 0.30 | 2742 | -0.087 | 1.51E-01 | 0.81 | 1974 | -2.011 | 5.33E-02 | 0.68 | 4.70E-02 |
| 19q13.32 | VLDL.medium | rs405509 | -0.920 | 2.74E-08 | 0.18 | - | - | - | - | - | - | - | - | - |
| 19q13.32 | VLDL.small | rs157580 | -1.299 | 3.79E-10 | 0.23 | 2742 | -0.004 | 9.29E-01 | 0.72 | 1975 | -0.725 | 2.14E-01 | 0.57 | 5.19E-01 |
| 20q13.12.A | APOA1.assay | rs1800961 | -4.059 | 3.56E-08 | 0.18 | 2885 | -0.108 | 4.22E-01 | 0.63 | 2018 | -3.903 | 3.15E-02 | 0.48 | 7.07E-02 |
| 20q13.12.B | HDL-C.assay | rs6065906 | -1.481 | 1.82E-14 | 0.35 | 7423 | -0.073 | 1.99E-03 | 1.00 | 2049 | -0.012 | 3.14E-01 | 0.76 | 5.24E-03 |
| 20q13.12.B | HDL.total | rs6065906 | 1.020 | 7.05E-38 | 0.98 | 2742 | -0.054 | 1.46E-01 | 1.00 | 2031 | 1.008 | 2.64E-07 | 0.99 | - |
| 20q13.12.B | HDL.large | rs6065906 | -0.693 | 3.35E-49 | 1.28 | 2742 | -0.179 | 1.24E-06 | 1.00 | 2013 | -0.552 | 1.39E-06 | 1.00 | 4.84E-11 |
| 20q13.12.B | HDL.small | rs6065906 | 1.413 | 6.02E-79 | 2.08 | 2742 | -0.088 | 1.75E-02 | 1.00 | 2024 | 1.438 | 2.10E-14 | 1.00 | - |
| 20q13.12.B | HDL.mean.size | rs6065906 | -0.083 | 1.47E-45 | 1.19 | 2742 | -0.154 | 2.89E-05 | 1.00 | 1998 | -0.062 | 5.12E-05 | 1.00 | 3.16E-08 |
| 20q13.12.B | LDL.total | rs6065906 | 0.023 | 4.40E-08 | 0.18 | - | - | - | - | - | - | - | - | - |
| 20q13.12.B | LDL.large | rs4810479 | -14.840 | 2.83E-08 | 0.18 | 2742 | -0.057 | 8.90E-02 | 0.61 | 1928 | -12.600 | 7.54E-02 | 0.47 | 4.03E-02 |
| 20q13.12.B | LDL.small | rs6065906 | 46.670 | 3.86E-13 | 0.31 | 2742 | 0.035 | 3.45E-01 | 0.83 | 1974 | 21.100 | 1.61E-01 | 0.70 | 2.16E-01 |
| 20q13.12.B | LDL.mean.size | rs6065906 | -0.078 | 1.28E-15 | 0.38 | 2742 | -0.062 | 9.29E-02 | 0.90 | 2011 | -0.060 | 2.20E-02 | 0.79 | 1.47E-02 |
| 20q13.12.B | TG.assay | rs6065906 | 0.042 | 5.00E-10 | 0.23 | 7423 | 0.066 | 5.29E-03 | 0.99 | 1948 | 0.026 | 1.83E-01 | 0.56 | 7.66E-03 |

^a^Beta coefficient, significance, proportion variance explained in regression model testing association of the does of the minor allele of SNPs with indicated lipoprotein fractions after adjustment for clinical covariates (see Methods).

^b^Available power given the effect estimate in the WGHS sample and the sample size in either the FHS or PROCARDIS cohorts

^c^P-value estimated by Fisher’s method for combined FHS and PROCARDIS cohorts when beta coefficients had same sign
