# Supplementary material for: Forty-Three Loci Associated with Plasma Lipoprotein Size, Concentration, and Cholesterol Content in Genome-Wide Analysis
Source: PLoS Genet. 2009 Nov 20;5(11):e1000730. doi: 10.1371/journal.pgen.1000730 (PMC2777390; doi:10.1371/journal.pgen.1000730)
Supplement: Table S4 — Replication of WGHS candidate associations from fasting sub-sample in PROCARDIS and the Framingham Heart Study. (0.45 MB DOC) [file pgen.1000730.s008.doc]

Table S4. Replication of genomewide associations from fasting WGHS subsample in the FHS and PROCARDIS samples

|  |  |  | WGHS | | | FHS | | | | PROCARDIS | | | | combined |
| --- | --- | --- | --- | --- | --- | --- | --- | --- | --- | --- | --- | --- | --- | --- |
| locus | lipid fraction | snp | beta^a^ | p^a^ | r2^a^ | N | locus | lipid fraction | snp | beta^a^ | p^a^ | r2^a^ | N | locus |
| 1p32.3 | APOB.assay | rs11591147 | -12.490 | 5.34E-23 | 0.80 | - | - | - | - | 1817 | -16.770 | 4.35E-02 | 0.97 | - |
| 1p32.3 | LDL-C.assay | rs11591147 | -16.280 | 1.34E-23 | 0.82 | - | - | - | - | 1805 | -0.665 | 3.36E-02 | 0.97 | - |
| 1p32.3 | LDL.total | rs11591147 | -0.125 | 8.20E-18 | 0.61 | - | - | - | - | 1791 | -0.147 | 1.78E-01 | 0.91 | - |
| 1p32.3 | LDL.large | rs11591147 | -66.990 | 1.15E-10 | 0.34 | - | - | - | - | 1744 | -120.400 | 1.27E-01 | 0.68 | - |
| 1p32.3 | VLDL.total | rs11591147 | -8.299 | 3.04E-08 | 0.25 | - | - | - | - | 1779 | -15.080 | 2.48E-01 | 0.56 | - |
| 1p32.3 | VLDL.small | rs11591147 | -6.343 | 4.59E-12 | 0.39 | - | - | - | - | 1755 | -13.440 | 6.30E-02 | 0.75 | - |
| 1p31.3 | TG.by.NMR | rs10889353 | -0.035 | 1.65E-11 | 0.37 | 3229 | -0.052 | 5.47E-02 | 0.93 | 1791 | -0.073 | 3.34E-06 | 0.73 | 3.01E-06 |
| 1p31.3 | VLDL.total | rs10889353 | -2.973 | 1.41E-12 | 0.41 | 2742 | -0.100 | 5.43E-04 | 0.92 | 1779 | -5.416 | 1.35E-06 | 0.77 | 1.62E-08 |
| 1p31.3 | VLDL.medium | rs10889353 | -1.320 | 3.01E-10 | 0.33 | 2742 | -0.059 | 4.08E-02 | 0.85 | 1807 | -3.127 | 1.11E-06 | 0.68 | 8.08E-07 |
| 1p31.3 | VLDL.small | rs1167998 | -1.505 | 4.13E-09 | 0.28 | 2742 | -0.103 | 3.29E-04 | 0.80 | 1755 | -2.139 | 6.03E-04 | 0.61 | 3.26E-06 |
| 1p13.3 | APOB.assay | rs646776 | -5.251 | 3.71E-39 | 1.41 | 2821 | -0.192 | 2.15E-08 | 1.00 | 1817 | -3.540 | 1.46E-05 | 1.00 | 9.36E-12 |
| 1p13.3 | LDL-C.assay | rs646776 | -6.114 | 1.23E-32 | 1.16 | 7344 | -0.233 | 2.00E-25 | 1.00 | 1805 | -0.098 | 1.44E-03 | 1.00 | 0.00E+00 |
| 1p13.3 | LDL.total | rs646776 | -0.043 | 2.25E-20 | 0.70 | 2742 | -0.171 | 6.31E-07 | 0.99 | 1791 | -0.028 | 8.23E-03 | 0.94 | 1.04E-07 |
| 1p13.3 | LDL.small | rs646776 | -41.970 | 5.01E-09 | 0.28 | 2742 | -0.086 | 1.25E-02 | 0.79 | 1821 | -26.480 | 7.84E-02 | 0.62 | 7.79E-03 |
| 1p13.3 | VLDL.small | rs646776 | -2.142 | 1.60E-13 | 0.45 | 2742 | -0.098 | 4.56E-03 | 0.94 | 1755 | -0.370 | 6.09E-01 | 0.80 | 1.91E-02 |
| 1q23.3 | HDL.medium | rs4073054 | -0.088 | 2.02E-08 | 0.30 | 2742 | -0.051 | 7.32E-02 | 0.81 | 1591 | -0.132 | 3.62E-03 | 0.58 | 2.45E-03 |
| 2p24.1 | APOB.assay | rs506585 | -4.355 | 1.23E-25 | 0.90 | 2821 | -0.130 | 9.20E-05 | 1.00 | 1817 | -4.127 | 2.13E-06 | 0.98 | 4.59E-09 |
| 2p24.1 | LDL-C.assay | rs1367117 | 3.916 | 2.11E-17 | 0.59 | 7344 | 0.142 | 3.69E-13 | 1.00 | 1805 | 0.128 | 3.63E-06 | 0.91 | 1.11E-16 |
| 2p24.1 | LDL.total | rs312985 | -0.041 | 1.18E-17 | 0.60 | 2742 | -0.161 | 1.46E-06 | 0.98 | 1791 | -0.046 | 6.55E-05 | 0.91 | 2.31E-09 |
| 2p24.1 | LDL.large | rs1713222 | -24.840 | 7.08E-11 | 0.35 | 2742 | -0.136 | 1.98E-04 | 0.87 | 1744 | -12.650 | 1.85E-01 | 0.70 | 4.10E-04 |
| 2p24.1 | TG.by.NMR | rs673548 | -0.046 | 1.75E-14 | 0.48 | 3229 | -0.087 | 7.30E-03 | 0.98 | 1791 | -0.049 | 6.35E-03 | 0.84 | 5.09E-04 |
| 2p24.1 | TG.assay | rs676210 | -0.047 | 1.51E-09 | 0.30 | 7423 | -0.105 | 1.65E-06 | 1.00 | 1809 | -0.045 | 1.59E-02 | 0.65 | 4.83E-07 |
| 2p24.1 | VLDL.total | rs673548 | -6.624 | 4.81E-42 | 1.51 | 2742 | -0.125 | 3.31E-04 | 1.00 | 1779 | -6.073 | 1.86E-06 | 1.00 | 1.37E-08 |
| 2p24.1 | VLDL.medium | rs673548 | -2.181 | 4.03E-19 | 0.65 | 2742 | -0.096 | 5.98E-03 | 0.99 | 1807 | -2.238 | 2.40E-03 | 0.93 | 1.74E-04 |
| 2p24.1 | VLDL.small | rs6754295 | -4.127 | 3.92E-47 | 1.69 | 2742 | -0.096 | 4.33E-03 | 1.00 | 1755 | -3.134 | 6.30E-06 | 1.00 | 5.02E-07 |
| 2p24.1 | VLDL.mean.size | rs676210 | 0.663 | 1.00E-08 | 0.27 | 2742 | 0.020 | 5.70E-01 | 0.78 | 1830 | 0.661 | 8.00E-02 | 0.60 | 1.86E-01 |
| 2p23.3 | APOA1.assay | rs1260326 | 1.756 | 3.43E-09 | 0.29 | 2885 | 0.051 | 5.91E-02 | 0.83 | 1824 | 1.174 | 1.31E-01 | 0.64 | 4.53E-02 |
| 2p23.3 | APOB.assay | rs780094 | 2.488 | 2.67E-13 | 0.44 | 2821 | 0.063 | 1.94E-02 | 0.94 | 1817 | 1.464 | 3.53E-02 | 0.81 | 5.67E-03 |
| 2p23.3 | HDL.total | rs1260326 | 0.817 | 8.06E-28 | 0.99 | 2742 | 0.054 | 5.29E-02 | 1.00 | 1831 | 0.347 | 4.07E-02 | 0.99 | 1.54E-02 |
| 2p23.3 | HDL.small | rs780094 | 0.594 | 3.10E-17 | 0.58 | 2742 | -0.023 | 4.04E-01 | 0.98 | 1856 | 0.290 | 6.97E-02 | 0.91 | - |
| 2p23.3 | HDL.mean.size | rs780094 | -0.033 | 1.72E-09 | 0.30 | 2742 | 0.019 | 4.86E-01 | 0.82 | 1833 | -0.021 | 1.17E-01 | 0.65 | - |
| 2p23.3 | IDL.total | rs780094 | 0.074 | 4.55E-08 | 0.28 | 2742 | -0.009 | 7.40E-01 | 0.80 | - | - | - | - | - |
| 2p23.3 | LDL.total | rs780094 | 0.032 | 2.87E-16 | 0.55 | 2742 | 0.029 | 2.98E-01 | 0.97 | 1791 | 0.013 | 1.45E-01 | 0.88 | 1.79E-01 |
| 2p23.3 | LDL.small | rs780094 | 52.130 | 8.60E-18 | 0.60 | 2742 | 0.031 | 2.54E-01 | 0.98 | 1821 | 27.540 | 3.18E-02 | 0.91 | 4.70E-02 |
| 2p23.3 | LDL.mean.size | rs780094 | -0.066 | 9.82E-13 | 0.42 | 2742 | -0.004 | 8.87E-01 | 0.92 | 1820 | -0.053 | 1.78E-02 | 0.79 | 8.13E-02 |
| 2p23.3 | TG.by.NMR | rs1260326 | 0.056 | 3.48E-29 | 1.04 | 3229 | 0.123 | 1.82E-06 | 1.00 | 1791 | 0.056 | 3.26E-04 | 0.99 | 1.32E-08 |
| 2p23.3 | TG.assay | rs1260326 | 0.075 | 4.45E-32 | 1.15 | 7423 | 0.119 | 2.86E-11 | 1.00 | 1809 | 0.064 | 5.09E-05 | 1.00 | 5.13E-14 |
| 2p23.3 | VLDL.large | rs1260326 | 0.362 | 3.61E-24 | 0.85 | 2742 | 0.120 | 1.51E-05 | 1.00 | 1799 | 0.519 | 2.17E-03 | 0.98 | 5.97E-07 |
| 2p23.3 | VLDL.total | rs780094 | 2.961 | 1.56E-13 | 0.45 | 2742 | 0.075 | 5.99E-03 | 0.94 | 1779 | 3.353 | 2.47E-03 | 0.81 | 1.79E-04 |
| 2p23.3 | VLDL.medium | rs1260326 | 1.566 | 6.10E-15 | 0.50 | 2742 | 0.086 | 1.98E-03 | 0.96 | 1807 | 1.981 | 1.84E-03 | 0.86 | 4.93E-05 |
| 2p23.3 | VLDL.mean.size | rs1260326 | 0.715 | 6.23E-14 | 0.47 | 2742 | 0.108 | 9.80E-05 | 0.95 | 1830 | 0.818 | 1.20E-02 | 0.83 | 1.73E-05 |
| 2p21 | LDL-C.assay | rs4299376 | 2.595 | 2.23E-08 | 0.26 | 7344 | 0.152 | 9.74E-14 | 0.99 | 1805 | 0.055 | 4.37E-02 | 0.58 | 1.45E-13 |
| 5q13.3 | LDL-C.assay | rs3846662 | 2.707 | 3.57E-10 | 0.32 | 7344 | 0.071 | 1.13E-04 | 1.00 | 1805 | 0.048 | 5.72E-02 | 0.68 | 8.37E-05 |
| 5q13.3 | LDL.large | rs5744680 | 16.670 | 3.68E-09 | 0.29 | 2742 | 0.056 | 5.47E-02 | 0.80 | 1744 | 11.280 | 8.82E-02 | 0.61 | 3.05E-02 |
| 6p21.32 | TG.by.NMR | rs3129882 | -0.030 | 1.70E-09 | 0.30 | 3229 | 0.001 | 9.74E-01 | 0.87 | 1791 | -0.023 | 1.28E-01 | 0.64 | - |
| 6p21.32 | VLDL.large | rs3129882 | -0.196 | 3.63E-08 | 0.25 | - | - | - | - | - | - | - | - | - |
| 7q11.23 | TG.by.NMR | rs11974409 | -0.041 | 8.45E-11 | 0.35 | 3229 | -0.110 | 8.85E-04 | 0.92 | 1791 | -0.002 | 9.31E-01 | 0.70 | 6.68E-03 |
| 7q11.23 | TG.assay | rs11974409 | -0.058 | 3.21E-13 | 0.44 | 7423 | -0.118 | 3.28E-07 | 1.00 | 1809 | 0.003 | 8.73E-01 | 0.80 | - |
| 7q11.23 | VLDL.medium | rs2240466 | -1.691 | 3.29E-08 | 0.25 | - | - | - | - | - | - | - | - | - |
| 8p23.1 | VLDL.mean.size | rs983309 | 0.869 | 6.38E-09 | 0.28 | 2742 | 0.020 | 6.87E-01 | 0.79 | 1830 | 0.736 | 1.32E-01 | 0.61 | 3.08E-01 |
| 8p21.3 | APOA1.assay | rs331 | 1.945 | 3.06E-09 | 0.29 | 2885 | 0.074 | 1.44E-02 | 0.83 | 1824 | 2.186 | 1.16E-02 | 0.63 | 1.62E-03 |
| 8p21.3 | HDL-C.assay | rs331 | 1.510 | 2.72E-14 | 0.48 | 7423 | 0.148 | 2.01E-13 | 1.00 | 1854 | 0.034 | 2.51E-03 | 0.84 | 1.82E-14 |
| 8p21.3 | HDL.large | rs331 | 0.369 | 3.55E-14 | 0.47 | 2742 | 0.116 | 1.94E-04 | 0.95 | 1836 | 0.187 | 9.22E-02 | 0.84 | 2.14E-04 |
| 8p21.3 | HDL.mean.size | rs331 | 0.041 | 2.11E-11 | 0.37 | 2742 | 0.089 | 4.21E-03 | 0.89 | 1833 | 0.023 | 1.16E-01 | 0.74 | 4.20E-03 |
| 8p21.3 | LDL.large | rs2083637 | 18.890 | 9.37E-10 | 0.31 | 2742 | 0.056 | 6.96E-02 | 0.83 | 1744 | 15.370 | 4.21E-02 | 0.64 | 2.00E-02 |
| 8p21.3 | LDL.small | rs2083637 | -46.950 | 2.99E-12 | 0.40 | - | - | - | - | - | - | - | - | - |
| 8p21.3 | LDL.mean.size | rs2083637 | 0.071 | 6.26E-12 | 0.39 | 2742 | 0.067 | 3.14E-02 | 0.90 | 1820 | 0.053 | 3.25E-02 | 0.76 | 8.06E-03 |
| 8p21.3 | HDL-C.by.NMR | rs331 | 1.256 | 1.02E-11 | 0.38 | 3225 | 0.151 | 1.44E-07 | 0.94 | 1839 | 0.821 | 5.69E-02 | 0.75 | 1.60E-07 |
| 8p21.3 | TG.by.NMR | rs1059611 | -0.065 | 1.65E-17 | 0.59 | 3229 | -0.137 | 1.26E-03 | 0.99 | 1791 | -0.091 | 2.37E-04 | 0.90 | 4.77E-06 |
| 8p21.3 | TG.assay | rs2083637 | -0.063 | 2.52E-19 | 0.66 | 7423 | -0.187 | 2.82E-21 | 1.00 | 1809 | -0.059 | 7.89E-04 | 0.93 | 0.00E+00 |
| 8p21.3 | VLDL.large | rs1059611 | -0.391 | 1.17E-12 | 0.41 | 2742 | -0.073 | 1.08E-01 | 0.92 | 1799 | -0.785 | 3.47E-03 | 0.78 | 3.33E-03 |
| 8p21.3 | VLDL.total | rs328 | -5.566 | 1.81E-18 | 0.63 | 2742 | -0.164 | 3.72E-04 | 0.99 | - | - | - | - | - |
| 8p21.3 | VLDL.medium | rs1059611 | -2.875 | 1.48E-20 | 0.71 | 2742 | -0.179 | 7.02E-05 | 0.99 | 1807 | -4.585 | 6.65E-06 | 0.95 | 1.05E-08 |
| 8p21.3 | VLDL.small | rs328 | -2.234 | 9.24E-09 | 0.27 | 2742 | -0.019 | 6.80E-01 | 0.78 | - | - | - | - | - |
| 8q24.13 | APOB.assay | rs6982636 | -2.103 | 4.23E-10 | 0.32 | 2821 | -0.056 | 3.84E-02 | 0.86 | 1817 | -2.773 | 4.27E-05 | 0.68 | 2.35E-05 |
| 8q24.13 | LDL.total | rs6982636 | -0.026 | 1.18E-11 | 0.38 | 2742 | -0.061 | 2.71E-02 | 0.90 | 1791 | -0.037 | 2.93E-05 | 0.74 | 1.20E-05 |
| 8q24.13 | LDL.small | rs6982636 | -37.190 | 5.96E-10 | 0.32 | - | - | - | - | - | - | - | - | - |
| 9q31.1 | APOA1.assay | rs2740486 | -1.744 | 2.82E-09 | 0.29 | 2885 | -0.009 | 7.36E-01 | 0.83 | 1824 | -1.130 | 1.41E-01 | 0.64 | 3.38E-01 |
| 9q31.1 | HDL-C.assay | rs2515614 | 1.031 | 3.40E-08 | 0.25 | 7423 | -0.002 | 9.24E-01 | 0.99 | - | - | - | - | - |
| 9q34.2 | LDL-C.assay | rs507666 | 3.314 | 7.65E-10 | 0.31 | - | - | - | - | - | - | - | - | - |
| 9q34.2 | VLDL.small | rs507666 | 1.894 | 4.55E-10 | 0.32 | - | - | - | - | - | - | - | - | - |
| 11q12.2 | HDL.large | rs174546 | -0.296 | 9.77E-11 | 0.34 | 2742 | -0.081 | 6.53E-03 | 0.87 | 1836 | -0.392 | 1.00E-04 | 0.71 | 9.97E-06 |
| 11q12.2 | HDL.medium | rs174537 | 0.094 | 4.50E-09 | 0.32 | 2742 | 0.085 | 4.24E-03 | 0.85 | 1591 | 0.043 | 3.52E-01 | 0.62 | 1.12E-02 |
| 11q12.2 | HDL.mean.size | rs1535 | -0.033 | 1.03E-08 | 0.27 | 2742 | -0.070 | 1.89E-02 | 0.78 | 1833 | -0.032 | 1.71E-02 | 0.60 | 2.93E-03 |
| 11q12.2 | LDL.large | rs1535 | -15.840 | 4.35E-08 | 0.25 | 2742 | -0.051 | 8.79E-02 | 0.74 | 1744 | -10.000 | 1.49E-01 | 0.55 | 6.97E-02 |
| 11q23.3 | APOA1.assay | rs12225230 | 3.253 | 1.87E-17 | 0.60 | 2885 | 0.055 | 1.21E-01 | 0.99 | 1824 | 3.480 | 6.35E-04 | 0.91 | 8.05E-04 |
| 11q23.3 | APOB.assay | rs3135506 | 5.224 | 1.82E-13 | 0.45 | - | - | - | - | - | - | - | - | - |
| 11q23.3 | HDL-C.assay | rs618923 | 1.204 | 3.71E-09 | 0.29 | 7423 | 0.020 | 3.38E-01 | 1.00 | 1854 | 0.004 | 7.04E-01 | 0.64 | 5.79E-01 |
| 11q23.3 | HDL.total | rs518181 | 0.615 | 8.14E-16 | 0.53 | 2742 | 0.005 | 8.56E-01 | 0.97 | 1831 | 0.481 | 5.64E-03 | 0.88 | 3.06E-02 |
| 11q23.3 | HDL.small | rs518181 | 0.675 | 6.80E-21 | 0.72 | 2742 | 0.031 | 2.76E-01 | 0.99 | 1856 | 0.333 | 4.27E-02 | 0.96 | 6.42E-02 |
| 11q23.3 | LDL.total | rs3135506 | 0.056 | 8.26E-12 | 0.38 | - | - | - | - | - | - | - | - | - |
| 11q23.3 | LDL.small | rs3135506 | 101.100 | 1.38E-15 | 0.52 | - | - | - | - | - | - | - | - | - |
| 11q23.3 | LDL.mean.size | rs3135506 | -0.132 | 7.16E-12 | 0.39 | - | - | - | - | - | - | - | - | - |
| 11q23.3 | HDL-C.by.NMR | rs12225230 | 1.456 | 1.31E-11 | 0.38 | 3225 | 0.039 | 2.41E-01 | 0.94 | 1839 | 0.714 | 1.59E-01 | 0.75 | 1.64E-01 |
| 11q23.3 | TG.by.NMR | rs3135506 | 0.115 | 9.25E-29 | 1.01 | - | - | - | - | - | - | - | - | - |
| 11q23.3 | TG.assay | rs662799 | 0.138 | 3.53E-27 | 0.96 | 7423 | 0.313 | 1.57E-15 | 1.00 | - | - | - | - | - |
| 11q23.3 | VLDL.large | rs3135506 | 0.528 | 1.11E-12 | 0.42 | - | - | - | - | - | - | - | - | - |
| 11q23.3 | VLDL.total | rs3135506 | 9.711 | 2.86E-31 | 1.11 | - | - | - | - | - | - | - | - | - |
| 11q23.3 | VLDL.medium | rs3135506 | 5.040 | 1.21E-33 | 1.19 | - | - | - | - | - | - | - | - | - |
| 11q23.3 | VLDL.small | rs3135506 | 4.144 | 5.65E-16 | 0.54 | - | - | - | - | - | - | - | - | - |
| 12q24.31 | HDL.large | rs7307277 | 0.254 | 2.86E-08 | 0.25 | 2742 | -0.018 | 5.28E-01 | 0.75 | - | - | - | - | - |
| 15q22.1 | APOA1.assay | rs1800588 | 3.574 | 2.05E-24 | 0.86 | 2885 | 0.133 | 4.71E-05 | 1.00 | - | - | - | - | - |
| 15q22.1 | HDL-C.assay | rs1532085 | 1.421 | 8.92E-15 | 0.49 | 7423 | 0.086 | 3.49E-06 | 1.00 | 1854 | 0.034 | 7.37E-04 | 0.86 | 5.35E-08 |
| 15q22.1 | HDL.large | rs1800588 | 0.804 | 4.01E-54 | 1.95 | 2742 | 0.124 | 1.92E-04 | 1.00 | - | - | - | - | - |
| 15q22.1 | HDL.small | rs1800588 | -0.605 | 3.86E-13 | 0.43 | 2742 | -0.077 | 2.05E-02 | 0.93 | - | - | - | - | - |
| 15q22.1 | HDL.mean.size | rs1800588 | 0.093 | 1.54E-46 | 1.67 | 2742 | 0.135 | 4.84E-05 | 1.00 | - | - | - | - | - |
| 15q22.1 | IDL.total | rs1532085 | 0.112 | 3.71E-16 | 0.63 | 2742 | 0.102 | 3.84E-04 | 0.99 | 1604 | 0.136 | 1.32E-04 | 0.89 | 8.99E-07 |
| 15q22.1 | LDL.large | rs1800588 | 42.880 | 7.43E-39 | 1.39 | 2742 | 0.086 | 1.01E-02 | 1.00 | - | - | - | - | - |
| 15q22.1 | LDL.small | rs1800588 | -43.460 | 1.52E-09 | 0.30 | 2742 | -0.115 | 5.74E-04 | 0.82 | - | - | - | - | - |
| 15q22.1 | LDL.mean.size | rs1800588 | 0.114 | 3.51E-25 | 0.88 | 2742 | 0.077 | 2.08E-02 | 1.00 | - | - | - | - | - |
| 15q22.1 | HDL-C.by.NMR | rs1800588 | 2.088 | 3.31E-26 | 0.92 | 3225 | 0.109 | 4.36E-04 | 1.00 | - | - | - | - | - |
| 16q13 | APOA1.assay | rs1532624 | 3.912 | 1.10E-39 | 1.47 | 2885 | 0.164 | 2.70E-07 | 1.00 | 1824 | 2.572 | 6.79E-04 | 1.00 | 4.30E-09 |
| 16q13 | HDL-C.assay | rs1532624 | 3.094 | 1.32E-66 | 2.49 | 7423 | 0.256 | 1.17E-35 | 1.00 | 1854 | 0.053 | 1.06E-07 | 1.00 | 0.00E+00 |
| 16q13 | HDL.total | rs7499892 | -0.742 | 1.66E-14 | 0.48 | 2742 | -0.299 | 1.84E-08 | 0.95 | 1831 | -0.756 | 2.52E-04 | 0.85 | 1.25E-10 |
| 16q13 | HDL.large | rs1800775 | 0.703 | 1.86E-59 | 2.15 | 2742 | -0.209 | 1.74E-10 | 1.00 | 1836 | 0.553 | 7.36E-09 | 1.00 | - |
| 16q13 | HDL.mean.size | rs1800775 | 0.084 | 1.13E-53 | 1.94 | 2742 | -0.157 | 1.79E-06 | 1.00 | 1833 | 0.062 | 8.12E-07 | 1.00 | - |
| 16q13 | IDL.total | rs1800775 | -0.108 | 4.51E-16 | 0.63 | 2742 | 0.166 | 4.05E-07 | 0.99 | 1604 | -0.095 | 6.32E-03 | 0.89 | - |
| 16q13 | LDL.total | rs1800775 | -0.034 | 1.22E-18 | 0.64 | 2742 | 0.001 | 9.74E-01 | 0.99 | 1791 | -0.018 | 4.61E-02 | 0.92 | - |
| 16q13 | LDL.large | rs1864163 | -30.380 | 7.40E-22 | 0.76 | 2742 | -0.159 | 8.17E-05 | 1.00 | 1744 | -24.480 | 9.65E-04 | 0.95 | 1.37E-06 |
| 16q13 | LDL.small | rs1800775 | -68.900 | 9.36E-31 | 1.09 | 2742 | 0.039 | 2.31E-01 | 1.00 | 1821 | -35.040 | 4.46E-03 | 0.99 | - |
| 16q13 | LDL.mean.size | rs1800775 | 0.119 | 1.03E-38 | 1.38 | 2742 | -0.129 | 9.29E-05 | 1.00 | - | - | - | - | - |
| 16q13 | HDL-C.by.NMR | rs1532624 | 2.608 | 2.98E-55 | 2.05 | 3225 | 0.230 | 1.73E-14 | 1.00 | 1839 | 1.878 | 6.01E-07 | 1.00 | 0.00E+00 |
| 16q13 | VLDL.total | rs1800775 | -2.350 | 2.93E-09 | 0.29 | 2742 | 0.014 | 6.74E-01 | 0.81 | 1779 | -2.366 | 2.65E-02 | 0.62 | - |
| 16q13 | VLDL.small | rs1800775 | -1.878 | 8.84E-15 | 0.49 | 2742 | 0.064 | 5.17E-02 | 0.96 | 1755 | -1.817 | 2.21E-03 | 0.84 | - |
| 18q21.1 | APOA1.assay | rs4939883 | -2.616 | 2.45E-11 | 0.37 | 2885 | -0.190 | 1.98E-07 | 0.91 | 1824 | -2.699 | 4.77E-03 | 0.74 | 2.05E-08 |
| 18q21.1 | HDL-C.assay | rs4939883 | -1.330 | 1.94E-08 | 0.26 | 7423 | -0.147 | 7.08E-10 | 0.99 | 1854 | -0.034 | 6.69E-03 | 0.60 | 1.28E-10 |
| 18q21.1 | HDL.large | rs4939883 | -0.353 | 1.27E-09 | 0.31 | 2742 | -0.151 | 5.51E-05 | 0.83 | 1836 | -0.361 | 3.36E-03 | 0.66 | 3.05E-06 |
| 18q21.1 | HDL.mean.size | rs4939883 | -0.044 | 1.85E-09 | 0.30 | 2742 | -0.124 | 9.21E-04 | 0.82 | 1833 | -0.040 | 1.47E-02 | 0.65 | 1.65E-04 |
| 18q21.1 | LDL.large | rs8090363 | -15.870 | 1.96E-08 | 0.26 | 2742 | 0.030 | 2.87E-01 | 0.76 | 1744 | -3.000 | 6.52E-01 | 0.57 | - |
| 18q21.1 | LDL.mean.size | rs4939883 | -0.071 | 6.14E-09 | 0.28 | 2742 | -0.058 | 1.20E-01 | 0.79 | 1820 | -0.040 | 1.53E-01 | 0.62 | 9.19E-02 |
| 18q21.1 | HDL-C.by.NMR | rs4939883 | -1.591 | 5.28E-13 | 0.43 | 3225 | -0.151 | 1.25E-05 | 0.96 | 1839 | -1.587 | 8.21E-04 | 0.81 | 1.99E-07 |
| 19p13.2 | APOB.assay | rs6511720 | -4.492 | 2.77E-18 | 0.63 | 2821 | -0.205 | 3.00E-03 | 0.99 | 1817 | -4.265 | 1.38E-04 | 0.92 | 6.49E-06 |
| 19p13.2 | LDL-C.assay | rs6511720 | -6.892 | 1.49E-25 | 0.89 | 7344 | -0.262 | 4.39E-09 | 1.00 | 1805 | -0.150 | 4.00E-04 | 0.98 | 4.93E-11 |
| 19p13.2 | LDL.total | rs6511720 | -0.035 | 2.09E-09 | 0.29 | 2742 | -0.178 | 1.17E-02 | 0.81 | 1791 | -0.046 | 1.95E-03 | 0.63 | 2.66E-04 |
| 19p13.2 | LDL.large | rs6511720 | -29.440 | 2.96E-12 | 0.40 | 2742 | -0.080 | 2.57E-01 | 0.91 | 1744 | -26.280 | 1.56E-02 | 0.75 | 2.61E-02 |
| 19p13.2 | VLDL.small | rs6511720 | -2.092 | 1.93E-08 | 0.26 | 2742 | 0.007 | 9.21E-01 | 0.76 | 1755 | -2.420 | 1.48E-02 | 0.57 | - |
| 19q13.32 | APOA1.assay | rs769449 | -3.089 | 1.12E-11 | 0.38 | - | - | - | - | - | - | - | - | - |
| 19q13.32 | APOB.assay | rs769449 | 6.597 | 1.10E-36 | 1.32 | - | - | - | - | - | - | - | - | - |
| 19q13.32 | HDL-C.assay | rs769449 | -1.748 | 2.31E-10 | 0.33 | - | - | - | - | - | - | - | - | - |
| 19q13.32 | HDL.medium | rs405509 | -0.097 | 2.47E-10 | 0.38 | 2742 | -0.052 | 3.21E-01 | 0.90 | 1591 | -0.163 | 2.18E-04 | 0.69 | 7.40E-04 |
| 19q13.32 | LDL-C.assay | rs4803750 | -9.276 | 1.30E-27 | 0.98 | 7344 | -0.285 | 1.41E-08 | 1.00 | 1805 | -0.222 | 1.44E-05 | 0.99 | 6.13E-12 |
| 19q13.32 | LDL.total | rs769449 | 0.072 | 1.73E-33 | 1.19 | - | - | - | - | - | - | - | - | - |
| 19q13.32 | LDL.large | rs4803750 | -49.130 | 2.42E-19 | 0.67 | 2742 | -0.184 | 2.38E-02 | 0.99 | 1744 | -37.280 | 4.20E-03 | 0.93 | 1.02E-03 |
| 19q13.32 | LDL.small | rs769449 | 88.990 | 1.37E-21 | 0.75 | - | - | - | - | - | - | - | - | - |
| 19q13.32 | HDL-C.by.NMR | rs769449 | -1.713 | 2.36E-11 | 0.37 | - | - | - | - | - | - | - | - | - |
| 19q13.32 | TG.by.NMR | rs439401 | -0.035 | 3.58E-12 | 0.40 | 3229 | -0.103 | 7.11E-02 | 0.95 | 1791 | -0.037 | 1.74E-02 | 0.77 | 9.51E-03 |
| 19q13.32 | TG.assay | rs439401 | -0.043 | 1.77E-11 | 0.38 | 7423 | -0.059 | 1.35E-01 | 1.00 | 1809 | -0.038 | 1.73E-02 | 0.74 | 1.65E-02 |
| 19q13.32 | VLDL.large | rs439401 | -0.198 | 4.74E-08 | 0.25 | 2742 | -0.141 | 2.06E-02 | 0.74 | 1799 | -0.321 | 5.73E-02 | 0.56 | 9.13E-03 |
| 19q13.32 | VLDL.total | rs439401 | -2.532 | 6.18E-10 | 0.32 | 2742 | -0.087 | 1.51E-01 | 0.84 | 1779 | -1.864 | 9.32E-02 | 0.66 | 7.43E-02 |
| 19q13.32 | VLDL.small | rs439401 | -1.421 | 1.38E-08 | 0.27 | 2742 | -0.015 | 8.02E-01 | 0.77 | 1755 | -0.546 | 3.79E-01 | 0.58 | 6.66E-01 |
| 20q13.12 | HDL-C.assay | rs6065906 | -1.504 | 2.03E-11 | 0.37 | 7423 | -0.073 | 1.99E-03 | 1.00 | 1854 | -0.017 | 1.64E-01 | 0.75 | 2.94E-03 |
| 20q13.12 | HDL.total | rs6065906 | 1.029 | 3.23E-28 | 0.99 | 2742 | -0.054 | 1.46E-01 | 1.00 | 1831 | 0.974 | 2.28E-06 | 0.99 | - |
| 20q13.12 | HDL.large | rs6065904 | -0.689 | 3.58E-40 | 1.44 | 2742 | -0.177 | 1.99E-06 | 1.00 | 1836 | -0.544 | 1.32E-06 | 1.00 | 7.28E-11 |
| 20q13.12 | HDL.small | rs4810479 | 1.246 | 1.04E-55 | 2.03 | 2742 | -0.087 | 9.05E-03 | 1.00 | 1856 | 1.137 | 6.37E-11 | 1.00 | - |
| 20q13.12 | HDL.mean.size | rs6065906 | -0.087 | 8.33E-37 | 1.31 | 2742 | -0.154 | 2.89E-05 | 1.00 | 1833 | -0.063 | 8.79E-05 | 1.00 | 5.28E-08 |
| 20q13.12 | LDL.large | rs4810479 | -17.990 | 1.18E-08 | 0.27 | 2742 | -0.057 | 8.90E-02 | 0.78 | 1744 | -11.270 | 1.28E-01 | 0.58 | 6.22E-02 |
| 20q13.12 | LDL.small | rs6065906 | 53.010 | 3.10E-12 | 0.40 | 2742 | 0.035 | 3.45E-01 | 0.91 | 1821 | 20.720 | 1.85E-01 | 0.77 | 2.40E-01 |
| 20q13.12 | LDL.mean.size | rs6065906 | -0.090 | 1.05E-14 | 0.49 | 2742 | -0.062 | 9.29E-02 | 0.96 | 1820 | -0.051 | 6.20E-02 | 0.85 | 3.55E-02 |
| 20q13.12 | TG.assay | rs6065906 | 0.045 | 1.39E-08 | 0.26 | 7423 | 0.066 | 5.29E-03 | 0.99 | 1809 | 0.031 | 1.07E-01 | 0.59 | 4.78E-03 |

^a^Beta coefficient, significance, proportion variance explained in regression model testing association of the does of the minor allele of SNPs with indicated lipoprotein fractions after adjustment for clinical covariates (see Methods).

^b^Available power given the effect estimate in the WGHS sample and the sample size in either the FHS or PROCARDIS cohorts

^c^P-value estimated by Fisher’s method for combined FHS and PROCARDIS cohorts when beta coefficients had same sign
