# Supplementary material for: Forty-Three Loci Associated with Plasma Lipoprotein Size, Concentration, and Cholesterol Content in Genome-Wide Analysis
Source: PLoS Genet. 2009 Nov 20;5(11):e1000730. doi: 10.1371/journal.pgen.1000730 (PMC2777390; doi:10.1371/journal.pgen.1000730)
Supplement: Table S5 — Proportion of variance in fully adjusted lipoprotein fractions explained in the whole sample by genetic variation at the candidate loci. (0.15 MB DOC) [file pgen.1000730.s009.doc]

Table S5. Proportion (%) of variance explained at genomewide loci in whole sample with fully adjusted lipoprotein fractions

|  | Lipoprotein fraction | | | | | | | | | | | | | | | | | | | | | |
| --- | --- | --- | --- | --- | --- | --- | --- | --- | --- | --- | --- | --- | --- | --- | --- | --- | --- | --- | --- | --- | --- | --- |
| Locus | LDL:L | LDL:S | LDL:Z | IDL | LDL:T | LDL-C | ApoB | HDL:T | HDL:L | HDL:M | HDL:S | HDL:Z | HDL-C:NMR | HDL-C | ApoA1 | VLDL:T | VLDL:S | VLDL:M | VLDL:S | VLDL:Z | TG:NMR | TG |
| 1p32.3 | 0.32 | 0.12 | - | 0.14 | 0.55 | 0.93 | 0.86 | - | - | 0.06 | - | - | - | - | - | 0.24 | - | - | 0.33 | 0.10 | 0.20 | - |
| 1p31.3 | 0.06 | - | 0.08 | 0.18 | - | 0.14 | 0.13 | 0.11 | - | 0.23 | - | - | 0.21 | - | 0.21 | 0.35 | 0.12 | 0.28 | 0.24 | - | 0.32 | 0.15 |
| 1p13.3 | 0.22 | 0.26 | - | - | 0.75 | 1.19 | 1.49 | 0.06 | - | 0.14 | - | - | - | - | - | 0.24 | - | - | 0.45 | 0.14 | - | - |
| 1q23.3 | - | - | - | - | - | - | - | 0.13 | - | 0.28 | - | 0.12 | - | - | - | - | - | - | - | - | - | - |
| 2p24.1 | 0.52 | 0.32 | 0.17 | 0.29 | 0.86 | 1.06 | 1.54 | - | 0.10 | - | - | 0.16 | 0.11 | 0.22 | 0.10 | 1.76 | 0.12 | 0.55 | 2.16 | 0.26 | 0.59 | 0.27 |
| 2p23.3 | 0.04 | 0.50 | 0.30 | 0.25 | 0.49 | 0.10 | 0.47 | 0.88 | - | 0.17 | 0.49 | 0.35 | 0.13 | - | 0.26 | 0.44 | 0.70 | 0.45 | 0.21 | 0.40 | 0.93 | 1.00 |
| 2p21 | 0.24 | - | - | - | 0.20 | 0.42 | 0.38 | - | - | - | - | - | - | - | - | 0.08 | - | - | 0.09 | - | 0.08 | - |
| 2q24.3 | - | - | 0.07 | - | 0.10 | - | - | - | 0.16 | - | - | 0.17 | 0.13 | 0.19 | 0.10 | - | 0.05 | - | - | - | 0.08 | 0.05 |
| 3q22.3 | - | - | - | - | - | - | - | 0.14 | - | - | 0.22 | - | - | 0.06 | 0.08 | - | - | - | - | - | 0.04 | 0.06 |
| 5q13.3 | 0.20 | - | 0.08 | - | - | 0.31 | 0.16 | - | - | - | - | - | - | - | - | 0.09 | - | - | 0.13 | 0.09 | - | - |
| 6p21.32 | - | 0.12 | 0.11 | - | 0.07 | 0.05 | - | 0.09 | 0.09 | - | - | 0.13 | - | 0.07 | 0.06 | 0.10 | 0.20 | 0.12 | - | 0.11 | 0.28 | 0.17 |
| 7q11.23 | 0.19 | 0.24 | 0.25 | - | 0.11 | - | 0.12 | 0.22 | 0.06 | - | 0.27 | 0.12 | - | - | - | 0.26 | 0.20 | 0.28 | 0.12 | - | 0.39 | 0.48 |
| 7q32.2 | - | 0.24 | 0.20 | - | 0.23 | - | 0.14 | - | 0.18 | - | - | 0.23 | 0.15 | 0.18 | 0.08 | 0.12 | 0.09 | - | 0.12 | - | 0.15 | 0.21 |
| 8p23.1 | - | - | - | - | - | 0.09 | - | 0.14 | - | 0.12 | - | - | - | 0.07 | - | - | - | - | - | 0.12 | - | - |
| 8p21.3 | 0.28 | 0.62 | 0.58 | - | 0.39 | - | 0.30 | 0.08 | 0.70 | 0.07 | 0.11 | 0.59 | 0.51 | 0.76 | 0.37 | 1.13 | 0.52 | 1.19 | 0.45 | 0.07 | 1.11 | 1.25 |
| 8q24.13 | 0.08 | 0.28 | 0.27 | 0.14 | 0.28 | 0.12 | 0.27 | - | 0.17 | - | 0.06 | 0.09 | 0.07 | 0.09 | - | 0.15 | 0.12 | 0.10 | 0.12 | - | 0.22 | 0.28 |
| 9q31.1 | - | - | - | - | - | - | - | 0.11 | 0.23 | 0.62 | 0.39 | 0.17 | 0.12 | 0.35 | 0.49 | 0.10 | - | 0.13 | - | 0.05 | 0.08 | 0.05 |
| 9q34.2 | 0.18 | - | 0.11 | - | - | 0.34 | - | - | - | - | 0.06 | - | - | 0.09 | - | 0.16 | - | - | 0.32 | 0.18 | - | - |
| 11q12.2 | 0.24 | - | - | - | 0.11 | 0.11 | 0.08 | - | 0.37 | 0.40 | - | 0.31 | 0.14 | 0.14 | - | 0.09 | 0.19 | - | 0.10 | 0.13 | 0.09 | 0.17 |
| 11q23.3 | 0.24 | 0.88 | 0.73 | 0.27 | 0.72 | 0.23 | 0.89 | 0.79 | 0.44 | 0.30 | 1.06 | 0.26 | 0.58 | 0.85 | 0.84 | 2.48 | 0.95 | 2.48 | 1.22 | - | 2.35 | 2.10 |
| 12q23.2 | - | - | - | - | - | - | - | 0.21 | 0.09 | 0.12 | - | - | 0.19 | 0.06 | 0.16 | - | 0.08 | - | - | 0.13 | - | - |
| 12q24.31.A | 0.12 | - | - | 0.08 | 0.06 | 0.21 | 0.16 | 0.06 | - | 0.05 | - | - | 0.07 | - | 0.11 | - | - | - | 0.06 | - | - | - |
| 12q24.31.B | 0.08 | 0.35 | 0.35 | - | 0.28 | - | 0.16 | - | 0.38 | - | - | 0.36 | 0.26 | 0.27 | 0.13 | 0.12 | 0.15 | 0.09 | 0.07 | 0.11 | 0.20 | 0.27 |
| 15q22.1 | 2.67 | 0.71 | 2.04 | 1.18 | - | 0.05 | 0.08 | - | 4.52 | 0.27 | 0.89 | 3.93 | 2.32 | 1.36 | 1.94 | 0.04 | - | 0.06 | 0.15 | - | - | 0.20 |
| 16q13 | 1.01 | 1.67 | 2.12 | 0.67 | 1.04 | 0.10 | 0.12 | 0.69 | 3.47 | - | - | 3.17 | 3.43 | 3.84 | 2.08 | 0.35 | - | - | 0.52 | 0.08 | 0.12 | 0.18 |
| 17q24.2 | - | - | - | - | - | - | - | - | - | 0.20 | - | 0.07 | - | - | - | 0.06 | - | - | 0.04 | - | 0.05 | 0.06 |
| 18q21.1 | 0.22 | 0.11 | 0.21 | - | - | - | - | 0.14 | 0.24 | 0.15 | - | 0.21 | 0.47 | 0.22 | 0.43 | 0.10 | - | 0.08 | 0.08 | - | 0.07 | - |
| 19p13.2 | 0.80 | - | - | 0.22 | 0.37 | 1.15 | 0.80 | 0.06 | 0.05 | - | - | - | 0.08 | - | 0.05 | 0.07 | - | - | 0.23 | - | - | - |
| 19q13.32 | 3.98 | 2.12 | 0.43 | 0.14 | 8.39 | 7.08 | 8.87 | 0.51 | 0.39 | 1.37 | 0.30 | 0.49 | 1.11 | 0.61 | 0.70 | 0.30 | 0.30 | 0.18 | 0.25 | 0.14 | 0.41 | 0.51 |
| 20q13.12.A | - | - | - | - | - | - | - | 0.11 | - | - | 0.08 | - | 0.13 | 0.13 | 0.16 | - | - | - | - | - | - | - |
| 20q13.12.B | 0.28 | 0.34 | 0.41 | - | 0.21 | - | 0.18 | 1.05 | 1.44 | 0.07 | 2.48 | 1.32 | 0.12 | 0.38 | - | 0.12 | - | - | 0.13 | - | 0.11 | 0.23 |
