# Supplementary material for: Forty-Three Loci Associated with Plasma Lipoprotein Size, Concentration, and Cholesterol Content in Genome-Wide Analysis
Source: PLoS Genet. 2009 Nov 20;5(11):e1000730. doi: 10.1371/journal.pgen.1000730 (PMC2777390; doi:10.1371/journal.pgen.1000730)
Supplement: Table S6 — Proportion of variance in fully adjusted lipoprotein fractions explained in the fasting sub-sample by genetic variation at the candidate loci. (0.15 MB DOC) [file pgen.1000730.s010.doc]

Table S6. Proportion (%) of variance explained at genomewide loci in fasting subsample with fully adjusted lipoprotein fractions

|  | Lipoprotein fraction | | | | | | | | | | | | | | | | | | | | | |
| --- | --- | --- | --- | --- | --- | --- | --- | --- | --- | --- | --- | --- | --- | --- | --- | --- | --- | --- | --- | --- | --- | --- |
| locus | LDL:L | LDL:S | LDL:Z | IDL | LDL:T | LDL-C | ApoB | HDL:T | HDL:L | HDL:M | HDL:S | HDL:Z | HDL-C:NMR | HDL-C | ApoA1 | VLDL:T | VLDL:S | VLDL:M | VLDL:S | VLDL:Z | TG:NMR | TG |
| 1p32.3 | 0.34 | 0.12 | - | 0.17 | 0.70 | 1.06 | 0.93 | - | - | 0.10 | - | - | - | - | - | 0.27 | - | - | 0.40 | 0.09 | 0.24 | - |
| 1p31.3 | 0.10 | - | - | 0.07 | - | 0.13 | 0.13 | 0.17 | - | 0.30 | - | - | 0.23 | - | - | 0.42 | 0.17 | 0.32 | 0.30 | - | 0.36 | 0.19 |
| 1p13.3 | 0.21 | 0.25 | - | - | 0.75 | 1.17 | 1.44 | 0.08 | - | 0.11 | - | - | - | - | - | 0.31 | - | - | 0.56 | 0.11 | - | - |
| 1q23.3 | 0.08 | - | - | - | - | - | - | 0.18 | - | 0.32 | - | 0.13 | - | - | 0.11 | - | - | - | - | - | - | - |
| 2p24.1 | 0.41 | 0.35 | 0.28 | 0.21 | 0.98 | 0.87 | 1.47 | - | - | - | - | 0.07 | - | 0.14 | 0.11 | 1.75 | 0.11 | 0.61 | 2.12 | 0.27 | 0.68 | 0.40 |
| 2p23.3 | 0.08 | 0.60 | 0.42 | 0.25 | 0.55 | 0.14 | 0.46 | 0.96 | - | 0.21 | 0.54 | 0.28 | 0.15 | - | 0.29 | 0.46 | 0.85 | 0.51 | 0.11 | 0.47 | 1.02 | 1.12 |
| 2p21 | 0.20 | - | - | - | 0.13 | 0.43 | 0.38 | - | - | - | - | - | - | - | - | - | - | - | 0.11 | - | - | - |
| 2q24.3 | - | - | 0.08 | - | 0.09 | - | - | - | 0.12 | - | - | - | - | 0.13 | - | - | 0.07 | - | - | - | - | - |
| 3q22.3 | - | - | - | - | - | - | - | 0.14 | - | - | 0.14 | - | - | 0.09 | 0.09 | 0.06 | - | 0.06 | - | - | 0.06 | 0.07 |
| 5q13.3 | 0.28 | - | - | - | - | 0.33 | 0.19 | - | - | - | - | - | - | - | - | 0.09 | - | - | 0.15 | 0.14 | - | - |
| 6p21.32 | - | 0.13 | 0.15 | - | 0.12 | 0.09 | 0.12 | 0.09 | 0.08 | - | - | 0.13 | 0.07 | 0.07 | 0.07 | 0.12 | 0.29 | 0.16 | - | 0.17 | 0.26 | 0.20 |
| 7q11.23 | 0.27 | 0.18 | 0.25 | - | - | - | - | 0.24 | 0.09 | - | 0.24 | 0.12 | - | - | - | 0.22 | 0.22 | 0.25 | - | - | 0.36 | 0.47 |
| 7q32.2 | - | 0.22 | 0.17 | 0.12 | 0.23 | 0.06 | 0.18 | - | 0.17 | - | - | 0.20 | 0.10 | 0.13 | - | 0.13 | 0.11 | - | 0.14 | - | 0.17 | 0.24 |
| 8p23.1 | - | - | - | - | - | 0.13 | - | 0.15 | - | 0.13 | 0.08 | - | - | - | - | - | 0.07 | - | - | 0.23 | - | - |
| 8p21.3 | 0.30 | 0.62 | 0.49 | - | 0.27 | - | 0.25 | - | 0.62 | 0.12 | 0.10 | 0.50 | 0.54 | 0.79 | 0.41 | 0.99 | 0.50 | 1.09 | 0.44 | - | 0.94 | 1.12 |
| 8q24.13 | - | 0.37 | 0.27 | 0.15 | 0.38 | 0.16 | 0.32 | - | 0.08 | - | - | 0.12 | - | - | - | 0.15 | 0.11 | 0.11 | 0.21 | - | 0.22 | 0.29 |
| 9q31.1 | - | - | - | - | - | - | - | 0.18 | 0.18 | 0.57 | 0.24 | 0.26 | 0.34 | 0.32 | 0.46 | 0.08 | - | 0.14 | - | - | 0.09 | 0.06 |
| 9q34.2 | 0.23 | - | 0.10 | - | - | 0.36 | - | - | - | - | 0.12 | - | - | 0.13 | 0.12 | 0.17 | - | - | 0.31 | 0.21 | - | - |
| 11q12.2 | 0.25 | - | - | - | 0.13 | 0.12 | - | - | 0.36 | 0.34 | - | 0.28 | 0.16 | 0.15 | - | 0.12 | 0.14 | - | 0.16 | 0.17 | 0.10 | 0.20 |
| 11q23.3 | - | 0.98 | 0.77 | 0.16 | 0.74 | 0.31 | 1.02 | 0.65 | 0.50 | 0.34 | 0.98 | 0.48 | 0.72 | 0.82 | 1.00 | 2.50 | 0.90 | 2.53 | 1.29 | - | 2.43 | 2.17 |
| 12q23.2 | - | - | - | - | - | - | - | 0.24 | 0.06 | - | - | - | 0.17 | - | 0.14 | - | 0.12 | - | 0.06 | 0.19 | - | - |
| 12q24.31.A | 0.09 | - | - | 0.12 | - | 0.16 | 0.16 | 0.08 | - | - | - | - | 0.07 | - | 0.11 | - | - | - | - | - | - | - |
| 12q24.31.B | 0.11 | 0.37 | 0.39 | - | 0.28 | - | 0.11 | - | 0.41 | - | - | 0.36 | 0.30 | 0.15 | 0.13 | - | 0.14 | - | - | 0.12 | 0.14 | 0.23 |
| 15q22.1 | 2.40 | 0.71 | 1.82 | 1.34 | - | 0.09 | 0.17 | - | 4.22 | 0.24 | 0.79 | 3.47 | 2.05 | 1.13 | 1.76 | - | - | 0.07 | 0.08 | - | - | 0.23 |
| 16q13 | 1.15 | 1.67 | 2.10 | 0.77 | 0.79 | 0.08 | 0.09 | 0.63 | 3.41 | - | - | 2.96 | 3.09 | 3.84 | 1.91 | 0.31 | - | - | 0.52 | 0.07 | - | 0.13 |
| 17q24.2 | - | - | - | - | - | - | - | - | - | 0.22 | - | 0.07 | - | - | - | - | - | - | - | - | - | - |
| 18q21.1 | 0.36 | 0.19 | 0.32 | - | 0.12 | - | - | 0.11 | 0.31 | 0.10 | - | 0.30 | 0.44 | 0.26 | 0.45 | - | - | - | - | - | - | - |
| 19p13.2 | 0.53 | - | - | 0.14 | 0.29 | 1.26 | 0.80 | - | - | - | - | - | 0.07 | - | - | 0.07 | - | - | 0.20 | - | - | - |
| 19q13.32 | 3.64 | 2.23 | 0.57 | - | 8.19 | 6.80 | 8.62 | 0.55 | 0.36 | 1.19 | 0.14 | 0.57 | 1.10 | 0.59 | 0.55 | 0.32 | 0.26 | 0.16 | 0.27 | 0.15 | 0.41 | 0.67 |
| 20q13.12.A | - | - | - | - | - | - | - | 0.10 | - | - | - | - | 0.11 | - | 0.11 | - | - | - | - | - | - | - |
| 20q13.12.B | 0.39 | 0.41 | 0.51 | - | 0.23 | 0.07 | - | 1.04 | 1.57 | 0.12 | 2.35 | 1.44 | 0.14 | 0.38 | - | 0.10 | 0.08 | - | - | 0.07 | 0.11 | 0.25 |
