# Supplementary material for: Forty-Three Loci Associated with Plasma Lipoprotein Size, Concentration, and Cholesterol Content in Genome-Wide Analysis
Source: PLoS Genet. 2009 Nov 20;5(11):e1000730. doi: 10.1371/journal.pgen.1000730 (PMC2777390; doi:10.1371/journal.pgen.1000730)
Supplement: Table S7 — Total proportion of variance explained by candidate loci for each of the unadjusted lipoprotein fractions. (0.04 MB DOC) [file pgen.1000730.s011.doc]

Table S7. Proportion variance (%) explained of unadjusted lipoprotein fractions at candidate loci

|  | whole sample | fasting subsample |
| --- | --- | --- |
| LDL large | 11.6 | 11.2 |
| LDL small | 7.7 | 7.7 |
| LDL mean size | 7.6 | 7.4 |
| IDL total | 3.0 | 3.1 |
| LDL total | 12.4 | 11.8 |
| LDL-C assay | 12.8 | 12.9 |
| APOB assay | 14.7 | 14.6 |
| HDL total | 4.5 | 4.5 |
| HDL large | 10.8 | 10.2 |
| HDL medium | 4.1 | 3.9 |
| HDL small | 5.9 | 5.5 |
| HDL mean size | 9.8 | 9.5 |
| HDL-C by NMR | 7.8 | 7.3 |
| HDL-C assay | 8.1 | 8.2 |
| APOA1 assay | 6.2 | 6.0 |
| VLDL total | 8.2 | 7.8 |
| VLDL large | 3.2 | 3.3 |
| VLDL medium | 5.7 | 5.6 |
| VLDL small | 6.6 | 6.9 |
| VLDL mean size | 1.9 | 2.0 |
| TG by NMR | 6.7 | 6.5 |
| TG assay | 6.1 | 6.4 |
