# Supplementary material for: Forty-Three Loci Associated with Plasma Lipoprotein Size, Concentration, and Cholesterol Content in Genome-Wide Analysis
Source: PLoS Genet. 2009 Nov 20;5(11):e1000730. doi: 10.1371/journal.pgen.1000730 (PMC2777390; doi:10.1371/journal.pgen.1000730)
Supplement: Table S8 — Sensitivity analysis for locus discovery procedure. (0.10 MB DOC) [file pgen.1000730.s012.doc]

Table S8. Sensitivity analysis of genomewide associations: Loci with genomewide associations in either the whole sample or the fasting subsample for a series of adjustment procedures

|  | Lipoprotein adjustment procedure* | | | | | | | |  |
| --- | --- | --- | --- | --- | --- | --- | --- | --- | --- |
| locus | full | full  ev.qnorm | min | min  ev.qnorm | age | age  ev.qnorm | trig | trig  ev.qnorm | candidate gene(s) |
| 1p32.3 | x | x | x | x | x | x | x | x | *PCSK9* |
| 1p31.3 | x | x | x | x | x | x | x | x | *ANGPTL3* |
| 1p13.3 | x | x | x | x | x | x | x | x | *CELSR2, PSRC1, SARS, SORT1* |
| 1q23.3 | x | x | x | x | x | x | x | x | *APOA2, B4GALT3* |
| 2p24.1 | x | x | x | x | x | x | x | x | *APOB* |
| 2p23.3 | x | x | x | x | x | x | x | x | *GCKR* |
| 2p21 | x | x | x | x | x | x | x | x | *ABCG5, ABCG8* |
| 2q24.3 | x | x |  |  |  |  |  |  | *COBLL1, GRB14* |
| 3q22.3 | x | x |  |  |  |  | x | x | *PCCB, STAG1* |
| 5q13.3 | x | x | x | x | x | x | x | x | *HMGCR* |
| 6p22.3 |  |  |  | x | x | x |  | x | *GMPR, MYLIP* |
| 6p21.32 | x | x | x | x | x | x |  |  | *BTNL2, HLA-DRA, HLA-DRB5* |
| 6q25.3 |  | x |  | x |  |  | x | x | *LPA, LPAL2* |
| 7q11.23 | x | x | x | x | x | x | x | x | *MLXIPL* |
| 7q32.2 | x | x |  |  |  |  | x | x | *COPG2, KLF14, TSGA13* |
| 8p23.1 | x |  | x |  | x |  |  |  | *intergenic, PPP1R3B* |
| 8p21.3 | x | x | x | x | x | x | x | x | *LPL* |
| 8q24.13 | x | x | x | x | x | x | x | x | *TRIB1* |
| 9q31.1 | x | x | x | x | x | x | x | x | *ABCA1* |
| 9q34.2 | x | x | x | x | x | x | x | x | *ABO* |
| 10q21.3 |  |  | x | x | x | x |  |  | *JMJD1C, NRBF2, REEP3* |
| 11p15.4 |  |  |  |  |  |  | x |  | *SBF2* |
| 11q12.2 | x | x | x | x | x | x | x | x | *FADS1, FADS2, FADS3* |
| 11q23.3 | x | x | x | x | x | x | x | x | *APOA1, APOA4, APOA5, APOC3* |
| 12q23.2 | x | x | x | x | x | x | x | x | *intergenic, ASCL1, PAH* |
| 12q24.31.A | x | x | x | x | x | x | x | x | *HNF1A/TCF1* |
| 12q24.31.B | x | x | x | x | x | x | x | x | *CCDC92, DNAH10, ZNF664* |
| 15q22.1 | x | x | x | x | x | x | x | x | *LIPC* |
| 16q13 | x | x | x | x | x | x | x | x | *CETP* |
| 16q22.1 |  |  | x |  | x |  | x | x | *LCAT* |
| 17q24.2.A |  | x | x | x | x | x | x | x | *APOH* |
| 17q24.2.B | x |  |  |  |  |  |  |  | *ARSG, FAM20A, PRKAR1A, WIPI1* |
| 18q21.1 | x | x | x | x | x | x | x | x | *LIPG* |
| 19p13.2 | x | x | x | x | x | x | x | x | *LDLR* |
| 19q13.32 | x | x | x | x | x | x | x | x | *APOC1, APOC2, APOC4, APOE* |
| 20q13.12.A | x |  | x |  |  |  | x |  | *HNF4A* |
| 20q13.12.B | x | x | x | x | x | x | x | x | *PLTP* |

*Adjustment procedures

full=age, BMI, menopausal status, smoking status, use of hormone replacement therapy

min=as for full, but omitting BMI

age=age only

trig=as for full, but including log transformed triglyceride levels

ev.qnorm=adjustment also includes top 10 population substructure eigenvectors followed by quantile normalization of residuals.
