# Supplementary material for: Forty-Three Loci Associated with Plasma Lipoprotein Size, Concentration, and Cholesterol Content in Genome-Wide Analysis
Source: PLoS Genet. 2009 Nov 20;5(11):e1000730. doi: 10.1371/journal.pgen.1000730 (PMC2777390; doi:10.1371/journal.pgen.1000730)
Supplement: Table S9 — Lipoprotein associations in the whole sample at loci in previous lipid fraction GWAS. (0.34 MB DOC) [file pgen.1000730.s013.doc]

Table S9. Lipoprotein associations at loci in previous lipid fraction GWAS (whole WGHS sample)

| locus | candidate gene(s) | N snps | P<Pmax^+^ | lipoprotein | best SNP | beta (se) | p-value |
| --- | --- | --- | --- | --- | --- | --- | --- |
| 1p36.11 | *TMEM57* | 8 |  | LDL large | rs873308 | -5.8 (2.3) | 0.013 |
| 1p36.11 | *TMEM57* | 8 |  | LDL small | - | - | - |
| 1p36.11 | *TMEM57* | 8 |  | LDL mean size | - | - | - |
| 1p36.11 | *TMEM57* | 8 |  | IDL total | - | - | - |
| 1p36.11 | *TMEM57* | 8 |  | LDL total | rs10903129 | -0.0082 (0.0033) | 0.013 |
| 1p36.11 | *TMEM57* | 8 | * | LDL-C assay | rs10903129 | -1.3 (0.37) | 0.00023 |
| 1p36.11 | *TMEM57* | 8 |  | APOB assay | rs10903129 | -0.89 (0.29) | 0.0018 |
| 1p36.11 | *TMEM57* | 8 |  | HDL total | - | - | - |
| 1p36.11 | *TMEM57* | 8 |  | HDL large | - | - | - |
| 1p36.11 | *TMEM57* | 8 |  | HDL medium | - | - | - |
| 1p36.11 | *TMEM57* | 8 |  | HDL small | rs3924486 | -0.15 (0.059) | 0.0095 |
| 1p36.11 | *TMEM57* | 8 |  | HDL mean size | - | - | - |
| 1p36.11 | *TMEM57* | 8 |  | HDL-C by NMR | - | - | - |
| 1p36.11 | *TMEM57* | 8 |  | HDL-C assay | - | - | - |
| 1p36.11 | *TMEM57* | 8 |  | APOA1 assay | - | - | - |
| 1p36.11 | *TMEM57* | 8 |  | VLDL total | - | - | - |
| 1p36.11 | *TMEM57* | 8 |  | VLDL large | - | - | - |
| 1p36.11 | *TMEM57* | 8 |  | VLDL medium | - | - | - |
| 1p36.11 | *TMEM57* | 8 |  | VLDL small | rs10903129 | -0.53 (0.20) | 0.0097 |
| 1p36.11 | *TMEM57* | 8 |  | VLDL mean size | - | - | - |
| 1p36.11 | *TMEM57* | 8 |  | TG by NMR | - | - | - |
| 1p36.11 | *TMEM57* | 8 |  | TG assay | - | - | - |
| 1q42.13 | *GALNT2* | 57 | * | LDL large | rs4846904 | -14 (2.9) | 1.00E-06 |
| 1q42.13 | *GALNT2* | 57 |  | LDL small | rs4846904 | 22 (6.3) | 0.00039 |
| 1q42.13 | *GALNT2* | 57 | * | LDL mean size | rs4846904 | -0.044 (0.0096) | 5.50E-06 |
| 1q42.13 | *GALNT2* | 57 |  | IDL total | rs3811492 | -0.039 (0.017) | 0.022 |
| 1q42.13 | *GALNT2* | 57 |  | LDL total | rs10779835 | 0.0095 (0.0034) | 0.0047 |
| 1q42.13 | *GALNT2* | 57 |  | LDL-C assay | rs12133061 | -0.95 (0.41) | 0.021 |
| 1q42.13 | *GALNT2* | 57 |  | APOB assay | rs1998064 | -0.7 (0.33) | 0.032 |
| 1q42.13 | *GALNT2* | 57 |  | HDL total | rs10779835 | -0.22 (0.064) | 0.00053 |
| 1q42.13 | *GALNT2* | 57 |  | HDL large | rs4846904 | -0.19 (0.046) | 5.40E-05 |
| 1q42.13 | *GALNT2* | 57 | * | HDL medium | rs10779835 | -0.055 (0.013) | 3.50E-05 |
| 1q42.13 | *GALNT2* | 57 |  | HDL small | rs4847022 | 0.21 (0.074) | 0.0048 |
| 1q42.13 | *GALNT2* | 57 |  | HDL mean size | rs4846908 | 0.014 (0.0046) | 0.0017 |
| 1q42.13 | *GALNT2* | 57 | * | HDL-C by NMR | rs10779835 | -0.69 (0.14) | 1.60E-06 |
| 1q42.13 | *GALNT2* | 57 |  | HDL-C assay | rs4846908 | 0.51 (0.15) | 0.00092 |
| 1q42.13 | *GALNT2* | 57 |  | APOA1 assay | rs4846908 | 1.0 (0.25) | 4.80E-05 |
| 1q42.13 | *GALNT2* | 57 |  | VLDL total | rs11122490 | -0.94 (0.38) | 0.015 |
| 1q42.13 | *GALNT2* | 57 |  | VLDL large | rs3811485 | -0.10 (0.047) | 0.028 |
| 1q42.13 | *GALNT2* | 57 |  | VLDL medium | rs2273970d | 0.81 (0.4) | 0.04 |
| 1q42.13 | *GALNT2* | 57 |  | VLDL small | rs11122490 | -0.7 (0.24) | 0.0032 |
| 1q42.13 | *GALNT2* | 57 |  | VLDL mean size | rs2306813 | 0.32 (0.11) | 0.0029 |
| 1q42.13 | *GALNT2* | 57 |  | TG by NMR | - | - | - |
| 1q42.13 | *GALNT2* | 57 |  | TG assay | rs2296065 | 0.020 (0.0076) | 0.0095 |
| 5q33.3 | *TIMD4|HAVCR1* | 27 |  | LDL large | rs7706174 | -10 (2.9) | 6.00E-04 |
| 5q33.3 | *TIMD4|HAVCR1* | 27 |  | LDL small | - | - | - |
| 5q33.3 | *TIMD4|HAVCR1* | 27 |  | LDL mean size | rs7706174 | -0.024 (0.0097) | 0.015 |
| 5q33.3 | *TIMD4|HAVCR1* | 27 |  | IDL total | rs1363232 | -0.033 (0.012) | 0.0062 |
| 5q33.3 | *TIMD4|HAVCR1* | 27 |  | LDL total | rs1363232 | -0.013 (0.0034) | 0.00022 |
| 5q33.3 | *TIMD4|HAVCR1* | 27 | * | LDL-C assay | rs1363232 | -1.7 (0.38) | 5.50E-06 |
| 5q33.3 | *TIMD4|HAVCR1* | 27 | * | APOB assay | rs1363232 | -1.5 (0.3) | 4.60E-07 |
| 5q33.3 | *TIMD4|HAVCR1* | 27 |  | HDL total | - | - | - |
| 5q33.3 | *TIMD4|HAVCR1* | 27 |  | HDL large | rs6873053d | 0.16 (0.067) | 0.015 |
| 5q33.3 | *TIMD4|HAVCR1* | 27 |  | HDL medium | rs4704810 | 0.038 (0.013) | 0.005 |
| 5q33.3 | *TIMD4|HAVCR1* | 27 |  | HDL small | - | - | - |
| 5q33.3 | *TIMD4|HAVCR1* | 27 |  | HDL mean size | - | - | - |
| 5q33.3 | *TIMD4|HAVCR1* | 27 |  | HDL-C by NMR | rs7706174 | -0.38 (0.18) | 0.03 |
| 5q33.3 | *TIMD4|HAVCR1* | 27 |  | HDL-C assay | rs2279804 | -0.31 (0.15) | 0.039 |
| 5q33.3 | *TIMD4|HAVCR1* | 27 |  | APOA1 assay | - | - | - |
| 5q33.3 | *TIMD4|HAVCR1* | 27 | * | VLDL total | rs1363232 | -1.4 (0.35) | 5.60E-05 |
| 5q33.3 | *TIMD4|HAVCR1* | 27 |  | VLDL large | rs1363232 | -0.091 (0.032) | 0.0044 |
| 5q33.3 | *TIMD4|HAVCR1* | 27 |  | VLDL medium | rs1363232 | -0.54 (0.17) | 0.0018 |
| 5q33.3 | *TIMD4|HAVCR1* | 27 |  | VLDL small | rs1363232 | -0.76 (0.21) | 0.00035 |
| 5q33.3 | *TIMD4|HAVCR1* | 27 |  | VLDL mean size | rs2279804 | -0.19 (0.081) | 0.02 |
| 5q33.3 | *TIMD4|HAVCR1* | 27 | * | TG by NMR | rs1363232 | -0.020 (0.0044) | 3.30E-06 |
| 5q33.3 | *TIMD4|HAVCR1* | 27 |  | TG assay | rs1363232 | -0.018 (0.0056) | 0.0011 |
| 11p11.2-11p11.12 | *MADD|FOLH1|NR1H3* | 125 |  | LDL large | rs2957873 | 9.7 (3.0) | 0.00098 |
| 11p11.2-11p11.12 | *MADD|FOLH1|NR1H3* | 125 |  | LDL small | rs10769253 | -17 (6.7) | 0.0089 |
| 11p11.2-11p11.12 | *MADD|FOLH1|NR1H3* | 125 |  | LDL mean size | rs2957873 | 0.035 (0.0097) | 0.00029 |
| 11p11.2-11p11.12 | *MADD|FOLH1|NR1H3* | 125 |  | IDL total | rs7126210 | -0.028 (0.013) | 0.028 |
| 11p11.2-11p11.12 | *MADD|FOLH1|NR1H3* | 125 |  | LDL total | rs3740689 | -0.009 (0.0034) | 0.0078 |
| 11p11.2-11p11.12 | *MADD|FOLH1|NR1H3* | 125 |  | LDL-C assay | rs1055447 | -0.8 (0.37) | 0.028 |
| 11p11.2-11p11.12 | *MADD|FOLH1|NR1H3* | 125 |  | APOB assay | rs4647709 | -1.1 (0.48) | 0.024 |
| 11p11.2-11p11.12 | *MADD|FOLH1|NR1H3* | 125 |  | HDL total | - | - | - |
| 11p11.2-11p11.12 | *MADD|FOLH1|NR1H3* | 125 |  | HDL large | rs2957873 | 0.18 (0.047) | 0.0001 |
| 11p11.2-11p11.12 | *MADD|FOLH1|NR1H3* | 125 |  | HDL medium | - | - | - |
| 11p11.2-11p11.12 | *MADD|FOLH1|NR1H3* | 125 |  | HDL small | rs4752904 | -0.15 (0.06) | 0.014 |
| 11p11.2-11p11.12 | *MADD|FOLH1|NR1H3* | 125 | * | HDL mean size | rs2957873 | 0.026 (0.0058) | 6.90E-06 |
| 11p11.2-11p11.12 | *MADD|FOLH1|NR1H3* | 125 |  | HDL-C by NMR | rs2957873 | 0.67 (0.18) | 0.00016 |
| 11p11.2-11p11.12 | *MADD|FOLH1|NR1H3* | 125 |  | HDL-C assay | rs2957873 | 0.69 (0.19) | 0.00038 |
| 11p11.2-11p11.12 | *MADD|FOLH1|NR1H3* | 125 |  | APOA1 assay | rs326217 | -0.79 (0.27) | 0.004 |
| 11p11.2-11p11.12 | *MADD|FOLH1|NR1H3* | 125 |  | VLDL total | rs12797843 | 1.4 (0.58) | 0.014 |
| 11p11.2-11p11.12 | *MADD|FOLH1|NR1H3* | 125 |  | VLDL large | rs2697920 | -0.067 (0.032) | 0.037 |
| 11p11.2-11p11.12 | *MADD|FOLH1|NR1H3* | 125 |  | VLDL medium | rs12797843 | 0.64 (0.29) | 0.026 |
| 11p11.2-11p11.12 | *MADD|FOLH1|NR1H3* | 125 |  | VLDL small | rs1393794 | 0.5 (0.22) | 0.028 |
| 11p11.2-11p11.12 | *MADD|FOLH1|NR1H3* | 125 |  | VLDL mean size | rs4752894 | -0.19 (0.083) | 0.021 |
| 11p11.2-11p11.12 | *MADD|FOLH1|NR1H3* | 125 |  | TG by NMR | rs12797843 | 0.015 (0.0073) | 0.04 |
| 11p11.2-11p11.12 | *MADD|FOLH1|NR1H3* | 125 |  | TG assay | rs1483121 | -0.018 (0.0076) | 0.019 |
| 12q24.11 | *MVK|MMAB* | 35 |  | LDL large | - | - | - |
| 12q24.11 | *MVK|MMAB* | 35 |  | LDL small | - | - | - |
| 12q24.11 | *MVK|MMAB* | 35 |  | LDL mean size | rs10850435 | -0.016 (0.0077) | 0.044 |
| 12q24.11 | *MVK|MMAB* | 35 |  | IDL total | - | - | - |
| 12q24.11 | *MVK|MMAB* | 35 |  | LDL total | - | - | - |
| 12q24.11 | *MVK|MMAB* | 35 |  | LDL-C assay | - | - | - |
| 12q24.11 | *MVK|MMAB* | 35 |  | APOB assay | rs2241209 | -1.3 (0.57) | 0.02 |
| 12q24.11 | *MVK|MMAB* | 35 |  | HDL total | rs34368092 | 0.3 (0.14) | 0.027 |
| 12q24.11 | *MVK|MMAB* | 35 | * | HDL large | rs2058804 | -0.17 (0.037) | 5.80E-06 |
| 12q24.11 | *MVK|MMAB* | 35 |  | HDL medium | - | - | - |
| 12q24.11 | *MVK|MMAB* | 35 |  | HDL small | rs2058804 | 0.17 (0.06) | 0.0056 |
| 12q24.11 | *MVK|MMAB* | 35 | * | HDL mean size | rs10850435 | -0.02 (0.0046) | 1.80E-05 |
| 12q24.11 | *MVK|MMAB* | 35 | * | HDL-C by NMR | rs2058804 | -0.59 (0.14) | 2.40E-05 |
| 12q24.11 | *MVK|MMAB* | 35 | * | HDL-C assay | rs2058804 | -0.62 (0.15) | 5.50E-05 |
| 12q24.11 | *MVK|MMAB* | 35 |  | APOA1 assay | rs11067376 | 0.94 (0.26) | 0.00026 |
| 12q24.11 | *MVK|MMAB* | 35 |  | VLDL total | rs34368092 | 1.5 (0.72) | 0.041 |
| 12q24.11 | *MVK|MMAB* | 35 |  | VLDL large | - | - | - |
| 12q24.11 | *MVK|MMAB* | 35 |  | VLDL medium | - | - | - |
| 12q24.11 | *MVK|MMAB* | 35 |  | VLDL small | rs11067392 | -0.8 (0.4) | 0.044 |
| 12q24.11 | *MVK|MMAB* | 35 |  | VLDL mean size | - | - | - |
| 12q24.11 | *MVK|MMAB* | 35 |  | TG by NMR | - | - | - |
| 12q24.11 | *MVK|MMAB* | 35 |  | TG assay | - | - | - |
| 16q22.1 | *LCAT* | 10 |  | LDL large | rs2271293 | 9.5 (3.6) | 0.0087 |
| 16q22.1 | *LCAT* | 10 |  | LDL small | rs2271293 | -19 (7.9) | 0.014 |
| 16q22.1 | *LCAT* | 10 |  | LDL mean size | rs2271293 | 0.035 (0.012) | 0.0031 |
| 16q22.1 | *LCAT* | 10 |  | IDL total | rs255049 | 0.031 (0.015) | 0.037 |
| 16q22.1 | *LCAT* | 10 |  | LDL total | - | - | - |
| 16q22.1 | *LCAT* | 10 |  | LDL-C assay | rs255052 | 1.6 (0.52) | 0.0017 |
| 16q22.1 | *LCAT* | 10 |  | APOB assay | - | - | - |
| 16q22.1 | *LCAT* | 10 | * | HDL total | rs1109166 | 0.4 (0.083) | 1.60E-06 |
| 16q22.1 | *LCAT* | 10 |  | HDL large | rs4986970 | -0.36 (0.11) | 0.00086 |
| 16q22.1 | *LCAT* | 10 |  | HDL medium | rs255052 | -0.047 (0.019) | 0.014 |
| 16q22.1 | *LCAT* | 10 | * | HDL small | rs1109166 | 0.3 (0.079) | 0.00015 |
| 16q22.1 | *LCAT* | 10 |  | HDL mean size | rs4986970 | -0.035 (0.013) | 0.0094 |
| 16q22.1 | *LCAT* | 10 | * | HDL-C by NMR | rs1109166 | 0.82 (0.18) | 8.20E-06 |
| 16q22.1 | *LCAT* | 10 | * | HDL-C assay | rs2271293 | 1.2 (0.24) | 1.50E-07 |
| 16q22.1 | *LCAT* | 10 | * | APOA1 assay | rs2271293 | 1.9 (0.39) | 7.40E-07 |
| 16q22.1 | *LCAT* | 10 |  | VLDL total | rs1109166 | 1.2 (0.44) | 0.0065 |
| 16q22.1 | *LCAT* | 10 |  | VLDL large | - | - | - |
| 16q22.1 | *LCAT* | 10 |  | VLDL medium | - | - | - |
| 16q22.1 | *LCAT* | 10 |  | VLDL small | rs1109166 | 0.72 (0.27) | 0.0078 |
| 16q22.1 | *LCAT* | 10 |  | VLDL mean size | - | - | - |
| 16q22.1 | *LCAT* | 10 |  | TG by NMR | rs1109166 | 0.016 (0.0055) | 0.0039 |
| 16q22.1 | *LCAT* | 10 |  | TG assay | - | - | - |
| 19p13.11 | *CILP2|PBX4|NCAN|SF4* | 44 |  | LDL large | rs4808206 | 10 (3.1) | 0.0013 |
| 19p13.11 | *CILP2|PBX4|NCAN|SF4* | 44 |  | LDL small | - | - | - |
| 19p13.11 | *CILP2|PBX4|NCAN|SF4* | 44 |  | LDL mean size | - | - | - |
| 19p13.11 | *CILP2|PBX4|NCAN|SF4* | 44 |  | IDL total | rs4808931 | 0.031 (0.013) | 0.018 |
| 19p13.11 | *CILP2|PBX4|NCAN|SF4* | 44 |  | LDL total | rs739461 | 0.010 (0.0038) | 0.0065 |
| 19p13.11 | *CILP2|PBX4|NCAN|SF4* | 44 |  | LDL-C assay | rs892023 | -1.0 (0.37) | 0.0065 |
| 19p13.11 | *CILP2|PBX4|NCAN|SF4* | 44 |  | APOB assay | rs4808931 | 0.74 (0.33) | 0.023 |
| 19p13.11 | *CILP2|PBX4|NCAN|SF4* | 44 |  | HDL total | rs2238675 | -0.26 (0.092) | 0.0044 |
| 19p13.11 | *CILP2|PBX4|NCAN|SF4* | 44 |  | HDL large | - | - | - |
| 19p13.11 | *CILP2|PBX4|NCAN|SF4* | 44 | * | HDL medium | rs2228603 | -0.11 (0.025) | 4.90E-06 |
| 19p13.11 | *CILP2|PBX4|NCAN|SF4* | 44 |  | HDL small | rs2163813 | 0.20 (0.068) | 0.0025 |
| 19p13.11 | *CILP2|PBX4|NCAN|SF4* | 44 |  | HDL mean size | rs12610185 | 0.018 (0.0084) | 0.036 |
| 19p13.11 | *CILP2|PBX4|NCAN|SF4* | 44 |  | HDL-C by NMR | rs8108647 | -0.38 (0.18) | 0.038 |
| 19p13.11 | *CILP2|PBX4|NCAN|SF4* | 44 |  | HDL-C assay | - | - | - |
| 19p13.11 | *CILP2|PBX4|NCAN|SF4* | 44 |  | APOA1 assay | - | - | - |
| 19p13.11 | *CILP2|PBX4|NCAN|SF4* | 44 |  | VLDL total | rs12610185 | -2.2 (0.6) | 0.00029 |
| 19p13.11 | *CILP2|PBX4|NCAN|SF4* | 44 |  | VLDL large | - | - | - |
| 19p13.11 | *CILP2|PBX4|NCAN|SF4* | 44 |  | VLDL medium | rs2304128 | -1.1 (0.3) | 0.00014 |
| 19p13.11 | *CILP2|PBX4|NCAN|SF4* | 44 |  | VLDL small | rs12610185 | -0.94 (0.37) | 0.011 |
| 19p13.11 | *CILP2|PBX4|NCAN|SF4* | 44 |  | VLDL mean size | rs11085270 | 0.27 (0.099) | 0.0058 |
| 19p13.11 | *CILP2|PBX4|NCAN|SF4* | 44 |  | TG by NMR | rs12610185 | -0.026 (0.0076) | 0.00075 |
| 19p13.11 | *CILP2|PBX4|NCAN|SF4* | 44 |  | TG assay | rs12610185 | -0.029 (0.0098) | 0.0028 |

^+^Pmax=0.05/(N locus SNPs tested)
