# Supplementary material for: Forty-Three Loci Associated with Plasma Lipoprotein Size, Concentration, and Cholesterol Content in Genome-Wide Analysis
Source: PLoS Genet. 2009 Nov 20;5(11):e1000730. doi: 10.1371/journal.pgen.1000730 (PMC2777390; doi:10.1371/journal.pgen.1000730)
Supplement: Table S10 — Lipoprotein associations in the fasting sub-sample at loci in previous lipid fraction GWAS. (0.16 MB DOC) [file pgen.1000730.s014.doc]

Table S10. Lipoprotein associations at loci in previous lipid fraction GWAS (fasting WGHS subsample)

| Locus | candidate gene(s) | N snps | P<Pmax^+^ | lipoprotein | best SNP | beta (se) | p-value |
| --- | --- | --- | --- | --- | --- | --- | --- |
| 5q33.3 | *TIMD4|HAVCR1* | 27 |  | LDL large | rs7706174 | -14 (3.5) | 9.00E-05 |
| 5q33.3 | *TIMD4|HAVCR1* | 27 |  | LDL small | rs7706174 | 16 (7.6) | 3.30E-02 |
| 5q33.3 | *TIMD4|HAVCR1* | 27 |  | LDL mean size | rs7706174 | -0.036 (0.012) | 1.90E-03 |
| 5q33.3 | *TIMD4|HAVCR1* | 27 |  | IDL total | rs1363232 | -0.041 (0.014) | 3.00E-03 |
| 5q33.3 | *TIMD4|HAVCR1* | 27 |  | LDL total | rs1363232 | -0.012 (0.004) | 2.40E-03 |
| 5q33.3 | *TIMD4|HAVCR1* | 27 |  | LDL-C assay | rs2277025 | 1.6 (0.44) | 2.30E-04 |
| 5q33.3 | *TIMD4|HAVCR1* | 27 | * | APOB assay | rs1363232 | -1.4 (0.35) | 4.00E-05 |
| 5q33.3 | *TIMD4|HAVCR1* | 27 |  | HDL total | - | - | - |
| 5q33.3 | *TIMD4|HAVCR1* | 27 |  | HDL large | rs6873053d | 0.16 (0.077) | 3.50E-02 |
| 5q33.3 | *TIMD4|HAVCR1* | 27 |  | HDL medium | rs4704810 | 0.044 (0.016) | 5.90E-03 |
| 5q33.3 | *TIMD4|HAVCR1* | 27 |  | HDL small | - | - | - |
| 5q33.3 | *TIMD4|HAVCR1* | 27 |  | HDL mean size | - | - | - |
| 5q33.3 | *TIMD4|HAVCR1* | 27 |  | HDL-C by NMR | - | - | - |
| 5q33.3 | *TIMD4|HAVCR1* | 27 |  | HDL-C assay | rs2279804 | -0.39 (0.17) | 2.50E-02 |
| 5q33.3 | *TIMD4|HAVCR1* | 27 |  | APOA1 assay | - | - | - |
| 5q33.3 | *TIMD4|HAVCR1* | 27 |  | VLDL total | rs1354163 | -1.7 (0.53) | 1.60E-03 |
| 5q33.3 | *TIMD4|HAVCR1* | 27 |  | VLDL large | rs1354163 | -0.13 (0.047) | 7.70E-03 |
| 5q33.3 | *TIMD4|HAVCR1* | 27 |  | VLDL medium | rs1354163 | -0.63 (0.27) | 1.80E-02 |
| 5q33.3 | *TIMD4|HAVCR1* | 27 |  | VLDL small | rs1363232 | -0.73 (0.25) | 3.80E-03 |
| 5q33.3 | *TIMD4|HAVCR1* | 27 |  | VLDL mean size | - | - | - |
| 5q33.3 | *TIMD4|HAVCR1* | 27 |  | TG by NMR | rs1354163 | -0.025 (0.0066) | 1.50E-04 |
| 5q33.3 | *TIMD4|HAVCR1* | 27 |  | TG assay | rs1354163 | -0.03 (0.0084) | 3.00E-04 |
| 16q22.1 | *LCAT* | 10 |  | LDL large | - | - | - |
| 16q22.1 | *LCAT* | 10 |  | LDL small | rs7200210 | 29 (14) | 3.90E-02 |
| 16q22.1 | *LCAT* | 10 |  | LDL mean size | rs7200210 | -0.051 (0.022) | 1.70E-02 |
| 16q22.1 | *LCAT* | 10 |  | IDL total | rs255049 | 0.046 (0.017) | 8.00E-03 |
| 16q22.1 | *LCAT* | 10 |  | LDL total | - | - | - |
| 16q22.1 | *LCAT* | 10 |  | LDL-C assay | rs255052 | 1.9 (0.62) | 2.00E-03 |
| 16q22.1 | *LCAT* | 10 |  | APOB assay | rs255049 | 0.92 (0.44) | 3.40E-02 |
| 16q22.1 | *LCAT* | 10 | * | HDL total | rs1109166 | 0.47 (0.097) | 1.40E-06 |
| 16q22.1 | *LCAT* | 10 |  | HDL large | rs2271293 | 0.16 (0.067) | 1.80E-02 |
| 16q22.1 | *LCAT* | 10 |  | HDL medium | - | - | - |
| 16q22.1 | *LCAT* | 10 |  | HDL small | rs1109166 | 0.32 (0.092) | 5.10E-04 |
| 16q22.1 | *LCAT* | 10 |  | HDL mean size | rs4986970 | -0.032 (0.016) | 4.10E-02 |
| 16q22.1 | *LCAT* | 10 | * | HDL-C by NMR | rs1109166 | 0.83 (0.22) | 1.40E-04 |
| 16q22.1 | *LCAT* | 10 | * | HDL-C assay | rs2271293 | 1.2 (0.27) | 1.90E-05 |
| 16q22.1 | *LCAT* | 10 | * | APOA1 assay | rs2271293 | 1.8 (0.45) | 7.10E-05 |
| 16q22.1 | *LCAT* | 10 |  | VLDL total | rs255052 | 1.8 (0.57) | 1.90E-03 |
| 16q22.1 | *LCAT* | 10 |  | VLDL large | rs1109166 | 0.13 (0.046) | 6.20E-03 |
| 16q22.1 | *LCAT* | 10 |  | VLDL medium | rs255052 | 0.62 (0.29) | 3.10E-02 |
| 16q22.1 | *LCAT* | 10 |  | VLDL small | rs255052 | 1.1 (0.35) | 1.40E-03 |
| 16q22.1 | *LCAT* | 10 |  | VLDL mean size | - | - | - |
| 16q22.1 | *LCAT* | 10 |  | TG by NMR | rs1109166 | 0.022 (0.0065) | 8.30E-04 |
| 16q22.1 | *LCAT* | 10 |  | TG assay | rs1109166 | 0.019 (0.0082) | 1.80E-02 |
| 20q12 | *MAFB* | 31 |  | LDL large | rs6102085 | 9.1 (2.9) | 1.50E-03 |
| 20q12 | *MAFB* | 31 |  | LDL small | rs2865892 | -32 (9.1) | 5.30E-04 |
| 20q12 | *MAFB* | 31 |  | LDL mean size | rs2865892 | 0.032 (0.014) | 2.20E-02 |
| 20q12 | *MAFB* | 31 |  | IDL total | rs1014748 | -0.057 (0.019) | 3.30E-03 |
| 20q12 | *MAFB* | 31 | * | LDL total | rs2865892 | -0.025 (0.0059) | 2.10E-05 |
| 20q12 | *MAFB* | 31 |  | LDL-C assay | rs6102085 | 1.5 (0.45) | 5.70E-04 |
| 20q12 | *MAFB* | 31 |  | APOB assay | rs2865892 | -1.9 (0.51) | 2.20E-04 |
| 20q12 | *MAFB* | 31 |  | HDL total | rs6016408 | 0.2 (0.084) | 1.80E-02 |
| 20q12 | *MAFB* | 31 |  | HDL large | rs2865879 | -0.11 (0.046) | 1.80E-02 |
| 20q12 | *MAFB* | 31 |  | HDL medium | - | - | - |
| 20q12 | *MAFB* | 31 |  | HDL small | - | - | - |
| 20q12 | *MAFB* | 31 |  | HDL mean size | rs2865892 | 0.019 (0.0083) | 1.90E-02 |
| 20q12 | *MAFB* | 31 |  | HDL-C by NMR | rs2425421 | -0.36 (0.18) | 4.30E-02 |
| 20q12 | *MAFB* | 31 |  | HDL-C assay | rs2865879 | -0.44 (0.19) | 1.90E-02 |
| 20q12 | *MAFB* | 31 |  | APOA1 assay | - | - | - |
| 20q12 | *MAFB* | 31 |  | VLDL total | rs2865892 | -1.5 (0.6) | 1.50E-02 |
| 20q12 | *MAFB* | 31 |  | VLDL large | rs1076759 | -0.13 (0.062) | 4.00E-02 |
| 20q12 | *MAFB* | 31 |  | VLDL medium | rs2865892 | -0.65 (0.3) | 3.00E-02 |
| 20q12 | *MAFB* | 31 |  | VLDL small | rs6102085 | 0.8 (0.25) | 1.70E-03 |
| 20q12 | *MAFB* | 31 |  | VLDL mean size | - | - | - |
| 20q12 | *MAFB* | 31 |  | TG by NMR | rs1076759 | -0.028 (0.0087) | 1.50E-03 |
| 20q12 | *MAFB* | 31 |  | TG assay | rs1076759 | -0.025 (0.011) | 2.20E-02 |

^+^Pmax=0.05/(N locus SNPs tested)
